# Colchicine-BODIPY Probes: Evidence for the Involvement of Intracellular Membranes in the Targeting of Colchicine to Tubulin

**DOI:** 10.1021/acsptsci.4c00730

**Published:** 2025-06-13

**Authors:** Michal Jurášek, Eduarda Dráberová, Jiří Řehulka, Soňa Gurská, Aleksandra Ivanova, Pavel Polishchuk, Kateřina Ječmeňová, Jan Fähnrich, Anna Marešová, Jan Tauchen, Atilio Reyes Romero, Alexander Dömling, Marián Hajdúch, Pavel B. Drašar, Pavel Dráber, Petr Džubák

**Affiliations:** 1 Department of Chemistry of Natural Compounds, 52735 University of Chemistry and Technology Prague (UCT Prague), Technická 5, Praha 6 CZ-166 28, Czech Republic; 2 Department of Analytical Chemistry, 52735 University of Chemistry and Technology Prague (UCT Prague), Technická 5, Praha 6 CZ-166 28, Czech Republic; 3 Department of Biology of Cytoskeleton, 89219 Institute of Molecular Genetics of the Czech Academy of Sciences, Vídeňská 1083, Praha 4 CZ-142 20, Czech Republic; 4 Institute of Molecular and Translational Medicine (IMTM), Faculty of Medicine and Dentistry, Palacký University and University Hospital in Olomouc, Hněvotínská 1333/5, Olomouc CZ-779 00, Czech Republic; 5 Department of Food Science, Faculty of Agrobiology, Food and Natural Resources, Czech University of Life Sciences Prague, Kamýcká 129, Praha 6 CZ-165 00, Czech Republic; 6 Laboratory of Experimental Medicine, IMTM, University Hospital Olomouc, Hněvotínská 976/3, Olomouc CZ-779 00, Czech Republic

**Keywords:** colchicine, BODIPY, cytotoxicity, flow-cytometry, cell-cycle, tubulin polymerization, intracellular membranes, fluorescence microscopy, in silico modeling

## Abstract

The natural product colchicine (**Col**) is a medication used to treat severe inflammatory conditions. Although its mechanism of action at the level of the cytoskeleton is known, its subcellular distribution has not yet been properly studied. In this work, we present the first rational approach to assess the intracellular localization and biological activity of this alkaloid. We conjugated **Col** to green-emitting BODIPY dyes (**CBs**) with alternative linkers of different lengths (**CB1**–**CB12**) via different types of linkages. Connections of **Col** with BODIPY generally reduced its cytotoxicity to different levels depending on the type of linker. From the analysis of **CB** effects on cytotoxicity, cell cycle, and tubulin polymerization, we selected the most potent substances for fluorescence microscopy. Treatment of cells with 10 μM conjugates for 15 h showed different effects on microtubule organization. Live-cell imaging revealed that **CBs** rapidly associated with cellular membranes. Double label experiments unveiled that the **CB4**, which was the most effective in inhibiting tubulin polymerization, binds to the endoplasmic reticulum (ER) and mitochondria. *In silico* modeling and SPR analyses confirmed the high potency of **CB4** to bind to the colchicine site on tubulin.

## Graphical abstract

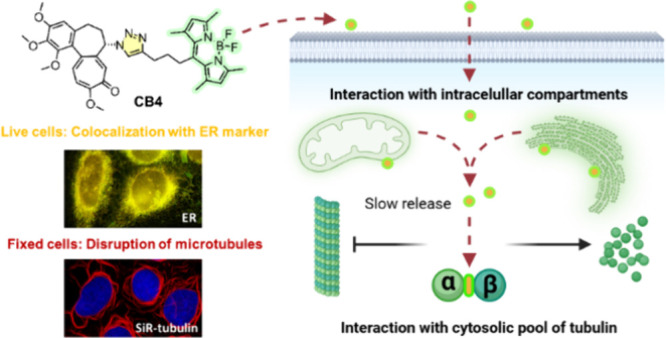

Colchicine (**Col**), the main alkaloid of the poisonous plant meadow saffron (*Colchicum autumnale*), is a unique anti-inflammatory drug approved by the FDA in 2009. **Col** is indicated primarily for the treatment and prophylaxis of gout and familial Mediterranean fever. Although, Col has been tested to suppress excessive inflammation in COVID-19 infection^1,2^ multiple studies have led the WHO to strongly advice against its use for the treatment of nonsevere cases of COVID-19.^3^

Although **Col** is not clinically used to treat cancer due to its toxicity, it does exhibit significant antiproliferative effects. Several **Col** semisynthetics are less toxic than **Col** itself, and research is ongoing into effective, less toxic colchicine-derived compounds with potential drug delivery strategies to directly target multiple solid tumors.^4^

The molecular mechanism of **Col** involves binding to free tubulin dimers, which, once incorporated into microtubules, block subsequent microtubule polymerization^5,6^ (Figure 1). However, the extent to which this mechanism contributes to **Col** effects at low therapeutic doses is not fully understood. **Col** demonstrates multiple effects on cellular function, including inhibition of neutrophil adhesion, suppression of the release of chemotactic agents, alteration of neutrophil deformability, and modulation of leukocyte-mediated inflammatory activities.^7^ Recently, **Col** has been shown to inhibit myeloid cells through an indirect mechanism involving selective activation of hepatocytes and release of hepatokines into plasma.^8^

**Figure 1:**
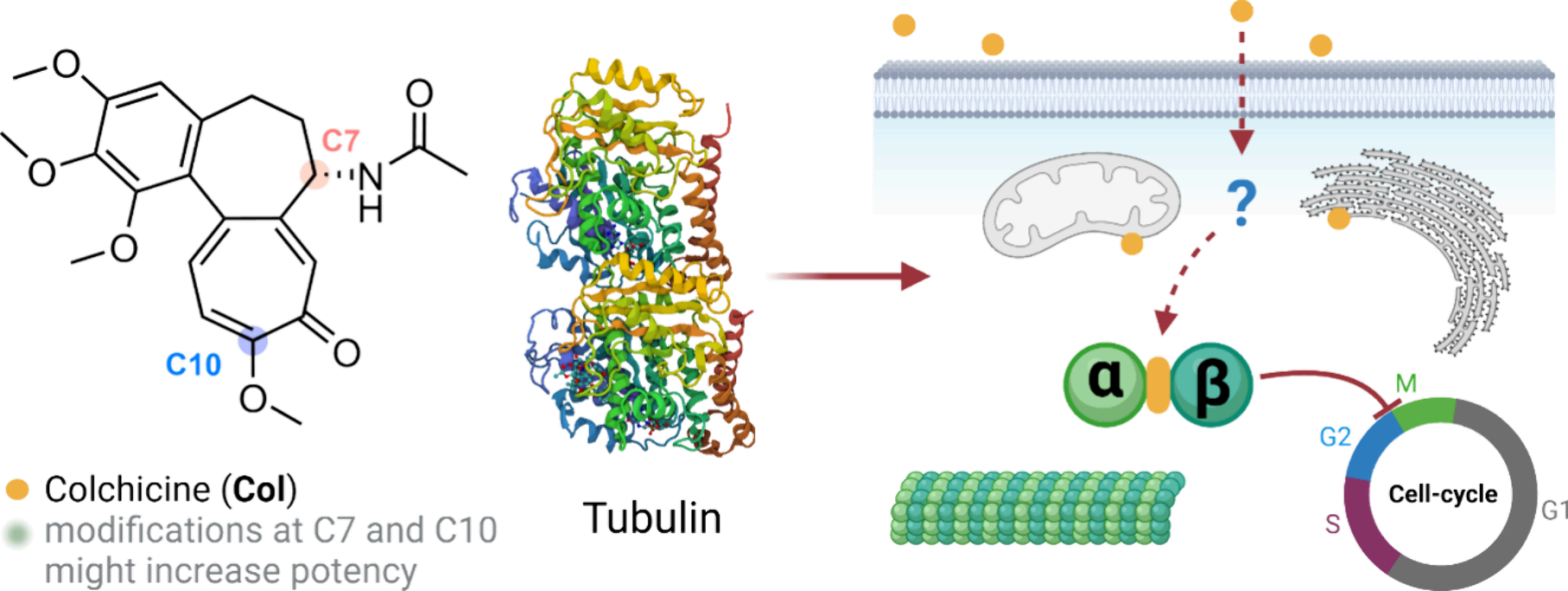
Structure of **Col**, amenable modification sites for chemical transformations and drawing depicting the principle of its biological activity. Created with Biorender.com.

A critical gap in our understanding concerns the intracellular distribution and trafficking of **Col**. Previous studies have suggested that **Col** may associate with cellular membranes,^9^ potentially affecting its bioavailability and interaction with tubulin. *In vitro* experiments with lipid-conjugated colchicine demonstrated that membrane association could make the drug inaccessible to tubulin.^10^ This led to the hypothesis that in cells, **Col** may initially associate with membranes before slowly releasing into the cytosol to interact with tubulin.^8^

Previous studies to track **Col** cellular distribution have utilized various fluorescent labels. Fluorescein-colchicine with preserved biological activities provided only general cytoplasmic fluorescence in cultured fibroblasts.^11^ In contrast, fluorescein-thiocolchicine, when loaded into cells that were subsequently fixed, successfully stained microtubules.^12^ However, its distribution in living cells remained unexamined. Other studies employed nitrobenzofurazan (NBD) as a fluorescent label, but these probes had limitations in tracking **Col** real-time cellular dynamics.^13–15^

Boron-dipyrromethene (BODIPY) dyes are characterized by charge neutrality, stability in physiological environments, high fluorescence quantum yield, low photodegradation, toxicity and sensitivity to different pH.^16^ A disadvantage may be low solubility in aqueous solutions. They are used in research on supramolecular systems,^17^ chemosensors of molecules^18^ and ions,^19^ fluorescent sensors for labeling cellular organelles,^20^ and photosensitizers for photodynamic therapy.^21^ Due to their low polarity, BODIPYs are one of the best tools for monitoring the interactions of lipoid substances using fluorescence microscopy. These fluorophores have been successfully used in fluorescence imaging of a number of natural substances, including thapsigargins,^22^ phorbol esters,^23^ betulinic acid,^24^ steroids^25^ and sugars.^26^ While BODIPY-labeled mitotic inhibitors such as paclitaxel^27–29^ and vindoline^30^ have been reported, previous attempts to label **Col** with BODIPY limited insight into its cellular distribution.^31^ To address these limitations, we have developed and characterized a new series of colchicine-BODIPY conjugates (**CBs**) with improved properties for visualizing of colchicine cellular fate. Although the prepared **CBs** were less cytotoxic than colchicine, they were capable of binding tubulin dimers and disrupted microtubule organization in cells. Through comprehensive biological evaluation and advanced imaging techniques, we provide the first experimental evidence for **Col** rapid association with intracellular membrane compartments, suggesting a more complex mechanism of action then previously recognized. The subsequent disruption of microtubules could be the result of a slow release of colchicine from membranes and its interaction with cytosolic tubulin.

## Results and Discussion

### Design and Synthesis of BODIPY Conjugates

The **CBs** were designed to target two key positions on **Col** that allow the modification without significant loss of its activity: C-7 and C-10 (Figure 1). Three types of linkages were applied: (a) C-7 amides, (b) C-7 1,4-disubstituted 1,2,3-triazoles, and (c) C-10 primary/secondary amines or secondary amides.

First, BODIPY fluorophores with different lengths of the linker and reactive moiety were synthesized (Scheme 1). The dyes intended for amide syntheses were terminated with a carboxyl moiety (Scheme 1A). These compounds were synthesized from monoesters of corresponding dioic acids in 3 consecutive steps.^32^ Dyes used for the synthesis of secondary, tertiary and quaternary amines were synthesized from difluoro-1,3,5,7-tetramethyl-4-bora-3a,4a-diaza-*s*-indacene-8-yl)­butyl bromide **B3** ^33^ by substitution reactions yielding amines **B4**, **B5** and **B6** (Scheme 1B).

**Scheme 1:**
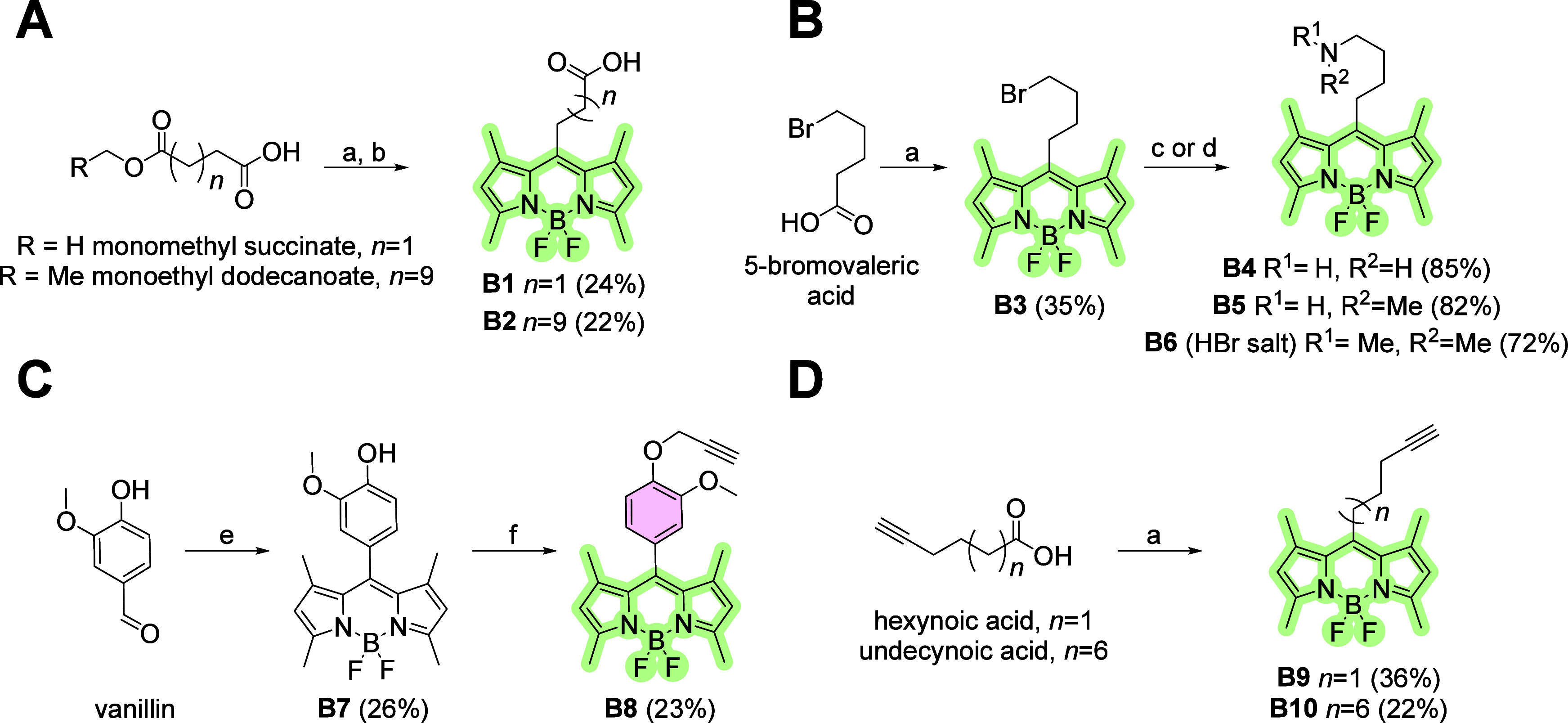
Synthesis of Functionalized BODIPY Fluorophores^a^

a (A) carboxyl-terminated, (B) amines, and (C, D) alkynes. *Reagents and conditions*: a) *i.* (COCl)_2_, DCM, DMF (cat.), ON, RT; *ii.* 2,4-dimethylpyrrole, DCM, ON, RT; *iii.* TEA, 30 min, then BF_3_·Et_2_O, RT, 5 h; b) LiOH, THF, H_2_O, RT→80°C; c) for **B4** – *i.* NaN_3_, DMF, 70°C, 3 h; *ii.* PPh_3_, H_2_O, THF, ON, RT; d) for **B5** – MeNH_2_ in THF, 100°C, 3 h, then 50°C, ON, and for **B6** – Me_2_NH in THF, AcCN, RT, ON; e) *i.* 2,4-dimethylpyrrole, TFA (cat.), DCM, ON, RT; *ii.* DDQ, RT, 2 h; *iii.* TEA, 1 h, then BF_3_·Et_2_O, RT, 5 h; f) propargyl bromide in toluene, K_2_CO_3_, DMF, 50°C, 19 h.

Two types of dyes for CuAAC chemistry were used (Scheme 1C, **D**). The BODIPY **B8** was derived from vanillin while **B9** and **B10** from aliphatic terminal alkynes. Synthetic protocols for these preparations were similar to those described previously.^34,35^

Second, the **Col** derived precursors **C1** (C-7 amine) and **C2** (C-7 azide) were prepared (Scheme 2A). To obtain precursor **C2** the acetamide group at C-7 was bocylated, the acetyl was removed by alkaline hydrolysis and the Boc group was finally cleaved under acidic conditions using TFA.^36^ Azide **C2** was prepared from **C1** by a copper-catalyzed diazo transfer reaction.^37,38^

**Scheme 2:**
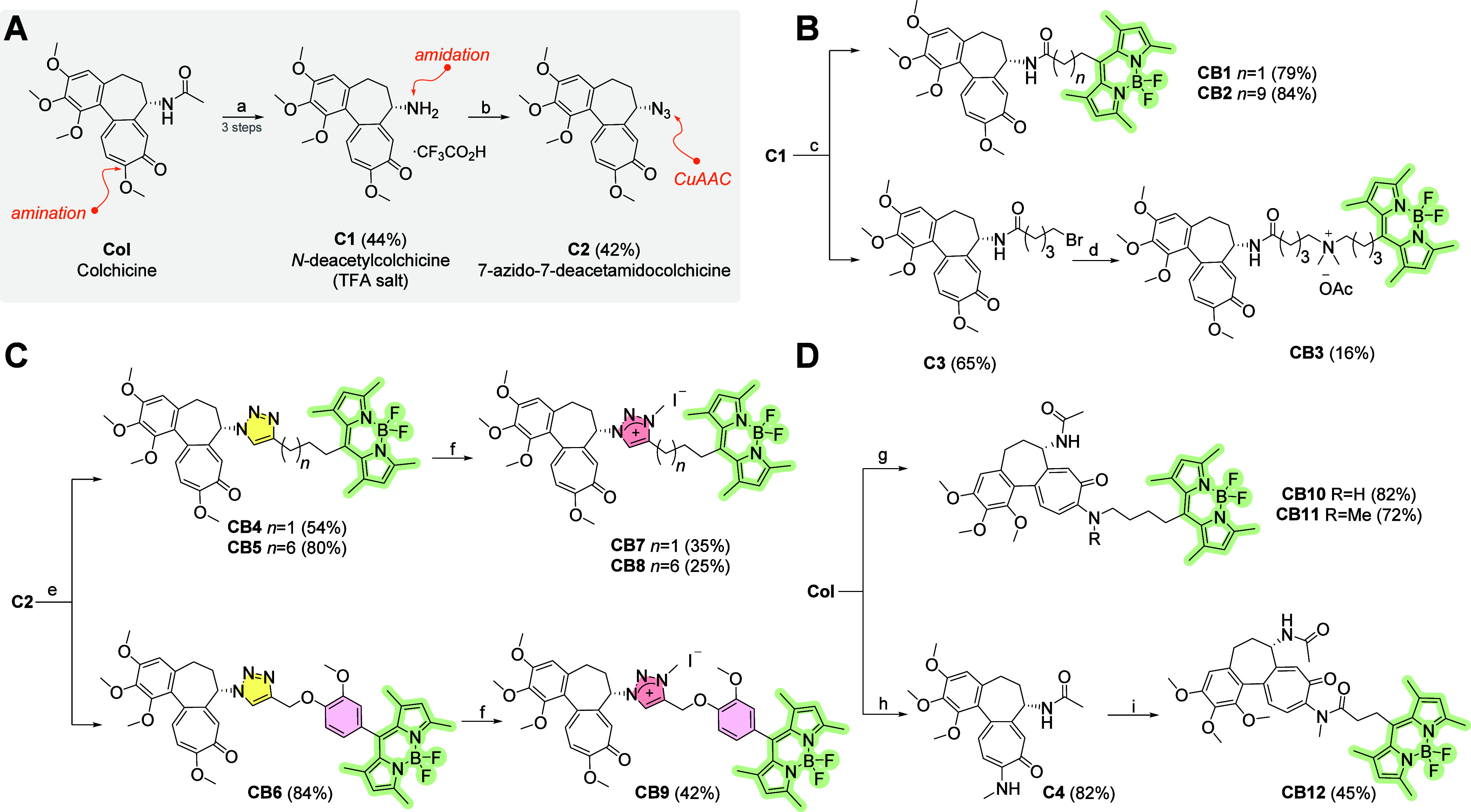
(A) Preparation of *N-*Deacetylcolchicine **C1** and 7-Azido-7-deacetamidocolchicine **C2**, (B) Syntheses of C-7 Colchicine-BODIPY Amides, (C) C-7 Colchicine-BODIPY Clickates, and (D) C-10 Linked **CBs** [Fn sch2-fn1]

a
*Reagents and conditions*: a) *i.* Boc_2_O, 4-DMAP, TEA, AcCN, RT, 30 min, then, 100°C, 4 h → RT; *ii.* NaOMe, MeOH, RT, 2 h; *iii.* TFA, DCM, RT, 2 h; b) *i.* preparation of TfN_3_: NaN_3_ (aq.), DCM, 0°C, 20 min cooled, then Tf_2_O, 2 h; *ii.* TfN_3_, K_2_CO_3_, CuSO_4_·5H_2_O (aq.), MeOH, 0°C → RT, ON; c) **B1** and **B2** for **CB1** and **CB2**, resp. and 5-bromovaleric acid for **C3**, EDCI, 4-DMAP, HOBt, DCM, RT, ON; d) **B6**, AcCN, 70°C, 3 h → 55°C, ON; e) **B8**, **B9** or **B10**, CuSO_4_·5H_2_O (aq.), sodium ascorbate (aq.), TBTA, *tert-*BuOH, MW-85°C, 20 min; f) MeI, AcCN, RT, 4 d; g) **B4**/**B5**, MeOH, 70°C, ON; h) MeNH_2_ in THF, MeOH, RT, 1 h; (i) **B1**, HBTU, DCM, EDIPA, RT, ON.

Fluorescent C-7 amides **CB1** (C3 linker) and **CB2** (C11 linker) were synthesized from **C1** and **B1/B2** using carbodiimide chemistry with EDCI reagent (Scheme 2B). Analogue **CB3** was synthesized from amide **C3** and **B6** by a quaternization reaction (Scheme 2B). The yield of **CB3** was very low (16%). Although the starting material was not completely consumed according to TLC, another reason was the difficult separation of the product.

The synthesis of fluorescent 1,4-disubstituted-1,2,3-triazoles **CB4**, **CB5** and **CB6** by CuAAC was performed using deep-rooted method using CuSO_4_ and sodium ascorbate^37,39^ (Scheme 2C). The subsequent quaternization reaction of triazoles by methyl iodide was performed by the synthetic protocol reported previously.^40–42^ The cationic *N*-methyl triazolium probes **CB7**, **CB8** and **CB9** were obtained in rather moderate yields (Scheme 2C).

The C-10 conjugates were based on the amination^43^ of **Col** at the C-10 methoxide group with **B5** and **B6** (Scheme 2D) yielding secondary and tertiary amine **CB10**, **CB11**, respectively (Scheme 2D). Amide **CB12** was synthesized from precursor **C4** ^44^ by acylation with **B1** using HBTU (Scheme 2D).

All newly synthesized compounds were thoroughly characterized by analytical methods (Supporting Information, Figures S1–S19). The calculated LogP values indicate that the cationic probes **CB3**, **CB7** and **CB9** are close to the polarity of **Col**. The rest of the **CBs** have rather lipophilic nature (Figure 2 and Table S2). The purity of all tested compounds was ≥ 95% according to HPLC (Figures S7, S8, S10–S19D). Since the substances were prepared as lyophilizates, their handling was problem-free. **CBs** were stable in solution and no decomposition was observed during their storage.

**Figure 2:**
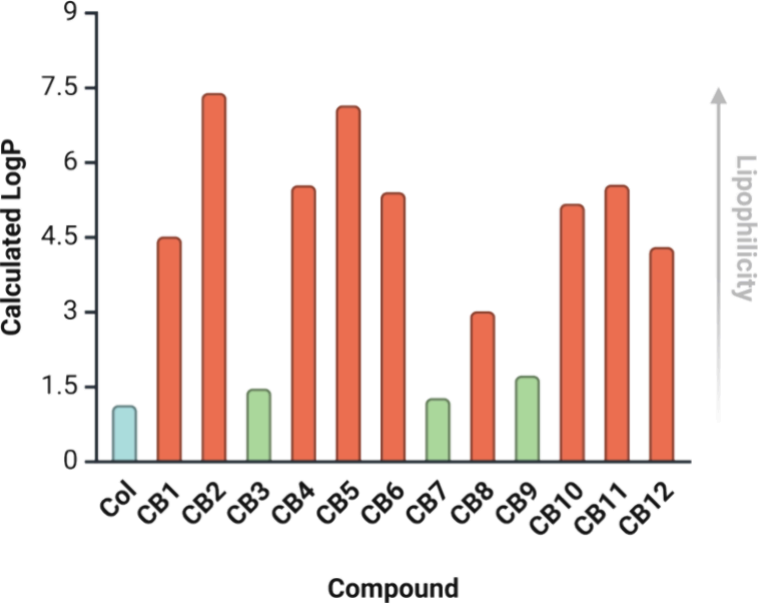
Calculated LogP of CBs. LogP is calculated for colchicine derivatives or parent cations, not for the salt, if applicable. Created with Biorender.com.

The absorbance and fluorescence spectra are shown in Supporting Information in Figure S21 and the characteristics are summarized in Table 1 and Table 2. The spectra show the absorption coefficient calculated from the more concentrated solution measured in the absorption cell. The absorption coefficient calculated from the absorption spectrum of the solution used for fluorescence measurement is also plotted for comparison. Corrected excitation and emission curves are in relative unit-less values. In most cases, the scaled corrected excitation spectrum is lower in the 300–400 nm range compared to the absorption spectrum. The quantum yields are presented for the two excitation wavelengths in Table 2. For the BODIPY excitation wavelength around 500 nm, the quantum yield is higher in comparison with the fluorescence excited at around 340 nm, where colchicines absorb. It appears that the energy absorbed by the **Col** moiety is not fully transferred to the BODIPY fluorophore.

**Table 1:** Absorption and Fluorescence Characteristics of Studied Compounds^a^

compd.	λ_Amax_ [nm]	ε [L mol^–1^ cm^–1^]	λ_Fmax_ [nm]	λ_Ex_ [nm]	λ_Em_ [nm]
**CB1**	499	137,900	507	308, 362, 472, 498	507, 539
**CB2**	497	79,800	504	480, 497	503, 530
**CB3**	499	62,800	506	470, 499	505, 530
**CB4**	497	75,100	504	470, 497	504, 530
**CB5**	496	68,600	503	470, 497	503, 530
**CB6**	500	78,800	508	480, 500	509, 530
**CB7**	500	101,900	507	480, 500	508, 530
**CB8**	497	103,000	504	480, 497	504, 530
**CB9**	501	70,800	509	470, 501	501, 510, 540
**CB10**	498	85,000	505	470, 498	505, 530
**CB11**	497	80,500	504	470, 497	503, 530
**CB12**	500	69,900	508	470, 500	509, 530

a λ_Amax_, ε – wavelength and absorption coefficient at absorption maximum, λ_Fmax_ – wavelength of a maximum of corrected emission spectra; λ_Ex_, λ_Em_ – wavelengths at which emission and excitation spectra were recorded.

**Table 2:** Fluorescence Quantum Yields of Studied Compounds^a^

compd.	λ_Ex_ [nm]	*Y*_f_	λ_Ex_ [nm]	*Y*_f_
**CB1**	499	0.74	350	0.39
**CB2**	497	0.76	350	0.27
**CB3**	499	0.59	351	0.33
**CB4**	497	0.81	350	0.37
**CB5**	496	0.78	349	0.27
**CB6**	500	0.57	351	0.27
**CB7**	500	0.64	351	0.29
**CB8**	497	0.70	350	0.23
**CB9**	501	0.48	352	0.25
**CB10**	498	0.22	356	0.17
**CB11**	497	0.08	370	0.03
**CB12**	500	0.07	369	0.03

a λ_Ex_ – excitation wavelengths, *Y*_f_ – relative quantum yield of fluorescence.

The emission spectra of **CB12** change slightly with excitation wavelength moving in the region of the first absorption band. This band in the corrected excitation spectrum is also narrower compared to the absorption band. This may be due to the presence of various conformations with different fluorescence efficiencies.

### Cytotoxic Profile on a Panel of Cell Lines

Colchicine cytotoxic activity was tested on the panel of seven cancer cell lines (CCRF-CEM, K562, CEM-DNR, K562-TAX, HCT116, HCT116p53–/–, and U2OS) and two normal human fibroblasts (BJ and MRC-5). The IC_50_ values after 72 h treatment are shown in Table 3. **Col** induced potent cytotoxic effect at nanomolar/submicromolar concentrations against most cell lines except BJ nontumor cells. The cytotoxicity of **Col** was partially reduced in drug resistant cell lines CEM-DNR (daunorubicin-resistant) and K562-TAX (paclitaxel-resistant) expressing proteins responsible for multidrug resistance (MDR), P-glycoprotein (Pgp-1) and Lung Resistance Protein (LRP), respectively.^45^ ^,^ Overcoming MDR phenomenon is a key aspect of cancer chemotherapy research. ABC (ATP-binding cassette) transporters have a key function in MDR, where cancer cells develop resistance to a relatively broad spectrum of drugs. The substrates of these proteins are neither structurally nor pharmacologically similar. Pgp-1 expressed by K562-TAX is a well-known member of this family and has a number of structurally diverse substrates. Similar cases are Multidrug Resistance-Related Protein 1 (MRP1), LRP, and Breast Cancer Resistance Protein (BCRP) expressed by CEM-DNR. **Col** is a known ligand of these proteins.^38,46^

**Table 3:** Summary of Cytotoxic Activities (IC_50_, μM)

compd. cell line	**IC**_**50**_ **(μM)**												
	Col	CB1	CB2	CB3	CB4	CB5	CB6	CB7	CB8	CB9	CB10	CB11	CB12
CCRF-CEM	0.011 ± 0.00031	0.40 ± 0.018	0.33 ± 0.005	9.33 ± 0.952	0.021 ± 0.0009	0.42 ± 0.014	0.14 ± 0.010	0.84 ± 0.058	7.11 ± 1.141	4.81 ± 0.641	7.11 ± 0.651	2.50 ± 0.567	5.54 ± 0.475
CEM-DNR	1.05 ± 0.120	2.07 ± 0.212	1.45 ± 0.060	>50	0.36 ± 0.014	1.39 ± 0.139	0.50 ± 0.037	1.34 ± 0.2288	6.53 ± 0.458	6.49 ± 0.639	11.09 ± 0.959	5.49 ± 0.935	36.03 ± 2.042
K562	0.014 ± 0.001	0.45 ± 0.054	0.19 ± 0.040	9.50 ± 2.370	0.088 ± 0.013	0.50 ± 0.122	0.026 ± 0.001	0.77 ± 0.0224	7.83 ± 0.892	7.69 ± 0.450	10.82 ± 0.431	1.85 ± 0.204	4.25 ± 0.89
K562-TAX	1.80 ± 0.062	1.47 ± 0.361	0.58 ± 0.035	40.11 ± 1.075	0.19 ± 0.021	0.90 ± 0.026	0.38 ± 0.034	0.62 ± 0.084	6.35 ± 0.335	5.78 ± 0.561	14.85 ± 2.939	5.18 ± 0.198	8.07 ± 0.706
RI	114.00	4.16	3.90	>4.79	5.05	2.49	5.30	1.22	0.86	0.98	1.45	2.45	4.50
HCT116	0.024 ± 0.002	0.50 ± 0.032	>50	31.61 ± 4.544	0.10 ± 0.004	0.64 ± 0.065	0.43 ± 0.0325	1.51 ± 0.33	8.81 ± 1.937	8.27 ± 1.432	35.77 ± 5.909	4.93 ± 0.729	8.09 ± 0.268
HCT116p53–/–	0.027 ± 0.006	0.58 ± 0.063	>50	36.36 ± 5.031	0.12 ± 0.012	0.77 ± 0.063	0.38 ± 0.057	1.43 ± 0.162	9.36 ± 0.870	8.56 ± 0.365	>50	5.52 ± 0.762	8.35 ± 0.549
U2OS	0.022 ± 0.0004	0.64 ± 0.019	>50	>50	0.14 ± 0.004	1.04 ± 0.141	0.48 ± 0.058	1.96 ± 0.096	11.88 ± 1.106	>50	>50	4.62 ± 1.230	9.12 ± 0.174
MRC-5	0.040 ± 0.008	5.69 ± 0.639	>50	>50	>50	>50	>50	>50	22.09 ± 1.223	>50	>50	>50	>50
BJ	>50	8.35 ± 0.419	>50	>50	>50	>50	>50	>50	36.92 ± 5.68	>50	>50	>50	>50
HUVEC	<0.0122	0.67 ± 0.033	0.36 ± 0.035	>50	<0.0122	0.45 ± 0.012	<0.0122	1.40 ± 0.104	23.54 ± 1.29	40.15 ± 2.93	32.26 ± 0.42	5.52 ± 0.29	8.24 ± 0.77
SI	>1276.53	13.66	>1.66	>1.83	>533.05	>74.18	>171.70	>38.40	3.28	>3.15	>1.63	>12.87	>7.07

Cytotoxic activity was determined by MTS assay following 3-day incubation. Values represent the means of IC_50_ from 3 independent experiments with SD. The resistance index was calculated as RI = (IC_50_ of resistant cell lines, CEM-DNR, K562-TAX)/(IC_50_ of non-resistant counterparts, CCRF-CEM, K562). The selectivity index was calculated as SI = (mean IC_50_ of non-tumor cell lines, BJ and MRC-5)/(mean IC_50_ of cancer cell lines without resistant variants, CCRF-CEM, K562, HCT116, HCT116p53–/–, U2OS). Dose-response curves used to calculate IC_50_ are shown in Supporting Information, Figure S22.

At the maximum concentration tested (50 μM), the fluorescent labels alone (**B1**-**B10**) were generally inactive across the cell line panel. Only slight cytotoxic activity was observed for **B4** in CCRF-CEM (32.89 μM), **B5** in CCRF-CEM (27.75 μM), CEM-DNR (36.33 μM), and K562-TAX (35.78 μM), and for **B7** in CEM-DNR (39.86 μM). Primary screening results and dose–response curves used to calculate IC_50_ are shown in Supporting Information, Figure S22.

Connections of **Col** with BODIPY generally reduced its cytotoxicity to different levels depending on the type of linker. Compared to **Col**, the cytotoxic activity of **CB4** and **CB6** changed only slightly. On the other hand, the cytotoxicity of **CB3** and **CB10** was significantly lower. With the exception of **CB8**, all **CBs** showed high selectivity toward tumor cells. **CB2** selectively killed/affected suspension cell lines (CCRF-CEM and K562) and their resistant clones (CEM-DNR and K562-TAX). Interestingly, some linkers probably changed the binding of **Col** to the Pgp-1 and the decline of resistance was observed in both resistant clones. While this effect was found in CEM-DNR cells after treatment with **CB4** and **CB6**, the resistance of K562-TAX cells was inhibited/partially suppressed by a group of conjugates: **CB5**, **CB6** and **CB7**. Structurally, **CBs** containing 1,4-disubstituted 1,2,3-triazoles showed the most promising cytotoxic activity and their IC_50_ values were closest to the IC_50_ of **Col**. Derivatives containing a cationic group (**CB1**, **CB2** and **CB3**) lost cytotoxic activity with the BODIPY linking. The C-10 substituted derivatives generally showed higher IC_50_ values. While those derivatives with a tertiary amine (**CB11**) and a secondary amide (**CB12**) as a linker were selectively cytotoxic to cancer cell lines with IC_50_ values in the range 2.5 μM – 9 μM, the secondary amine as linker (**CB10**) caused only moderate cytotoxicity to cancer cell lines with the lowest IC_50_ value of 7.13 μM for the most sensitive CCRF-CEM cells.

In the work by Arnold et al.^31^ the cytotoxicity of C-7 amide conjugates of **Col** with green-emitting BODIPY FL and red-emitting BODIPY-650/665-X was reported. These substances were tested on HeLa, HepG2, Raji and Vero cells. Compared to **Col**, the IC_50_ values were shifted to an order of magnitude higher concentrations.

Collectively taken, **CB4** was most toxic to CCRF-CEM (IC_50_ = 21 nM), K562 (IC_50_ = 88 nM) and **CB6** to K562 (IC_50_ = 26 nM). It was interesting that these substances demonstrated better resistance indices compared to **Col**, with significant reduction in IC_50_ values compared to **Col**, reaching low submicromolar concentrations in resistant CEM-DNR and K562-TAX sublines.

### Effect on Cell Cycle and Apoptosis

The effect of **CBs** on the cell cycle was investigated in CCRF-CEM cells treated with compounds at 1 × IC_50_ and 5 × IC_50_ concentrations for 24 h (Figure 3A). An increase in G_2_/M phase was observed for most of them. However, in the case of **CB2**, **CB4**, **CB5** and **CB6**, the increase at 5 × IC_50_ exceeded 90% (Supporting Information Table S3). **Col** prevents mitotic spindle formation and chromosome separation. It is clear from Figure 3B that all **CBs** inhibited mitosis and the most pronounced effect was generally observed at 5 × IC_50_ concentration.

**Figure 3:**
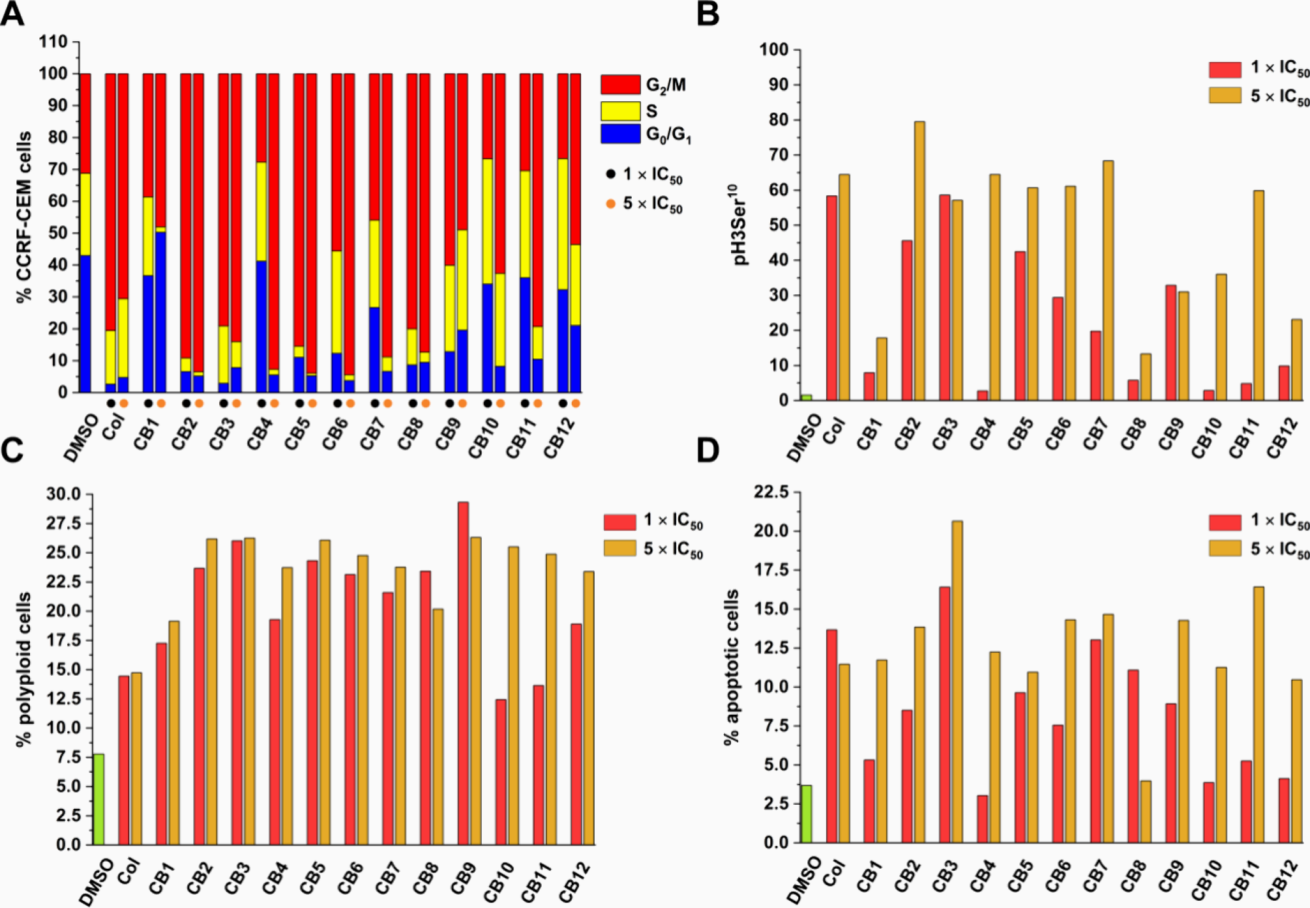
Effect of cytotoxic compounds on cell cycle and apoptosis in CCRF-CEM lymphoblasts (% of positive cells). Flow cytometry analysis was used for the quantification of cell cycle distribution (A), mitosis (B) and polyploidy (C) and apoptosis (sub-G0/G1 DNA content) (D) with a concentration equal to 1 × IC_50_ and 5 × IC_50_. The sum of the percentages for G_0_/G_1_, S, and G_2_/M is equal to 100%. Values represent the means from 3 independent experiments. The table with values is shown in Supporting Information Table S3 and raw data in Figure S22.

**Col** and other mitotic inhibitors, such as paclitaxel, and nocodazole, are known inducers of polyploid cell formation. In normal mitosis, the cell membrane collapses, mitotic spindles form and condensed chromosomes can be identified in metaphase. This phase may be followed by a deviation from normal mitosis, causing restitution of the nuclear membrane and inhibition of nuclear and cell division. However, the number of chromosome sets exceeds two and the cells become polyploid (genome multiplication). Colchicine-induced polyploidization has been previously described in the megakaryocytic lines MEG-01, DAMI, and UT-7, but not in the T-cell line MOLT-T4 or the promyelocytic line HL-60.^47^ Here, we compared colchicine-induced polyploidization versus **CBs** (Figure 3C). As a result, **Col** and all **CBs** induced polyploidization in CCRF-CEM cells at both 1 × IC_50_ and 5 × IC_50_ concentrations.

Induction of apoptosis by **Col** has been reported previously.^48–50^ The mechanism of **Col** induced apoptosis depends on the cell line type. However, the main mechanism is reported to be cytochrome *c* release and caspase activation.^51,52^ Figure 3D shows that compared with the negative control (DMSO), all agents induced apoptosis in CCRF-CEM cells. The most pronounced effect was observed for **CB3** (20%) at a concentration of 1× and 5 × IC_50_.

### *In Vitro* Tubulin Assembly

To assess the impact of the most active **CBs** on αβ-tubulin heterodimer assembly, we selected derivatives with the IC_50_ values below 1 uM or showing more than 30% mitotic cells at 1 × IC_50_. These compounds were then evaluated using an *in vitro* tubulin polymerization assay (Figure 4). The assay is based on light scattering by newly formed microtubules in the presence or absence of the tested compounds. Surprisingly, of the eight derivatives tested, only **CB3** and **CB4** significantly interfered with tubulin assembly. **CB1** and **CB5**-**CB7** displayed only weak inhibition of tubulin assembly (Figure 4A). The control reaction containing only solvent (DMSO) showed a *V*_max_ of 11.6 mOD/min, whereas tubulin assembly was inhibited by the reference compound **Col** to a *V*_max_ of 1.5 mOD/min (Figure 4B). BODIPY fluorophores tagged to **Col** thus differentially impair its binding to tubulin, most likely due to steric hindrance. Nevertheless, any disruption of mitotic spindle formation interferes with successful completion of mitosis, as visible in the cell cycle data (Figure 3 **A-B**). Although the **CB1** was rather mediocre in the assays, it has the greatest similarity to the published substance colchicine-BODIPY FL.^31^ To exclude contamination of **CB4** with synthetic precursor C2 the sample of **CB4** was spiked and evaluated by HRMS analysis. As shown in Figure S20, the **CB4** was not contaminated by synthetic precursor.

**Figure 4:**
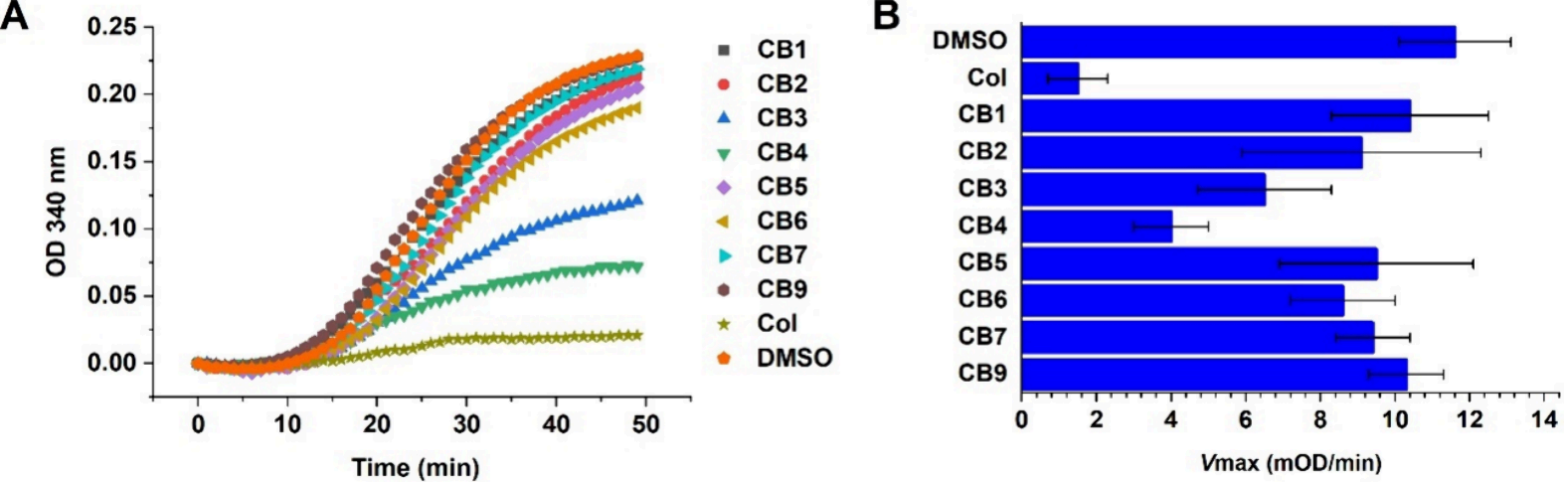
Effect of derivatives on tubulin polymerization curve (A). Polymerization curves are mean values from three independent experiments. *V*_max_ (maximal velocity of polymerization) is calculated from the growth phase of the polymerization curves (B). Data represent the mean ± SD (*n* = 3). The table with values is shown in Supporting Information Table S4.

### Endothelial Cell Tube Formation Angiogenesis Assay

To evaluate the ability of **CBs** to inhibit the formation of endothelial cell tubes, an angiogenesis assay was conducted using the HUVEC cell line (human umbilical vein endothelial cells). Compounds dissolved in the media were added to wells coated with Matrigel, and after a 24-h incubation period, disruption of endothelial cell tube formation was observed for all **CBs** except **CB6** (Figure 5). The results were compared to the control substance colchicine. Equivalent concentrations of DMSO were also used as a control, where normal endothelial cell tube formation was observed.

**Figure 5:**
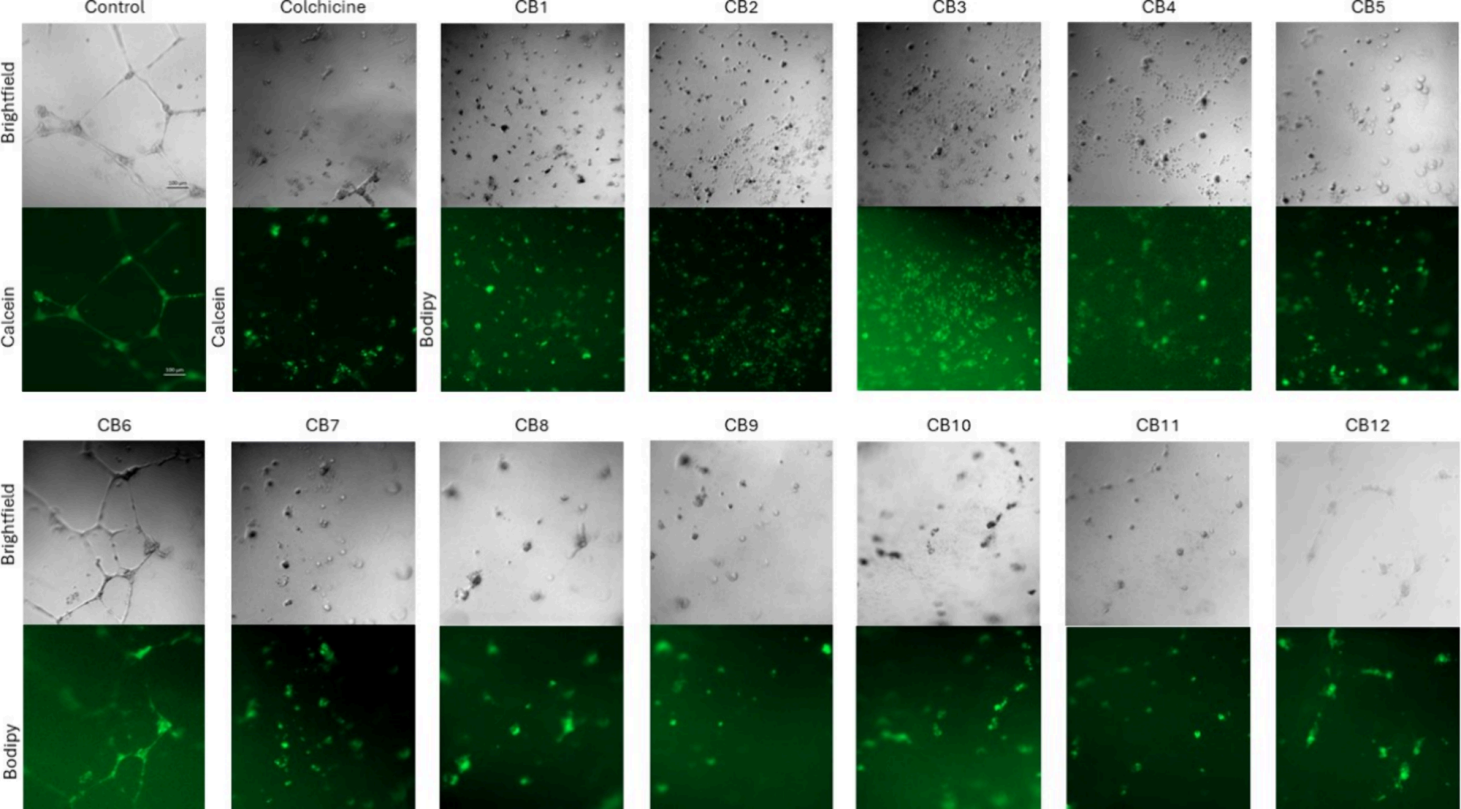
**CBs** inhibit the formation of endothelial cell tubes except **CB6**. The formation of the primary network of HUVEC endothelial cells in the presence of DMSO (control), colchicine, and **CBs** at 1 × IC_50_ concentration. Control and colchicine-treated cells, which, unlike **CBs**, are not labeled with BODIPY, were labeled with calcein (green fluorescence). The scale bar equals 100 μm.

### Fluorescence Microscopy

Changes in the organization of microtubules can be easily observed in well-adherent cell lines, such as human osteosarcoma U2OS cells. The **CBs** (**CB1**, **CB4**, **CB5**, **CB6**, and **CB7**), which had the most potent cytotoxic effect (IC_50_ < 2.00 μM; Table 3) on U2OS cells, were evaluated for their efficacy to disrupt microtubules. Cells were incubated with conjugates at a concentration of 0.1–100 μM for 15 h before fixation and staining with an anti-α-tubulin antibody. **Col** and vehicle (DMSO) served as controls. **Col** effectively depolymerized microtubules at a concentration of 0.1 μM, whereas all conjugates tested were without effect at this concentration. At higher concentrations (1–10 μM), the conjugates affected microtubules differentially. The **CB1** and **CB5** completely disrupted microtubules at 10 μM concentration (Figure 6 **c-d**). On the other hand, microtubule remnants were detectable after treatment of cells with **CB6**, **CB7**, and **CB4** conjugates (Figure 6 **e-g**). In addition, perinuclear bundles were observed in the case of **CB4** and to a lesser extent in the case of **CB6**. In cells, approximately 50–60% of tubulin is polymerized into microtubules, while the remainder exists as a soluble fraction. We suggest that **CBs** with impaired tubulin binding (Figure 4) may still form stable complexes with soluble tubulin over a prolonged period (15 h), leading to inhibition of polymerization and disruption of microtubule organization. On fixed cells, colchicine-BODIPY conjugates provided only very faint diffuse staining, as shown for **CB4** (Figure 6h).

**Figure 6:**
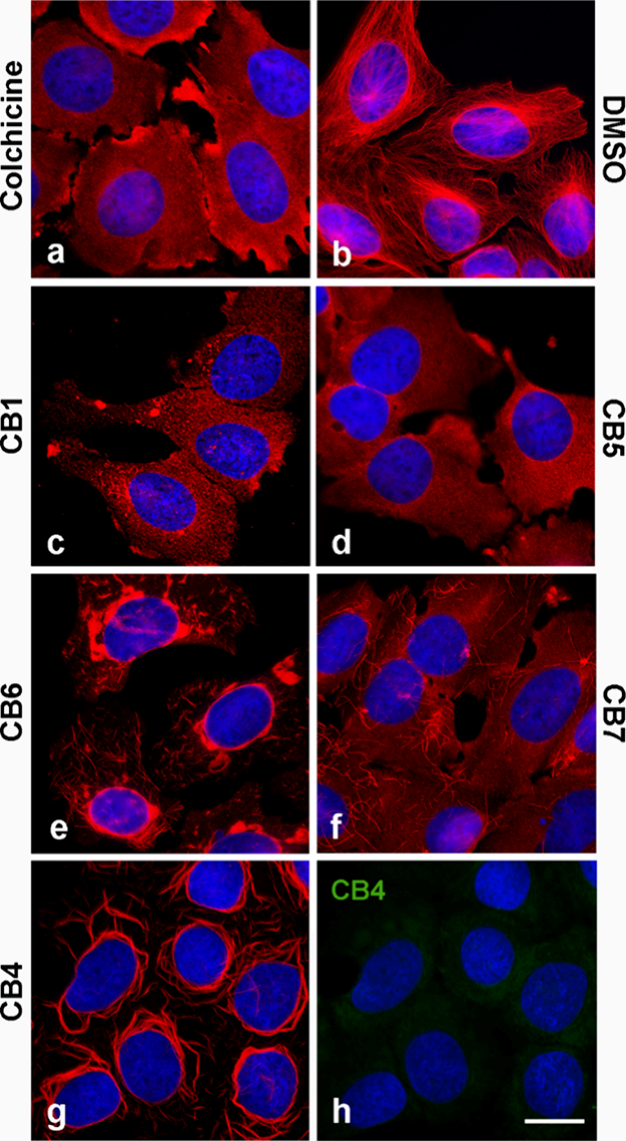
Comparison of microtubule arrangements in cells treated with colchicine-BODIPY conjugates **CB1**, **CB5**, **CB6**, **CB7**, **CB4**, or colchicine. U2OS cells were incubated for 15 h at 37 °C with the compounds studied at a concentration of 10 μM (**a**, **c-h**). The preparations were fixed and stained with an antibody against α-tubulin (red). Cells pretreated with DMSO served as negative controls (**b**). In cells incubated with **CB4**, double-label staining for α-tubulin (**g**; red) and **CB4** (**h**; green) is shown. Cell nuclei were stained with DAPI (blue). Scale bar for **a-h**, 20 μm.

To study the distribution of **CBs** in living cells, we treated cells with the conjugates and immediately examined them by spinning disk confocal microscopy. All **CBs** at a concentration of 1 μM associated with intracellular membranes during 5 min, mainly in the perinuclear region, as shown for **CB4** in Figure 7a, and for **CB1**, **CB5**, **CB6**, and **CB7** in Figure S24. In living cells, the binding of **CB4** to membranes resembling the ER and mitochondria was not due to its BODIPY moiety, **B9**. When images for **CB4** and **B9** were acquired and processed in the same manner, **B9** stained cells only weakly, with the highest staining intensities occurring in discrete spots in the cytoplasm. Therefore, the staining pattern of **B9** was different compared to the conjugate (Figure 7b). The staining of discrete spots throughout the cytoplasm by **B9**, which is not reminiscent of the ER or mitochondria, is clearly seen in Figure S25. In living cells, microtubules decorated with fluorogenic cell-permeable SiR-tubulin, highly specific for microtubules, were not stained with **CB4**. This was expected, as colchicine binding sites are occluded in assembled tubulin polymers.^6^ However, some of the membrane components stained with **CB4** were in contact with microtubules (Figure 8 **a-c**). Because SiR-tubulin is based on the microtubule-binding drug docetaxel, we also used cells expressing mScarlet-tagged α-tubulin to visualize microtubules in live cells and to rule out a possible stabilizing effect of SiR-tubulin. Similar results were also obtained in this experimental setup (not shown). Since the membrane structures stained with **CBs** resembled the ER and mitochondria, both of which are known to be associated with microtubules,^53–55^ we double-stained these organelles with **CB4** in living cells. Decoration of the ER with ER-Tracker (Figure 8 **d-f**) or mitochondria with Mito-Tracker (Figure 8 **g-i**) confirmed the association of **CB4** with these organelles. In control experiments, there was no overlap fluorescence of green **CBs** into the channel for orange Mito-Tracker and vice versa (Supporting Information Figure S26a–d). Similarly, no overlap of **CBs** into the channel for red ER-Tracker and vice versa was observed (not shown).

**Figure 7:**
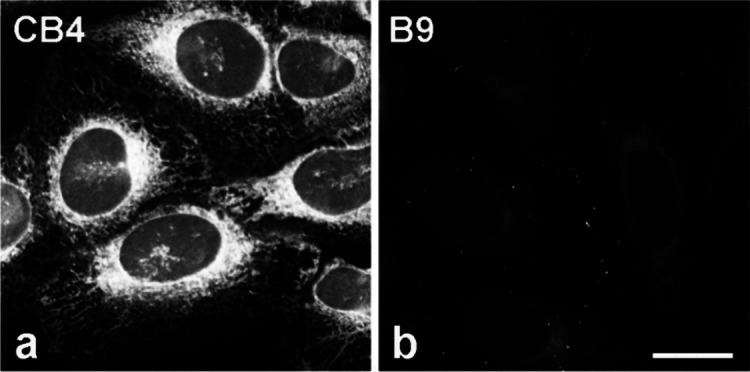
Comparison of **CB4** and its BODIPY moiety B9 distribution in living cells. U2OS cells were incubated for 5 min at 37 °C with **CB4** (**a**) or **B9** (**b**) at a concentration of 1 μM and evaluated by live-cell imaging. The images (a-b) were collected and processed in the same manner. Scale bar for **a-b**, 20 μm.

**Figure 8:**
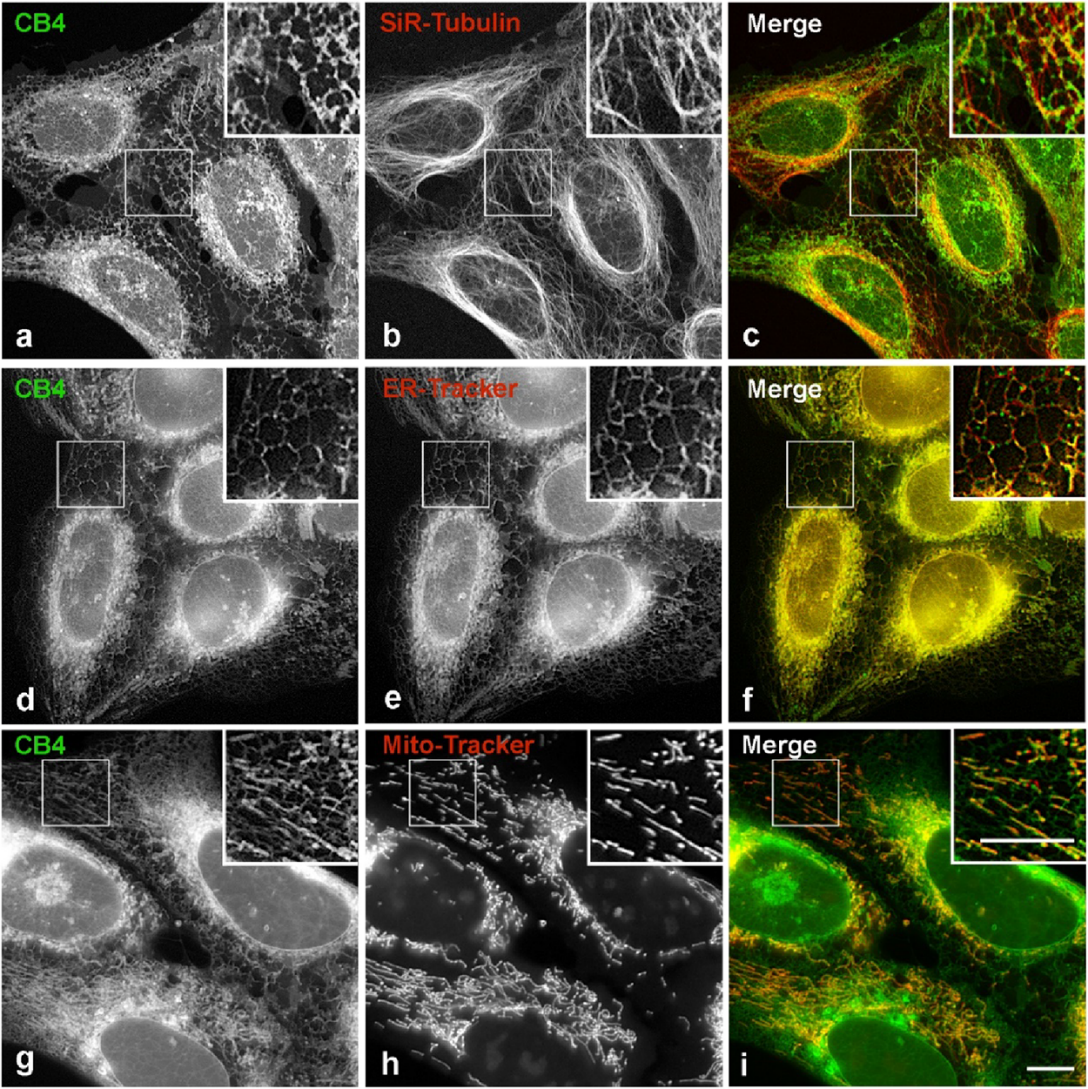
Distribution of **CB4** in living cells. U2OS cells with labeled microtubules, ER or mitochondria were incubated for 5 min at 37 °C with **CB4** at a concentration of 1 μM and evaluated by live-cell imaging. (**a-c**) Double label staining of **CB4** (**a**) and microtubules visualized by SiR-Tubulin (**b**). Superposition of images (**c**; **CB4**, green; SiR-Tubulin, red). (**d-f**) Double label staining of **CB4** (**d**) and ER visualized by ER-Tracker (**e**). Superposition of images (**f**; **CB4**, green; ER-Tracker, red). (**g-i**) Double label staining of **CB4** (**g**) and mitochondria visualized by Mito-Tracker (**h**). Superposition of images (**i**; **CB4**, green; Mito-Tracker, red). Boxed areas are shown at higher magnification. Scale bars for **a-i**, 10 μm.

Staining of perinuclear regions and ER with BODIPY-labeled **Col** in living Vero cells was reported previously by Arnold et al.^31^ In the latter work, only amide BODIPY conjugates with one linker length were tested. From our results, it appears that the BODIPY derivatives containing the triazole-based linker appear to be more potent. In addition, unlike the amide linkage, triazole derivatives are characterized by high chemical stability and are not hydrolyzable in the biological environment.

Previous studies suggested that fluorophore-labeled **Col** is a suitable tool for visualizing microtubules in living cells.^11–13,31^ However, double staining of microtubules and tagged **Col** in live cells was not performed in these studies. Reported staining of microtubules in living cells might actually reflect the decoration of membrane structures along the microtubules.

Taken together, our data suggest that **CBs** in cells primarily target intracellular membranes. Microtubules are preserved during this initial phase (time scale minutes). The later disruption of microtubules (time scale hours) may hypothetically be due to the slow release of **CBs** from membranes and to their irreversible binding to cytosolic pools of soluble tubulin, leading to microtubule destabilization. Such a mechanism was previously predicted based on results from *in vitro* experiments with colchicinoid-anchored lipids.^10^

### *In* *Silico* Modeling

We performed docking of all **CBs** into the colchicine site of tubulin (PDB: 4O2B). Almost all compounds were able to preserve the native position of the **Col** moiety in the binding site in their top scored poses. The exceptions were **CB1**, **CB7** and **CB9**, whose top scored poses differed. However, for them we found colchicine-like conformations in the lower ranked poses. Thus, physically, all ligands can fit sterically into the binding site, but not all were found to prefer this pose according to molecular docking.

To better investigate flexibility and dynamic behavior of the molecules, we performed 50 ns molecular dynamics simulations of top-ranked (colchicine-like) docking poses of **CB2**-**CB6** compounds and **Col** itself. The entire structures of the complexes stabilized after 10 ns of simulations and the RMSD reached a plateau (Figure S27). Ligands in the complexes also stabilized rapidly, but we observed a large change in **CB3** after 40 ns (Figure S28). This can be explained by the movement of the BODIPY moiety, which is connected by a flexible linker and looks outside the protein between alpha and beta chains of tubulin. This was confirmed by analyzing the RMSD values for the **Col** core of the ligands. In all cases, we observed small deviations, less than 1.5 Å, of the **Col** core (Figure S29). The only exception is compound **CB6**, where the **Col** core was shifted at the beginning of the simulation, probably due to the unfavorable pose of the phenyl ring of the linker, which could not fit well into the available space between the two tubulin chains. For each complex, we retrieved protein–ligand interactions from every frame and calculated the average occupancy of each protein–ligand contact. For analysis, we considered contacts that occurred in at least 50% of the frames from the 10 to 50 ns segment (where the RMSD reached a plateau) (Figure 9). Additionally, we provided 2D plots of protein–ligand interaction in Figure S30.

**Figure 9:**
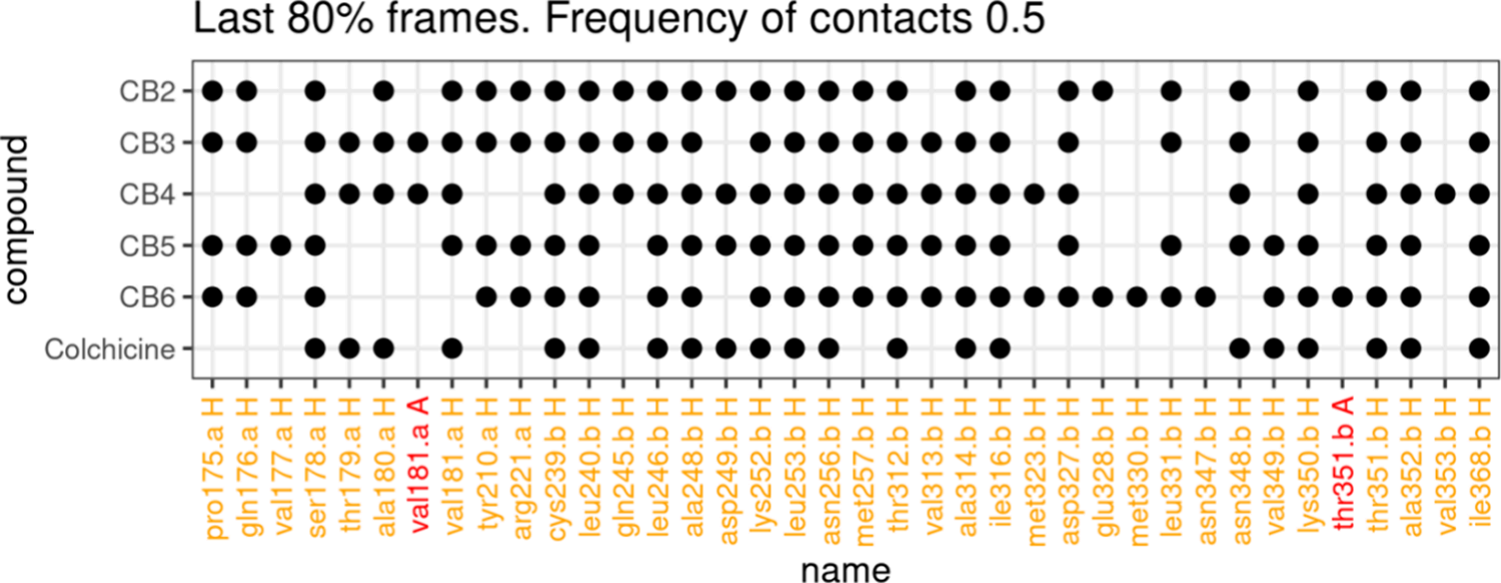
Contacts observed in at least 50% of frames from MD trajectories of complexes of **CB2-CB6** and colchicine with tubulin. We considered only 10–50 ns segment of trajectories where RMSD reached a plateau. Small letters, a or b, after a residue name designate a chain name. Letters A and H designate H-bond acceptor or hydrophobic contact.

We observed almost exclusively hydrophobic contacts, which was not surprising, taking into account the structures of investigated ligands, which have a small number of heteroatoms capable of forming H-bonds. The same pattern was observed for **Col**. The triazole rings in the linker moieties of the **CB4**-**CB6** ligands did not form stable H-bonding with the protein. Overall, **CB4** had the most similar pattern of protein–ligand interactions relative to **Col** compared with the other simulated ligands (Figure 9). We calculated Tanimoto and Tversky scores. The latter show how many contacts demonstrated by **Col** were found for the investigated compounds (Table 4). The higher similarity of the protein–ligand interaction pattern of **CB4** with **Col** can be explained by the smaller size of **CB4** compared to the other compounds (**CB2**, **CB3**, **CB5** and **CB6**), which have a longer linker allowing contact of the BODIPY moiety with the more distant residues of the α-tubulin chain. While the BODIPY residue of **CB4** switched between α and β tubulin chains during the simulation, the BODIPY residues of the other compounds stably interacted with the same Gln176 residue on the α-chain (Figure 10). This was possible because all these ligands have comparable linker lengths.

**Figure 10:**
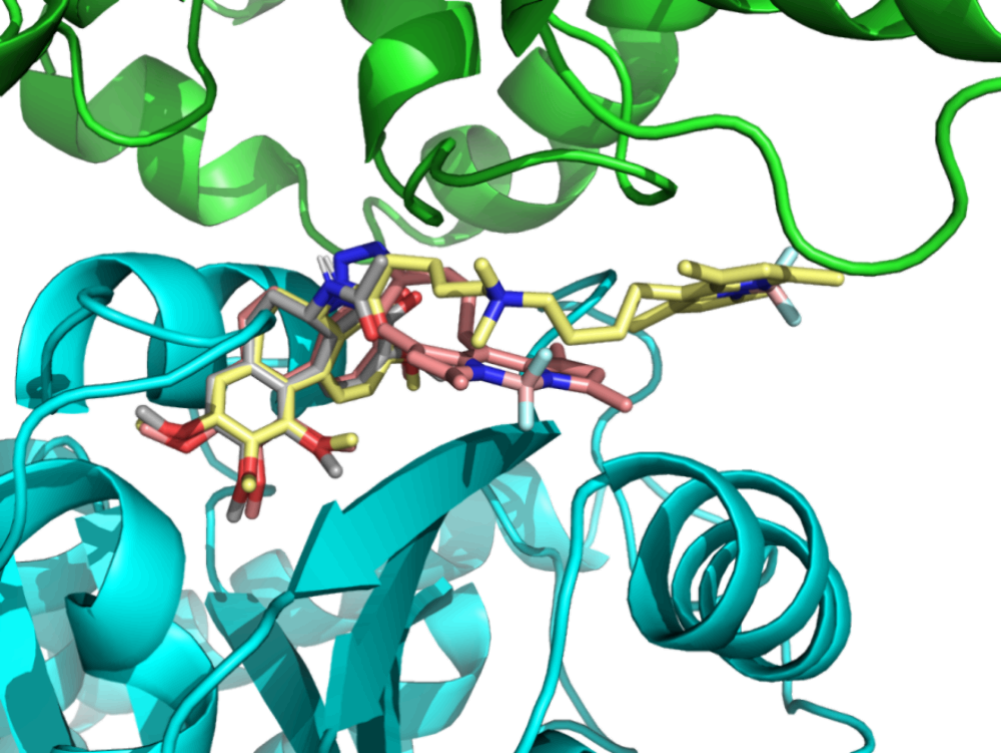
Binding poses of colchicine (gray), **CB3** (yellow), **CB4** (pink) in tubulin (α-tubulin - green, β-tubulin - cyan) obtained from MD simulations. Binding poses of other compounds are provided in Figure S31.

**Table 4:** Similarity of Protein-Ligand Interactions of **CB2-CB6** Ligands Relatively to Colchicine Calculated From 10-50 ns Segments of Corresponding MD Trajectories

compd.	Tanimoto	Tversky (α = 1, β = 0)
CB2	0.63	0.91
CB3	0.61	0.91
CB4	0.71	0.95
CB5	0.63	0.91
CB6	0.47	0.76

### Surface Plasmon Resonance (SPR)

Since **CB4** inhibits tubulin polymerization, its direct interaction with the target was assessed by SPR with colchicine, a well-known tubulin destabilizer, serving as a positive control. Six distinct concentrations of **CB4** and colchicine were injected onto immobilized tubulin on a high-capacity amine sensor chip and real-time sensor readings were recorded. As shown in Figure 11, both **CB4** and colchicine injections resulted in a concentration dependent increase in SPR response. Data analysis revealed that **CB4** has a slightly stronger affinity binding for tubulin compared to colchicine, with dissociation constant (*K*_d_) values of 16.6 ± 1.8 μM and 26.9 ± 1.6 μM, respectively. These *K*_d_ values for colchicine binding to tubulin are consistent with those reported in previous studies using similar methodologies,^56–59^ thereby supporting the reproducibility and reliability of our experimental setup. Furthermore, molecular dynamics (MD) simulations suggest that **CB4**’s higher binding affinity is due to its ability to form more contacts with tubulin. Specifically, **CB4** forms 27 contacts, including interactions with amino acids Val181, Gln245, Met257, Val313, Met323, and Val353 compared to the 21 contacts formed by colchicine (Figure 9). These additional interactions likely contribute to **CB4**’s stronger dissociation constant as measured by SPR.

**Figure 11:**
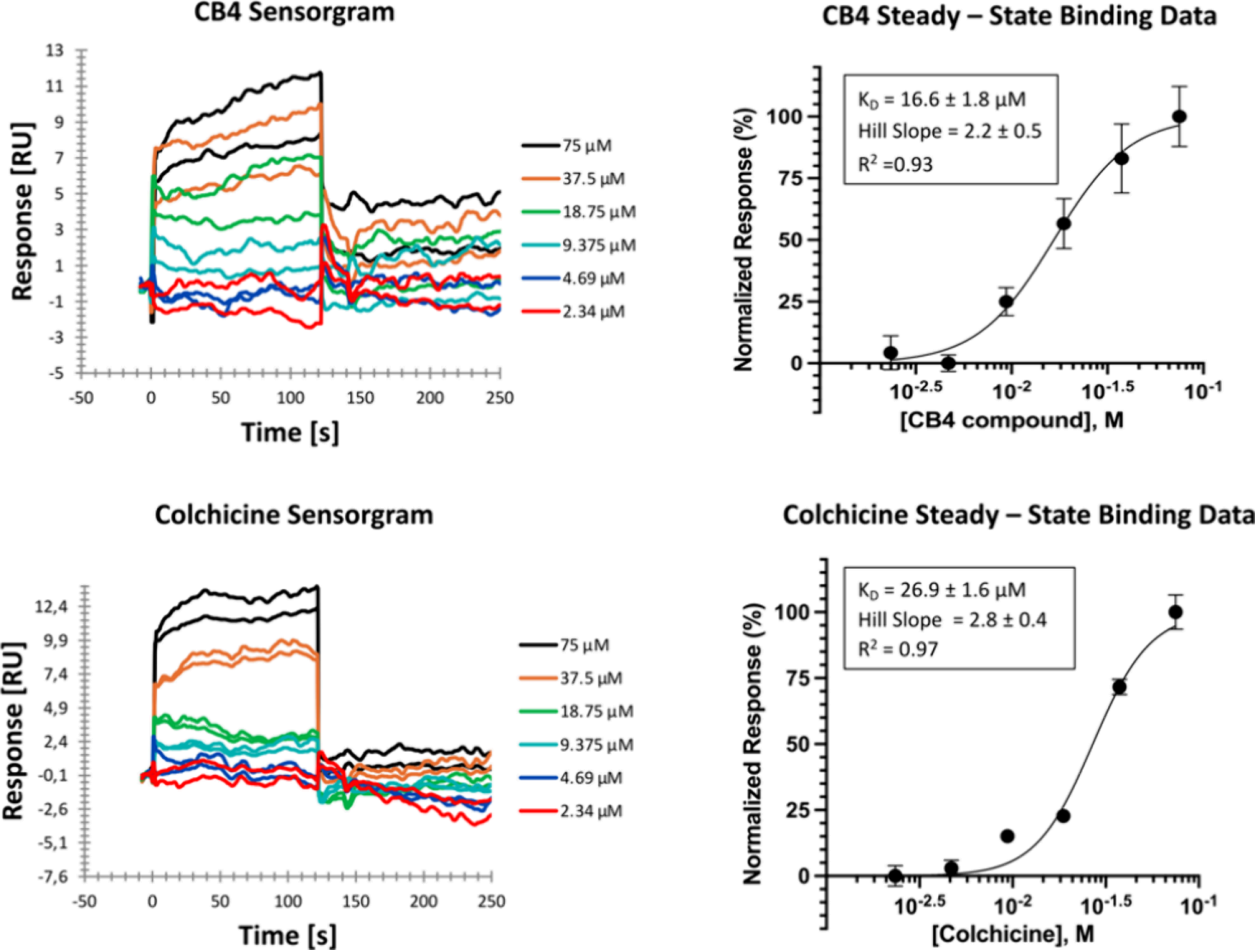
Sensorgrams and four-parameters regression curves for colchicine (2.34, 4.69, 9.375, 18.75, 37.5, and 75 μM, upper graphs) and **CB4** compound (2.34, 4.69, 9.375, 18.75, 37.5, and 75 μM, lower graphs) binding to tubulin as determined by surface plasmon resonance spectroscopy. Data are presented as the mean value of duplicate measurements (*n* = 2), with error bars representing the standard error of the mean (SEM), while R^2^ represents the sum of the squares of the distances of the points from the best-fit curve as determined by nonlinear regression.

## Conclusions

In summary, 12 **CBs** were designed, synthesized and thoroughly characterized. Our SAR analysis revealed that conjugation at C7 is preferred, while BODIPY installation at C10 diminished potency. The structural study highlighted a key moiety essential for tubulin binding, whereas other regions can be substituted without major losses in activity. Notably, a triazole linkage proved superior to an amide bond, and shorter, nonionic linkers enhanced performance (see Figure 12 for a graphical overview). We determined the **CB4** conjugate as the most potent one. Compared to the others, **CB4** had a very strong cytotoxic effect in lymphoblastic leukemia CCRF-CEM cells as well as in paclitaxel-resistant K562-TAX cells. It arrested almost 93% of cells in the G_2_/M phase of the cell cycle and showed the greatest similarity to **Col** in respect of the inhibition of tubulin polymerization *in vitro*. Prolonged treatment of cells with **CB4** (10 μM for 15 h) resulted in disruption of microtubule organization and generation of perinuclear microtubule bundles. Interestingly, live cell imaging revealed that **CB4**, similarly as the other tested conjugates **CB1**, **CB5**, **CB6**, and **CB7**, rapidly associated with intracellular membranes. Furthermore, **CB4** exhibited a higher binding affinity to tubulin compared to **Col**, as elucidated by biophysical analysis using surface plasmon resonance. These findings not only underscore the value of the SAR insights but also establish the newly prepared BODIPY-tagged colchicines as useful probes for studying the fate of **Col** in living cells and thereby expand the scarce knowledge of the molecular mechanism of its action, e.g., during inflammation.

**Figure 12:**
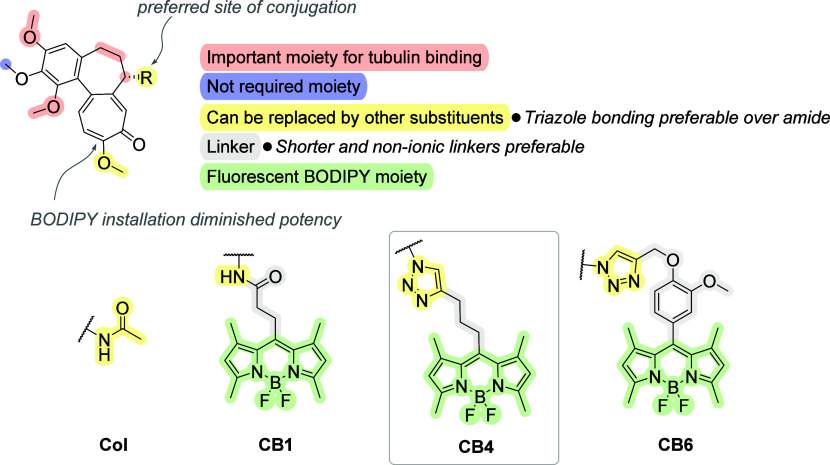
Structure–activity relationship of **Col** and **CBs**.

## Experimental Section

### Chemistry

#### Methods and Materials

For thin-layer chromatography (TLC), aluminum silica gel sheets for detection in UV light (TLC Silica gel 60 F_254,_ Merck) were used. For TLC visualization diluted solution of sulfuric acid in methanol was used and plates were heated. Silica gel (30–60 μm, SiliTech, MP Biomedicals) for column chromatography was used. NMR spectra were recorded on Agilent-MR DDR2 (Varian, Palo Alto, CA, USA). Chemical shifts are given in δ (ppm). HRMS were measured by LTQ ORBITRAP VELOS with HESI^+^/HESI^–^ ionization (Thermo Scientific, Waltham, USA). HPLC analyses were performed with C18 column (100 mm) and UV detection. There was used gradient elution with a flow rate of 0.3 mL/min and the following systems: system A – 50% MeOH, system B absolute MeOH. The method was optimized for 20 min with a gradient system documented in Supporting Information Table S1. LogP was calculated by software ACD/Percepta 14.54.0 (Build 3666).

Chemicals were purchased from TCI Europe (Zwijndrecht, Belgium): Colchicine **C1** (>97%, contains 5% AcOEt at maximum); 2,4-dimethylpyrrole (>97%); boron trifluoride - ethyl ether complex – BF_3_·Et_2_O (>98%); *N,N,N-*triethylamine – TEA (>99%); oxalyl chloride (>98%); *N*,*N*-diisopropylethylamine – EDIPA (>99%); 4*-*dimethylaminopyridine – 4-DMAP (>99%); ethyl-dimethylaminopropyl carbodiimide hydrochloride – EDCI (>98%); *N*-hydroxybenzotriazole – HOBt (>98%); monoethyl dodecanedioate (>98%); monomethyl succinate (>98%); vanillin (>98%); propargyl bromide (>97%, stabilized with MgO); 5-hexynoic acid (>96%); 10-undecynoic acid (>98%); methylamine (ca. 2 mol/L in THF); dimethylamine (ca. 2 mol/L in THF); 5-bromovaleric acid (>97%); 1-[bis­(dimethylamino)­methylene]-1*H*-benzotriazolium 3-oxide hexafluorophosphate – HBTU (>98%); tris­[(1-benzyl-1*H*-1,2,3-triazol-4-yl)­methyl]­amine – TBTA (>97%). The solvents for column chromatography and reactions were purchased from PENTA (Praha, Czech Republic) and were used without further distillation.

Syntheses of BODIPY dyes **B1,** ^60^ **B3,** ^33^ **B4,** ^33^ **B7** ^61^ and **B9** ^35^ were described previously. **B8** was synthesized by different procedure than published by Panovic et al.^34^ Colchicine derivatives *N*-deacetyl colchicine **C1,** ^62^ 7-azido-7-deacetamidocolchicine **C2,** ^37^ and 10-*N-*methylaminocolchiceine **C4** ^44^ used in this research were prepared and analytically described previously.

#### Synthesis of BODIPY dyes

##### BODIPY **B2**

To a solution of monoethyl dodecanoic acid (2 g, 7.7 mmol) in DCM (100 mL) oxalyl chloride (3.3 mL, 39 mmol) and 3 drops of DMF were added. The mixture was stirred ON at RT. Toluene was added and the solvents were evaporated under reduced pressure. The mixture was coevaporated with toluene (2 × 50 mL) and dissolved in dry DCM (150 mL). 2,4-Dimethylpyrrole (1.6 g, 17 mmol) was added and the mixture was stirred ON at RT. TEA (7.7 mL) was added and after 30 min BF_3_·Et_2_O (12 mL). The mixture was stirred for 5 h at RT on air. MeOH (100 mL) was added and the solvents were evaporated under reduced pressure. The mixture was filtered through a plug of silica (hexane-AcOEt 9:1, *v/v*). The fractions containing product were collected and the solvents were evaporated under reduced pressure. The matter thus obtained was chromatographed (hexane-AcOEt 9:1, *v/v*) to obtain ethylester **B2a** (1.7 g, 3.7 mmol) as dark orange-brown gel in 47% yield. **R**_**F**_ = 0.8 in hexane-AcOEt 5:1 (*v/v*). ^**1**^ **H NMR** (400 MHz, CDCl_3_) δ ppm: 1.25 (t, *J* = 7.0 Hz, 3 H, OCH_2_C*H*_3_), 1.29 (br s, 10 H, linker 5 × C*H*_2_), 1.47 (dt, *J* = 14.1, 7.0 Hz, 2 H, linker C*H*_2_), 1.55–1.68 (m, 4 H, linker 2 × C*H*_2_), 2.29 (t, *J* = 7.4 Hz, 2 H, linker C*H*_2_), 2.40 (s, 6 H, BODIPY 2 × C*H*_3_), 2.51 (s, 6 H, BODIPY 2 × C*H*_3_), 2.84–2.97 (m, 2 H, linker C*H*_2_), 4.12 (q, *J* = 7.0 Hz, 2 H, CH_3_C*H*_2_OCO), 6.04 (s, 2 H, BODIPY 2 × C*H*); Figure S1A. ^**13**^ **C NMR** (101 MHz, CDCl_3_) δ ppm: 13.34, 13.48 (t, *J*_CF_ = 2.3 Hz), 15.40, 24.01, 27.52, 28.17, 28.30, 28.42, 28.45, 28.55, 29.44, 30.93, 33.42, 59.22, 120.59, 130.48, 139.38, 145.77, 152.70, 172.93; Figure S1B. **HRMS-ESI**: for C_26_H_39_BF_2_N_2_O_2_ calcd 460.30727 Da, found *m*/*z* 483.29709 [M + Na]^+^ and 499.26992 [M+K]^+^; Figure S1C.

The ethylester **B2a** (1.7 g, 3.7 mmol) was dissolved in THF (50 mL) and aqueous LiOH (30 mL, 4M) was added during 20 min at RT. After the addition was completed, the mixture was heated up to 80 °C for 6 h. Then, the heating was removed and the mixture was allowed to cool to RT. The mixture was diluted with water (200 mL) and pH was adjusted with concentrated HCl. The product was extracted with AcOEt (5 × 40 mL). The combined organic layer was washed with water (1 × 50 mL), saturated brine (1 × 50 mL) and dried over MgSO_4_. The mixture was filtered and solvents were evaporated on rotary evaporator to the dryness. The residue was chromatographed (hexane-AcOEt 3:1 → 1:1, *v/v*). The fractions containing product were collected and solvents evaporated under reduced pressure. The matter thus obtained was dissolved in DCM (15 mL) and the product was precipitated by the addition of hexanes. BODIPY **B2** (672 mg, 1.55 mmol) was isolated as orange solids in 42% yield (by chromatography 365 mg (0.8 mmol) of ethylester was recovered). Overall yield was 20%. **R**_**F**_ = 0.3 in hexane-AcOEt 2:1 (*v/v*). ^**1**^ **H NMR** (400 MHz, CDCl_3_) δ ppm: 1.30 (br s, 10 H, linker 5 × C*H*_2_), 1.49 (dt, *J* = 14.1, 7.0 Hz, 2 H, linker C*H*_2_), 1.57–1.70 (m, 4 H, linker 2 × C*H*_2_), 2.36 (t, *J* = 7.6 Hz, 2 H, linker C*H*_2_), 2.42 (s, 6 H, BODIPY 2 × C*H*_3_), 2.52 (s, 6 H, BODIPY 2 × C*H*_3_), 2.86–2.99 (m, 2 H, linker C*H*_2_), 6.05 (s, 2 H, BODIPY 2 × C*H*); Figure S2A. ^**13**^ **C NMR** (101 MHz, CDCl_3_) δ ppm: 14.43 (t, *J*_CF_ = 2.3 Hz), 16.37, 24.63, 28.47, 28.99, 29.18, 29.31, 29.36, 29.46, 30.36, 31.89, 33.87, 121.54, 131.40, 140.27, 146.65, 153.69, 179.27; Figure S2B. **HRMS-ESI**: for C_24_H_35_BF_2_N_2_O_2_ calcd 432.27597 Da, found *m*/*z* 455.26573 [M + Na]^+^ and 471.23946 [M+K]^+^; Figure S2C.

##### BODIPY **B5**

Dye **B3** (350 mg, 0.91 mmol) was dissolved in MeNH_2_ solution (ca. 2 mol/L in THF, 8 mL) in screw-cap vial. The flask was closed with pressure cap and the solution was heated in oil bath up to 100 °C for 3 h and then stirred at 50 °C for 16 h. The heating was removed and the solvents were evaporated under reduced pressure. The residue was passed through a short column of silica (CHCl_3_-MeOH 20:1 → 9:1, *v/v*). BODIPY **B5** (250 mg, 0.72 mmol) was obtained as orange solids in 82% yield. **R**_**F**_ = 0.3 in DCM-MeOH 10:1 (*v/v*). ^**1**^ **H NMR** (400 MHz, CDCl_3_) δ: 1.65–1.75 (m, 2 H, linker C*H*_2_), 1.93 (dt, *J* = 15.4, 7.8 Hz, 2 H, linker C*H*_2_), 2.41 (s, 6 H, BODIPY 2 × C*H*_3_), 2.51 (s, 6 H, BODIPY 2 × C*H*_3_), 2.56 (s, 3 H, C*H*_3_NH−), 2.82–2.89 (m, 2 H, linker C*H*_2_), 2.95–3.03 (m, 2 H, linker C*H*_2_), 4.21 (br s, 1 H, N*H*), 6.05 (s, 2 H, BODIPY 2 × C*H*); Figure S3A. ^**13**^ **C NMR** (101 MHz, CDCl_3_) δ: 14.42, 16.53, 27.69, 27.79, 29.12, 34.13, 49.90, 121.81, 131.33, 140.25, 145.08, 154.16; Figure S3B. **HRMS-ESI**: for C_18_H_26_BF_2_N_3_ calcd 333.21878 Da, found *m*/*z* 334.22650 [M + H]^+^; Figure S3C.

##### BODIPY **B6**

To a solution of **B3** (500 mg, 1.3 mmol) in AcCN (3 mL) was added Me_2_NH solution (ca. 2 mol/L in THF, 10 mL) and the mixture was stirred for 18 h at RT. The solvent was evaporated under reduced pressure. The residue was taken up with DCM (50 mL) and washed with water (1 × 50 mL) and brine (1 × 50 mL). The organic layer was dried over MgSO_4_, filtered and the solvents were evaporated under reduced pressure. The matter thus obtained was dissolved in DCM (10 mL) and precipitated by the addition of hexane. The solids were fritted and dried *in vacuo.* BODIPY **B6** (326 mg, 9.4 mmol) was obtained as orange solids in 72% yield. **R**_**F**_ = 0.5 in DCM-MeOH 10:1 (*v/v*). ^**1**^ **H NMR** (400 MHz, CDCl_3_) δ: 1.56–1.73 (m, 2 H, linker C*H*_2_), 1.99–2.11 (m, 2 H, linker C*H*_2_), 2.38 (s, 6 H, BODIPY 2 × C*H*_3_), 2.49 (s, 6 H, BODIPY 2 × C*H*_3_), 2.77 (s, 6 H, (C*H*_3_)_2_NH−), 2.93–3.04 (m, 4 H, 2 × C*H*_2_), 6.05 (s, 2 H, BODIPY 2 × C*H*), 12.16 (br s, 1 H, Me_2_N*H* ^+^-); Figure S4A. ^**13**^ **C NMR** (101 MHz, CDCl_3_) δ: 14.46, 16.61, 24.64, 27.38, 28.62, 42.93, 57.53, 121.97, 131.30, 140.33, 144.49, 154.33; Figure S4B. **HRMS-ESI**: for C_19_H_28_BF_2_N_3_ calcd 347.23443 Da, found *m*/*z* 348.24208 [M + H]^+^; Figure S4C.

##### BODIPY **B8**

To a solution of **B7** (1.7 g, 4.2 mmol) in anhydrous DMF (40 mL), solid K_2_CO_3_ (5.4 g, 6.3 mmol) was added and the heterogeneous mixture was stirred for 30 min at RT. Propargyl bromide (2 mL, 26 mmol) diluted with toluene (8 mL) was added dropwise via syringe. A mixture was further stirred at 50 °C for 19 h and then cooled to RT. The mixture was poured into AcOEt (50 mL), washed with saturated brine (1 × 50 mL) and water (1 × 50 mL). The separated organic layer was dried over MgSO_4_ and filtered. The solvents were evaporated under reduced pressure and the residue was chromatographed (hexane-DCM 3:2 → 1:1, *v/v*). The matter thus obtained was dissolved in AcOEt and the product was precipitated by the addition of hexane and collected by filtration on a paper. BODIPY **B8** (401 mg, 0.98 mmol) was obtained as brick-colored crystals in 23% yield. **R**_**F**_ = 0.6 in hexane-AcOEt 5:1 (*v/v*). ^**1**^ **H NMR** (400 MHz, CDCl_3_) δ ppm: 1.48 (s, 6 H, BODIPY 2 × C*H*_3_), 2.54 (t, *J* = 2.7 Hz, 1 H, propargyl C*H*), 2.55 (s, 6 H, BODIPY 2 × C*H*_3_), 3.85 (s, 3 H, -OC*H*_3_), 4.83 (d, *J* = 2.4 Hz, 2 H, propargyl C*H*_2_), 5.99 (s, 2 H, BODIPY 2 × C*H*), 6.80–6.86 (m, 2 H, Ar*H*), 7.13 (d, *J* = 8.2 Hz, 1 H, Ar*H*); Figure S5A. ^**13**^ **C NMR** (101 MHz, CDCl_3_) δ ppm: 14.41, 14.58 (t, *J*_CF_ = 2.3 Hz), 56.12, 56.91, 76.17, 77.99, 111.52, 114.77, 120.22, 121.14, 121.17, 128.55, 131.58, 141.27, 143.12, 147.22, 150.49, 155.46; Figure S5B. **HRMS-ESI**: for C_23_H_23_BF_2_N_2_O_2_ calcd 408.18206 Da, found *m*/*z* 431.17161 [M + H]^+^; Figure S5C.

##### BODIPY **B10**

To undecynoic acid (2.2 g, 12 mmol) in DCM (20 mL) (COCl)_2_ (2 mL, 24 mmol) and 3 drops of DMF were added. The mixture was stirred 19 h at RT. The solvents were evaporated under reduced pressure and the residue was coevaporated with toluene (3 × 25 mL). The brownish residue was dissolved in DCM (150 mL) and 2,4-dimethylpyrrole (2.5 g, 26.4 mmol) was added. The reaction proceeded 21 h at RT on air. DCM (50 mL) and TEA (26 mL) were added and the mixture was stirred for 1 h at RT. Then, BF_3_·Et_2_O (27 mL) was added during 30 min and the reaction proceeded for 5 h. To the vigorously stirred mixture MeOH (100 mL) was added. After 30 min the solvents were concentrated on rotary evaporator. The residue was then chromatographed (hexane-DCM 3:2, *v/v*). The crude product was dissolved in AcOEt (20 mL) and precipitated by the addition of hexanes. BODIPY **B10** (1.01 g, 2.64 mmol) was obtained as orange solids in 22% yield. **R**_**F**_ = 0.8 in hexane-AcOEt 5:1 (*v/v*). ^**1**^ **H NMR** (400 MHz, CDCl_3_) δ ppm: 1.27–1.45 (m, 6 H, linker 3× C*H*_2_), 1.45–1.55 (m, 4 H, linker 2× C*H*_2_), 1.55–1.67 (m, 2 H, linker C*H*_2_), 1.94 (t, *J* = 2.7 Hz, 1 H, −CC*H*), 2.18 (td, *J* = 6.9, 2.5 Hz, 2 H, linker C*H*_2_), 2.40 (s, 6 H, BODIPY 2 × C*H*_3_), 2.51 (s, 6 H, BODIPY 2 × C*H*_3_), 2.89–2.96 (m, 2 H, linker C*H*_2_), 6.04 (s, 2 H, BODIPY 2 × C*H*); Figure S6A. ^**13**^ **C NMR** (101 MHz, CDCl_3_) δ ppm: 14.43 (t, *J*_CF_ = 2.7 Hz), 16.37, 18.35, 28.39, 28.45, 28.61, 29.00, 29.25, 30.30, 31.86, 68.16, 84.62, 121.53, 131.41, 140.26, 146.61, 153.69; Figure S6B. **HRMS-ESI**: for C_23_H_31_BF_2_N_2_ calcd 384.25484 Da, found *m*/*z* 407.24490 [M + Na]^+^; Figure S6C.

### Synthesis of CBs

#### General Procedure for the Synthesis of C-7 Amides

To a solution of **C1** (1 equiv) and **B1**, **B2** or **B3** (1.2 equiv) in dry DCM (5–15 mL), EDCI (1.5 equiv), HOBt (1 equiv) and 4-DMAP (1.5 equiv) were added. The mixture was stirred under argon atmosphere ON at RT. The solvents were evaporated under reduced pressure and the residue was chromatographed. The products thus obtained were dissolved in the DCM-MeOH and precipitated by the addition Et_2_O-hexane (4:6, *v/v*) mixture. The solids were filtered on a paper and dried *in vacuo*. After analytical characterizations the samples were lyophilized from 1,4-dioxane and stored in plastic vials in the fridge.

##### Conjugate **CB1**

In reaction: **C1** (100 mg, 0.28 mmol) and **B1** (109 mg, 0.34 mmol). Chromatography (2×): AcOEt-MeOH 20:1 → 10:1 (*v/v*). **CB1** (146 mg, 0.22 mmol) was obtained as orange colored lyophilizate in 79% yield. **R**_**F**_ = 0.3 in AcOEt. ^**1**^ **H NMR** (400 MHz, CDCl_3_) δ ppm: 1.87 (dt, *J* = 11.7, 5.9 Hz, 1 H, Col C*H*), 2.17–2.25 (m, 1 H, Col C*H*), 2.30 (br s, 6 H, BODIPY 2 × C*H*_3_), 2.35–2.41 (m, 1 H, *overlap with signal of BODIPY CH*_*3*_), 2.46 (br s, 6 H, BODIPY 2 × C*H*_3_), 2.50–2.64 (m, 2 H, *signal overlap*), 3.10 (td, *J* = 12.7, 5.1 Hz, 1 H, linker C*H*), 3.20 (td, *J* = 12.7 Hz, 12.7, 5.1 Hz, 1 H, linker C*H*), 3.72 (s, 3 H, Col OC*H*_3_), 3.77 (s, 3 H, Col OC*H*_3_), 3.90 (s, 3 H, Col OC*H*_3_), 3.95 (s, 3 H, Col OC*H*_3_), 4.68 (dt, *J* = 11.7, 6.8 Hz, 1 H, Col C*H*-NH−), 5.93 (br s, 2 H, BODIPY 2 × C*H*), 6.54 (s, 1 H, Col Ar*H*), 6.75 (d, *J* = 11.0 Hz, 1 H, tropolone C*H*), 7.31 (d, *J* = 11.0 Hz, 1 H, tropolone C*H*), 7.47 (br d, *J* = 7.4 Hz, 1 H, N*H*), 7.55 (s, 1 H, tropolone C*H*); Figure S7A. ^**13**^ **C NMR** (101 MHz, CDCl_3_) δ ppm: 14.37, 16.45, 23.44, 30.04, 36.10, 37.20, 52.04, 56.04, 56.09, 61.34, 61.60, 107.41, 112.41, 121.82, 125.58, 130.94, 131.13, 134.30, 135.36, 136.44, 140.68, 141.62, 144.80, 151.14, 151.74, 153.50, 163.96, 170.41, 179.42; Figure S7B. **HRMS-ESI**: for C_36_H_40_BF_2_N_3_O_6_ calcd 659.29782 Da, found *m*/*z* 682.28760 [M + Na]^+^; Figure S7C. **HPLC**: R_T_ = 5.646 min; Figure S7D.

##### Conjugate **CB2**

In reaction: **C1** (100 mg, 0.28 mmol) and **B2** (145 mg, 0.34 mmol). Chromatography (2×): CHCl_3_-MeOH 200:1 (*v/v*). **CB2** (181 mg, 0.23 mmol) was obtained as brick- colored solids in 84% yield. **R**_**F**_ = 0.4 in AcOEt. ^**1**^ **H NMR** (400 MHz, CDCl_3_) δ ppm: 1.15–1.32 (m, 10 H, linker 5 × C*H*_2_), 1.42 (dt, *J* = 14.6, 7.0 Hz, 2 H, linker C*H*_2_), 1.47–1.62 (m, 4 H, linker 2 × C*H*_2_), 1.82 (td, *J* = 11.6, 5.7 Hz, 1 H, Col C*H*), 2.16–2.22 (m, 2 H, *overlap*), 2.23–2.33 (m, 2 H, *overlap*), 2.36 (s, 6 H, BODIPY 2 × C*H*_3_), 2.47 (s, 6 H, BODIPY 2 × C*H*_3_), 2.80–2.91 (m, 2 H, −C*H*_2_-BODIPY), 3.64 (s, 3 H, Col OC*H*_3_), 3.87 (s, 3 H, Col OC*H*_3_), 3.92 (s, 3 H, Col OC*H*_3_), 3.96 (s, 3 H, Col OC*H*_3_), 4.62 (dt, *J* = 12.0, 6.3 Hz, 1 H, −C*H*-NH−), 6.01 (s, 2 H, BODIPY 2 × C*H*), 6.50 (s, 1 H, Col Ar*H*), 6.83 (d, *J* = 11.0 Hz, 1 H, tropolone C*H*), 7.30 (d, *J* = 11.0 Hz, 1 H, tropolone C*H*), 7.49 (s, 1 H, tropolone C*H*), 7.53 (br d, *J* = 6.7 Hz, 1 H, N*H*); Figure S8A. ^**13**^ **C NMR** (101 MHz, CDCl_3_) δ ppm: 14.40 (t, *J*_CF_ = 2.7 Hz), 16.33, 25.31, 28.43, 29.28, 29.30, 29.34, 29.50, 29.91, 30.35, 31.86, 36.08, 36.61, 52.22, 56.09, 56.36, 61.37, 61.59, 107.30, 112.59, 121.51, 125.63, 130.52, 131.38, 134.23, 135.33, 136.64, 140.32, 141.61, 146.71, 151.18, 152.17, 153.45, 153.57, 163.97, 172.94, 179.43; Figure S8B. **HRMS-ESI**: for C_44_H_56_BF_2_N_3_O_6_ calcd 771.42302 Da, found *m*/*z* 794.41274 [M + Na]^+^; Figure S8C. **HPLC**: R_T_ = 6.810 min; Figure S8D.

##### Intermediate **C3**

In reaction: **C1** (300 mg, 0.84 mmol) and 5-bromovaleric acid (228 mg, 1.26 mmol). Chromatography (2×): AcOEt-MeOH 20:1 → 10:1 (*v/v*). **C3** (288 mg, 0.55 mmol) was obtained as yellowish lyophilizate in 65% yield. **R**_**F**_ = 0.4 in AcOEt-MeOH 15:1 (*v/v*). ^**1**^ **H NMR** (400 MHz, CDCl_3_) δ ppm: 1.62–1.72 (m, 2 H, linker C*H*_2_), 1.74–1.82 (m, 2 H, linker C*H*_2_), 1.86–1.96 (m, 1 H, Col C*H*), 2.11–2.40 (m, 4 H, linker C*H*_2_, Col 2 × C*H*), 2.46–2.53 (m, 1 H, Col C*H*), 3.30 (t, *J* = 6.7 Hz, 2 H, −C*H*_2_-Br), 3.64 (s, 3 H, Col OC*H*_3_), 3.88 (s, 3 H, Col OC*H*_3_), 3.92 (s, 3 H, Col OC*H*_3_), 3.99 (s, 3 H, Col OC*H*_3_), 4.64 (dt, *J* = 11.9, 6.2 Hz, 1 H, −C*H*-NH−), 6.52 (s, 1 H, Col Ar*H*), 6.87 (d, *J* = 10.6 Hz, 1 H, tropolone C*H*), 7.34 (d, *J* = 10.6 Hz, 1 H, tropolone C*H*), 7.54 (s, 1 H, tropolone C*H*), 7.92 (d, *J* = 6.7 Hz, 1 H, N*H*); Figure S9A. ^**13**^ **C NMR** (101 MHz, CDCl_3_) δ ppm: 23.90, 29.92, 32.18, 33.18, 34.80, 36.66, 52.37, 56.09, 56.46, 61.38, 61.62, 107.32, 112.88, 125.51, 130.52, 134.24, 135.59, 136.87, 141.59, 151.15, 152.54, 153.51, 164.00, 172.20, 179.43; Figure S9B. **HRMS-ESI**: for C_25_H_30_BrNO_6_ calcd 519.12565 Da, found *m*/*z* 520.13252 [M + H]^+^; Figure S9C.

##### Conjugate **CB3**

Bromoderivative **C3** (150 mg, 0.29 mmol) and **B6** (101 mg, 0.29 mmol) were dissolved in AcCN (3.5 mL). The vial was closed with pressure cap and heated (oil bath) up to 70 °C for 3 h and then at 55 °C ON. The heating was removed and the mixture was allowed to cool to RT. The mixture was poured into the mixture of Et_2_O-hexane (100 mL, 8:2 *v/v*). Solids formed were collected by filtration on a paper and dried *in vacuo*. The material thus obtained was chromatographed (DCM-MeOH-AcONH_4_, 10:1:0.1, *v/v/v*). The fractions containing product were collected and the solvents were evaporated under reduced pressure. The crude was dissolved in the DCM-MeOH (10 mL, 4:6 *v/v*) and precipitated into the cold mixture of Et_2_O-hexanes (50 mL, 1:1 *v*/*v*). The solids formed were immediately filtered on a paper, washed with Et_2_O (2 × 5 mL), dried and lyophilized from the mixture of 1,4-dioxane and water. Cationic conjugate **CB3** (39 mg, 0.046 mmol) was obtained as orange colored lyophilizate in 16% yield. ^**1**^ **H NMR** (400 MHz, CDCl_3_) δ ppm: 1.44 (br s, 1 H, linker), 1.68 (br s, 3 H, linker), 1.85–2.01 (m, 5 H, linker) *overlap with* 1.95 (s, 3 H, C*H*_3_CO_2_^–^), 2.08–2.27 (m, 2 H, linker C*H*_2_), 2.36 (br s, 6 H, BODIPY 2 × C*H*_3_), 2.48 (s, 6 H, BODIPY 2 × C*H*_3_), 2.74 (br t, *J* = 12.5 Hz, 1 H, Col C*H*), 3.00 (br s, 2 H, C*H*_2_), 3.10–3.19 (m, 1 H, C*H*), 3.27 (br s, 3 H, -N^+^C*H*_3_), 3.32 (br s, 3 H, -N^+^C*H*_3_), 3.48 (br t, *J* = 8.0 Hz, 2 H, C*H*_2_-NMe_2_^+^-), 3.62 (s, 3 H, Col OC*H*_3_), 3.87 (s, 3 H, Col OC*H*_3_), 3.88 (br s, 3 H, Col OC*H*_3_), 3.91 (s, 3 H, Col OC*H*_3_), 4.43–4.52 (m, 1 H, −C*H*-NH−), 6.03 (s, 2 H, 2× BODIPY C*H*), 6.51 (s, 1 H, Col Ar*H*), 6.75 (d, *J* = 10.6 Hz, 1 H, tropolone C*H*), 7.22 (dd, *J* = 10.6, 1.6 Hz, 1 H, tropolone C*H*), 7.45 (s, 1 H, tropolone C*H*), 10.01 (br d, *J* = 5.5 Hz, 1 H, N*H*); Figure S10A. ^**13**^ **C NMR** (101 MHz, CDCl_3_) δ: 14.48 (t, *J*_CF_ = 2.3 Hz), 16.56, 20.72, 22.13, 22.73, 25.02, 27.38, 27.88, 29.99, 33.09, 35.39, 50.50, 50.97, 53.30, 56.06, 56.14, 61.37, 61.57, 64.02, 64.38, 107.26, 112.36, 122.02, 125.74, 131.04, 131.34, 134.67, 134.95, 136.93, 140.31, 141.37, 144.35, 151.03, 152.45, 153.34, 154.39, 163.70, 172.59, 176.98, 179.25; Figure S10B. **HRMS-ESI**: for C_44_H_58_BF_2_N_4_O_6_^+^ calcd 787.44120 Da, found *m*/*z* 787.44182 [M]^+^; Figure S10C. **HPLC**: R_T_ = 5.324 min; Figure S10D.

#### General Procedure for the Synthesis of C-7 Clickates

The **C2** (1 equiv), BODIPY alkyne **B8–B10** (1.3 equiv), TBTA (0.05 equiv) were dissolved in *tert-*BuOH (5 mL) in a microwave vial. To this mixture aqueous solutions of CuSO_4_·5H_2_O (0.1 equiv) and sodium ascorbate (0.2 equiv) were added. The vial was then closed with pressure cap and placed onto a microwave reactor (MW) and heated to 85 °C for 20 min. Than the mixture was allowed to cool to RT and poured into CHCl_3_ (30 mL). The organic layer was washed with saturated brine (2 × 20 mL). Separated organic layer was dried over MgSO_4_, filtered, and the solvents were evaporated under reduced pressure. The residue was chromatographed. After analytical characterizations the samples were lyophilized from 1,4-dioxane and stored in plastic vials in the fridge.

##### Clickate **CB4**

In reaction: **C2** (75 mg, 0.19 mmol) and **B9** (78 mg, 0.25 mmol). Chromatography (2×): DCM-MeOH 100:1 (*v/v*). **CB4** (72 mg, 0.1 mmol) was obtained as orange lyophilizate in 54% yield. **R**_**F**_ = 0.3 in AcOEt. ^**1**^ **H NMR** (400 MHz, CDCl_3_) δ ppm: 1.94–2.04 (m, 2 H, linker C*H*_2_), 2.33 (s, 6 H, BODIPY 2 × C*H*_3_), 2.48 (s, 6 H, BODIPY 2 × C*H*_3_), 2.50–2.64 (m, 2 H, Col C*H*_2_), 2.68–2.81 (m, 2 H, Col C*H*_2_), 2.92 (t, *J* = 7.4 Hz, 2 H, linker C*H*_2_), 3.01–3.09 (m, 2 H, linker C*H*_2_), 3.75 (s, 3 H, Col OC*H*_3_), 3.91 (s, 3 H, Col OC*H*_3_), 3.93 (s, 3 H, Col OC*H*_3_), 3.96 (s, 3 H, Col OC*H*_3_), 5.37 (dd, *J* = 11.9, 5.7 Hz, 1 H, Col–C*H*-triazole), 6.02 (s, 2 H, BODIPY 2 × C*H*), 6.51 (s, 1 H, Col Ar*H*), 6.58 (s, 1 H, tropolone C*H*), 6.79 (d, *J* = 11.0 Hz, 1 H, tropolone C*H*), 7.28 (d, *J* = 10.2 Hz, 1 H, tropolone C*H*), 7.37 (s, 1 H, triazole C*H*); Figure S11A. ^**13**^ **C NMR** (101 MHz, CDCl_3_) δ ppm: 14.43 (t, *J*_CF_ = 2.7 Hz), 16.40, 26.03, 27.80, 29.72, 31.22, 35.48, 56.12, 56.40, 61.21, 61.30, 62.56, 107.35, 111.71, 121.40, 121.67, 124.93, 131.41, 131.75, 133.54, 134.37, 135.40, 140.43, 141.67, 145.59, 147.00, 147.64, 151.01, 153.88, 153.93, 164.25, 178.82; Figure S11B. **HRMS-ESI**: for C_38_H_42_BF_2_N_5_O_5_ calcd 697.32471 Da, found *m*/*z* 720.31445 [M + Na]^+^; Figure S11C. **HPLC**: R_T_ = 5.325 min; Figure S11D.

##### Clickate **CB5**

In reaction: **C2** (100 mg, 0.26 mmol) and **B10** (130 mg, 0.34 mmol). Chromatography (2×): DCM-MeOH 100:1 (*v/v*). **CB5** (160 mg, 0.21 mmol) was obtained as orange lyophilizate in 80% yield. **R**_**F**_ = 0.5 in AcOEt. ^**1**^ **H NMR** (400 MHz, CDCl_3_) δ ppm: 1.33 (br s, 6 H, linker 3× C*H*_2_), 1.41–1.51 (m, 2 H, linker C*H*_2_), 1.54–1.69 (m, 4 H, linker 2 × C*H*_2_), 2.38 (s, 6 H, BODIPY 2 × C*H*_3_), 2.49 (s, 6 H, BODIPY 2 × C*H*_3_), 2.51–2.62 (m, 2 H, Col C*H*_2_), 2.66–2.74 (m, 4 H, linker C*H*_2_ and Col C*H*_2_), 2.85–2.95 (m, 2 H, linker C*H*_2_), 3.74 (s, 3 H, Col OC*H*_3_), 3.89 (s, 3 H, Col OC*H*_3_), 3.92 (s, 3 H, Col OC*H*_3_), 3.94 (s, 3 H, Col OC*H*_3_), 5.36 (dd, *J* = 11.9, 5.7 Hz, 1 H, ColC*H*-triazole), 6.02 (s, 2 H, BODIPY 2 × C*H*), 6.52 (s, 1 H, Col Ar*H*), 6.57 (s, 1 H, tropolone C*H*), 6.78 (d, *J* = 11.0 Hz, 1 H, tropolone C*H*), 7.26 (d, *J* = 11.0 Hz, 1 H, tropolone C*H*), 7.33 (s, 1 H, triazole C*H*); Figure S12A. ^**13**^ **C NMR** (101 MHz, CDCl_3_) δ ppm: 14.41 (t, *J*_CF_ = 2.3 Hz), 16.37, 25.70, 28.44, 29.18, 29.25, 29.28, 29.72, 30.35, 31.86, 35.41, 56.12, 56.37, 61.19, 61.27, 62.43, 107.34, 111.70, 120.94, 121.51, 124.99, 131.40, 131.86, 133.62, 134.45, 135.28, 140.34, 141.62, 146.71, 147.83, 148.57, 150.98, 153.59, 153.88, 164.22, 178.89; Figure S12B. **HRMS-ESI**: for C_43_H_52_BF_2_N_5_O_5_ calcd 767.40296 Da, found *m*/*z* 790.39281­[M + Na]^+^; Figure S12C. **HPLC**: R_T_ = 6.279 min; Figure S12D.

##### Clickate **CB6**

In reaction: **C2** (100 mg, 0.26 mmol) and **B8** (139 mg, 0.34 mmol). Chromatography (2×): CHCl_3_-MeOH 100:1 (*v/v*). **CB6** (173 mg, 0.22 mmol) was obtained as orange lyophilizate in 84% yield. **R**_**F**_ = 0.5 in hexane-AcOEt 1:1 (*v/v*). ^**1**^ **H NMR** (400 MHz, CDCl_3_) δ ppm: 1.40 (s, 3 H, BODIPY C*H*_3_), 1.44 (s, 3 H, BODIPY C*H*_3_), 2.53 (s, 6 H, BODIPY 2 × C*H*_3_), 2.55–2.65 (m, 2 H, Col C*H*_2_), 2.69–2.83 (m, 2 H, Col C*H*_2_), 3.76 (s, 3 H, Col OC*H*_3_), 3.81 (s, 3 H, BODIPY OC*H*_3_), 3.91 (s, 3 H, Col OC*H*_3_), 3.93 (s, 3 H, Col OC*H*_3_), 3.95 (s, 3 H, Col OC*H*_3_), 5.33 (s, 2 H, -OC*H*_2_-triazole), 5.41 (dd, *J* = 11.9, 5.7 Hz, 1 H, ColC*H*-triazole), 5.96 (s, 1 H, BODIPY C*H*), 5.97 (s, 1 H, BODIPY C*H*), 6.49 (s, 1 H, Col Ar*H*), 6.59 (s, 1 H, tropolone C*H*), 6.78 (s, 1 H, BODIPY Ar*H*), 6.79–6.82 (m, 2 H, BODIPY 2 × Ar*H*), 7.14 (d, *J* = 7.8 Hz, 1 H, tropolone C*H*), 7.28 (d, *J* = 11.0 Hz, 1 H, tropolone C*H*), 7.72 (s, 1 H, triazole C*H*); Figure S13A. ^**13**^ **C NMR** (101 MHz, CDCl_3_) δ ppm: 15.29, 15.40, 15.51, 30.65, 36.34, 57.05, 57.07, 57.34, 62.14, 62.23, 63.70, 64.28, 108.33, 112.46, 112.68, 115.90, 121.42, 121.98, 122.09, 124.36, 125.80, 129.20, 132.51, 132.57, 132.71, 134.43, 135.26, 136.40, 142.34, 142.64, 143.97, 144.18, 145.02, 148.42, 148.99, 151.36, 151.95, 154.92, 156.18, 156.41, 165.22, 179.72; Figure S13B. **HRMS-ESI**: for C_43_H_44_BF_2_N_5_O_7_ calcd 791.33019 Da, found *m*/*z* 814.31994 [M + Na]^+^; Figure S13C. **HPLC**: R_T_ = 5.399 min; Figure S13Dhe.

#### General Procedure for the Synthesis of *N*-Methyl Triazolium Salts

In an argon flushed flask clickate **CB4**, **CB5** or **CB6** was dissolved in dry AcCN (3 mL) and methyl iodide (500 μL, 8 mmol) in AcCN (1 mL) was added dropwise at RT under argon. The reaction was stirred for 4 days at the dark at RT. Solvents were evaporated under reduced pressure and the residue was dissolved in DCM (5 mL) and poured into Et_2_O (40 mL). The solids formed were collected by filtration on a paper and dried *in vacuo*. The material thus obtained was chromatographed (CHCl_3_-MeOH 20:1→5:1, *v/v*). The products were dissolved in small volume of MeOH and dropwise added to Et_2_O at 0 °C. The solids were collected by filtration on a paper and dried. After analytical characterizations the samples were lyophilized from 1,4-dioxane and stored in plastic vials in the fridge.

##### Triazolium **CB7**

In reaction: **CB4** (100 mg, 0.13 mmol). **CB7** (38 mg, 0.045 mmol) was obtained as orange colored lyophilizate in 35% yield. **R**_**F**_ = 0.3 in DCM-MeOH-TEA 10:1:0.01 (*v/v/v*). ^**1**^ **H NMR** (400 MHz, CDCl_3_) δ ppm: 2.06–2.24 (m, 2 H, linker C*H*_2_), 2.43 (br s, 12 H, BODIPY 4 × C*H*_3_), 2.48 (s, 3 H, *isomer*), 2.68–2.99 (m, 4 H, Col–C*H*_2_ and linker–C*H*_2_), 3.03–3.33 (m, 4 H, Col C*H*_2_ and linker C*H*_2_), 3.56 (s, 1 H, *isomer*), 3.59 (s, 1 H, *isomer*), 3.69 (s, 1 H, *isomer*), 3.77 (s, 3 H, Col OC*H*_3_), 3.89 (s, 3 H, Col OC*H*_3_), 3.90 (br s, 1 H, *isomer*), 3.91 (s, 3 H, Col OC*H*_3_), 3.99 (br s, 1 H, *isomer*), 3.99 (s, 1 H, *isomer*), 4.25 (s, 3 H, triazole C*H*_3_), 5.52 (dd, *J* = 11.3, 6.3 Hz, 1 H, ColC*H*-triazole), 5.74 (br d, *J* = 5.5 Hz, 1 H, *isomer*), 6.02 (s, 2 H, BODIPY C*H*_2_), 6.07 (br s, 1 H), 6.51 (s, 1 H, Col Ar*H*), 6.59 (s, 1 H, tropolone C*H*), 6.68 (s, 1 H, *isomer*), 6.93 (d, *J* = 11.0 Hz, 1 H, tropolone C*H*), 6.97 (d, *J* = 11.0 Hz, 1 H, *isomer*), 7.37 (d, *J* = 11.0 Hz, 1 H, tropolone C*H*), 7.61 (s, 1 H, *isomer*), 8.46 (br s, 1 H, triazole C*H*), 8.59 (s, 1 H, *isomer*); Figure S14A. ^**13**^ **C NMR** (101 MHz, CDCl_3_) δ: 14.44, 17.31, 24.35, 27.16, 28.39, 29.19, 34.85, 39.27, 56.20, 57.12, 61.18, 61.69, 66.75, 107.93, 113.29, 122.11, 123.53, 129.69, 131.28, 132.72, 134.82, 136.86, 141.77, 143.68, 145.10, 145.67, 150.85, 154.53, 164.33, 178.43; Figure S14B. **HRMS-ESI**: for C_39_H_45_BF_2_N_5_O_5_^+^ calcd 712.34763 Da, found *m*/*z* 712.34833 [M]^+^; Figure S14C. **HPLC**: R_T_ = 11.352 min; Figure S14D.

##### Triazolium **CB8**

In reaction: **CB5** (100 mg, 0.13 mmol). **CB8** (29 mg, 0.032 mmol) was obtained as orange colored lyophilizate in 25% yield. **R**_**F**_ = 0.4 in DCM-MeOH 10:1 (*v/v*). ^**1**^ **H NMR** (400 MHz, CDCl_3_) δ ppm: 1.34 (br s, 6 H, linker 3 × C*H*_2_), 1.38–1.51 (m, 2 H, linker C*H*_2_), 1.54–1.66 (m, 2 H, linker C*H*_2_), 1.67–1.87 (m, 2 H, linker C*H*_2_), 2.38 (s, 6 H, BODIPY 2 × C*H*_3_), 2.48 (s, 6 H, BODIPY 2 × C*H*_3_), 2.51–2.57 (m, 2 H, Col C*H*_2_), 2.71–2.85 (m, 4 H, linker C*H*_2_ and Col C*H*_2_), 2.85–2.97 (m, 2 H, linker C*H*_2_), 3.54 (s, 3 H, C*H*_3_ *isomer*), 3.82 (s, 3 H, Col OC*H*_3_), 3.90 (s, 3 H, Col OC*H*_3_), 3.93 (s, 3 H, Col OC*H*_3_), 3.99 (s, 3 H, Col OC*H*_3_), 4.25 (s, 3 H, triazole C*H*_3_), 5.65 (br dd, *J* = 11.7, 5.9 Hz, 1 H, ColC*H*-triazole), 6.02 (s, 2 H, BODIPY 2 × C*H*), 6.36 (s, 1 H, Col Ar*H*), 6.59 (s, 1 H, tropolone C*H*), 6.89 (d, *J* = 11.0 Hz, 1 H, tropolone C*H*), 7.35 (d, *J* = 11.0 Hz, 1 H, tropolone C*H*), 8.29 (br d, *J* = 4.3 Hz, 1 H, triazole C*H*); Figure S15A. ^**13**^ **C NMR** (101 MHz, CDCl_3_) δ ppm: 14.41 (t, *J*_CF_ = 2.7 Hz), 16.40, 23.73, 26.55, 28.37, 29.01, 29.11, 29.17, 29.28, 30.24, 31.85, 34.59, 38.04, 54.17, 56.11, 56.67, 61.12, 61.55, 66.54, 107.82, 112.71, 121.55, 123.63, 129.24, 131.39, 132.86, 134.77, 136.72, 140.34, 141.72, 145.83, 146.57, 150.89, 153.63, 154.46; Figure S15B. **HRMS-ESI**: for C_44_H_55_BF_2_N_5_O_5_^+^ calcd 782.42588 Da, found *m*/*z* 782.42663 [M]^+^; Figure S15C. **HPLC**: R_T_ = 5.717 min; Figure S15D.

##### Triazolium **CB9**

In reaction: **CB6** (100 mg, 0.13 mmol). **CB9** (51 mg, 0.055 mmol) was obtained as orange lyophilizate in 42% yield. **R**_**F**_ = 0.4 in DCM-MeOH 10:1 (*v/v*). ^**1**^ **H NMR** (400 MHz, CDCl_3_) δ ppm: 1.33 (s, 3 H, BODIPY C*H*_3_), 1.39 (s, 3 H, BODIPY C*H*_3_), 1.99 (s, 1 H), 2.54 (s, 6 H, 2× BODIPY C*H*_3_), 2.55–2.60 (m, 2 H, Col–C*H*_2_; *overlap with* BODIPY C*H*_3_), 2.73–2.90 (m, 3 H, Col 3 × C*H*), 3.55 (s, 3 H, C*H*_3_ *isomer*), 3.83 (s, 3 H, BODIPY OC*H*_3_), 3.84 (s, 3 H, Col OC*H*_3_), 3.91 (s, 3 H, Col OC*H*_3_), 3.92 (s, 3 H, Col OC*H*_3_), 3.94 (s, 2 H, Col OC*H*_3_), 4.56 (s, 3 H, triazole C*H*_3_), 5.42–5.55 (m, 2 H, -OC*H*_2_-triazole), 5.62 (dd, *J* = 11.0, 6.3 Hz, 1 H, Col C*H*-triazole), 5.94 (s, 1 H, BODIPY C*H*), 5.97 (s, 1 H, BODIPY C*H*), 6.37 (s, 1 H, Col Ar*H*), 6.59 (s, 1 H, tropolone C*H*), 6.78–6.86 (m, 3 H, BODIPY 3 × Ar*H*), 7.15 (d, *J* = 8.6 Hz, 1 H, tropolone C*H*), 7.35 (d, *J* = 11.0 Hz, 1 H, tropolone C*H*), 8.62 (s, 1 H, triazole C*H*); Figure S16A. ^**13**^ **C NMR** (101 MHz, CDCl_3_) δ ppm: 14.32, 14.42, 14.58, 29.31, 34.61, 39.44, 54.31, 56.10, 56.24, 56.58, 60.66, 61.11, 61.55, 66.66, 107.85, 112.27, 112.39, 118.14, 120.95, 121.15, 121.25, 123.63, 130.77, 131.38, 131.45, 132.75, 134.21, 136.64, 140.47, 140.68, 141.83, 142.88, 143.01, 145.57, 146.34, 150.95, 151.28, 154.46, 155.47, 155.67, 164.48, 178.31; Figure S16B. **HRMS-ESI**: for C_44_H_47_BF_2_N_5_O_7_^+^ calcd 806.35311 Da, found *m*/*z* 806.35308 [M]^+^; Figure S16C. **HPLC**: R_T_ = 5.221 min; Figure S16D.

#### General Procedure for the Synthesis of C-10 Derivatives

The **C1** (250 mg, 0.63 mmol) and **B4** (102 mg, 0.32 mmol) or **B5** (107 mg, 0.32 mmol) were dissolved in MeOH (15 mL). The reaction mixture was heated to 70 °C ON. The heating was removed and the solvent was evaporated under reduced pressure. The residue was chromatographed (AcOEt-MeOH 25:1→15:1, *v/v*). The matter thus obtained was dissolved in the mixture of MeOH-DCM (4:16, 20 mL) and the product was precipitated by the addition of Et_2_O-hexane (1:1) mixture. The solids formed were immediately collected by filtration on a paper and dried *in vacuo*. After analytical characterizations the samples were lyophilized from 1,4-dioxane and stored in plastic vials in the fridge.

##### Amine **CB10**

The **CB10** (180 mg, 0.26 mmol) was obtained as orange colored lyophilizate in 82% yield. **R**_**F**_ = 0.4 in AcOEt-MeOH 10:1 (*v/v*). ^**1**^ **H NMR** (400 MHz, CDCl_3_) δ ppm: 1.71–1.84 (m, 2 H, linker C*H*_2_), 1.94 (s, 3 H, Col NHCOC*H*_3_), 1.95–2.00 (m, 2 H, Col C*H*_2_), 2.17–2.34 (m, 2 H, Col C*H*_2_), 2.36 (s, 6 H, BODIPY 2 × C*H*_3_), 2.49 (s, 6 H, BODIPY 2 × C*H*_3_), 2.93–3.01 (m, 2 H, linker C*H*_2_), 3.35–3.48 (m, 2 H, linker C*H*_2_), 3.61 (s, 3 H, Col OC*H*_3_), 3.88 (s, 3 H, Col OC*H*_3_), 3.93 (s, 3 H, Col OC*H*_3_), 4.67 (dt, *J* = 12.3, 6.4 Hz, 1 H, Col C*H*-NHAc), 6.02 (s, 2 H, BODIPY 2 × C*H*), 6.52 (s, 1 H, Col Ar*H*), 6.58 (d, *J* = 11.3 Hz, 1 H, tropolone C*H*), 7.22 (t, *J* = 5.7 Hz, 1 H, N*H*), 7.43 (d, *J* = 11.0 Hz, 1 H, tropolone C*H*), 7.57 (s, 1 H, tropolone C*H*), 8.54 (br s, 1 H, N*H*); Figure S17A. ^**13**^ **C NMR** (101 MHz, CDCl_3_) δ ppm: 14.42 (t, *J*_CF_ = 2.3 Hz), 16.31, 22.71, 27.80, 28.94, 29.13, 30.07, 37.01, 42.22, 52.70, 56.12, 56.13, 61.32, 61.34, 61.39, 107.25, 108.37, 121.79, 123.00, 126.82, 130.74, 131.34, 134.54, 139.17, 140.15, 141.56, 145.24, 151.05, 151.65, 152.95, 153.96, 154.07, 169.96, 175.01; Figure S17B. **HRMS-ESI**: for C_38_H_45_BF_2_N_4_O_5_ calcd 686.34511 Da, found *m*/*z* 709.33502 [M + Na]^+^; Figure S17C. **HPLC**: R_T_ = 5.856 min; Figure S17D.

##### Amine **CB11**

The **CB11** (159 mg, 0.23 mmol) was obtained as orange colored lyophilizate in 72% yield. **R**_**F**_ = 0.2 in DCM-MeOH 10:1 (*v/v*). ^**1**^ **H NMR** (400 MHz, CDCl_3_) δ ppm: 1.59–1.96 (m, 5 H, linker 2 × C*H*_2_ and Col C*H*_2_), 1.98 (s, 3 H, Col NHCOC*H*_3_), 2.05–2.20 (m, 1 H, Col C*H*), 2.27–2.35 (m, 1 H, Col C*H*), 2.37 (s, 6 H, BODIPY 2 × C*H*_3_), 2.48 (s, 6 H, BODIPY 2 × C*H*_3_), 2.92–3.00 (m, 2 H, linker C*H*_2_), 3.08 (s, 3 H), 3.56–3.74 (m, 2 H, linker C*H*_2_), 3.62 (s, 3 H, Col OC*H*_3_), 3.85 (s, 3 H, Col OC*H*_3_), 3.91 (s, 3 H, Col OC*H*_3_), 4.56 (dt, *J* = 12.1, 6.7 Hz, 1 H, Col C*H*-NHAc), 6.01 (s, 2 H, BODIPY 2 × C*H*), 6.46 (s, 1 H, Col Ar*H*), 6.54 (d, *J* = 11.3 Hz, 1 H, tropolone C*H*), 7.16 (s, 1 H, tropolone C*H*), 7.22 (d, *J* = 11.3 Hz, 1 H, tropolone C*H*), 7.68 (d, *J* = 7.0 Hz, 1 H, N*H*); Figure S18A. ^**13**^ **C NMR** (101 MHz, CDCl_3_) δ ppm: 14.42 (t, *J*_CF_ = 2.3 Hz), 16.40, 22.91, 28.05, 28.61, 29.29, 30.05, 37.07, 40.56, 51.85, 53.23, 56.07, 61.30, 61.37, 107.27, 113.54, 121.69, 126.09, 126.23, 130.83, 131.35, 134.41, 136.41, 140.21, 141.52, 145.74, 149.62, 151.27, 152.87, 153.90, 156.89, 169.76, 179.79; Figure S18B. **HRMS-ESI**: for C_39_H_47_BF_2_N_4_O_5_ calcd 700.36076 Da, found *m*/*z* 723.35065 [M + Na]^+^; Figure S18C. **HPLC**: R_T_ = 5.898 min; Figure S18D.

##### Amide **CB12**

To a solution of **C4** (150 mg, 0.38 mmol) and **B1** (122 mg, 0.38 mmol) in DCM (5 mL), HBTU (185 mg, 0.49 mmol) and EDIPA (100 μL, 0.57 mmol) were added. The mixture was stirred under argon for 19 h. The solvents were evaporated under reduced pressure and the residue was purified by column chromatography (1. AcOEt-MeOH-TEA 20:1:0.01, *v/v/v*; 2. CHCl_3_-MeOH-TEA 25:1:0.01, *v/v/v*). The material obtained from the second purification was dissolved in the mixture of MeOH-DCM and precipitated by Et_2_O at 0 °C. The solids were collected by filtration on a paper, washed with Et_2_O (2 × 5 mL) and dried *in vacuo*. The **CB12** (120 mg, 0.17 mmol) was obtained as a dark orange matter in 45% yield. **R**_**F**_ = 0.3 in DCM-MeOH 20:1 (*v/v*). ^**1**^ **H NMR** (400 MHz, CDCl_3_) δ ppm: 1.77 (td, *J* = 11.5, 5.9 Hz, 1 H, Col C*H*), 1.93 (s, 3 H, Col NHCOC*H*_3_), 2.08–2.20 (m, 1 H, Col C*H*), 2.38 (s, 6 H, BODIPY 2 × C*H*_3_), 2.43 (br s, 8 H, BODIPY 2 × C*H*_3_, *overlap with linker* C*H*_2_ *signal*), 2.45–2.51 (m, 2 H, Col C*H*_2_), 3.16 (s, 3 H, N–C*H*_3_), 3.27–3.41 (m, 2 H, linker C*H*_2_), 3.67 (s, 3 H, Col OC*H*_3_), 3.90 (s, 3 H, Col OC*H*_3_), 3.92 (s, 3 H, Col OC*H*_3_), 4.58 (dt, *J* = 12.1, 6.7 Hz, 1 H, Col C*H*-NHAc), 5.98 (s, 2 H, BODIPY 2 × C*H*), 6.51 (s, 1 H, Col Ar*H*), 7.16 (d, *J* = 10.2 Hz, 1 H, tropolone C*H*), 7.36 (d, *J* = 9.8 Hz, 1 H, tropolone C*H*), 7.40–7.42 (m, 1 H, tropolone C*H*); Figure S19A. ^**13**^ **C NMR** (101 MHz, CDCl_3_) δ ppm: 15.28 (t, *J*_CF_ = 2.3 Hz), 17.35, 23.73, 24.78, 30.75, 35.88, 37.00, 53.12, 57.01, 62.21, 62.64, 108.33, 122.64, 125.68, 132.21, 135.06, 135.14, 136.27, 137.06, 141.67, 142.62, 145.66, 145.98, 151.00, 152.16, 153.19, 155.12, 170.63, 172.19, 182.17; Figure S19B. **HRMS-ESI**: for C_38_H_43_BF_2_N_4_O_6_ calcd 700.32437 Da, found *m*/*z* 723.31443 [M + Na]^+^; Figure S19C. **HPLC**: R_T_ = 5.281 min; Figure S19D.

### Photochemical Properties of CBs

Absorption and fluorescence spectra were measured in ethanol using quartz cells of 1 cm path length. Absorption spectra were recorded using a Cary 60 (Agilent, Santa Clara, CA, USA) spectrophotometer (data interval 0.5 nm, averaging time 0.1 s). For fluorescence measurement, a Cary Eclipse fluorescence spectrophotometer (Agilent, Santa Clara, CA, USA) equipped with R3896 PMT (data interval 0.5 nm, averaging time 0.1 s, PMT voltage 600 V, excitation filter ″Auto″, emission filter ″Open″) was used. Absorption spectra were measured against air. The solvent spectra were recorded prior to addition of the concentrated sample solution and was subtracted during data processing in Microsoft Excel. The solution from the absorption cell was then further diluted to the ethanol in the fluorescence cell so that the maximum absorbance of the solution in fluorescence cell was lower than 0.1. Absorption spectrum of this solution was also measured.

The fluorescence spectra of the solvent measured under the same conditions were subtracted from the fluorescence spectra of the samples. The correction curves applied to the correction of excitation and emission spectra were measured using the method recommended by the manufacturer. The method is based on Rhodamine B quantum counter in a triangular cell and synchronous scan with a diffuser plate in a cell holder. In this way wavelength range of 220 to 600 nm was covered. The emission correction curve was then extrapolated to longer wavelengths using an incandescent lamp with coiled wire operated at several currents, which was considered to be approximately tungsten thermal radiator with single temperature. This temperature was evaluated by fitting its spectra in the wavelength range 400 to 580 nm, where the emission correction function was obtained in the previous step. From the extended emission correction function and diffuser plate synchronous spectrum, also excitation correction function in the red was evaluated. The relative fluorescence quantum yields were evaluated in relation to the value of 0.51 for quinine in 0.05 M sulfuric acid.^63^

### Biochemistry and Cell Analysis

#### Cell Lines

The cancer cell lines CCRF-CEM (acute lymphoblastic leukemia), K562 (chronic myelogenous leukemia), U2OS (osteosarcoma), normal human fibroblasts MRC-5 and BJ were purchased from the American Tissue Culture Collection (ATCC) and Human umbilical vein endothelial cells HUVEC from PromoCell. Colorectal cancer cell lines HCT116 and HCT116p53–/– (null p53 gene) were purchased from Horizon Discovery. Resistant clones CEM-DNR (resistant to daunorubicin) and K562-TAX (resistant to paclitaxel) were established in IMTM laboratory.^45^ The cells were maintained and subcultured according to ATCC or Horizon recommendations under the following conditions: CCRF-CEM, CEM-DNR and K562-TAX (RPMI 1640, 10% FBS, 100 U/mL Penicillin-Steptomycin); K562 (IMDM, 10% FBS, 100 U/mL Penicillin-Steptomycin); U2OS, HCT116 and HCT116p53–/– (McCoy′s 5A modified medium 10% FBS, 100 U/mL Penicillin-Steptomycin); MRC-5 and BJ (EMEM, 10% FBS, 100 U/mL Penicillin-Steptomycin); HUVEC (Endothelial cell growth medium with Supplement Mix for endothelial cells). Cells were incubated at 37 °C in a 5% CO_2_ atmosphere of a humidified incubator.

#### Cytotoxicity Assay

Cell suspensions were prepared and diluted according to the particular cell type and the expected target cell density (500 – 4,000 cells/well). The cells (30 μL/well) were seeded to 384-well clear Corning plates by MultiDrop Combi (Thermo Fisher Scientific, USA). After 24 h the cells were treated with tested compounds as well as with vehicle (DMSO) and high controls (2.67 μM of Actinomycin D and 100 μM Mitomycin C) using contact-free acoustic liquid handler ECHO 555 (Labcyte, USA). Compounds were analyzed at a concentration range from 0.012 μM to 50 μM. Treated cells were incubated at 37 °C in a 5% CO_2_ atmosphere of a humidified incubator for 72 h. At the end of the incubation period, aliquots of the MTS (Promega) solution prepared according to manufacturer's instructions were added to cells (https://worldwide.promega.com/resources/protocols/technical-bulletins/0/celltiter-96-aqueous-nonradioactive-cell-proliferation-assay-protocol/). After 1 – 4 h of the incubation period, the optical density (OD) at 490 nm was measured on a multimode plate reader EnVision (PerkinElmer, USA). The experiments were performed in technical duplicates and at least three biological replicates. The IC_50_ values, the drug concentration lethal to 50% of the treated cells, were calculated from the appropriate dose–response curves by the Dotmatics software platform. The assay quality was monitored by determining the Z′-factor for each 384-well plate. The resistance index (RI), representing the reduction in compound activity on resistant cell lines, was calculated as RI = (IC_50_ of resistant cell lines, CEM-DNR, K562-TAX)/(IC_50_ of nonresistant counterparts, CCRF-CEM, K562). Similarly, the selectivity index (SI), indicating preferential cytotoxicity toward tumor cell lines, was calculated as SI = (mean IC_50_ of nontumor cell lines, BJ and MRC5)/(mean IC_50_ of cancer cell lines without resistant variants, CCRF-CEM, K562, HCT116, HCT116p53–/–, U2OS).

#### FACS Analysis

Cell cycle analysis and immunolabeling of cell cycle markers have been described previously.^64^ Briefly, CCRF-CEM were incubated with compounds for 24 h, then harvested, washed with cold phosphate-buffered saline (PBS), fixed in cold 70% ethanol, treated with RNase (0.5 mg/mL) and stained with propidium iodide (0.1 mg/mL). Data were acquired using the FACSCalibur (Becton Dickinson) and analyzed by the program ModFitLT (Verity). As a mitotic marker was used antiphospho-Histone H3 (Ser10) antibody (Merck Millipore). Primary antibody was diluted in blocking buffer and labeled with an antimouse-FITC conjugated secondary antibody (Sigma-Aldrich). Following the labeling, cells were washed with PBS and incubated with 0.1 mg/mL propidium iodide and 0.5 mg/mL RNase A for 1 h and analyzed by flow cytometry using a 488 nm single beam laser (FACSCalibur, Becton Dickinson). Data analysis was performed using CellQuest software.

#### Tubulin Polymerization Assay

The effect of compounds on tubulin assembly was assessed using the tubulin polymerization assay kit (Cytoskeleton). The kit was used according to the manufacturer’s protocol (see https://www.cytoskeleton.com/pdf-storage/datasheets/bk006p.pdf). Briefly, porcine brain tubulin (>99% pure) was dissolved to a final concentration of 3 mg/mL in tubulin polymerization buffer containing 80 mM PIPES pH 6.9, 2 mM MgCl_2_, 0.5 mM EGTA, 1 mM GTP, 10.2% glycerol. Changes in absorbance in the presence of 10 μM compounds or DMSO were measured at 340 nm using EnSpire Plate Reader (PerkinElmer) at 37 °C and recorded every 60 s for 50 min. The maximal velocity (*V*max) of tubulin polymerization was calculated for the growth phase of polymerization in each reaction. The growth phase was determined using linear regression of the OD data.

#### Endothelial Cell Tube Formation Angiogenesis Assay

The experiment was conducted on 96-well plates coated with 50 μL of Matrigel, a growth factor-reduced basement membrane matrix (Corning), and incubated for 1 h at 37 °C. Subsequently, HUVEC cells (15,000 cells/well), and dissolved test substances in a volume of 100 μL, were applied to this layer. The prepared plate was then incubated for 24 h. Then Calcein AM (final concentration 2 μg/mL, Invitrogen) was added to the control cells and cells treated with colchicine, followed by a 30 min incubation. The cells were then visualized using a confocal microscope (Zeiss).^65^

#### Fluorescence Microscopy

U2OS were incubated in the presence of colchicine, colchicine-BODIPY conjugates or the corresponding BODIPY moieties at concentrations of 0.1–10 μM for various time intervals before cell fixation and immunofluorescence examination or live cell imaging. DMSO at appropriate dilution served as control.

#### Immunofluorescence

Immunofluorescence microscopy on formaldehyde-fixed, Triton X-100 extracted cells (F/Tx) was performed as previously described.^66^ Extraction and fixation steps were carried out in a microtubule stabilizing buffer consisting of 0.1 M KMes, 2 mM EGTA, 2 mM MgCl_2_, 4% polyethylene glycol 6000, pH 6.9. Cells were fixed for 30 min in 3% formaldehyde before extraction for 4 min with 0.5% Triton X-100 at 37 °C. Mouse monoclonal antibody TU-01 (IgG1) directed to α-tubulin^67,68^ in the form of hybridoma spent culture medium was diluted 1:10. The DyLight 549-conjugated antimouse antibody (Jackson Immunoresearch Laboratories, West Grove, PA, USA) was diluted 1:500. Samples were mounted in MOWIOL 4–88 (Calbiochem, San Diego, CA, USA) supplemented with 4,6-diamidino-2-phenylindole (DAPI; Sigma-Aldrich, St. Louis, MO, USA) and examined with an Olympus AX-70 Provis microscope (Olympus, Hamburg, Germany) equipped with a 60×/1.0 water objective.

#### Live-Cell Imaging

Live-cell imaging was performed as described.^69^ Cells were grown on a 35 mm μ-Dish with a polymer coverslip bottom (Ibidi GmbH, Gräfelfing, Germany; Cat No. 81156). To visualize microtubules, the medium was replaced for FluoroBrite DMEM (Thermo Fisher Scientific, Waltham, MA, USA, Cat. No. A1896701) supplemented with 1% FS (FB) and 0.5 mM SiR-tubulin (Spirochrome AG, Stein am Rhein, Switzerland, Cat. No. SC002) 60 min before imaging. To visualize the endoplasmic reticulum, the medium was replaced with FB medium supplemented with 1 μM ER-Tracker ReD (Thermo Fischer Scientific, Cat. No. E34250) 15 min before imaging. To visualize mitochondria, the medium was replaced for FB medium supplemented with 100 nM Mito-tracker Orange (Thermo Fischer Scientific, Cat. No. M7510) 15 min before imaging. The colchicine-BODIPY conjugates or corresponding BODIPY moieties at a concentration of 1 μM were added 5 min before imaging. As control served DMSO carrier. The preparations were examined with the Andor Dragonfly 503 spinning disc confocal system (Oxford Instruments, Abingdon, UK) equipped with a stage top microscopy incubator (Okolab, Ottaviano, Italy), HCX PL APO 63×/1.2 water objective, and Zyla 4.2 PLUS sCMOS camera. The following illumination lasers and bandpass filters were used: green BODIPY conjugates (488 nm solid-state 150 mW laser; 525/50 nm bandpass filter), Mito-Tracker Orange (561 nm solid-state 100 mW laser, 600/50 nm bandpass filter), SiR-Tubulin, ER-Tracker ReD (637 nm solid-state 140 mW laser, 700/75 bandpass filter).

### *In Silico* Modeling

#### Molecular Modeling

For this study, the 3D complex (4O2B) of bovine tubulin alpha 1B (P81947) and beta-2B (Q6B856) chains with the known inhibitor colchicine was considered. We removed water molecules and native inhibitors from the structures. The 3D structure of the unresolved residues was rebuilt by Modeler Tool^70^ built-in Chimera.^71^ Remodeling of incomplete side chains and protonation of the protein structure were performed by Chimera Dock Prep tool.^71^ It is reported that both the GTP molecule and Mg^2+^ ion nearby the active site are important for the regulation of the polymerization thereby these crucial cofactors were kept.

#### Molecular Docking

All compounds were docked using Autodock Vina.^72^ Due to the large size of the docked molecules, we used a large docking box with a size of 25 × 26 × 26 Å centered around the active site. The exhaustiveness value was set to 96. Initial conformers were generated by RDKit 2019.03 version.^73^ Due to the inability to sample cycles by Autodock Vina, the initial conformation of the colchicine moiety for all compounds was assigned to the native conformation of colchicine in the 4O2B complex. Since Autodock Vina cannot process boron atoms we replaced boron atoms with carbon atoms to make docking. These atoms have similar van der Waals parameters and Autodock Vina ignores the charges of the atoms. Therefore, we consider such a replacement acceptable. Ligand protonation was performed by Marvin cxcalc utility at pH 7.4.^74^

#### Molecular Dynamics

We used GROMACS software version 2021.^75,76^ For target preparation, we used Amber 99SB-ILDN force field^77^ and the TIP3P water model. Na and Cl ions were added to neutralize the system. Ligand topologies were prepared by AmberTools version 20.9 together with the generated by Gaussian ESP charges for the boron center.^78^ Energy minimization took 50,000 steps for every simulation, followed by NVT and NPT equilibrations for 1000 ps each. Production simulations were conducted for 50 ns in an NPT ensemble at 310 K. For the visualization and analysis of the protein–ligand interaction we used the ProLIF package.^79^

### Biophysical Study

#### Surface Plasmon Resonance (SPR)

Surface plasmon resonance experiments were conducted using an SPR-24 Pro instrument (Bruker Daltonics GmbH & Co KG, USA). Porcine brain tubulin (>99% pure, Cytoskeleton, Inc., cat. #T240-DX, Denver, USA) was prediluted to 0.05 mg/mL in 10 mM sodium acetate buffer adjusted to pH 4.5 (Bruker Daltonics, part no: 1862646, USA) which was used as an immobilization buffer. Tubulin was immobilized onto a high-capacity amine sensor chip (Bruker Daltonics, part no: 1862614, USA) at 25 °C, achieving 15,000 Resonance Units (RU) on four detection cells of channel B. The immobilization process utilized EDC/NHS included in the amine coupling kit (Bruker Daltonics, part no: 1862634, USA). All samples and coupling agents were prepared in a polypropylene 96-well plate (Bruker Daltonics, part no: 1862984, USA) sealed with a precut sealer for 96-well (deep) microplates (Bruker Daltonics, part no: 1862985, USA) to minimize evaporation during immobilization and binding measurements. Four detection cells in channel A were used as a blank control. Interaction experiments were conducted in 10 mM sodium phosphate, 150 mM NaCl, and 0.05% Tween-20 running buffer at pH 7.5 (cat. no. 28352, Thermo Fisher Scientific, USA) at 25 °C. Eight different concentrations, each 2× diluted, of colchicine and **CB4**, ranging from 75 μM to 2.34 μM, were injected at a flow rate of 30 μL/min for 120 s, followed by a 300-s dissociation phase. Compound binding to tubulin was evaluated using Sierra SPR Control software (version 3.6.28.5, Bruker Daltonics GmbH & Co KG) on both flow cells. Measurements were conducted in duplicate. Data were processed using SPR-24 Pro analyzer software (Bruker Daltonics GmbH & Co KG) and analyzed with GraphPad Prism (version 10.2.2, 341). The data were fitted to a 1:1 steady-state model, and a four-parameter logistic (4PL) regression curve was employed to calculate the dissociation constant (*K*_d_).

## Supplementary Material



## Notes

1H, ^13^C NMR and HRMS spectra of new compounds; chromatograms of the tested compounds; absorption and emission spectra; effects of compounds on cellular activities and tubulin polymerization; molecular docking (PDF)

## ABBREVIATIONS

Col: colchicine
C-No: nonfluorescent colchicine precursor
B-No: nonconjugated BODIPY dye
CB: colchicine-BODIPY derivative
RT: room temperature
ON: overnight
