# Supplementary material for: Colchicine-BODIPY Probes: Evidence for the Involvement of Intracellular Membranes in the Targeting of Colchicine to Tubulin
Source: ACS Pharmacol Transl Sci. 2025 Jun 13;8(7):1965–85. doi: 10.1021/acsptsci.4c00730 (PMC12261228; doi:10.1021/acsptsci.4c00730)
Supplement: Supplementary file 1 [file pt4c00730_si_001.pdf]

Supporting Information

Colchicine-BODIPY probes: Evidence for the involvement of intracellular membranes in the targeting of colchicine to tubulin

Michal Jurášek[1a], Eduarda Dráberová[2], Jiří Řehulka[3], Soňa Gurská[3], Aleksandra Ivanova[3], Pavel Polishchuk[3], Kateřina Ječmeňová[3], Jan Fähnrich[1b], Anna Marešová[1a], Jan Tauchen[4], Atilio Reyes Romero[3], Alexander Dömling[3], Marián Hajdúch[3,5], Pavel B. Drašar[1a], Pavel Dráber[2] and Petr Džubák[3,5]**

[1]University of Chemistry and Technology Prague (UCT Prague), [a]Department of Chemistry of Natural Compounds, [b]Department of Analytical Chemistry, Technická 5, CZ-166 28 Praha 6, Czech Republic

[2]Institute of Molecular Genetics of the Czech Academy of Sciences, Department of Biology of Cytoskeleton, Vídeňská 1083, CZ-142 20 Praha 4, Czech Republic

[3]Institute of Molecular and Translational Medicine (IMTM), Faculty of Medicine and Dentistry, Palacký University and University Hospital in Olomouc, Hněvotínská 1333/5, CZ-779 00 Olomouc, Czech Republic

[4]Czech University of Life Sciences Prague, Department of Food Science, Faculty of Agrobiology, Food and Natural Resources, Kamýcká 129, CZ-165 00 Praha 6, Czech Republic

[5]Laboratory of Experimental Medicine, IMTM, University Hospital Olomouc, Hněvotínská 976/3, CZ-779 00 Olomouc, Czech Republic

Corresponding authors

Petr Džubák, e-mail: petr.dzubak@upol.cz

Pavel Dráber, e-mail: pavel.draber@img.cas.cz

Table of contents

1. ^{1}H NMR, ^{13}C NMR, HRMS of new compounds, chromatograms of the tested compounds and calculated lipophilicity, absorbtion and emission spectra S-3

2. Effects of compounds on cellular activities and tubulin polymerization S-25

3. Molecular docking S-71

1. ^{1}H NMR, ^{13}C NMR, HRMS of new compounds, chromatograms of the tested compounds and calculated lipophilicity, absorbtion and emission spectra

Tab. S1. Gradient system used for HPLC analyses

	***Time** [min]*	***A** (%)*	***B** (%)*
1	0-16	0	100
2	18	50	50
3	20	100	0

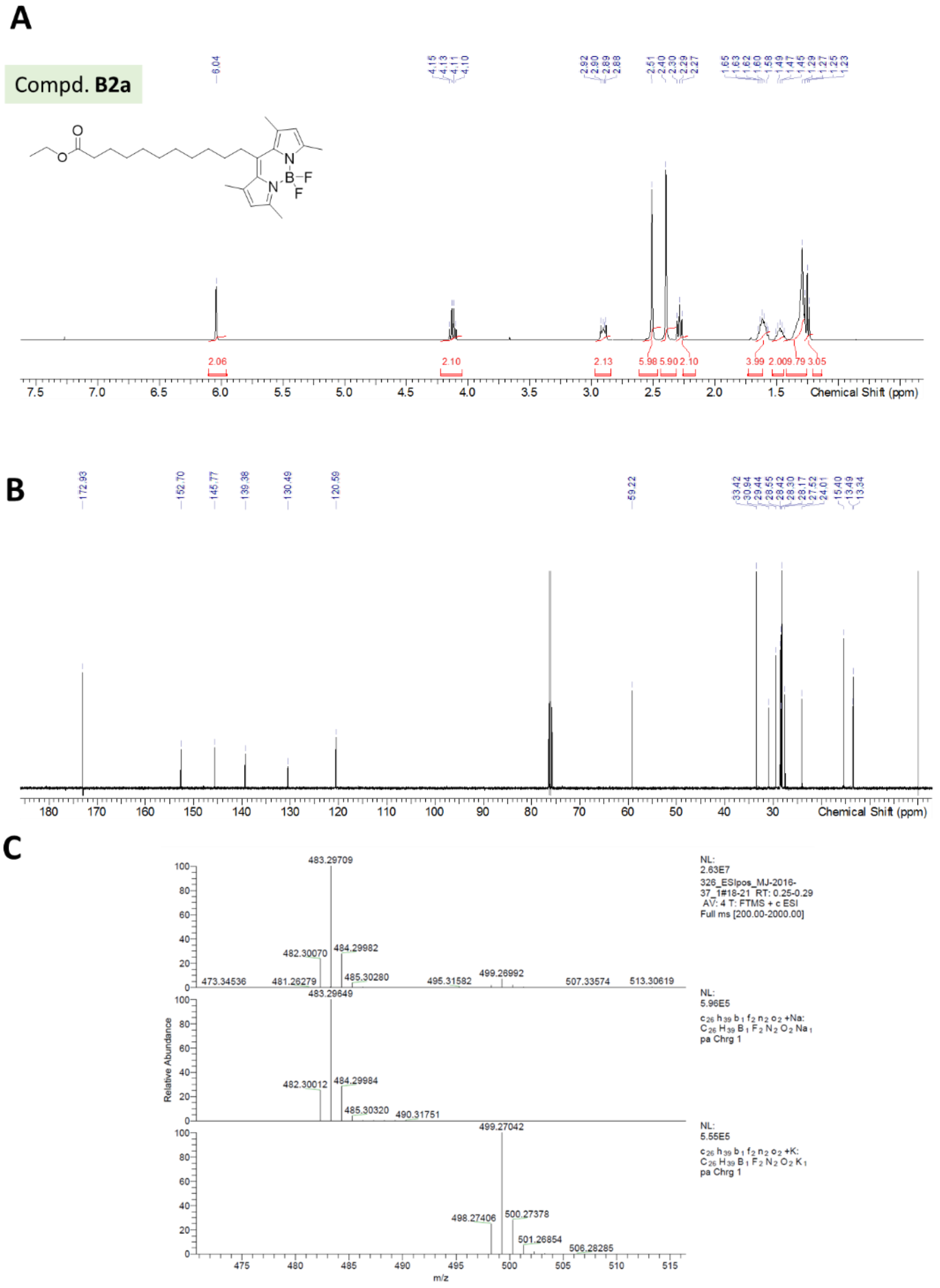

Fig. S1. ^{1}H NMR (A), ^{13}C NMR (B) and HRMS (C) of intermediate **B2a**

A

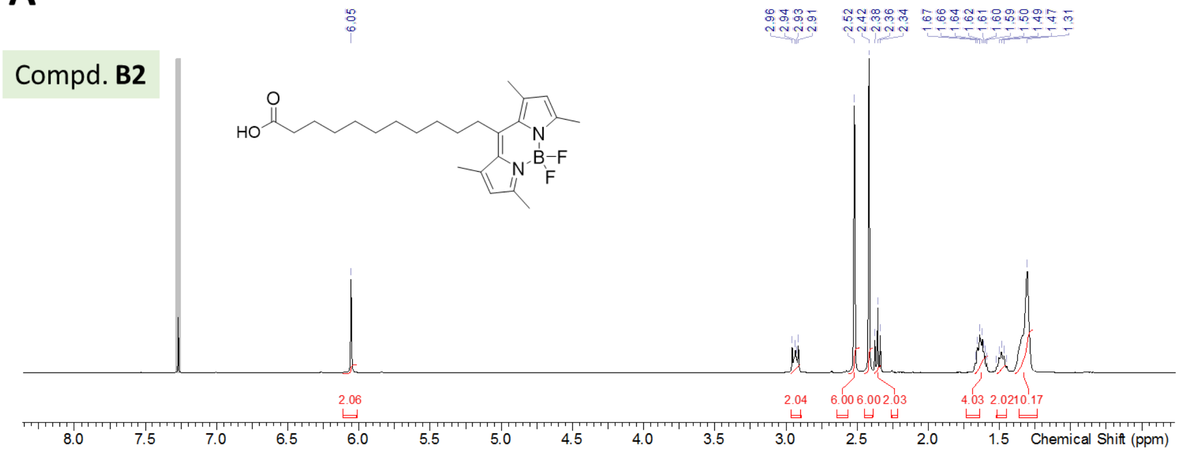

B

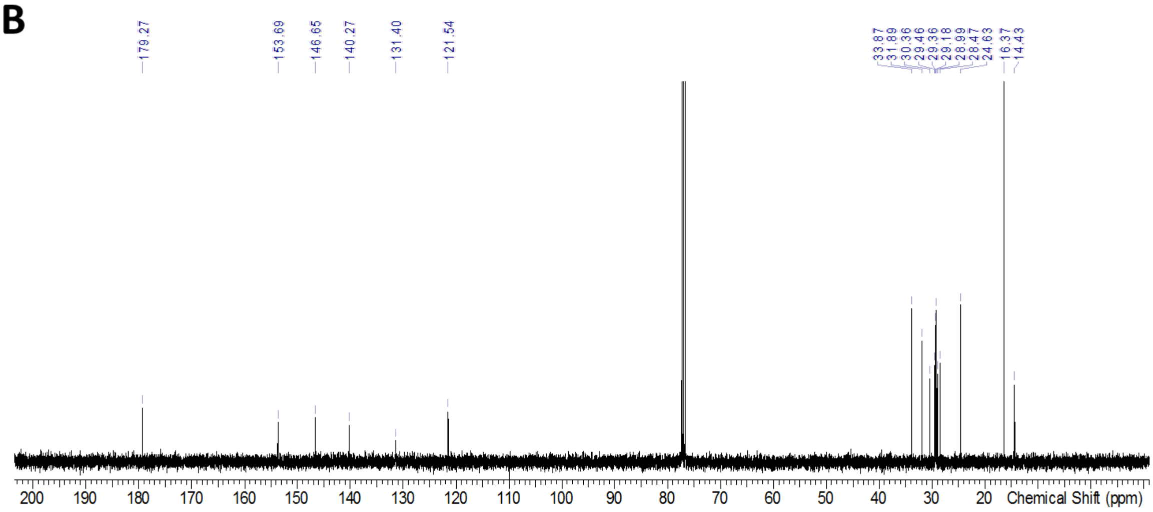

C

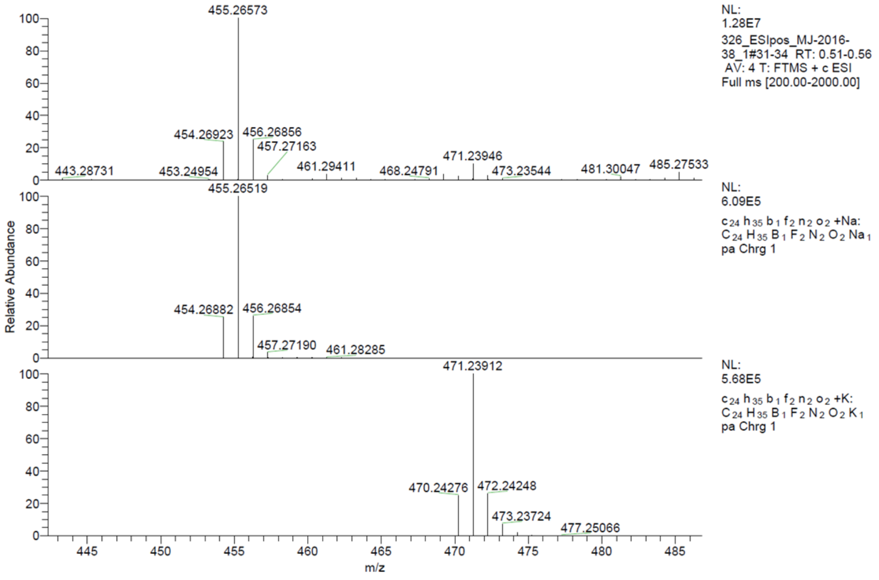

Fig. S2. ^{1}H NMR (A), ^{13}C NMR (B) and HRMS (C) of BODIPY **B2**

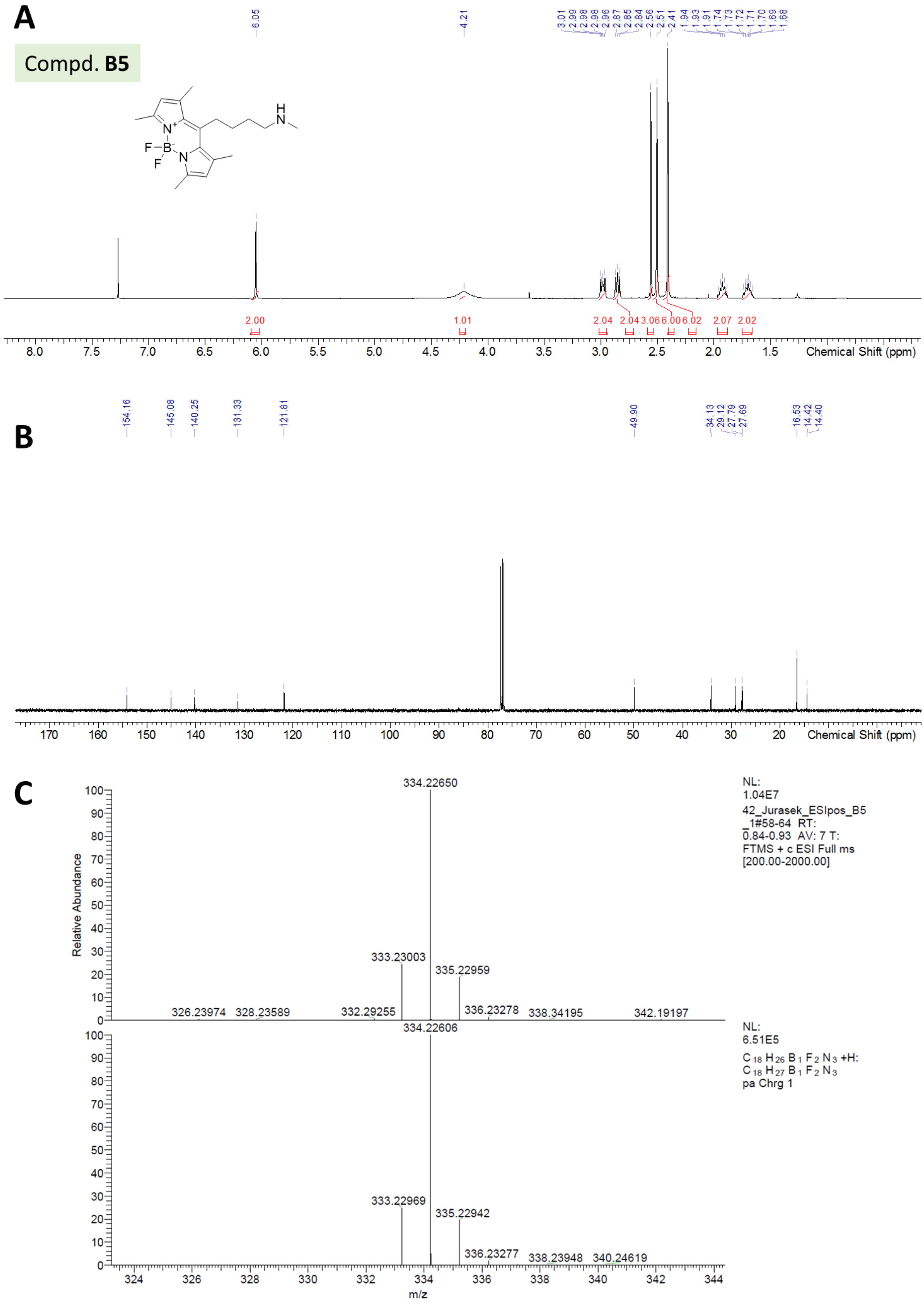

Fig. S3. ^{1}H NMR (A), ^{13}C NMR (B) and HRMS (C) of BODIPY **B5**

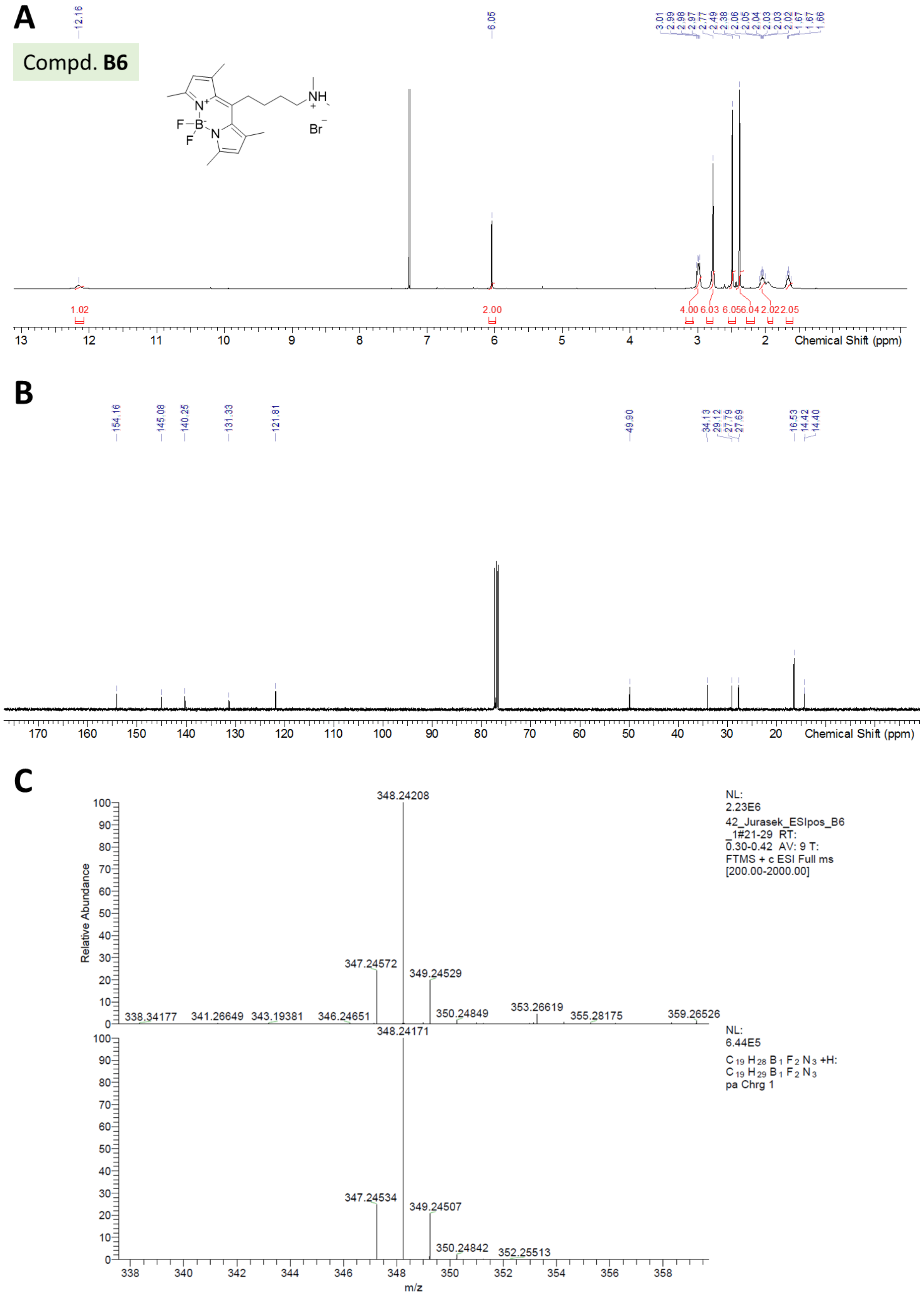

Fig. S4. ^{1}H NMR (A), ^{13}C NMR (B) and HRMS (C) of BODIPY **B6**

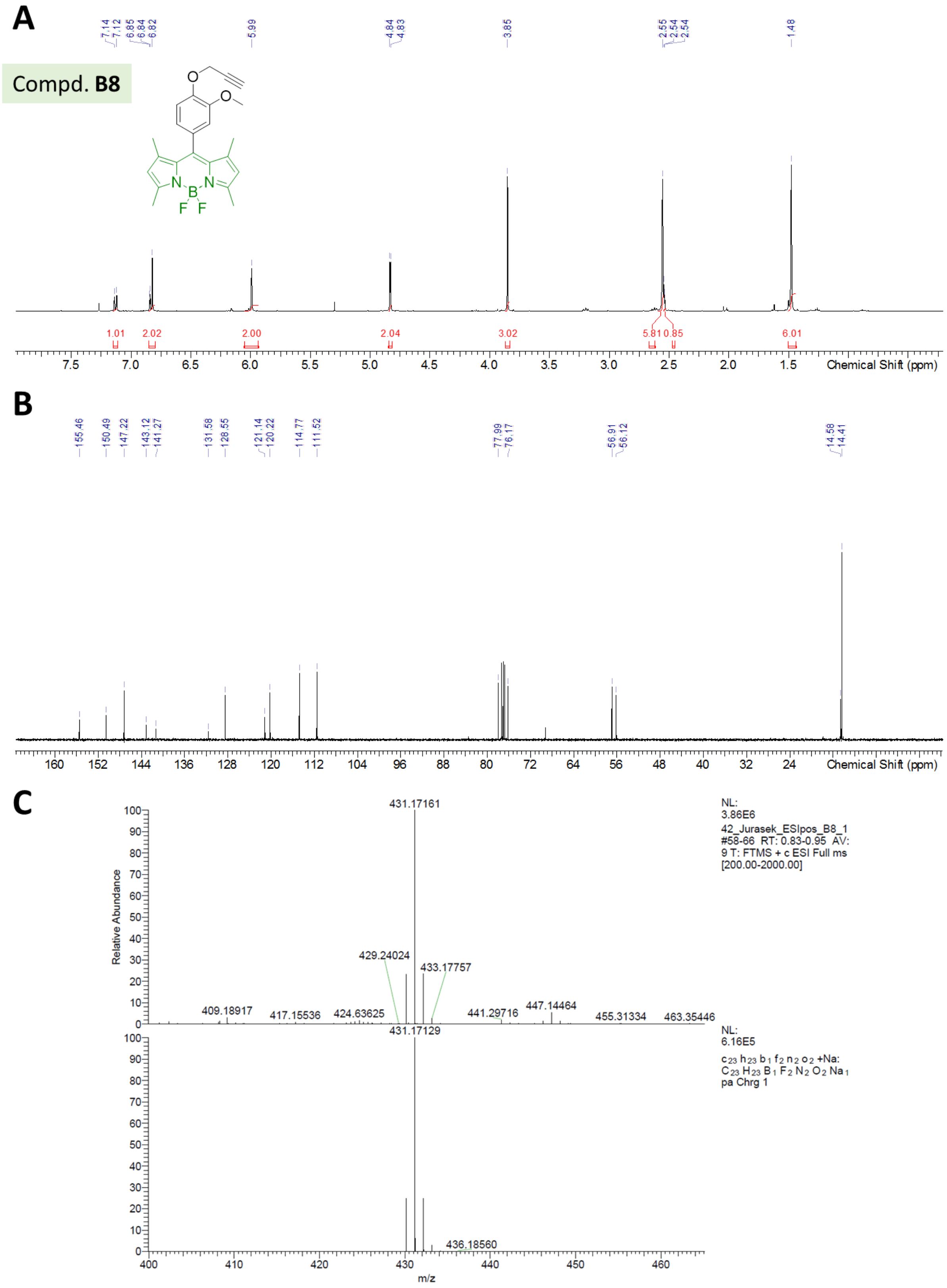

Fig. S5. ^{1}H NMR (A), ^{13}C NMR (B) and HRMS (C) of BODIPY **B8**

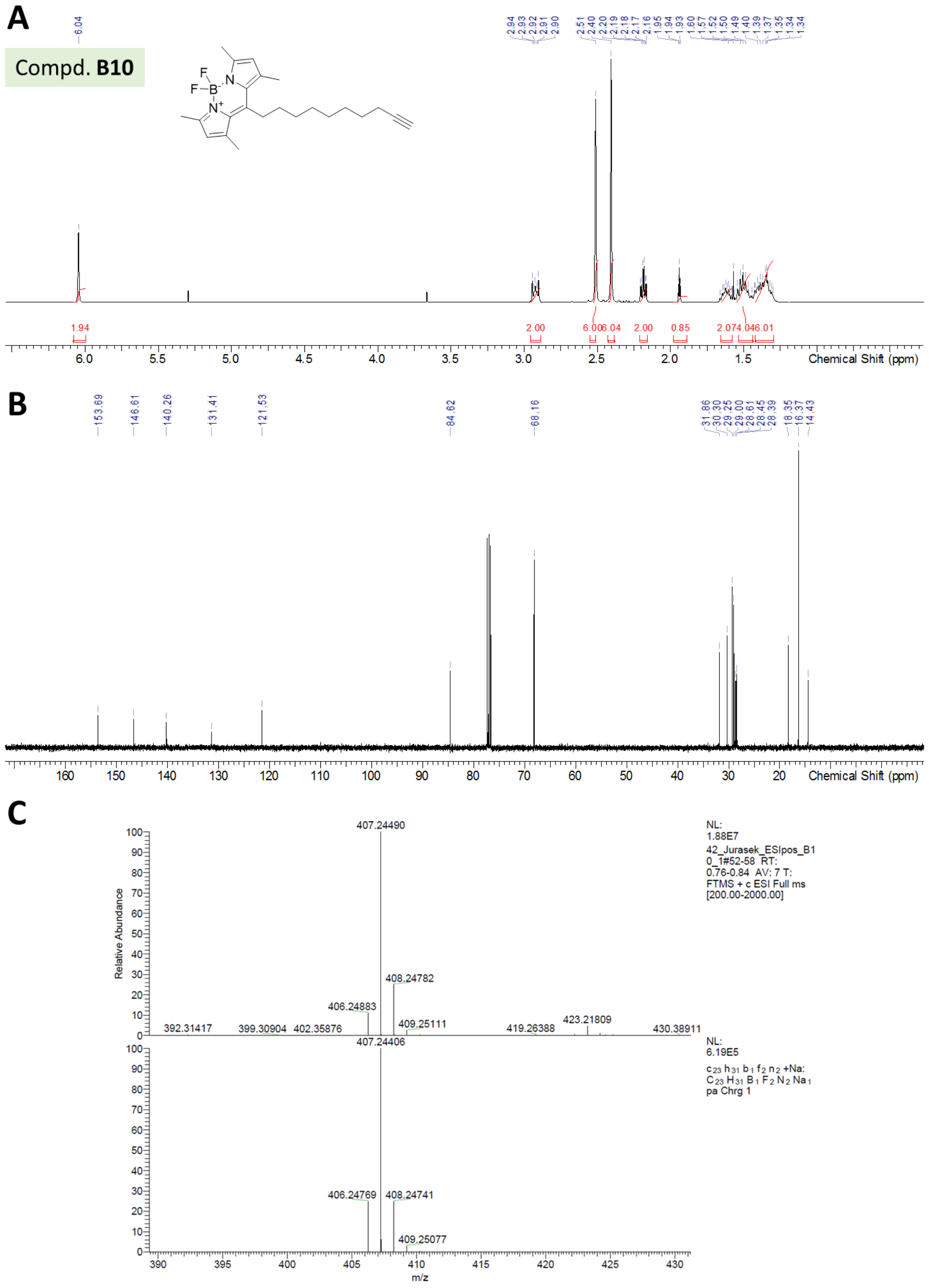

Fig. S6. ^{1}H NMR (A), ^{13}C NMR (B) and HRMS (C) of BODIPY **B10**

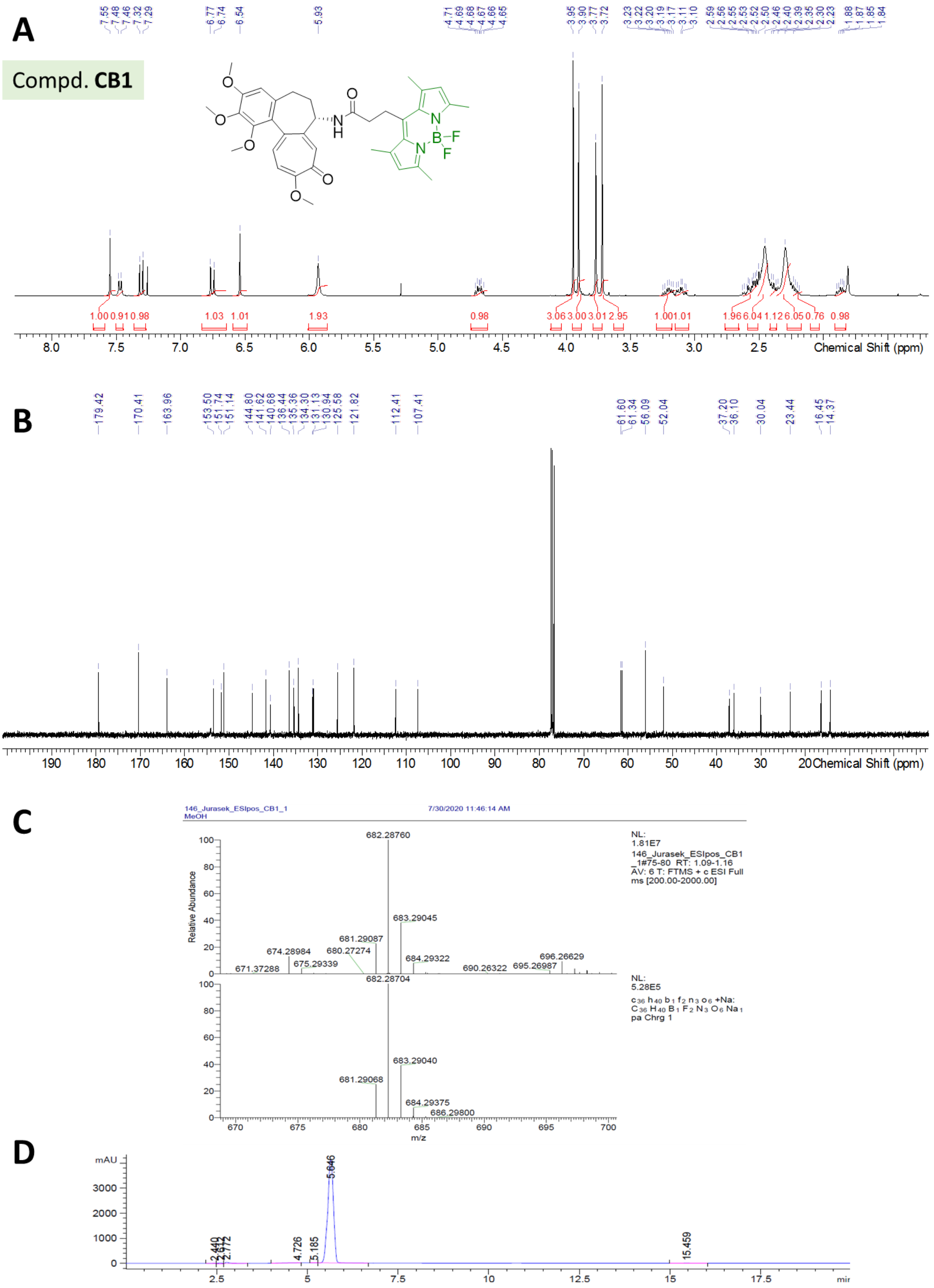

Fig. S7. ^{1}H NMR (A), ^{13}C NMR (B), HRMS (C) spectra and HPLC chromatogram (D) of **CB1**

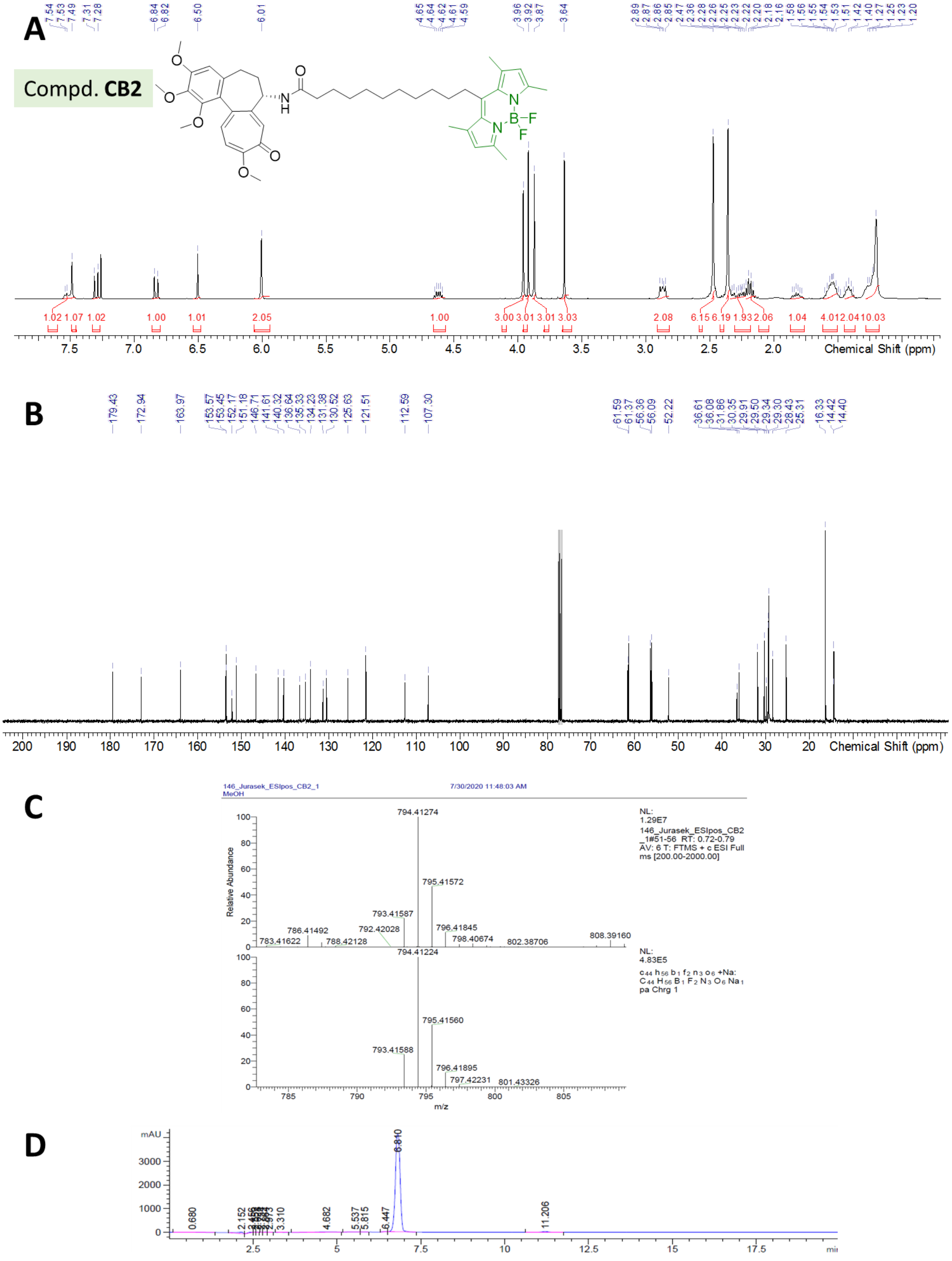

Fig. S8. ^{1}H NMR (A), ^{13}C NMR (B), HRMS (C) spectra and HPLC chromatogram (D) of **CB2**

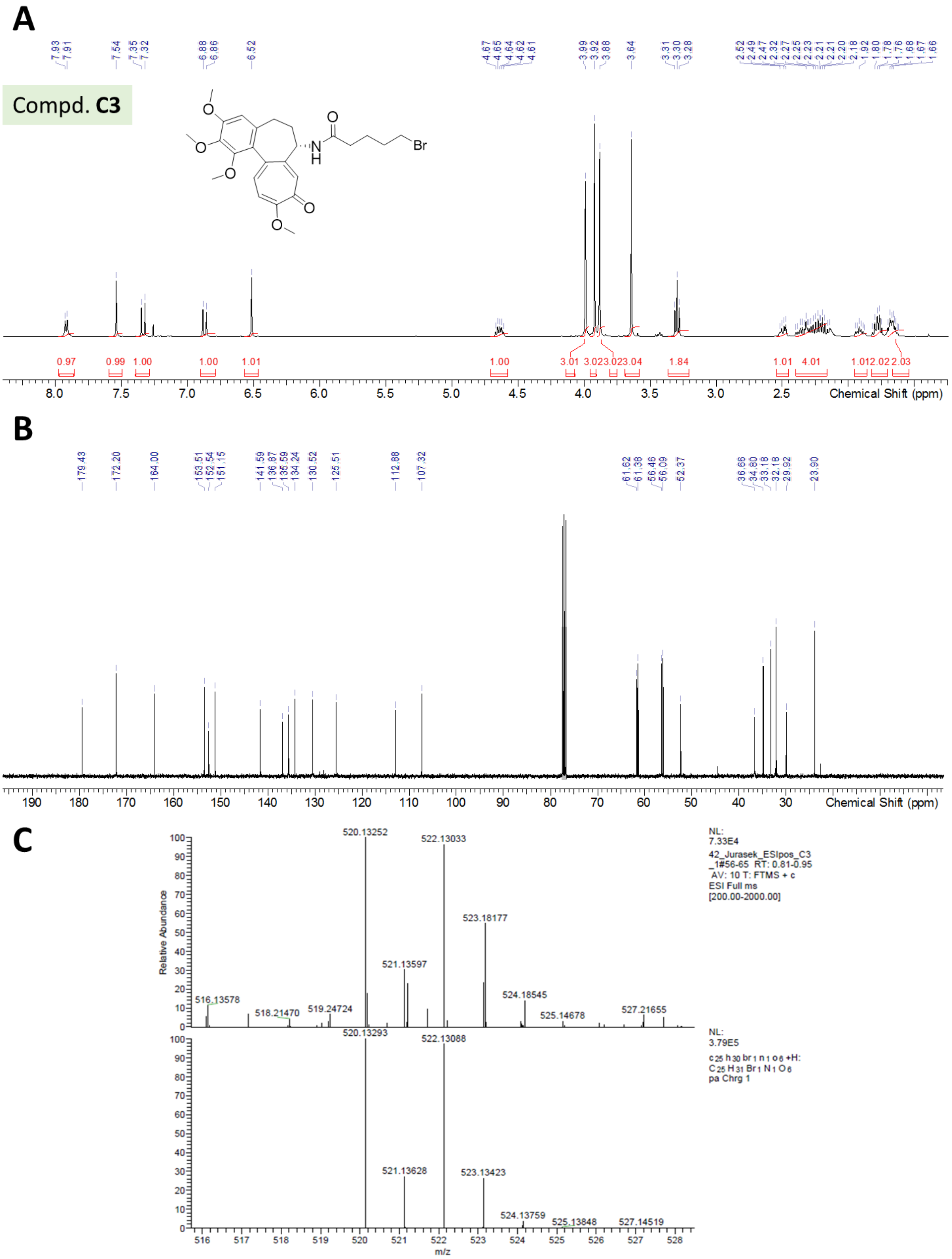

Fig. S9. ^{1}H NMR (A), ^{13}C NMR (B) and HRMS (C) of intermediate **C3**

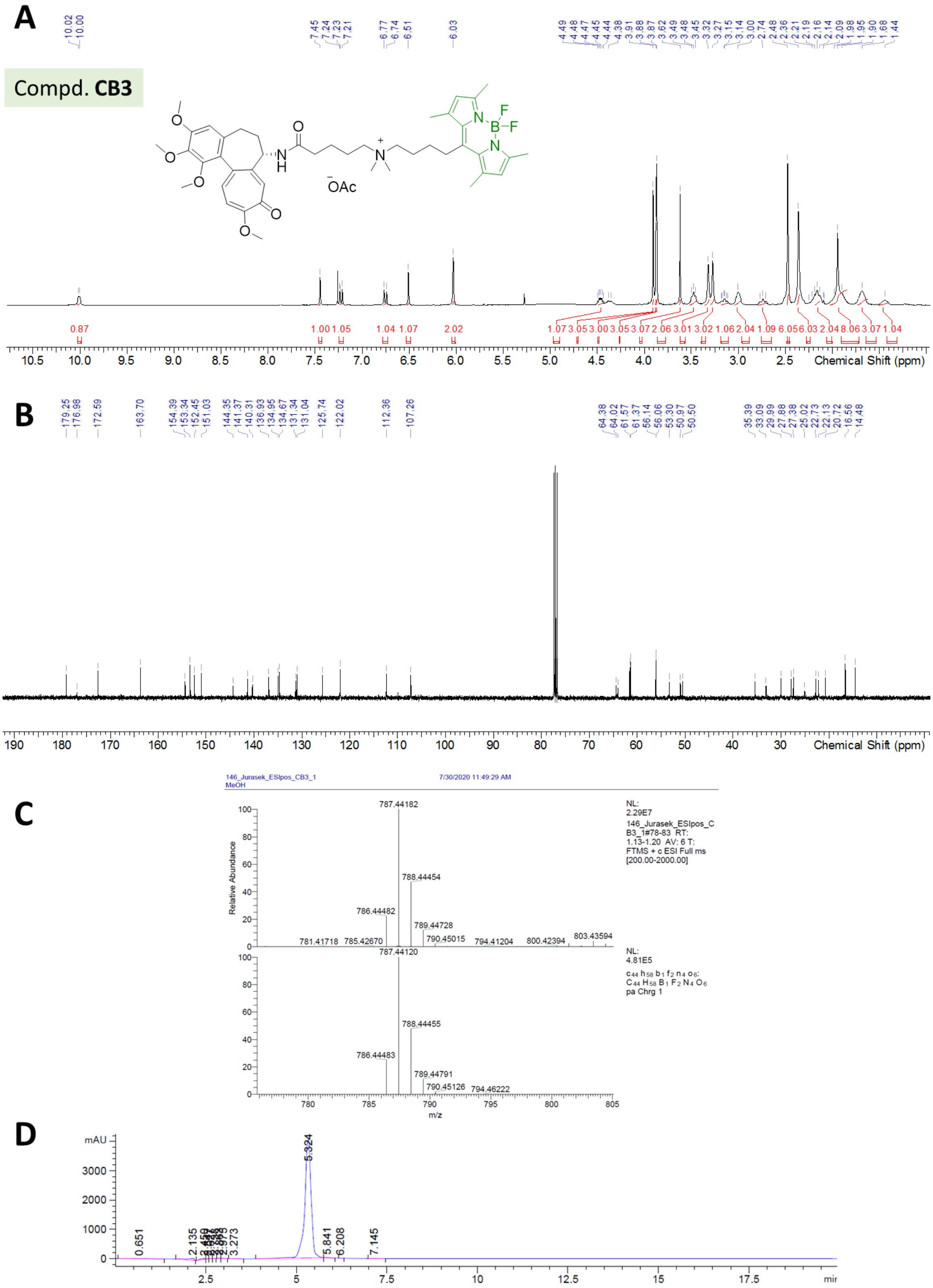

Fig. S10. ^{1}H NMR (A), ^{13}C NMR (B), HRMS (C) spectra and HPLC chromatogram (D) of **CB3**

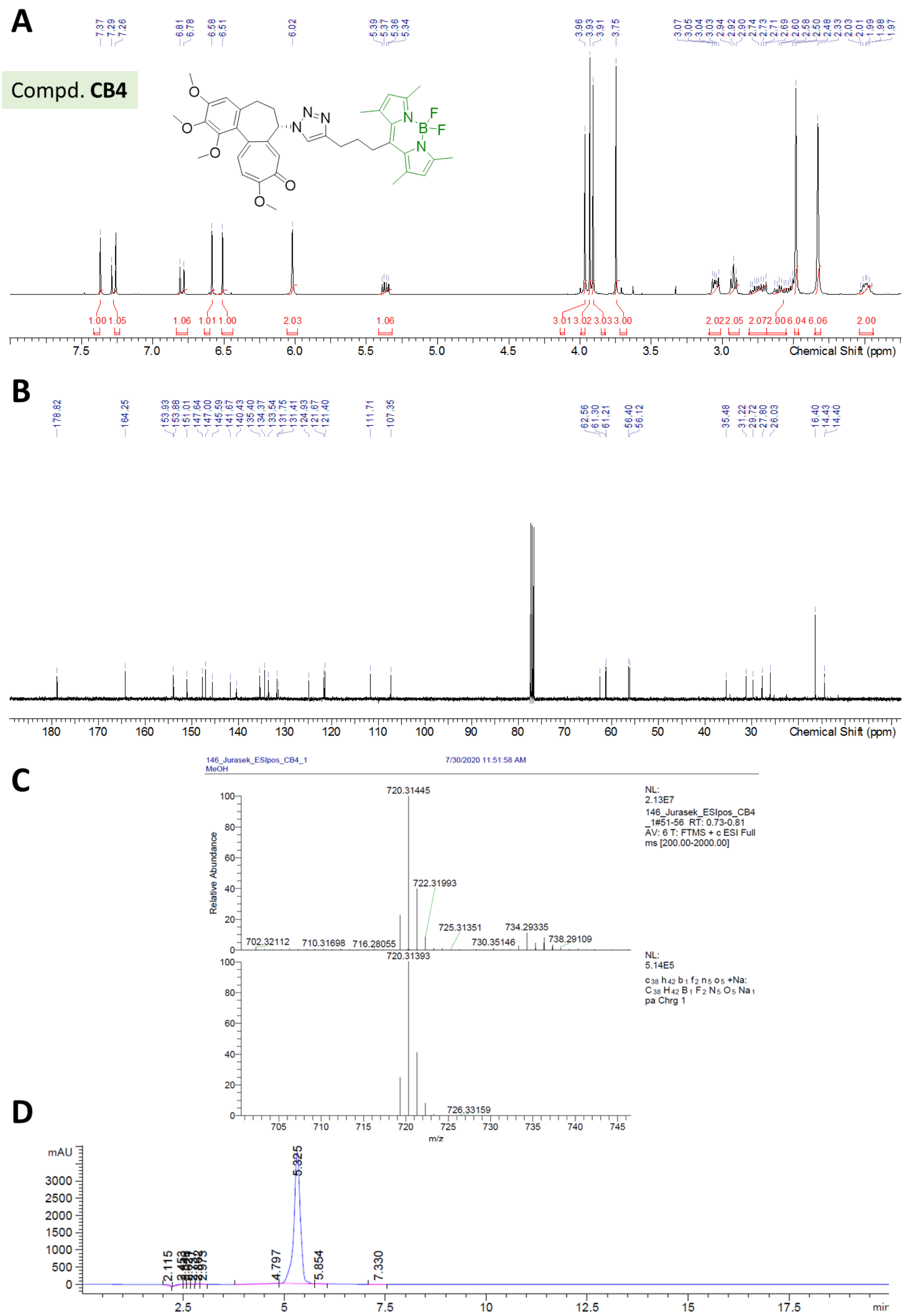

Fig. S11. ^{1}H NMR (A), ^{13}C NMR (B), HRMS (C) spectra and HPLC chromatogram (D) of **CB4**

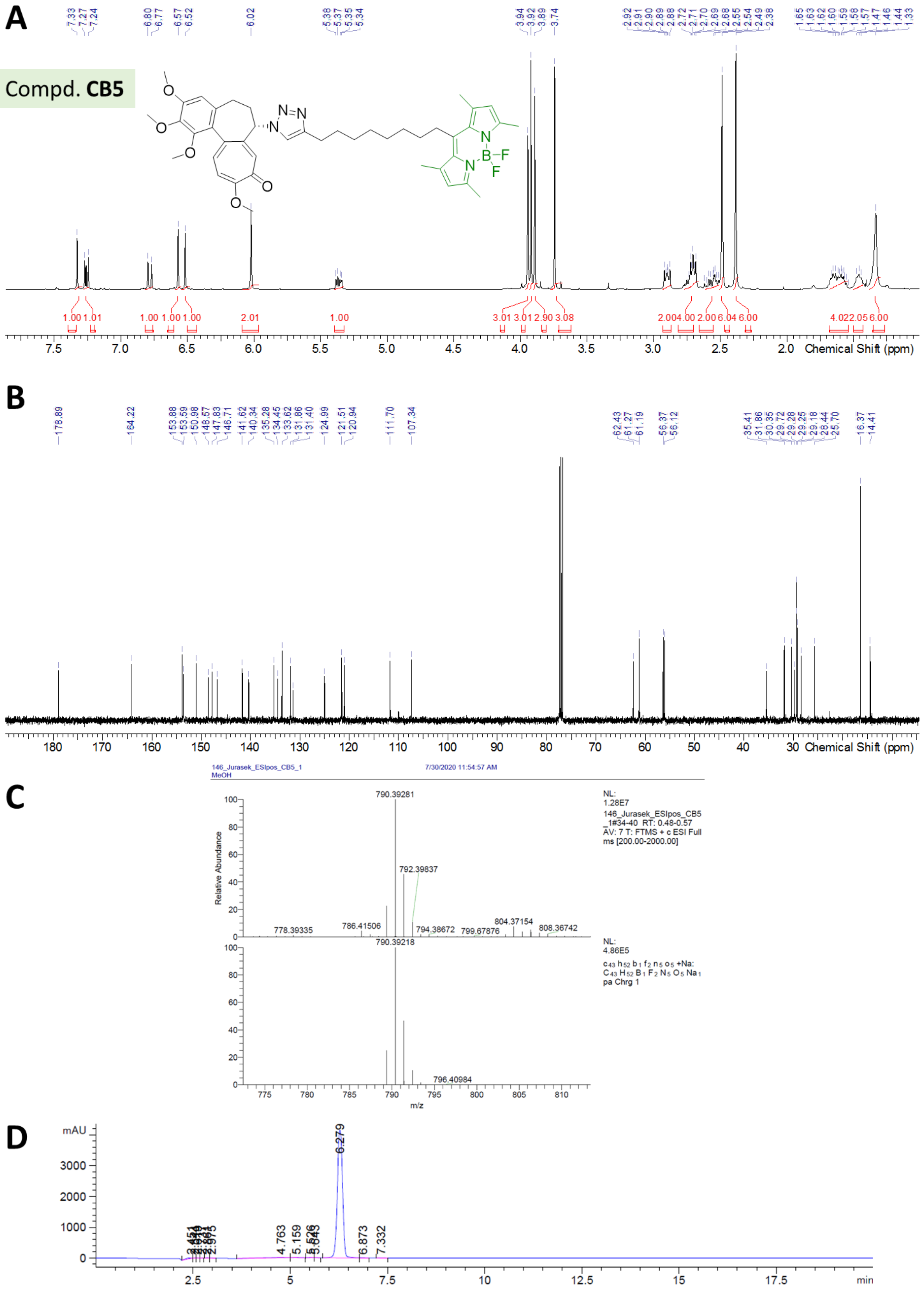

Fig. S12. ^{1}H NMR (A), ^{13}C NMR (B), HRMS (C) spectra and HPLC chromatogram (D) of **CB5**

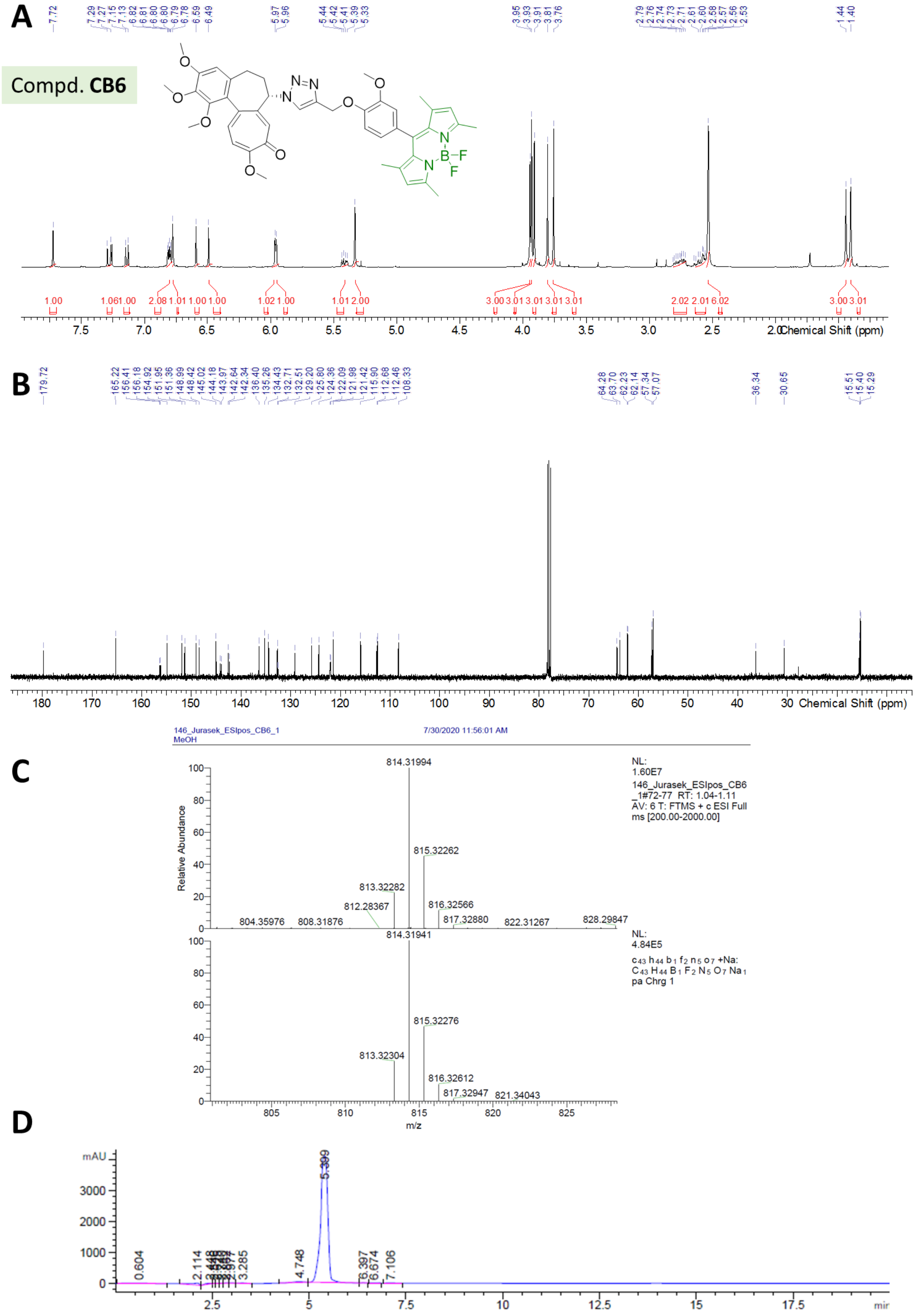

Fig. S13. ^{1}H NMR (A), ^{13}C NMR (B), HRMS (C) spectra and HPLC chromatogram (D) of **CB6**

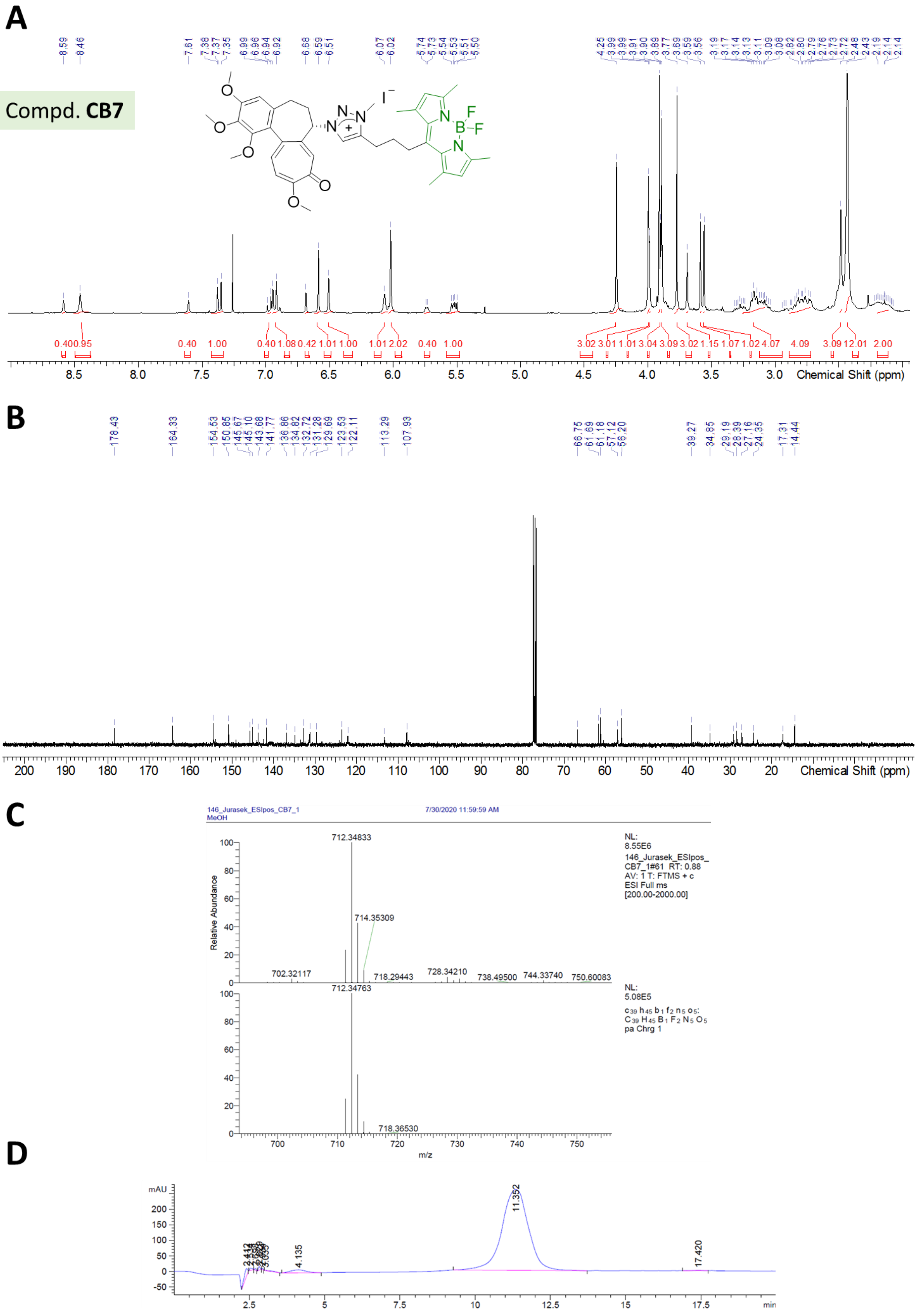

Fig. S14. ^{1}H NMR (A), ^{13}C NMR (B), HRMS (C) spectra and HPLC chromatogram (D) of **CB7**

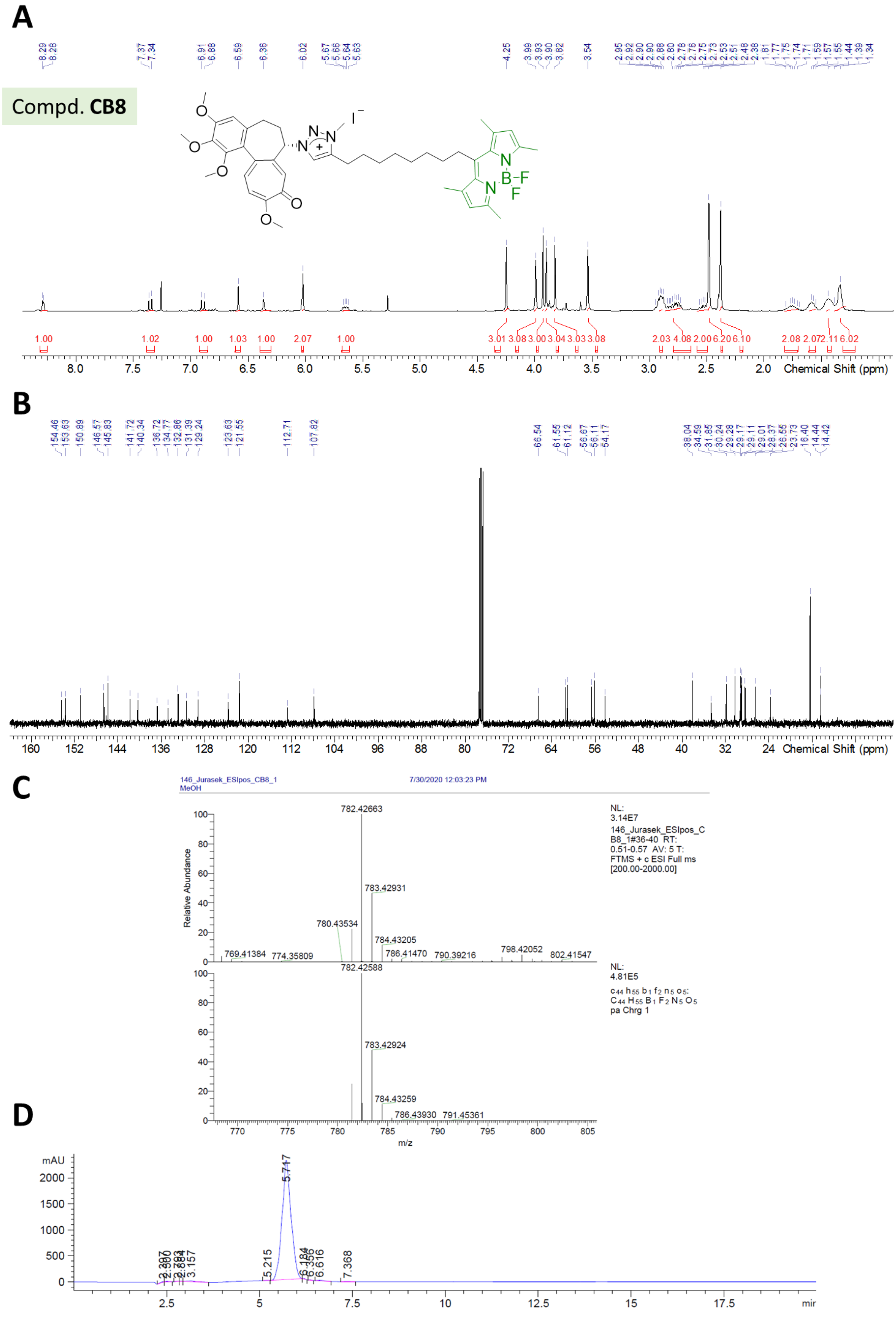

Fig. S15. ^{1}H NMR (A), ^{13}C NMR (B), HRMS (C) spectra and HPLC chromatogram (D) of **CB8**

A

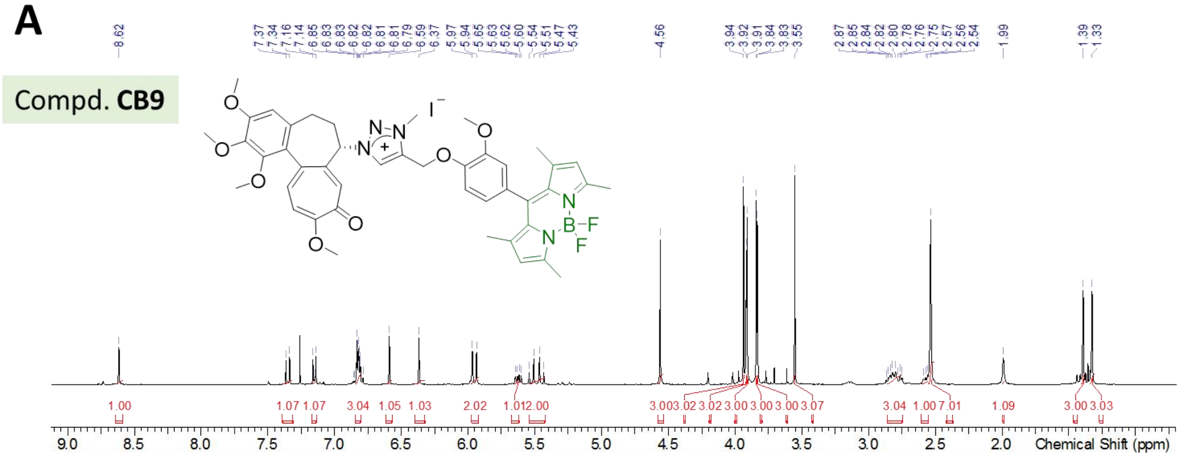

B

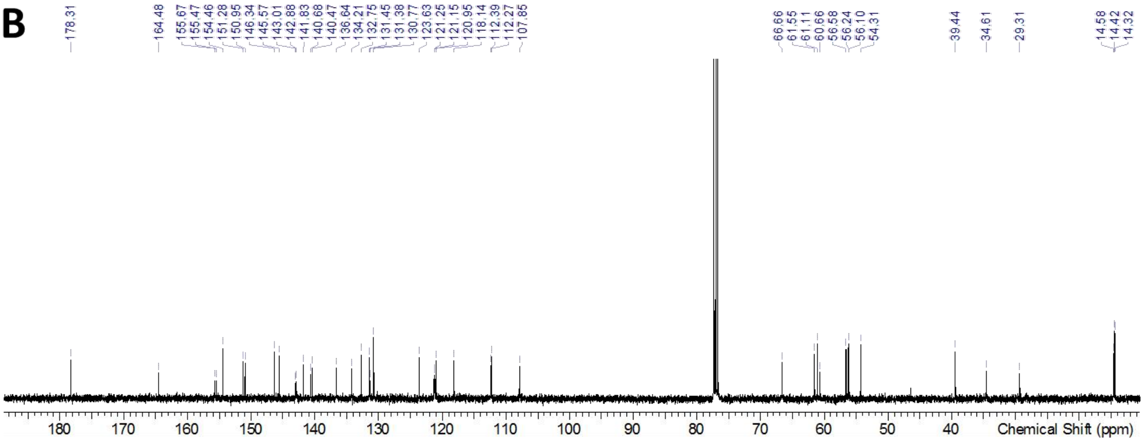

C

D

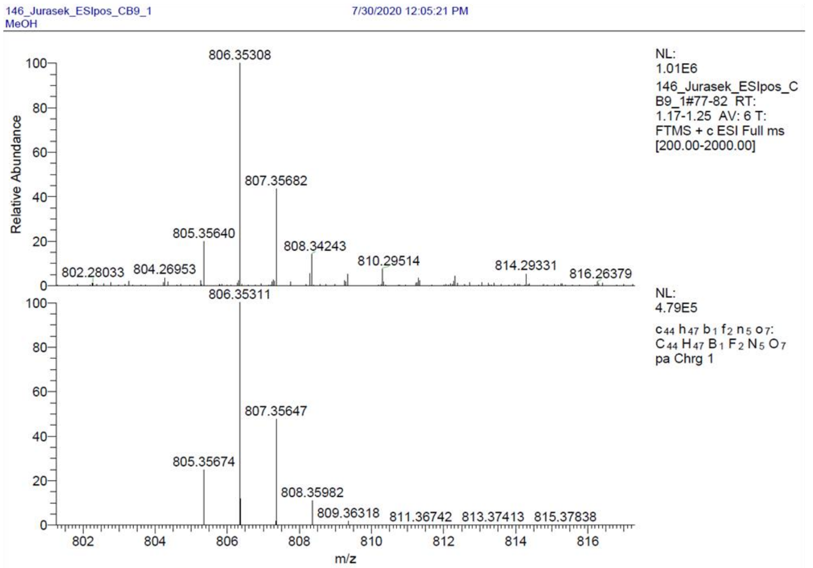

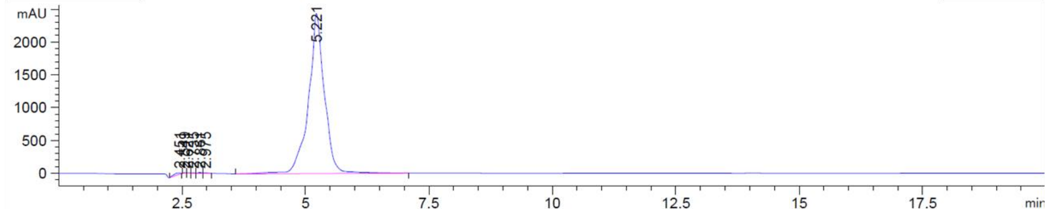

Fig. S16. ^{1}H NMR (A), ^{13}C NMR (B), HRMS (C) spectra and HPLC chromatogram (D) of **CB9**

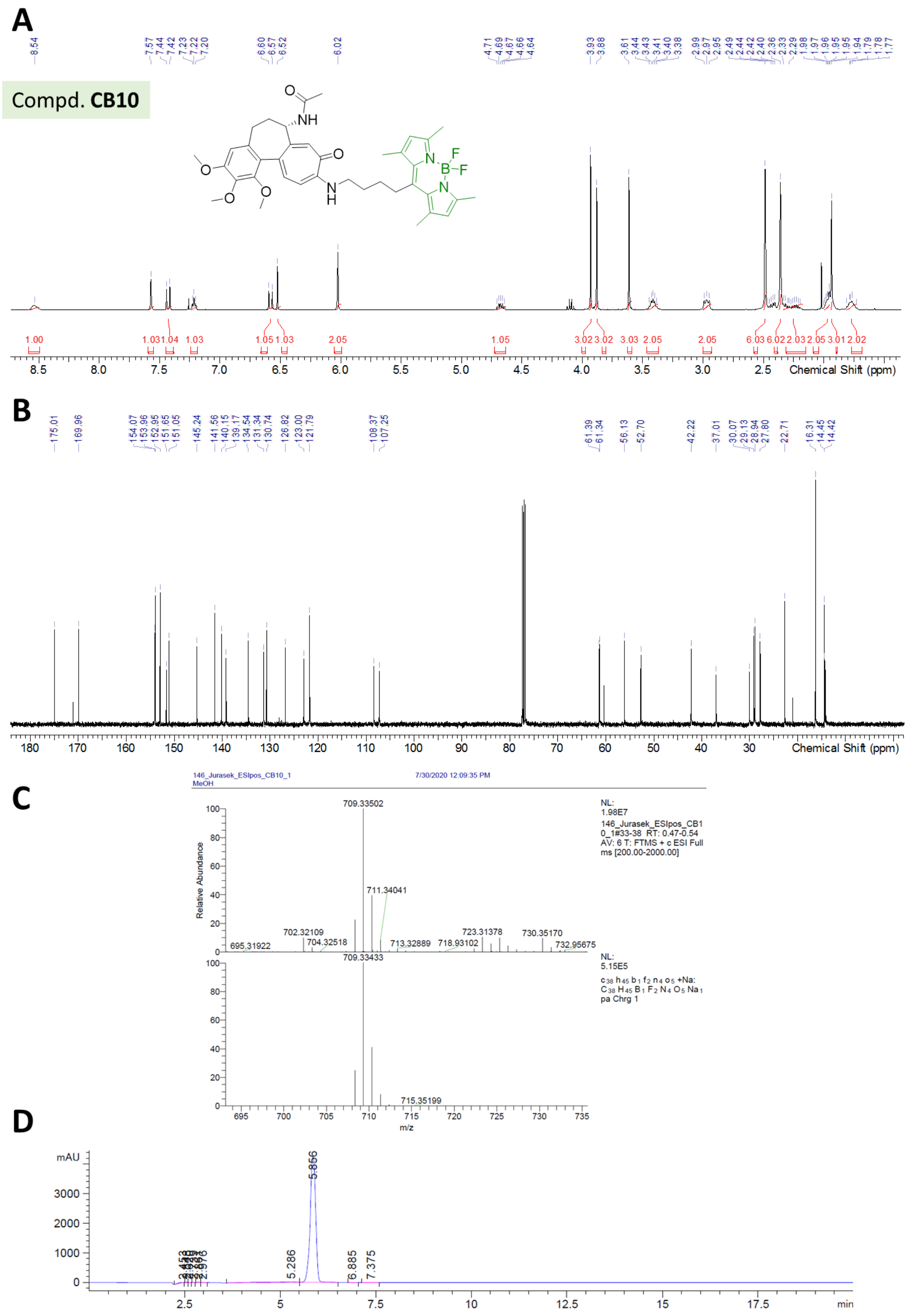

Fig. S17. ^{1}H NMR (A), ^{13}C NMR (B), HRMS (C) spectra and HPLC chromatogram (D) of **CB10**

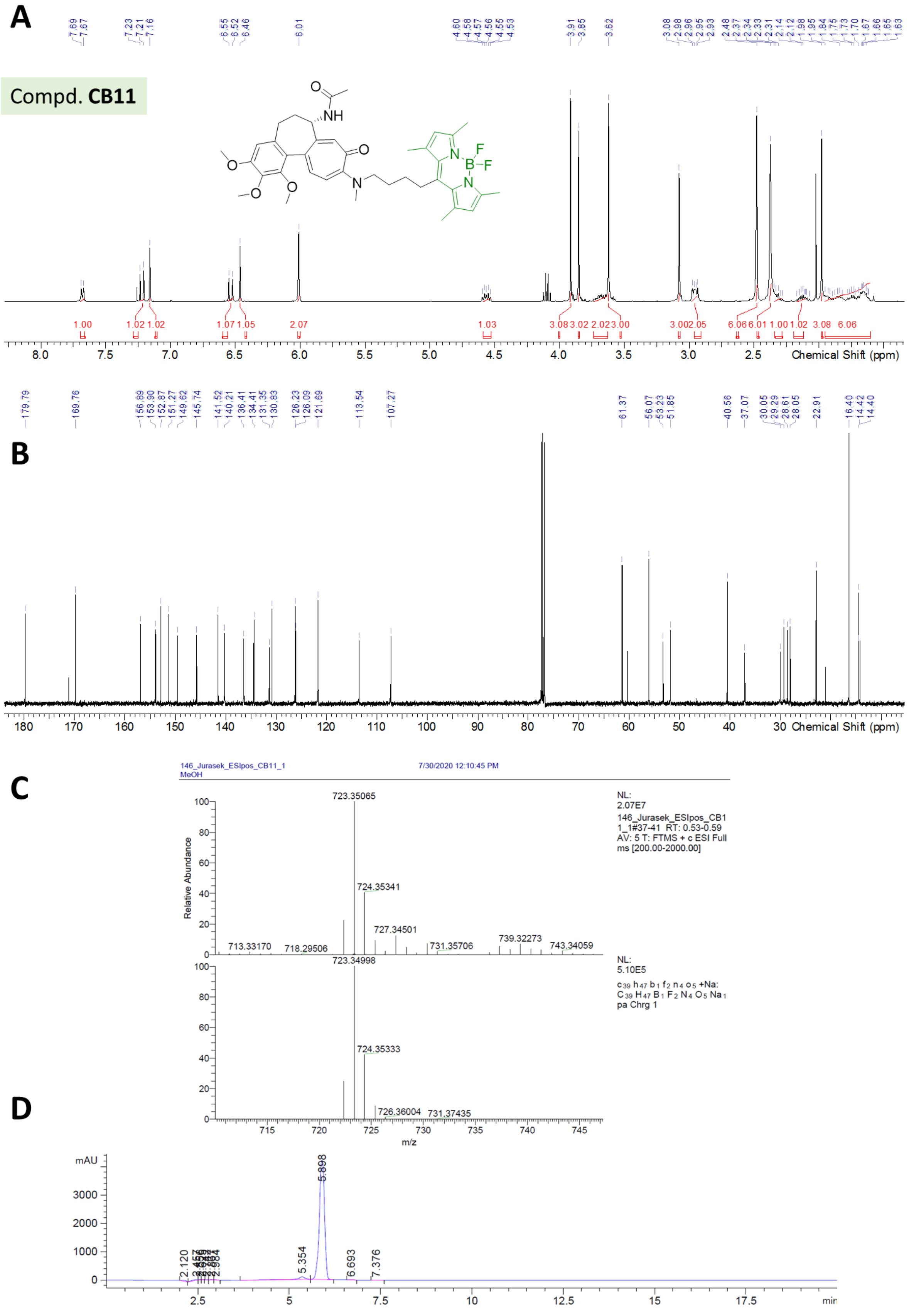

Fig. S18. ^{1}H NMR (A), ^{13}C NMR (B), HRMS (C) spectra and HPLC chromatogram (D) of **CB11**

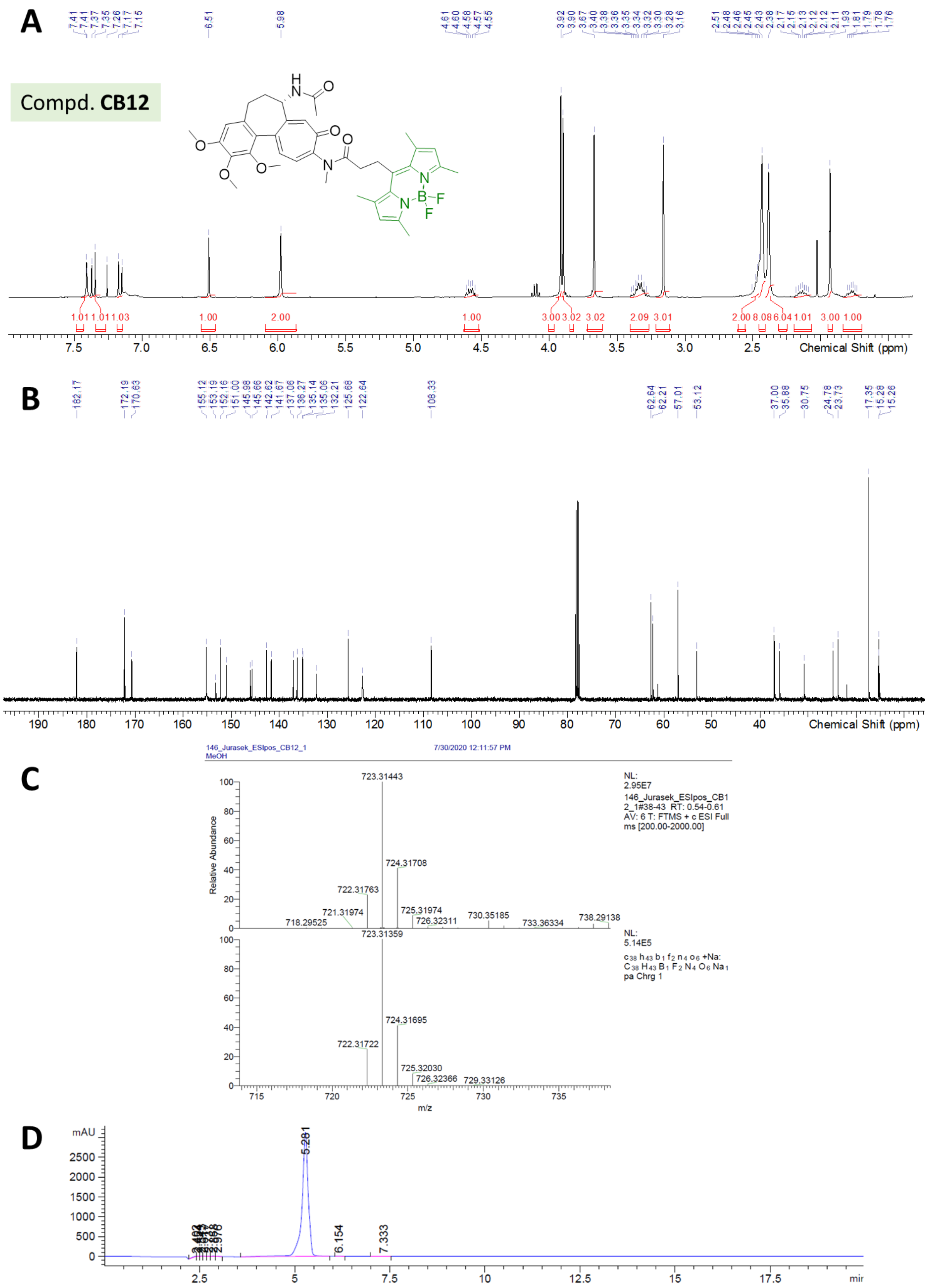

Fig. S19. ^{1}H NMR (A), ^{13}C NMR (B), HRMS (C) spectra and HPLC chromatogram (D) of **CB11**

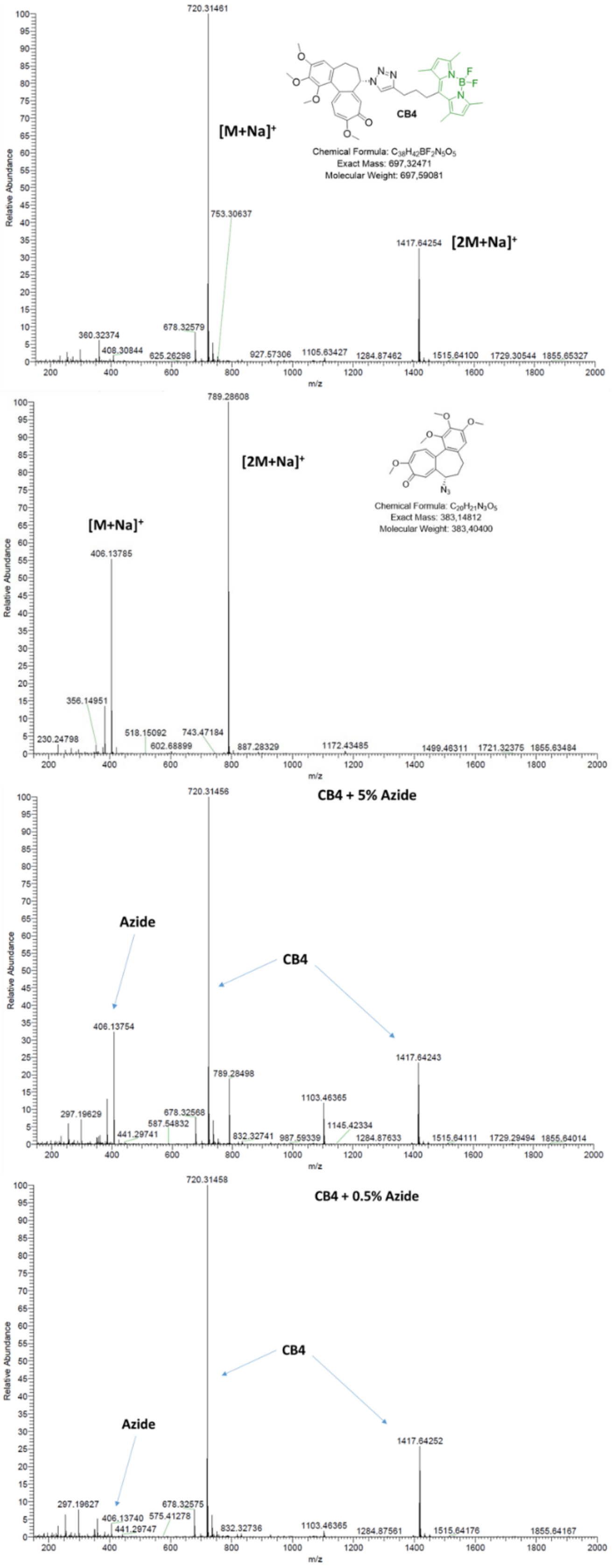

Fig. S20. HRMS spectra of the spiked **CB4** with azide **C3**

Tab. S2. Calculated properties of CBs

Compd.	**Col**	**CB1**	**CB2**	**CB3**	**CB4**	**CB5**	**CB6**
Lipinski	good	moderate	bad	moderate	bad	bad	bad
LogP*	1.14	4.52	7.40	1.47	5.55	7.15	5.41
Compd.	**CB7**	**CB8**	**CB9**	**CB10**	**CB11**	**CB12**	
Lipinski	moderate	moderate	bad	bad	bad	moderate	
LogP	1.28	3.02	1.73	5.18	5.56	4.31	

*LogP is calculated for colchicine derivatives or parent cations, not for the salt, if applicable. LogP and Lipinski's rule was calculated by software ACD/Percepta 14.54.0 (Build 3666)

Absorption and emission spectra

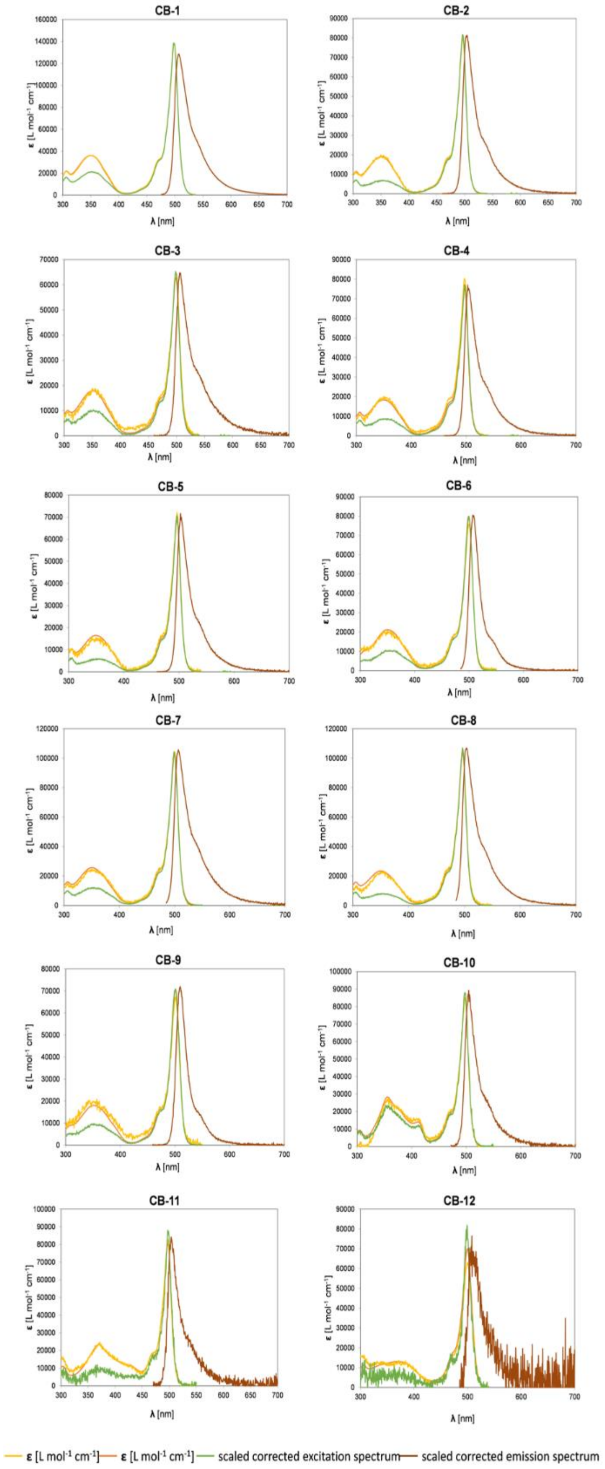

Fig. S21. Absorption, excitation and emission spectra of CB1 – CB12.

2. Effects of compounds on cellular activities and tubulin polymerization

Colchicine (Col)

CCRF-CEM (IC_{50} = 0.011 ± 0.00031 μM)

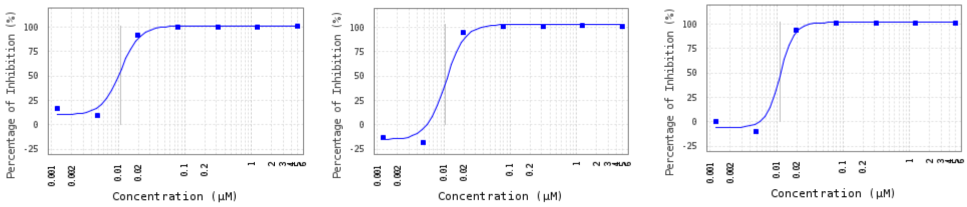

IC50: 0.0110 *(0.0088 - 0.0137)* pass
slope=3.000 min=10.198 max=101.284 err=129.504 R^2=0.9877

IC50: 0.0106 *(0.0082 - 0.0137)* pass
slope=3.000 min=-15.000 max=103.095 err=263.306 R^2=0.9863

IC50: 0.0112 *(0.0093 - 0.0136)* pass
slope=4.000 min=-6.014 max=101.640 err=106.531 R^2=0.9932

CEM-DNR (IC_{50} = 1.05 ± 0.12 μM)

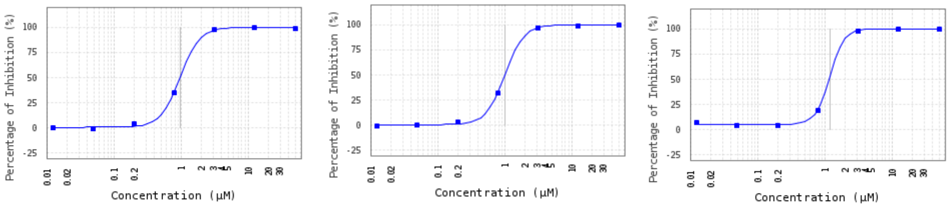

IC50: 0.9679 *(0.9103 - 1.0291)* pass
slope=3.000 min=0.791 max=100.000 err=10.503 R^2=0.9993

IC50: 1.0064 *(0.9485 - 1.0677)* pass
slope=3.091 min=0.666 max=100.000 err=8.088 R^2=0.9994

IC50: 1.1868 *(1.0779 - 1.3067)* pass
slope=4.187 min=5.420 max=100.000 err=5.477 R^2=0.9996

K562 (IC_{50} = 0.014 ± 0.001 μM)

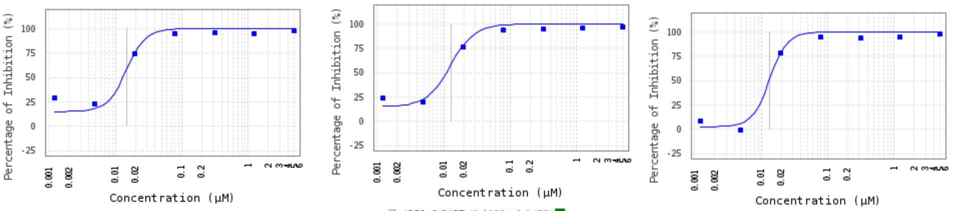

IC50: 0.0147 *(0.0117 - 0.0185)* pass
slope=3.000 min=15.000 max=100.000 err=284.380 R^2=0.9566

IC50: 0.0127 *(0.0092 - 0.0175)* pass
slope=2.416 min=15.000 max=100.000 err=166.178 R^2=0.9769

IC50: 0.0134 *(0.0113 - 0.0160)* pass
slope=3.300 min=2.599 max=100.000 err=178.020 R^2=0.9842

K562-TAX (IC_{50} = 1.80 ± 0.062 μM)

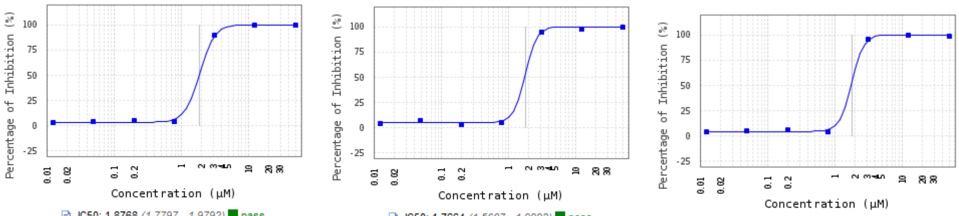

IC50: 1.8768 *(1.7797 - 1.9792)* pass
slope=4.000 min=3.636 max=100.000 err=7.483 R^2=0.9995

IC50: 1.7664 *(1.5607 - 1.9992)* pass
slope=5.000 min=4.889 max=100.000 err=15.870 R^2=0.9989

IC50: 1.7714 *(1.6353 - 1.9188)* pass
slope=5.000 min=4.599 max=100.000 err=6.794 R^2=0.9995

HCT116 (IC_{50} = 0.024 ± 0.002 μM)

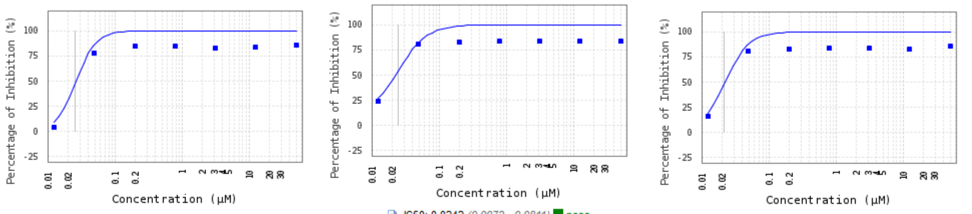

IC50: 0.0253 *(1.52643E-10 - 4.1936E6)* pass
slope=2.784 min=-3.136 max=100.000 err=1298.814 R^2=0.7602

IC50: 0.0242 *(0.0072 - 0.0811)* pass
slope=2.000 min=7.917 max=100.000 err=1307.853 R^2=0.5548

IC50: 0.0215 *(8.80304E-12 - 5.23628E7)* pass
slope=2.377 min=-1.850 max=100.000 err=1355.857 R^2=0.6544

HCT116p53-/- (IC_{50} = 0.027 ± 0.006 µM)

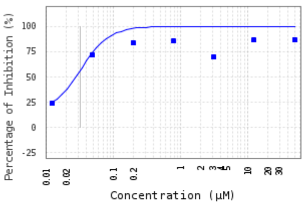

IC50: 0.0324 (0.0120 - 0.0877) pass
slope=2.000 min=14.357 max=100.000 err=1627.756 R^2=0.4684

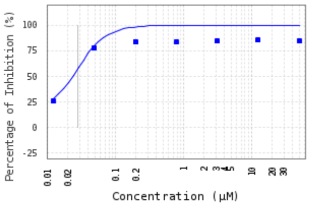

IC50: 0.0276 (0.0102 - 0.0748) pass
slope=2.000 min=14.169 max=100.000 err=1136.606 R^2=0.5991

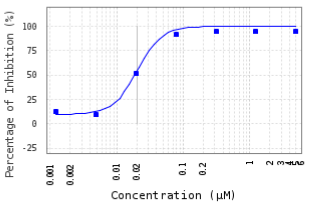

IC50: 0.0205 (0.0174 - 0.0242) pass
slope=2.402 min=9.705 max=100.000 err=126.089 R^2=0.9866

U2OS (IC_{50} = 0.022 ± 0.0004 µM)

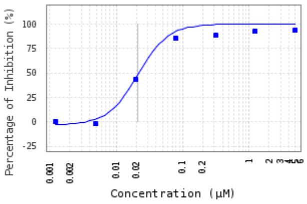

IC50: 0.0212 (0.0160 - 0.0280) pass
slope=1.918 min=-3.235 max=100.000 err=288.178 R^2=0.9743

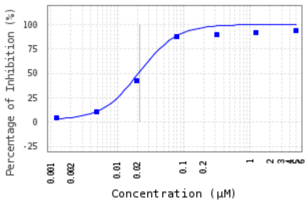

IC50: 0.0217 (0.0159 - 0.0296) pass
slope=1.562 min=2.312 max=100.000 err=201.308 R^2=0.9792

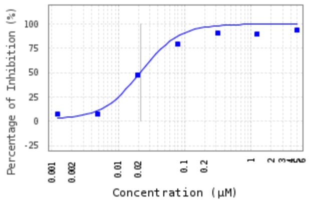

IC50: 0.0220 (0.0151 - 0.0320) pass
slope=1.500 min=1.885 max=100.000 err=273.201 R^2=0.9698

MRC5 (IC_{50} = 0.040 ± 0.008 µM)

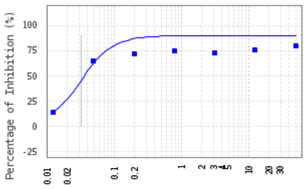

IC50: 0.0318 (0.0143 - 0.0707) pass
slope=1.800 min=0.664 max=90.000 err=1070.237 R^2=0.6603

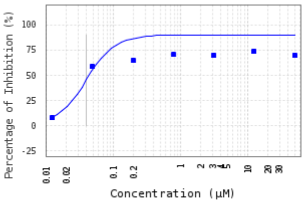

IC50: 0.0396 (0.0176 - 0.0888) pass
slope=2.000 min=0.882 max=90.000 err=1905.691 R^2=0.4117

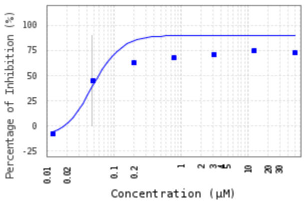

IC50: 0.0475 (0.0244 - 0.0923) pass
slope=2.000 min=-12.447 max=90.000 err=1874.878 R^2=0.6375

BJ (IC_{50} > 50 µM)

Primary screen (50 µM)

1 (Plate 1)	L21	LEM00013030	42.521		50.0uM	+	✓ ⊗
1 (Plate 1)	L22	LEM00013030	39.769		50.0uM	+	
1 (Plate 1)	L23	LEM00013030	36		50.0uM	+	

1 (Plate 1)	L21	LEM00013030	36.15		50.0uM	+	✓ ⊗
1 (Plate 1)	L22	LEM00013030	39.388		50.0uM	+	
1 (Plate 1)	L23	LEM00013030	37.769		50.0uM	+	

B1

CCRF-CEM (IC50 > 50 µM)

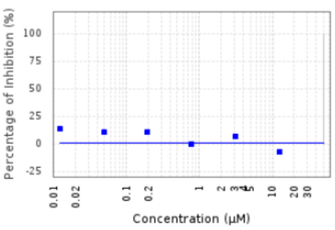

IC50: >50 pass
slope=10.529 min=0.090 max=100.000 err=1640.113 R^2=-0.0000

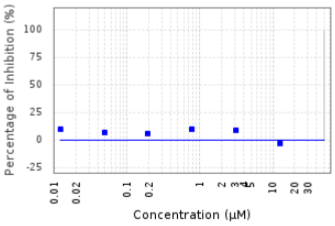

IC50: >50 pass
slope=8.123 min=-0.391 max=100.000 err=1803.983 R^2=-0.0000

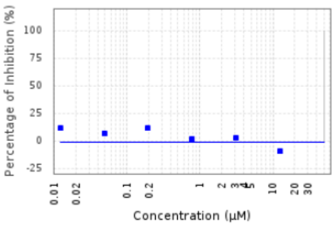

IC50: >50 pass
slope=9.835 min=-1.664 max=100.000 err=1596.145 R^2=-0.0000

K562 (IC50 > 50 μM)

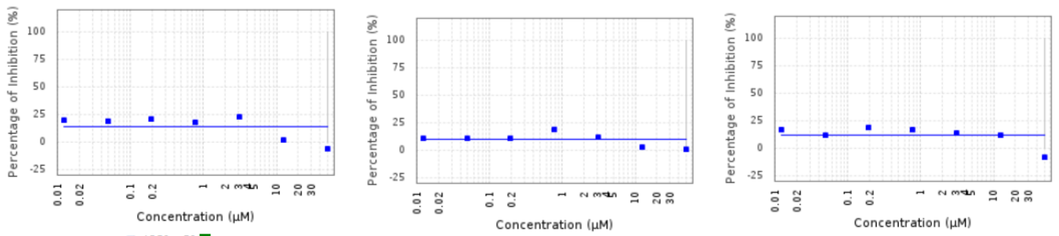

IC50: >50 pass
slope=15.204 min=13.109 max=100.000 err=745.937 R^2=0.0000

IC50: >50 pass
slope=9.565 min=9.185 max=100.000 err=220.945 R^2=-0.0000

IC50: >50 pass
slope=5.200 min=11.311 max=100.000 err=510.937 R^2=-0.0000

A549 (IC50 > 50 μM)

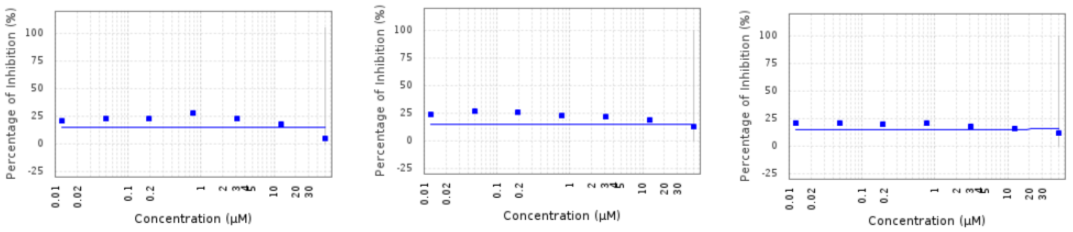

IC50: >50 pass
slope=13.979 min=15.000 max=104.736 err=469.690 R^2=-0.4648

IC50: >50 pass
slope=7.734 min=15.000 max=100.000 err=440.266 R^2=-2.0745

IC50: >50 pass
slope=5.316 min=15.000 max=100.000 err=138.692 R^2=-0.7546

U2OS (IC50 > 50 μM)

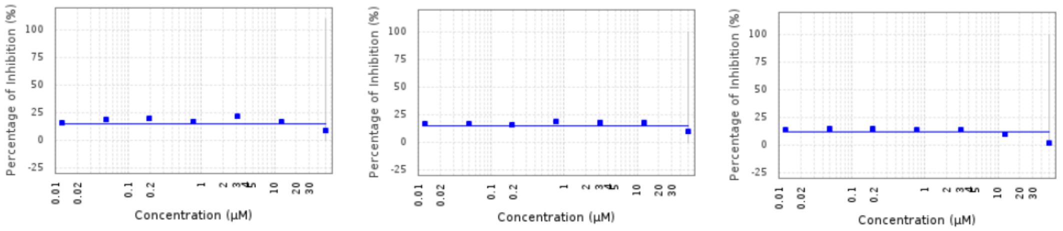

IC50: >50 pass
slope=2.650 min=15.000 max=110.000 err=116.170 R^2=-0.1758

IC50: >50 pass
slope=13.012 min=15.000 max=100.000 err=59.414 R^2=-0.1159

IC50: >50 pass
slope=26.644 min=11.323 max=100.000 err=125.219 R^2=0.0000

HCT116 (IC50 > 50 μM)

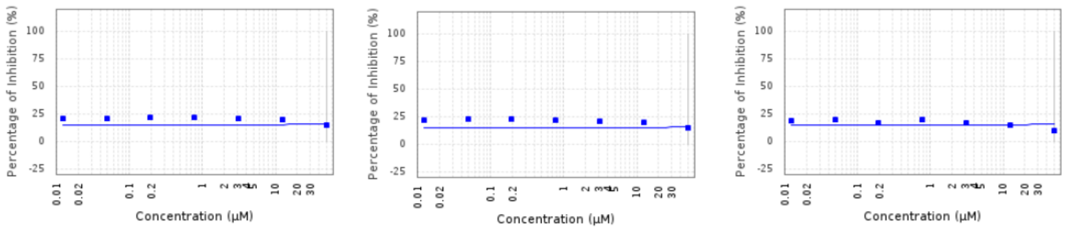

IC50: >50 pass
slope=3.158 min=15.000 max=100.000 err=191.250 R^2=-4.8913

IC50: >50 pass
slope=8.821 min=15.000 max=100.000 err=228.220 R^2=-4.7164

IC50: >50 pass
slope=5.579 min=15.000 max=100.000 err=91.197 R^2=-0.1345

HCT116p53-/- (IC50 > 50 μM)

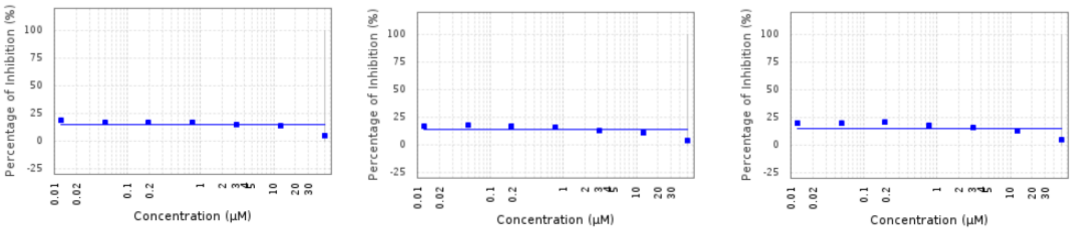

IC50: >50 pass
slope=10.714 min=14.257 max=100.000 err=119.557 R^2=-0.0000

IC50: >50 pass
slope=10.299 min=13.258 max=100.000 err=139.669 R^2=-0.0000

IC50: >50 pass
slope=8.259 min=15.000 max=100.000 err=205.416 R^2=-0.0112

MRC5 (IC50 > 50 µM)

Primary screen (50 µM)

1 (Plate 1)	K3	LEM00230985	-11.655		50.0uM
1 (Plate 1)	K4	LEM00230985	-6.8657		50.0uM
1 (Plate 1)	K5	LEM00230985	-8.0631		50.0uM

1 (Plate 1)	K3	LEM00230985	-11.04		50.0uM
1 (Plate 1)	K4	LEM00230985	-7.0989		50.0uM
1 (Plate 1)	K5	LEM00230985	15.234		50.0uM

BJ (IC50 > 50 µM)

Primary screen (50 µM)

1 (Plate 1)	K3	LEM00230985	0.7197		50.0uM
1 (Plate 1)	K4	LEM00230985	5.8129		50.0uM
1 (Plate 1)	K5	LEM00230985	6.3665		50.0uM

1 (Plate 1)	K3	LEM00230985	-1.342		50.0uM
1 (Plate 1)	K4	LEM00230985	2.2974		50.0uM
1 (Plate 1)	K5	LEM00230985	5.3681		50.0uM

B2

CCRF-CEM (IC50 > 50 µM)

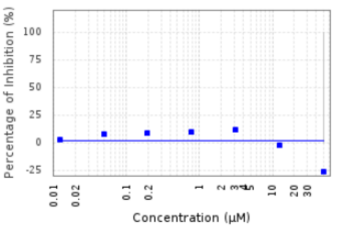

IC50: >50 pass
slope=8.212 min=1.474 max=100.000 err=1042.848 R^2=-0.0000

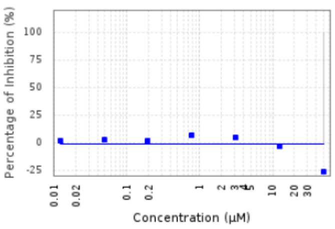

IC50: >50 pass
slope=6.196 min=-1.775 max=100.000 err=787.927 R^2=-0.0000

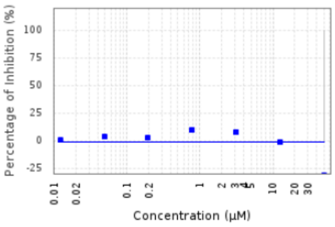

IC50: >50 pass
slope=7.438 min=-1.374 max=100.000 err=1158.908 R^2=-0.0000

K562 (IC50 > 50 µM)

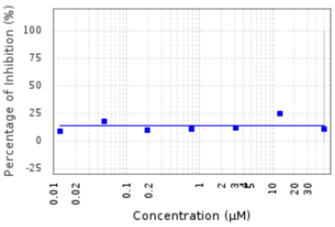

IC50: >50 pass
slope=11.488 min=13.175 max=100.000 err=197.750 R^2=0.0000

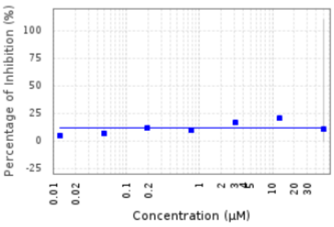

IC50: >50 pass
slope=16.570 min=11.374 max=110.000 err=163.598 R^2=0.0000

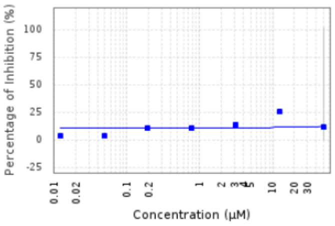

IC50: >50 pass
slope=1.500 min=10.905 max=101.057 err=324.133 R^2=0.0124

A549 (IC50 > 50 µM)

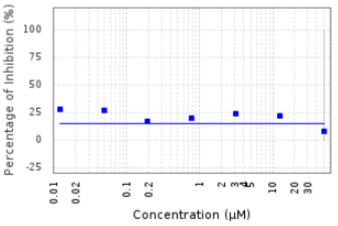

IC50: >50 pass
slope=5.385 min=15.000 max=100.000 err=479.047 R^2=-0.6863

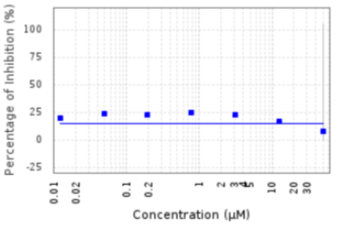

IC50: >50 pass
slope=14.255 min=15.000 max=104.454 err=354.683 R^2=-0.7009

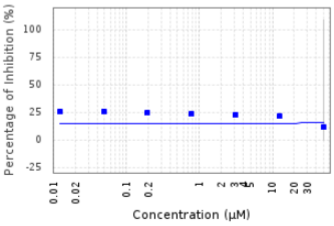

IC50: >50 pass
slope=6.043 min=15.000 max=108.177 err=507.125 R^2=-2.4183

U2OS (IC50 > 50 μM)

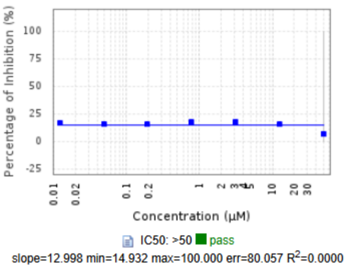

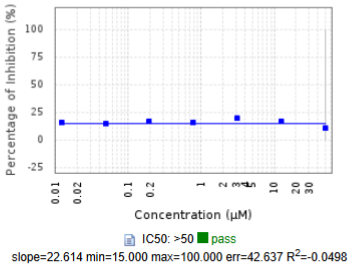

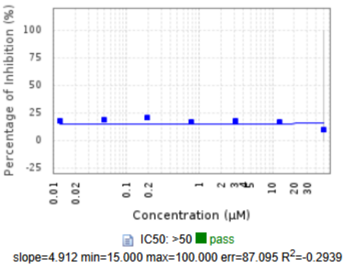

HCT116 (IC50 > 50 μM)

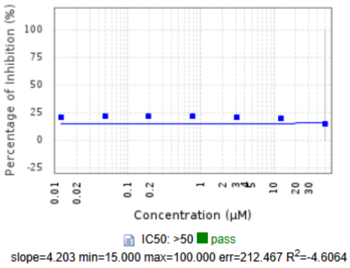

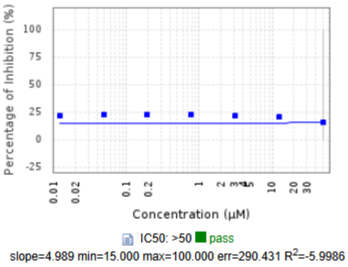

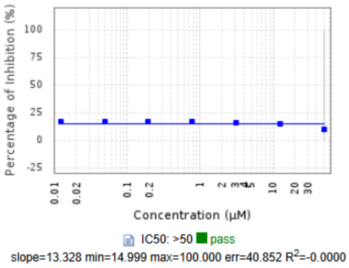

HCT116p53-/- (IC50 > 50 μM)

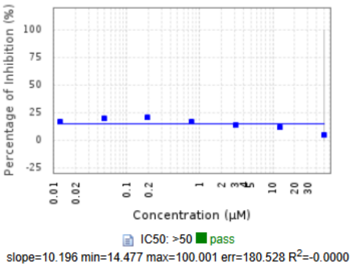

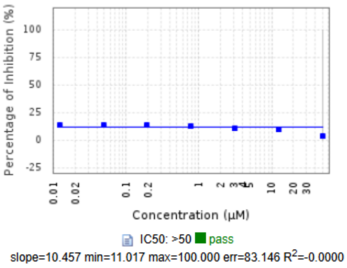

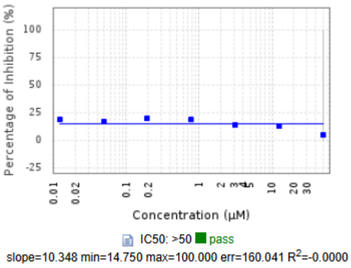

MRC5 (IC50 > 50 μM)

Primary screen (50 μM)

1 (Plate 1)	M3	LEM00230987	-7.8042		50.0uM
1 (Plate 1)	M4	LEM00230987	-3.2704		50.0uM
1 (Plate 1)	M5	LEM00230987	-1.9891		50.0uM

1 (Plate 1)	M3	LEM00230987	8.5331		50.0uM
1 (Plate 1)	M4	LEM00230987	-8.4146		50.0uM
1 (Plate 1)	M5	LEM00230987	-5.3332		50.0uM

BJ (IC50 > 50 μM)

Primary screen (50 μM)

1 (Plate 1)	M4	LEM00230987	-11.46		50.0uM
1 (Plate 1)	M5	LEM00230987	-8.4702		50.0uM
1 (Plate 1)	N3	LEM00230988	-6.8094		50.0uM

1 (Plate 1)	M3	LEM00230987	-18.743		50.0uM
1 (Plate 1)	M4	LEM00230987	-14.99		50.0uM
1 (Plate 1)	M5	LEM00230987	-11.464		50.0uM

B3

CCRF-CEM (IC50 > 50 µM)

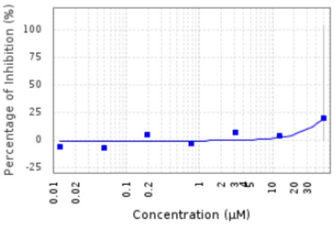

IC50: >50
slope=1.500 min=-1.093 max=103.909 err=166.109 R^2=0.6773

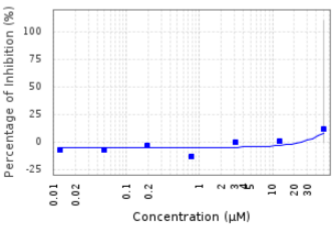

IC50: >50
slope=1.500 min=-5.292 max=110.000 err=122.482 R^2=0.6653

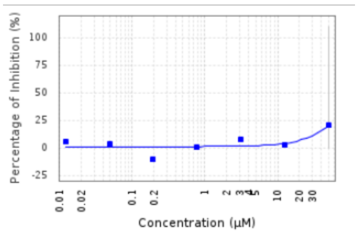

IC50: >50 pass
slope=1.500 min=0.937 max=110.000 err=195.269 R^2=0.6122

CEM-DNR (IC50 > 50 µM)

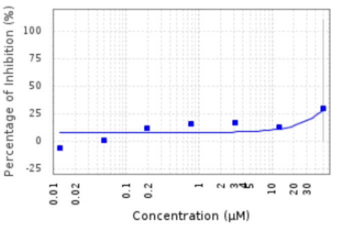

IC50: >50
slope=1.500 min=7.651 max=110.000 err=419.365 R^2=0.4990

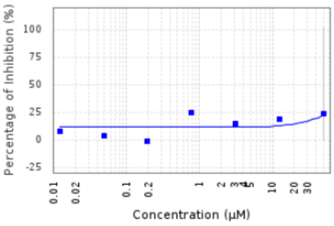

IC50: >50
slope=1.500 min=11.019 max=101.878 err=460.725 R^2=0.2359

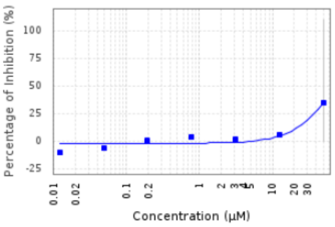

IC50: >50
slope=1.500 min=-2.224 max=110.000 err=133.894 R^2=0.8957

K562 (IC50 > 50 µM)

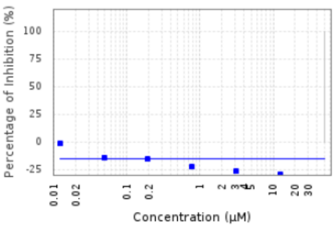

IC50: >50
slope=11.308 min=-15.000 max=100.000 err=1807.941 R^2=-0.3344

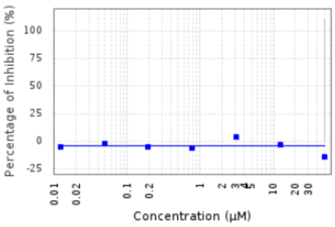

IC50: >50
slope=7.920 min=-4.938 max=110.000 err=171.985 R^2=-0.0000

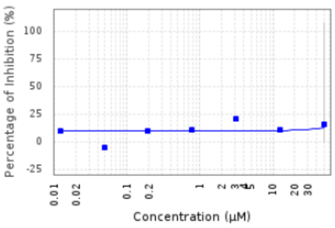

IC50: >50
slope=1.500 min=9.319 max=107.451 err=358.715 R^2=0.0708

K562-TAX (IC50 > 50 µM)

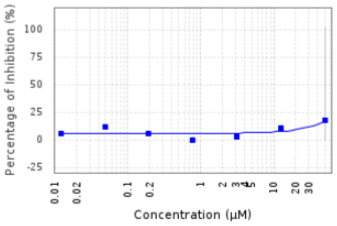

IC50: >50 pass
slope=1.500 min=5.747 max=101.780 err=103.971 R^2=0.5577

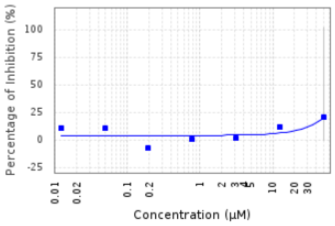

IC50: >50 pass
slope=1.500 min=3.858 max=101.034 err=285.591 R^2=0.4532

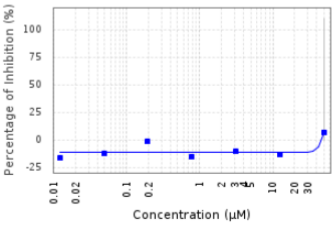

IC50: >50 pass
slope=8.589 min=-11.834 max=109.994 err=137.257 R^2=0.6799

A549 (IC50 > 50 µM)

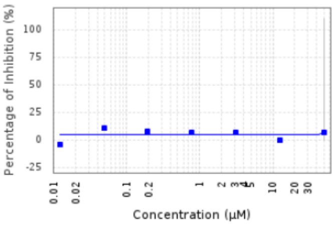

IC50: >50
slope=8.415 min=4.244 max=110.000 err=142.932 R^2=0.0275

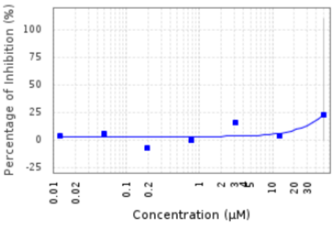

IC50: >50
slope=1.500 min=2.809 max=110.000 err=282.703 R^2=0.5380

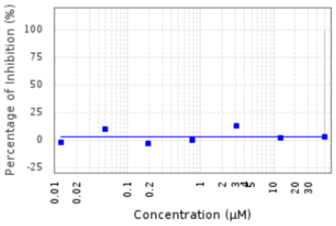

IC50: >50
slope=27.873 min=2.707 max=100.000 err=216.492 R^2=0.0000

U2OS (IC50 > 50 µM)

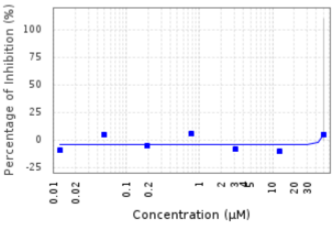

IC50: >50 pass
slope=8.721 min=-4.206 max=110.000 err=266.927 R^2=0.2075

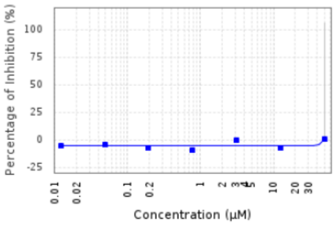

IC50: >50 pass
slope=8.393 min=-5.917 max=105.369 err=52.671 R^2=0.4015

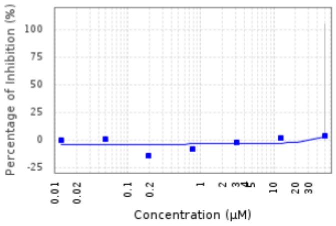

IC50: >50 pass
slope=1.500 min=-4.009 max=104.674 err=185.687 R^2=0.2176

HCT116 (IC50 > 50 µM)

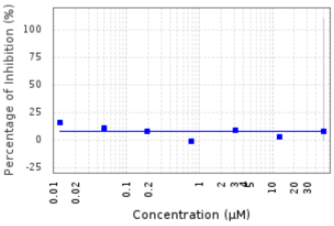

IC50: >50
slope=7.543 min=7.128 max=110.000 err=166.938 R^2=0.0037

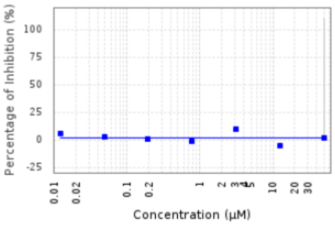

IC50: >50
slope=5.892 min=1.653 max=110.000 err=137.721 R^2=0.0001

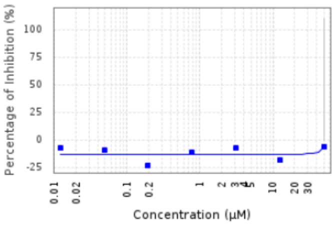

IC50: >50
slope=8.539 min=-13.074 max=110.000 err=199.255 R^2=0.1575

HCT116p53-/- (IC50 > 50 µM)

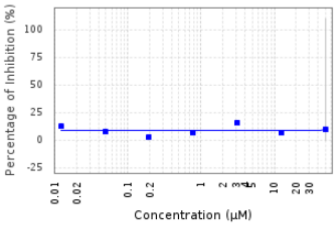

IC50: >50
slope=8.021 min=8.451 max=110.000 err=107.242 R^2=0.0168

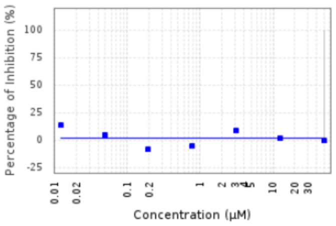

IC50: >50
slope=17.645 min=1.926 max=100.000 err=345.877 R^2=0.0000

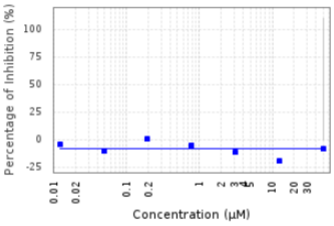

IC50: >50 pass
slope=11.471 min=-8.552 max=109.844 err=241.821 R^2=0.0002

MRC5 (IC50 > 50 µM)

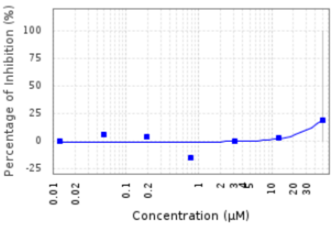

IC50: >50
slope=1.500 min=-1.241 max=100.000 err=292.675 R^2=0.5325

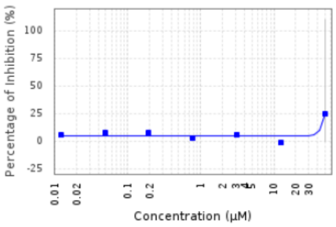

IC50: >50
slope=9.376 min=4.459 max=110.000 err=62.163 R^2=0.8447

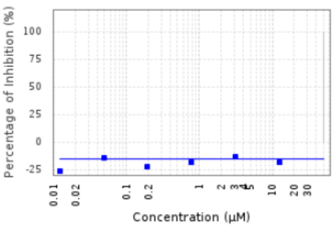

IC50: >50
slope=7.816 min=-15.000 max=100.000 err=804.984 R^2=-0.6815

BJ (IC50 > 50 µM)

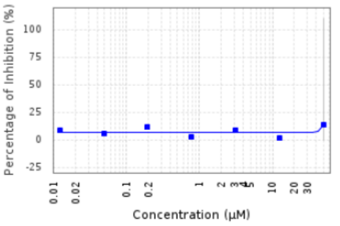

IC50: >50
slope=8.768 min=6.289 max=110.000 err=82.013 R^2=0.3443

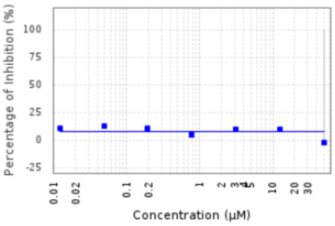

IC50: >50
slope=12.670 min=7.753 max=100.000 err=162.392 R^2=-0.0000

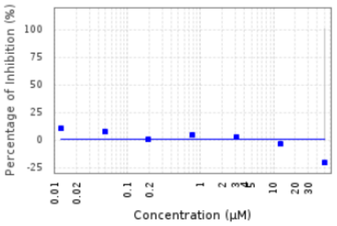

IC50: >50
slope=9.057 min=0.167 max=100.283 err=636.258 R^2=-0.0000

B4

CCRF-CEM (IC50 = 32.89 ± 10.75 µM)

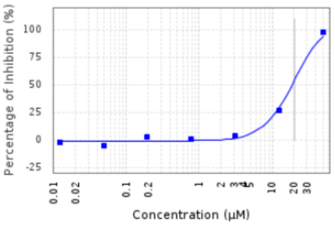

IC50: 20.547 *(17.699 - 23.854)* pass
slope=2.000 min=-1.156 max=110.000 err=51.255 R^2=0.9937

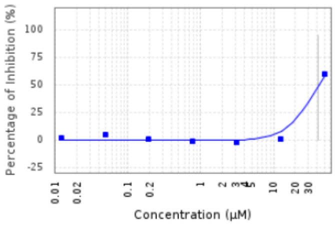

IC50: 40.182 *(35.245 - 45.811)* pass
slope=2.000 min=-0.953 max=95.000 err=93.332 R^2=0.9695

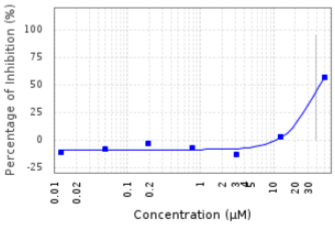

IC50: 37.932 *(33.631 - 42.782)* pass
slope=1.949 min=-9.107 max=95.000 err=71.487 R^2=0.9804

CEM-DNR (IC50 > 50 µM)

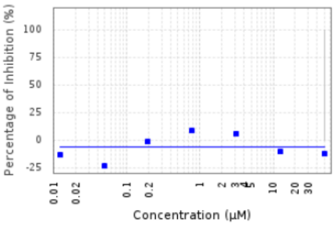

IC50: >50
slope=8.024 min=-6.882 max=100.000 err=789.024 R^2=-0.0000

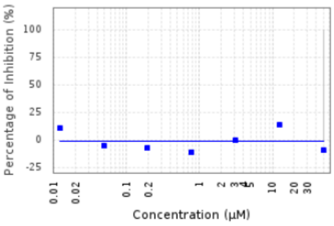

IC50: >50
slope=6.798 min=-1.200 max=100.000 err=576.372 R^2=-0.0000

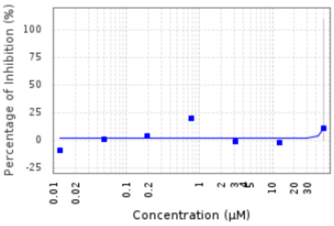

IC50: >50
slope=8.688 min=1.646 max=108.994 err=491.694 R^2=0.1189

K562 (IC50 > 50 µM)

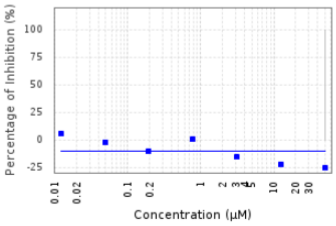

IC50: >50
slope=11.558 min=-10.063 max=100.000 err=845.608 R^2=-0.0000

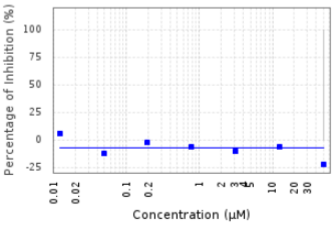

IC50: >50
slope=8.222 min=-7.895 max=100.000 err=461.575 R^2=-0.0000

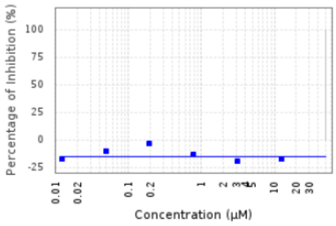

IC50: >50
slope=10.476 min=-15.000 max=100.000 err=3661.639 R^2=-0.1116

K562-TAX (IC50 > 50 μM)

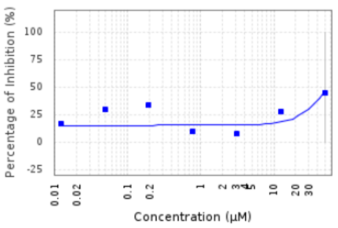

IC50: >50 pass
slope=2.000 min=15.000 max=100.000 err=766.050 R^2=0.3124

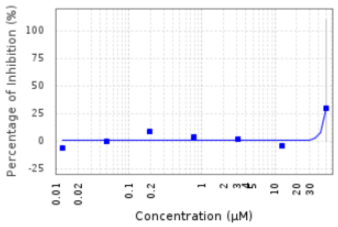

IC50: >50 pass
slope=9.349 min=0.391 max=110.000 err=147.569 R^2=0.8328

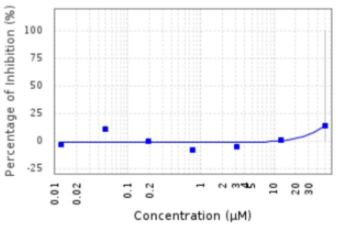

IC50: >50 pass
slope=1.770 min=-1.703 max=100.000 err=215.892 R^2=0.4823

A549 (IC50 > 50 μM)

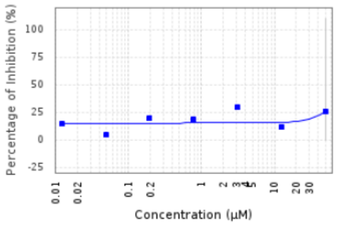

IC50: >50
slope=2.089 min=15.000 max=110.000 err=382.668 R^2=0.1442

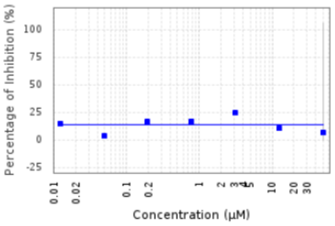

IC50: >50
slope=12.281 min=13.161 max=105.237 err=294.526 R^2=0.0000

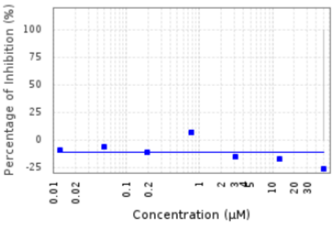

IC50: >50 pass
slope=13.139 min=-11.673 max=100.000 err=654.168 R^2=-0.0000

U2OS (IC50 > 50 μM)

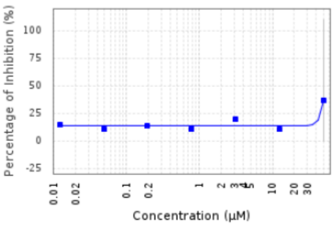

IC50: >50 pass
slope=9.487 min=13.135 max=110.000 err=63.090 R^2=0.8829

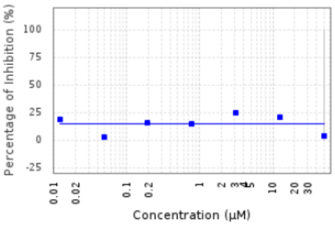

IC50: >50 pass
slope=12.473 min=14.469 max=100.000 err=407.899 R^2=0.0000

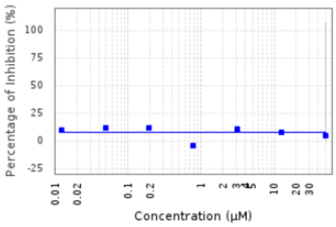

IC50: >50 pass
slope=8.096 min=7.267 max=106.772 err=201.883 R^2=-0.0000

HCT116 (IC50 > 50 μM)

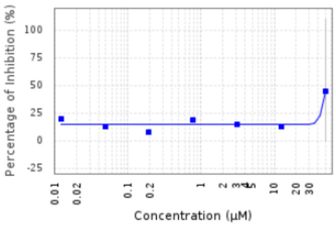

IC50: >50
slope=9.669 min=14.165 max=110.000 err=98.964 R^2=0.8907

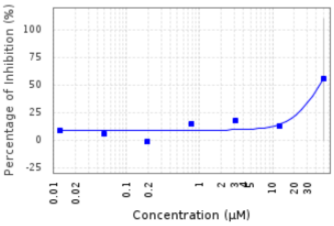

IC50: >50
slope=1.941 min=8.695 max=110.000 err=218.489 R^2=0.8923

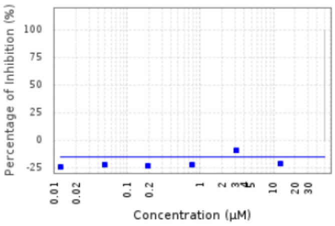

IC50: >50
slope=8.298 min=-15.000 max=100.000 err=681.073 R^2=-1.2810

HCT116p53-/- (IC50 > 50 μM)

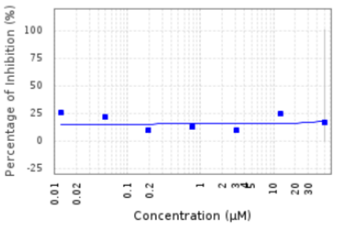

IC50: >50
slope=1.500 min=15.000 max=100.007 err=282.205 R^2=-0.0889

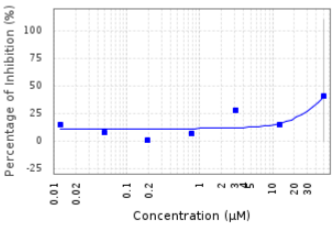

IC50: >50
slope=1.500 min=10.893 max=110.000 err=431.148 R^2=0.6255

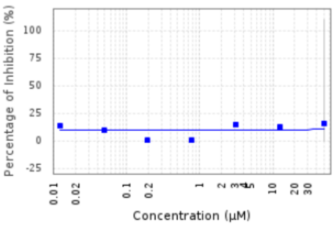

IC50: >50 pass
slope=1.500 min=9.296 max=110.000 err=254.584 R^2=0.0570

MRC5 (IC50 > 50 µM)

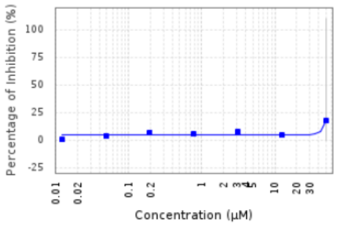

IC50: >50
slope=8.858 min=4.698 max=110.000 err=33.941 R^2=0.7968

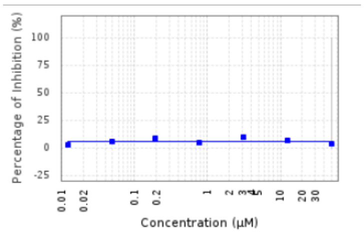

IC50: >50
slope=10.995 min=5.687 max=100.000 err=45.975 R^2=0.0000

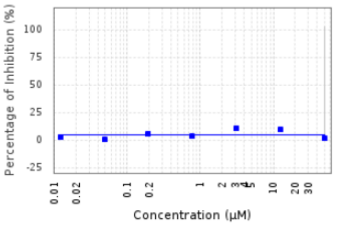

IC50: >50
slope=8.669 min=4.876 max=102.040 err=95.156 R^2=0.0000

BJ (IC50 > 50 µM)

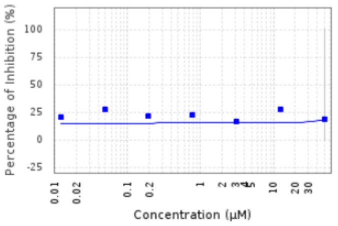

IC50: >50
slope=1.500 min=15.000 max=100.052 err=435.345 R^2=-3.1998

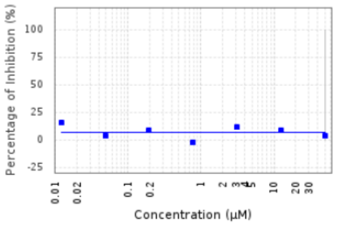

IC50: >50
slope=5.950 min=6.855 max=100.000 err=194.620 R^2=0.0000

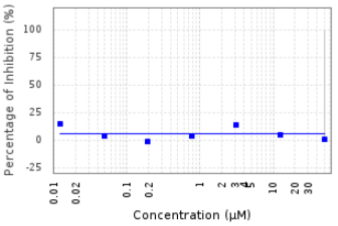

IC50: >50
slope=3.703 min=5.561 max=100.000 err=227.147 R^2=-0.0000

B5

CCRF-CEM (IC50 = 27.75 ± 1.74 µM)

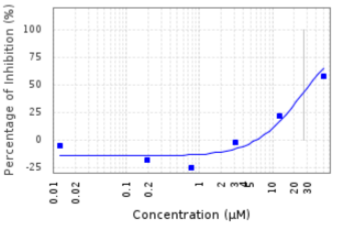

IC50: 26.671 *(8.049 - 88.379)* pass
slope=1.300 min=-15.000 max=100.000 err=777.646 R^2=0.8700

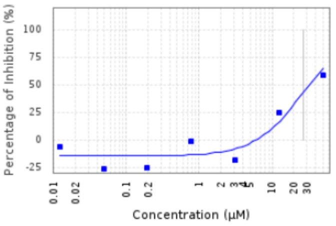

IC50: 26.826 *(8.4912 - 84.752)* pass
slope=1.300 min=-15.000 max=100.000 err=708.348 R^2=0.8765

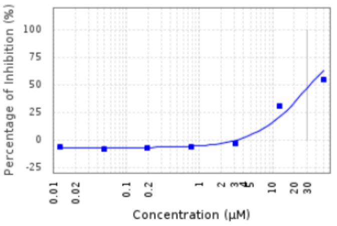

IC50: 29.762 *(14.117 - 62.744)* pass
slope=1.200 min=-7.324 max=100.000 err=177.067 R^2=0.9529

CEM-DNR (IC50 = 36.33 ± 2.93 µM)

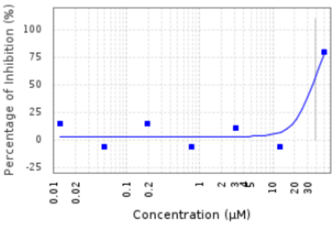

IC50: 38.083 *(2.7347 - 530.32)* pass
slope=3.000 min=2.765 max=110.000 err=695.342 R^2=0.8750

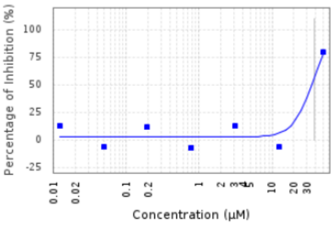

IC50: 38.004 *(3.2063 - 450.45)* pass
slope=3.000 min=2.254 max=110.000 err=626.925 R^2=0.8870

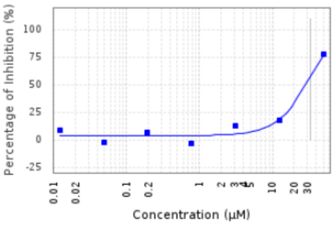

IC50: 32.942 *(0.0682 - 15901)* pass
slope=1.879 min=3.687 max=110.000 err=175.538 R^2=0.9620

K562 (IC50 > 50 μM)

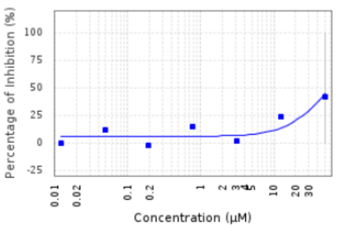

IC50: >50 pass
slope=1.500 min=5.502 max=100.000 err=358.817 R^2=0.7649

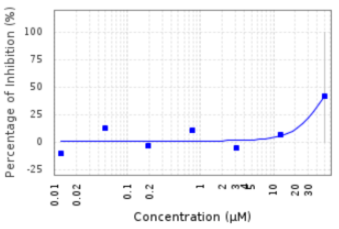

IC50: >50 pass
slope=1.912 min=0.838 max=100.000 err=414.287 R^2=0.7700

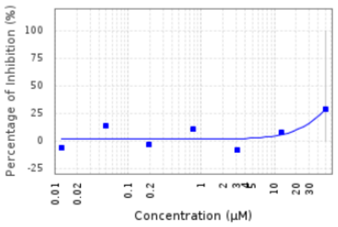

IC50: >50 pass
slope=1.629 min=1.353 max=100.000 err=397.089 R^2=0.6099

K562-TAX (IC50 = 35.78 ± 1.13 μM)

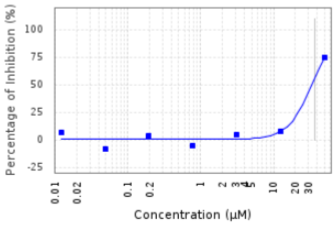

IC50: 36.633 *(4.31457E-8 - 3.11032E10)* pass
slope=2.432 min=0.089 max=110.000 err=171.389 R^2=0.9647

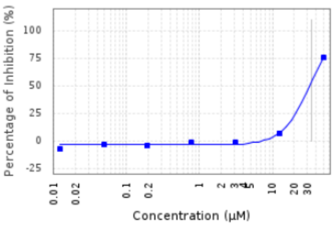

IC50: 34.495 *(1.031 - 1154.1)* pass
slope=2.199 min=-3.956 max=110.000 err=18.447 R^2=0.9965

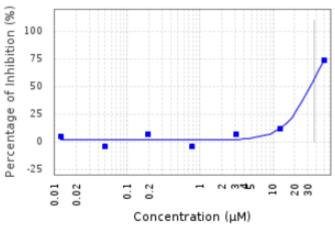

IC50: 36.217 *(0.0017 - 785656)* pass
slope=2.104 min=1.285 max=110.000 err=129.555 R^2=0.9710

A549 (IC50 > 50 μM)

Primary screen (50 μM)

1 (Plate 1)	E9	LEM00014568	-5.385		50.0uM
1 (Plate 1)	E10	LEM00014568	-6.1167		50.0uM
1 (Plate 1)	E11	LEM00014568	-8.4246		50.0uM

1 (Plate 1)	E9	LEM00014568	0.8210		50.0uM
1 (Plate 1)	E10	LEM00014568	-4.7049		50.0uM
1 (Plate 1)	E11	LEM00014568	-3.2963		50.0uM

U2OS (IC50 > 50 μM)

Primary screen (50 μM)

1 (Plate 1)	E10	LEM00014568	10.8		50.0uM
1 (Plate 1)	E11	LEM00014568	21.046		50.0uM
1 (Plate 1)	F9	LEM00014569	7.6907		50.0uM

1 (Plate 1)	E9	LEM00014568	17.423		50.0uM
1 (Plate 1)	E10	LEM00014568	15.482		50.0uM
1 (Plate 1)	E11	LEM00014568	15.611		50.0uM

HCT116 (IC50 > 50 μM)

Primary screen (50 μM)

1 (Plate 1)	E9	LEM00014568	5.598		50.0uM
1 (Plate 1)	E10	LEM00014568	2.5222		50.0uM
1 (Plate 1)	E11	LEM00014568	13.041		50.0uM

1 (Plate 1)	E9	LEM00014568	25.608		50.0uM
1 (Plate 1)	E10	LEM00014568	42.374		50.0uM
1 (Plate 1)	E11	LEM00014568	26.036		50.0uM

HCT116p53-/- (IC50 > 50 μM)

Primary screen (50 μM)

1 (Plate 1)	E9	LEM00014568	11.686		50.0uM
1 (Plate 1)	E10	LEM00014568	2.3646		50.0uM
1 (Plate 1)	E11	LEM00014568	22.104		50.0uM

1 (Plate 1)	E9	LEM00014568	6.8045		50.0uM
1 (Plate 1)	E10	LEM00014568	13.476		50.0uM
1 (Plate 1)	E11	LEM00014568	13.542		50.0uM

MRC5 (IC50 > 50 µM)

Primary screen (50 µM)

1 (Plate 1)	E9	LEM00014568	-22.41		50.0uM
1 (Plate 1)	E10	LEM00014568	-14.745		50.0uM
1 (Plate 1)	E11	LEM00014568	-13.851		50.0uM

1 (Plate 1)	E9	LEM00014568	-9.9459		50.0uM
1 (Plate 1)	E10	LEM00014568	-12.259		50.0uM
1 (Plate 1)	E11	LEM00014568	-6.3607		50.0uM

BJ (IC50 > 50 µM)

Primary screen (50 µM)

1 (Plate 1)	E9	LEM00014568	-9.9005		50.0uM
1 (Plate 1)	E10	LEM00014568	-3.5797		50.0uM
1 (Plate 1)	E11	LEM00014568	-3.2572		50.0uM

1 (Plate 1)	E9	LEM00014568	-8.1929		50.0uM
1 (Plate 1)	E10	LEM00014568	-11.555		50.0uM
1 (Plate 1)	E11	LEM00014568	-8.2601		50.0uM

B6

CCRF-CEM (IC50 > 50 µM)

Primary screen (50 µM)

2 (Plate 2)	E12	LEM00014569	-16.366		50.0uM
2 (Plate 2)	E13	LEM00014569	-11.141		50.0uM
2 (Plate 2)	E14	LEM00014569	-13.858		50.0uM

2 (Plate 2)	E12	LEM00014569	-20.06		50.0uM
2 (Plate 2)	E13	LEM00014569	-25.865		50.0uM
2 (Plate 2)	E14	LEM00014569	-24.86		50.0uM

CEM-DNR (IC50 > 50 µM)

Primary screen (50 µM)

1 (Plate 1)	F9	LEM00014569	-0.6706		50.0uM
1 (Plate 1)	F10	LEM00014569	-1.0317		50.0uM
1 (Plate 1)	F11	LEM00014569	-0.4643		50.0uM

1 (Plate 1)	F9	LEM00014569	-1.3951		50.0uM
1 (Plate 1)	F10	LEM00014569	-1.4998		50.0uM
1 (Plate 1)	F11	LEM00014569	-2.5994		50.0uM

K562 (IC50 > 50 µM)

Primary screen (50 µM)

1 (Plate 1)	F9	LEM00014569	0.3459		50.0uM
1 (Plate 1)	F10	LEM00014569	-1.5672		50.0uM
1 (Plate 1)	F11	LEM00014569	-0.3715		50.0uM

1 (Plate 1)	F9	LEM00014569	-1.4888		50.0uM
1 (Plate 1)	F10	LEM00014569	-2.071		50.0uM
1 (Plate 1)	F11	LEM00014569	-0.6737		50.0uM

K562-TAX (IC50 > 50 µM)

Primary screen (50 µM)

1 (Plate 1)	F9	LEM00014569	-1.199		50.0uM
1 (Plate 1)	F10	LEM00014569	-1.4579		50.0uM
1 (Plate 1)	F11	LEM00014569	-2.2864		50.0uM

1 (Plate 1)	F9	LEM00014569	0.1722		50.0uM
1 (Plate 1)	F10	LEM00014569	-2.4627		50.0uM
1 (Plate 1)	F11	LEM00014569	-2.5144		50.0uM

A549 (IC50 > 50 µM)

Primary screen (50 µM)

1 (Plate 1)	F9	LEM00014569	-2.4017		50.0uM
1 (Plate 1)	F10	LEM00014569	-6.2856		50.0uM
1 (Plate 1)	F11	LEM00014569	-6.9048		50.0uM

1 (Plate 1)	F9	LEM00014569	-2.5379		50.0uM
1 (Plate 1)	F10	LEM00014569	-5.0841		50.0uM
1 (Plate 1)	F11	LEM00014569	-3.3505		50.0uM

U2OS (IC50 > 50 µM)

Primary screen (50 µM)

1 (Plate 1)	F9	LEM00014569	7.6907		50.0uM
1 (Plate 1)	F10	LEM00014569	-28.843		50.0uM
1 (Plate 1)	F11	LEM00014569	-5.1703		50.0uM

1 (Plate 1)	F9	LEM00014569	-24.893		50.0uM
1 (Plate 1)	F10	LEM00014569	-6.2585		50.0uM
1 (Plate 1)	F11	LEM00014569	-9.4289		50.0uM

HCT116 (IC50 > 50 µM)

Primary screen (50 µM)

1 (Plate 1)	F9	LEM00014569	-17.04		50.0uM
1 (Plate 1)	F10	LEM00014569	-13.657		50.0uM
1 (Plate 1)	F11	LEM00014569	-11.934		50.0uM

1 (Plate 1)	F9	LEM00014569	-17.47		50.0uM
1 (Plate 1)	F10	LEM00014569	-13.003		50.0uM
1 (Plate 1)	F11	LEM00014569	-12.697		50.0uM

HCT116p53-/- (IC50 > 50 µM)

Primary screen (50 µM)

1 (Plate 1)	F9	LEM00014569	-21.899		50.0uM
1 (Plate 1)	F10	LEM00014569	-15.387		50.0uM
1 (Plate 1)	F11	LEM00014569	-32.317		50.0uM

1 (Plate 1)	F9	LEM00014569	-18.145		50.0uM
1 (Plate 1)	F10	LEM00014569	-19.079		50.0uM
1 (Plate 1)	F11	LEM00014569	-6.8712		50.0uM

MRC5 (IC50 > 50 µM)

Primary screen (50 µM)

1 (Plate 1)	F9	LEM00014569	-18.386		50.0uM
1 (Plate 1)	F10	LEM00014569	-6.889		50.0uM
1 (Plate 1)	F11	LEM00014569	-18.003		50.0uM

1 (Plate 1)	F9	LEM00014569	-6.3029		50.0uM
1 (Plate 1)	F10	LEM00014569	-10.871		50.0uM
1 (Plate 1)	F11	LEM00014569	-10.813		50.0uM

BJ (IC50 > 50 µM)

Primary screen (50 µM)

1 (Plate 1)	F9	LEM00014569	-20.865		50.0uM
1 (Plate 1)	F10	LEM00014569	-16.608		50.0uM
1 (Plate 1)	F11	LEM00014569	-18.027		50.0uM

1 (Plate 1)	F9	LEM00014569	-16.194		50.0uM
1 (Plate 1)	F10	LEM00014569	-17.203		50.0uM
1 (Plate 1)	F11	LEM00014569	-17.674		50.0uM

B7

CCRF-CEM (IC50 > 50 μM)

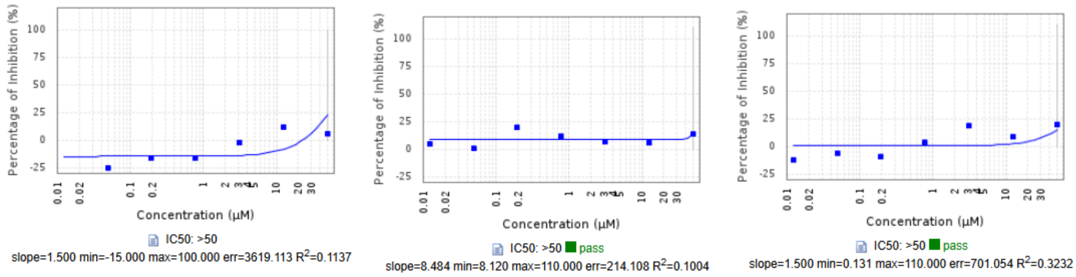

CEM-DNR (IC50 = 39.86 ± 2.94 μM)

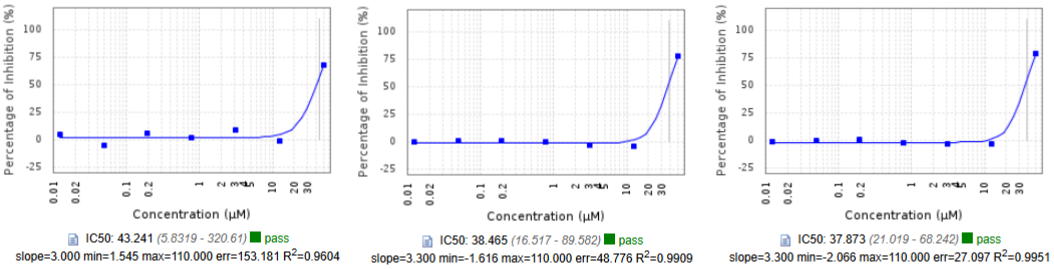

K562 (IC50 > 50 μM)

Primary screen (50 μM)

1 (Plate 1)	C3	LEM00018223	29.152		50.0uM
1 (Plate 1)	C4	LEM00018223	20.8		50.0uM
1 (Plate 1)	C5	LEM00018223	29.003		50.0uM

1 (Plate 1)	C3	LEM00018223	39.632		50.0uM
1 (Plate 1)	C4	LEM00018223	35.718		50.0uM
1 (Plate 1)	C5	LEM00018223	38.748		50.0uM

K562-TAX (IC50 > 50 μM)

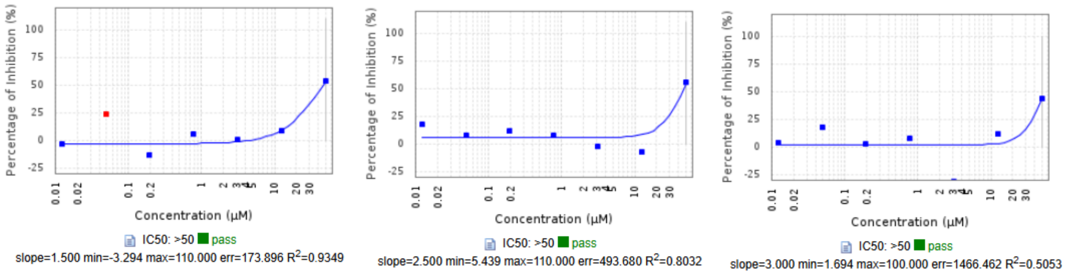

A549 (IC50 > 50 μM)

Primary screen (50 μM)

1 (Plate 1)	C3	LEM00018223	17.81		50.0uM
1 (Plate 1)	C4	LEM00018223	-0.2369		50.0uM
1 (Plate 1)	C5	LEM00018223	9.3768		50.0uM

1 (Plate 1)	C3	LEM00018223	48.643		50.0uM
1 (Plate 1)	C4	LEM00018223	32.639		50.0uM
1 (Plate 1)	C5	LEM00018223	27.405		50.0uM

U2OS (IC50 > 50 µM)

Primary screen (50 µM)

1 (Plate 1)	C3	LEM00018223	30.754	50.0uM
1 (Plate 1)	C4	LEM00018223	11.74	50.0uM
1 (Plate 1)	C5	LEM00018223	27.237	50.0uM

1 (Plate 1)	C3	LEM00018223	35.488	50.0uM
1 (Plate 1)	C4	LEM00018223	16.045	50.0uM
1 (Plate 1)	C5	LEM00018223	28.143	50.0uM

HCT116 (IC50 > 50 µM)

Primary screen (50 µM)

1 (Plate 1)	C3	LEM00018223	29.165	50.0uM
1 (Plate 1)	C4	LEM00018223	4.7872	50.0uM
1 (Plate 1)	C5	LEM00018223	13.806	50.0uM

1 (Plate 1)	C3	LEM00018223	22.898	50.0uM
1 (Plate 1)	C4	LEM00018223	16.635	50.0uM
1 (Plate 1)	C5	LEM00018223	22.286	50.0uM

HCT116p53-/- (IC50 > 50 µM)

Primary screen (50 µM)

1 (Plate 1)	C3	LEM00018223	18.639	50.0uM
1 (Plate 1)	C4	LEM00018223	8.0524	50.0uM
1 (Plate 1)	C5	LEM00018223	16.011	50.0uM

1 (Plate 1)	C3	LEM00018223	20.119	50.0uM
1 (Plate 1)	C4	LEM00018223	10.964	50.0uM
1 (Plate 1)	C5	LEM00018223	12.78	50.0uM

MRC5 (IC50 > 50 µM)

Primary screen (50 µM)

1 (Plate 1)	C3	LEM00018223	19.963	50.0uM
1 (Plate 1)	C4	LEM00018223	-4.4361	50.0uM
1 (Plate 1)	C5	LEM00018223	13.733	50.0uM

1 (Plate 1)	C3	LEM00018223	15.331	50.0uM
1 (Plate 1)	C4	LEM00018223	-2.8015	50.0uM
1 (Plate 1)	C5	LEM00018223	12.145	50.0uM

BJ (IC50 > 50 µM)

Primary screen (50 µM)

1 (Plate 1)	C3	LEM00018223	-12.076	50.0uM
1 (Plate 1)	C4	LEM00018223	-32.694	50.0uM
1 (Plate 1)	C5	LEM00018223	-15.577	50.0uM

1 (Plate 1)	C3	LEM00018223	-4.5425	50.0uM
1 (Plate 1)	C4	LEM00018223	-26.384	50.0uM
1 (Plate 1)	C5	LEM00018223	-9.3324	50.0uM

B8

CCRF-CEM (IC50 > 50 µM)

Primary screen (50 µM)

2 (Plate 2)	F12	LEM00014570	16.237	50.0uM
2 (Plate 2)	F13	LEM00014570	14.565	50.0uM
2 (Plate 2)	F14	LEM00014570	14.252	50.0uM

2 (Plate 2)	F12	LEM00014570	8.7385	50.0uM
2 (Plate 2)	F13	LEM00014570	8.7385	50.0uM
2 (Plate 2)	F14	LEM00014570	8.9617	50.0uM

CEM-DNR (IC50 > 50 µM)

Primary screen (50 µM)

1 (Plate 1)	G9	LEM00014570	-0.7222		50.0uM
1 (Plate 1)	G10	LEM00014570	3.972		50.0uM
1 (Plate 1)	G11	LEM00014570	3.1983		50.0uM

1 (Plate 1)	G9	LEM00014570	-1.6569		50.0uM
1 (Plate 1)	G10	LEM00014570	-2.0235		50.0uM
1 (Plate 1)	G11	LEM00014570	-0.8715		50.0uM

K562 (IC50 > 50 µM)

Primary screen (50 µM)

1 (Plate 1)	G9	LEM00014570	-4.0183		50.0uM
1 (Plate 1)	G10	LEM00014570	-7.4858		50.0uM
1 (Plate 1)	G11	LEM00014570	-3.9585		50.0uM

1 (Plate 1)	G9	LEM00014570	-5.2149		50.0uM
1 (Plate 1)	G10	LEM00014570	-7.893		50.0uM
1 (Plate 1)	G11	LEM00014570	-4.5744		50.0uM

K562-TAX (IC50 > 50 µM)

Primary screen (50 µM)

1 (Plate 1)	G9	LEM00014570	-1.4579		50.0uM
1 (Plate 1)	G10	LEM00014570	-3.4774		50.0uM
1 (Plate 1)	G11	LEM00014570	-0.5776		50.0uM

1 (Plate 1)	G9	LEM00014570	-0.1894		50.0uM
1 (Plate 1)	G10	LEM00014570	-0.0861		50.0uM
1 (Plate 1)	G11	LEM00014570	1.1022		50.0uM

A549 (IC50 > 50 µM)

Primary screen (50 µM)

1 (Plate 1)	G9	LEM00014570	-1.3885		50.0uM
1 (Plate 1)	G10	LEM00014570	-7.7491		50.0uM
1 (Plate 1)	G11	LEM00014570	-3.9215		50.0uM

1 (Plate 1)	G9	LEM00014570	-6.9261		50.0uM
1 (Plate 1)	G10	LEM00014570	-3.1338		50.0uM
1 (Plate 1)	G11	LEM00014570	-0.8043		50.0uM

U2OS (IC50 > 50 µM)

Primary screen (50 µM)

1 (Plate 1)	G9	LEM00014570	14.687		50.0uM
1 (Plate 1)	G10	LEM00014570	-0.9304		50.0uM
1 (Plate 1)	G11	LEM00014570	-6.1596		50.0uM

1 (Plate 1)	G9	LEM00014570	20.464		50.0uM
1 (Plate 1)	G10	LEM00014570	19.493		50.0uM
1 (Plate 1)	G11	LEM00014570	19.105		50.0uM

HCT116 (IC50 > 50 µM)

Primary screen (50 µM)

1 (Plate 1)	G9	LEM00014570	-10.212		50.0uM
1 (Plate 1)	G10	LEM00014570	-11.38		50.0uM
1 (Plate 1)	G11	LEM00014570	-9.8426		50.0uM

1 (Plate 1)	G9	LEM00014570	-13.37		50.0uM
1 (Plate 1)	G10	LEM00014570	-12.207		50.0uM
1 (Plate 1)	G11	LEM00014570	-14.838		50.0uM

HCT116p53-/- (IC50 > 50 µM)

Primary screen (50 µM)

1 (Plate 1)	G9	LEM00014570	-26.079		50.0uM
1 (Plate 1)	G10	LEM00014570	-36.223		50.0uM
1 (Plate 1)	G11	LEM00014570	-29.506		50.0uM

1 (Plate 1)	G9	LEM00014570	-23.082		50.0uM
1 (Plate 1)	G10	LEM00014570	-17.879		50.0uM
1 (Plate 1)	G11	LEM00014570	-21.414		50.0uM

MRC5 (IC50 > 50 µM)

Primary screen (50 µM)

1 (Plate 1)	G9	LEM00014570	-15.001		50.0uM
1 (Plate 1)	G10	LEM00014570	-12.829		50.0uM
1 (Plate 1)	G11	LEM00014570	-10.977		50.0uM

1 (Plate 1)	G9	LEM00014570	-6.1294		50.0uM
1 (Plate 1)	G10	LEM00014570	-9.3676		50.0uM
1 (Plate 1)	G11	LEM00014570	-5.3777		50.0uM

BJ (IC50 > 50 µM)

Primary screen (50 µM)

1 (Plate 1)	G9	LEM00014570	-5.7726		50.0uM
1 (Plate 1)	G10	LEM00014570	-6.8691		50.0uM
1 (Plate 1)	G11	LEM00014570	-10.03		50.0uM

1 (Plate 1)	G9	LEM00014570	-8.3946		50.0uM
1 (Plate 1)	G10	LEM00014570	-8.4618		50.0uM
1 (Plate 1)	G11	LEM00014570	-4.8309		50.0uM

B9

CCRF-CEM (IC50 > 50 µM)

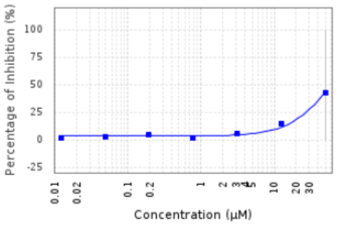

IC50: >50 pass

slope=1.500 min=3.230 max=100.000 err=25.398 R^2=0.9806

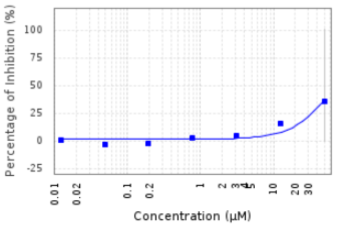

IC50: >50 pass

slope=1.500 min=1.174 max=100.003 err=101.587 R^2=0.9102

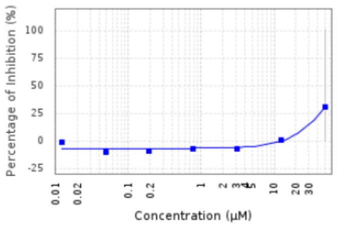

IC50: >50 pass

slope=1.500 min=-7.121 max=100.027 err=51.349 R^2=0.9574

CEM-DNR (IC50 > 50 µM)

Primary screen (50 µM)

1 (Plate 1)	I9	LEM00014572	7.9441		50.0uM
1 (Plate 1)	I10	LEM00014572	-2.4761		50.0uM
1 (Plate 1)	I11	LEM00014572	1.9602		50.0uM

1 (Plate 1)	I9	LEM00014572	-0.4002		50.0uM
1 (Plate 1)	I10	LEM00014572	-0.9238		50.0uM
1 (Plate 1)	I11	LEM00014572	-0.5573		50.0uM

K562 (IC50 > 50 µM)

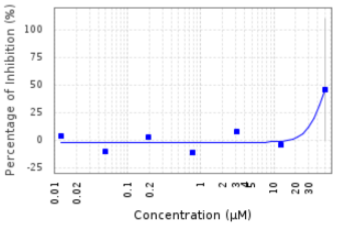

IC50: >50 pass

slope=3.300 min=-2.301 max=110.000 err=308.092 R^2=0.8639

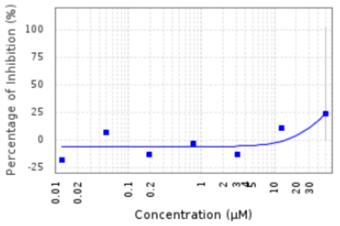

IC50: >50 pass

slope=1.500 min=-6.583 max=101.163 err=579.022 R^2=0.5878

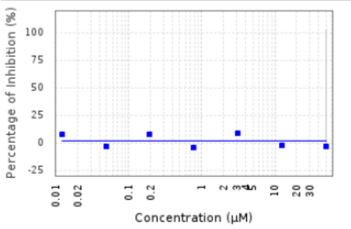

IC50: >50 pass

slope=9.517 min=1.378 max=102.851 err=215.523 R^2=-0.0000

K562-TAX (IC50 > 50 µM)

Primary screen (50 µM)

1 (Plate 1)	I9	LEM00014572	-1.8721		50.0uM
1 (Plate 1)	I10	LEM00014572	2.7883		50.0uM
1 (Plate 1)	I11	LEM00014572	6.413		50.0uM

1 (Plate 1)	I9	LEM00014572	5.9071		50.0uM
1 (Plate 1)	I10	LEM00014572	5.3904		50.0uM
1 (Plate 1)	I11	LEM00014572	2.7038		50.0uM

A549 (IC50 > 50 µM)

Primary screen (50 µM)

1 (Plate 1)	I9	LEM00014572	1.6511		50.0uM
1 (Plate 1)	I10	LEM00014572	3.6775		50.0uM
1 (Plate 1)	I11	LEM00014572	3.6775		50.0uM

1 (Plate 1)	I9	LEM00014572	3.0963		50.0uM
1 (Plate 1)	I10	LEM00014572	1.6878		50.0uM
1 (Plate 1)	I11	LEM00014572	-2.4837		50.0uM

U2OS (IC50 > 50 µM)

Primary screen (50 µM)

1 (Plate 1)	I9	LEM00014572	3.5921		50.0uM
1 (Plate 1)	I10	LEM00014572	9.5986		50.0uM
1 (Plate 1)	I11	LEM00014572	17.442		50.0uM

1 (Plate 1)	I9	LEM00014572	-4.5762		50.0uM
1 (Plate 1)	I10	LEM00014572	-2.441		50.0uM
1 (Plate 1)	I11	LEM00014572	-6.7761		50.0uM

HCT116 (IC50 > 50 µM)

Primary screen (50 µM)

1 (Plate 1)	I9	LEM00014572	-7.628		50.0uM
1 (Plate 1)	I10	LEM00014572	-8.4893		50.0uM
1 (Plate 1)	I11	LEM00014572	1.907		50.0uM

1 (Plate 1)	I9	LEM00014572	-8.4135		50.0uM
1 (Plate 1)	I10	LEM00014572	-17.102		50.0uM
1 (Plate 1)	I11	LEM00014572	-8.7195		50.0uM

HCT116p53-/- (IC50 > 50 µM)

Primary screen (50 µM)

1 (Plate 1)	I9	LEM00014572	-17.786		50.0uM
1 (Plate 1)	I10	LEM00014572	-27.999		50.0uM
1 (Plate 1)	I11	LEM00014572	-8.5332		50.0uM

1 (Plate 1)	I9	LEM00014572	-16.478		50.0uM
1 (Plate 1)	I10	LEM00014572	-8.1388		50.0uM
1 (Plate 1)	I11	LEM00014572	-10.073		50.0uM

MRC5 (IC50 > 50 µM)

Primary screen (50 µM)

1 (Plate 1)	I9	LEM00014572	-1.3322		50.0uM
1 (Plate 1)	I10	LEM00014572	7.3544		50.0uM
1 (Plate 1)	I11	LEM00014572	2.564		50.0uM

1 (Plate 1)	I9	LEM00014572	5.7247		50.0uM
1 (Plate 1)	I10	LEM00014572	-1.7347		50.0uM
1 (Plate 1)	I11	LEM00014572	2.8912		50.0uM

BJ (IC50 > 50 μM)

Primary screen (50 μM)

1 (Plate 1)	I9	LEM00014572	6.9336		50.0uM
1 (Plate 1)	I10	LEM00014572	3.6442		50.0uM
1 (Plate 1)	I11	LEM00014572	0.3547		50.0uM

1 (Plate 1)	I9	LEM00014572	8.4153		50.0uM
1 (Plate 1)	I10	LEM00014572	4.5826		50.0uM
1 (Plate 1)	I11	LEM00014572	8.0791		50.0uM

B10

CCRF-CEM (IC50 > 50 μM)

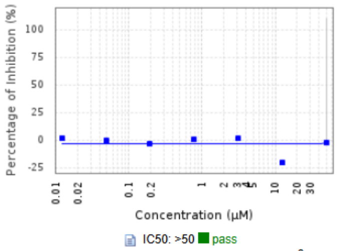

slope=11.119 min=-3.455 max=110.000 err=369.113 R^2=0.0032

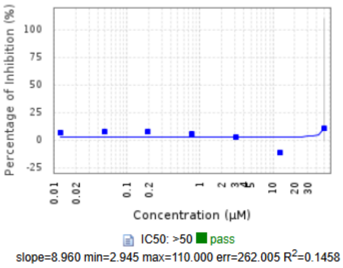

slope=8.960 min=2.945 max=110.000 err=262.005 R^2=0.1458

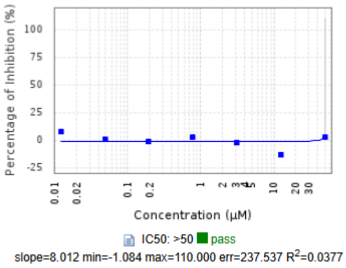

slope=8.012 min=-1.084 max=110.000 err=237.537 R^2=0.0377

CEM-DNR (IC50 > 50 μM)

Primary screen (50 μM)

1 (Plate 1)	H9	LEM00014571	-1.1349		50.0uM
1 (Plate 1)	H10	LEM00014571	-1.9086		50.0uM
1 (Plate 1)	H11	LEM00014571	-2.4761		50.0uM

1 (Plate 1)	H9	LEM00014571	-1.1856		50.0uM
1 (Plate 1)	H10	LEM00014571	-1.3951		50.0uM
1 (Plate 1)	H11	LEM00014571	-1.6569		50.0uM

K562 (IC50 > 50 μM)

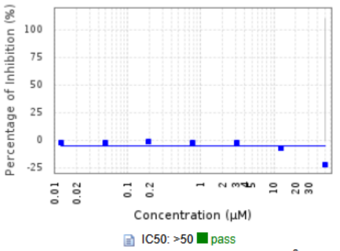

slope=6.044 min=-5.865 max=110.000 err=343.588 R^2=-0.0000

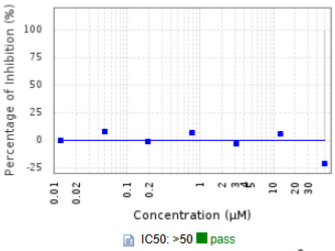

slope=14.003 min=-0.942 max=100.000 err=610.447 R^2=0.0000

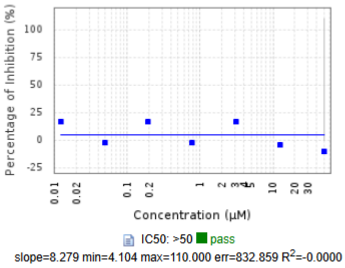

slope=8.279 min=4.104 max=110.000 err=832.859 R^2=-0.0000

K562-TAX (IC50 > 50 μM)

Primary screen (50 μM)

1 (Plate 1)	H9	LEM00014571	-2.7524		50.0uM
1 (Plate 1)	H10	LEM00014571	-1.4061		50.0uM
1 (Plate 1)	H11	LEM00014571	0.3027		50.0uM

1 (Plate 1)	H9	LEM00014571	-1.6361		50.0uM
1 (Plate 1)	H10	LEM00014571	-2.1527		50.0uM
1 (Plate 1)	H11	LEM00014571	-0.6028		50.0uM

A549 (IC50 > 50 µM)

Primary screen (50 µM)

1 (Plate 1)	H9	LEM00014571	-6.5108	●	50.0uM
1 (Plate 1)	H10	LEM00014571	-2.4017	●	50.0uM
1 (Plate 1)	H11	LEM00014571	-4.7095	●	50.0uM

1 (Plate 1)	H9	LEM00014571	-4.8674	●	50.0uM
1 (Plate 1)	H10	LEM00014571	-1.4544	●	50.0uM
1 (Plate 1)	H11	LEM00014571	-2.9171	●	50.0uM

U2OS (IC50 > 50 µM)

Primary screen (50 µM)

1 (Plate 1)	H9	LEM00014571	-18.809	●	50.0uM
1 (Plate 1)	H10	LEM00014571	-17.254	●	50.0uM
1 (Plate 1)	H11	LEM00014571	-31.74	●	50.0uM

1 (Plate 1)	H9	LEM00014571	-16.029	●	50.0uM
1 (Plate 1)	H10	LEM00014571	-9.2348	●	50.0uM
1 (Plate 1)	H11	LEM00014571	-12.599	●	50.0uM

HCT116 (IC50 > 50 µM)

Primary screen (50 µM)

1 (Plate 1)	H9	LEM00014571	-10.458	●	50.0uM
1 (Plate 1)	H10	LEM00014571	-12.488	●	50.0uM
1 (Plate 1)	H11	LEM00014571	-11.688	●	50.0uM

1 (Plate 1)	H9	LEM00014571	-9.6985	●	50.0uM
1 (Plate 1)	H10	LEM00014571	-12.819	●	50.0uM
1 (Plate 1)	H11	LEM00014571	-9.8209	●	50.0uM

HCT116p53-/- (IC50 > 50 µM)

Primary screen (50 µM)

1 (Plate 1)	H9	LEM00014571	-22.378	●	50.0uM
1 (Plate 1)	H10	LEM00014571	-32.385	●	50.0uM
1 (Plate 1)	H11	LEM00014571	-26.833	●	50.0uM

1 (Plate 1)	H9	LEM00014571	-21.881	●	50.0uM
1 (Plate 1)	H10	LEM00014571	-20.547	●	50.0uM
1 (Plate 1)	H11	LEM00014571	-20.881	●	50.0uM

MRC5 (IC50 > 50 µM)

Primary screen (50 µM)

1 (Plate 1)	H9	LEM00014571	-18.897	●	50.0uM
1 (Plate 1)	H10	LEM00014571	-19.088	●	50.0uM
1 (Plate 1)	H11	LEM00014571	-18.705	●	50.0uM

1 (Plate 1)	H9	LEM00014571	-10.119	●	50.0uM
1 (Plate 1)	H10	LEM00014571	-12.722	●	50.0uM
1 (Plate 1)	H11	LEM00014571	-10.351	●	50.0uM

BJ (IC50 > 50 µM)

Primary screen (50 µM)

1 (Plate 1)	H9	LEM00014571	-19.253	●	50.0uM
1 (Plate 1)	H10	LEM00014571	-17.447	●	50.0uM
1 (Plate 1)	H11	LEM00014571	-19.124	●	50.0uM

1 (Plate 1)	H9	LEM00014571	-20.027	●	50.0uM
1 (Plate 1)	H10	LEM00014571	-25.339	●	50.0uM
1 (Plate 1)	H11	LEM00014571	-20.161	●	50.0uM

CB1

CCRF-CEM (IC_{50} = 0.40 ± 0.018 μM)

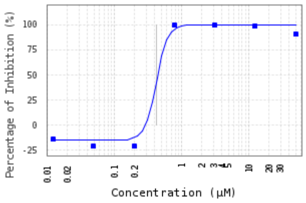

IC50: 0.4175 (0.2705 - 0.6444) pass
slope=5.000 min=-15.000 max=100.000 err=213.780 R^2=0.9907

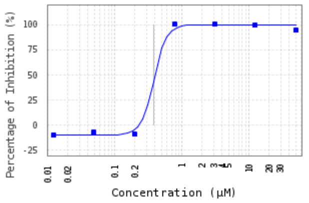

IC50: 0.3808 (0.2975 - 0.4873) pass
slope=4.500 min=-9.924 max=100.000 err=79.441 R^2=0.9960

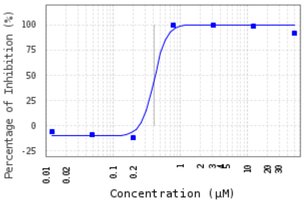

IC50: 0.4025 (0.2901 - 0.5584) pass
slope=4.500 min=-10.117 max=100.000 err=144.824 R^2=0.9925

CEM-DNR (IC_{50} = 2.07 ± 0.212 μM)

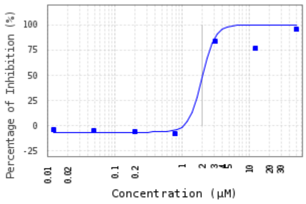

IC50: 1.9747 (1.3338 - 2.9237) pass
slope=4.500 min=-6.199 max=100.000 err=576.494 R^2=0.9601

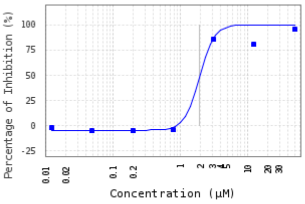

IC50: 1.9208 (1.3664 - 2.7) pass
slope=4.000 min=-4.245 max=100.000 err=408.743 R^2=0.9716

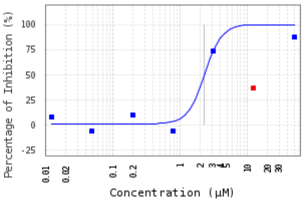

IC50: 2.3121 (1.7199 - 3.1082) pass
slope=3.500 min=1.459 max=100.000 err=428.736 R^2=0.9502

K562 (IC_{50} = 0.45 ± 0.054 μM)

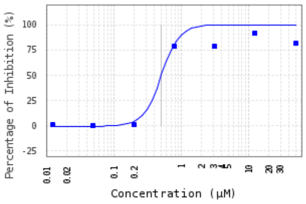

IC50: 0.5061 (0.3340 - 0.7668) pass
slope=3.200 min=-0.459 max=100.000 err=852.958 R^2=0.9267

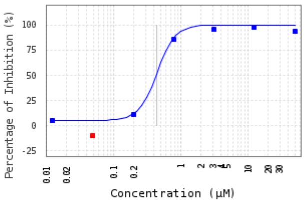

IC50: 0.4424 (0.3477 - 0.5629) pass
slope=3.147 min=5.141 max=100.000 err=70.509 R^2=0.9927

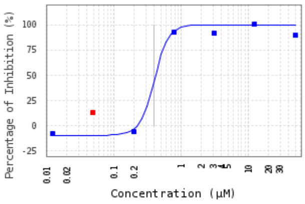

IC50: 0.3977 (0.2768 - 0.5715) pass
slope=4.000 min=-9.418 max=100.000 err=173.446 R^2=0.9872

K562-TAX (IC_{50} = 1.47 ± 0.361 μM)

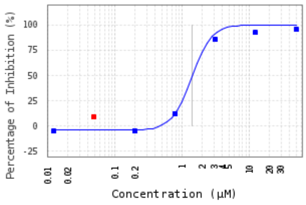

IC50: 1.3956 (1.134 - 1.7175) pass
slope=3.000 min=-4.059 max=100.000 err=106.940 R^2=0.9914

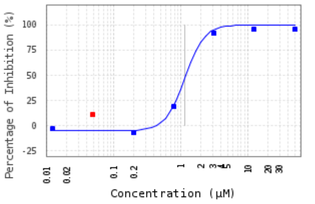

IC50: 1.1601 (0.9605 - 1.4011) pass
slope=3.054 min=-4.933 max=100.000 err=62.215 R^2=0.9951

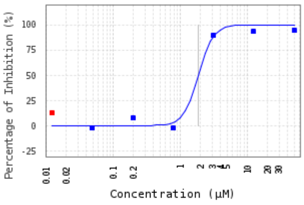

IC50: 1.8684 (1.4119 - 2.4725) pass
slope=4.000 min=0.582 max=100.000 err=165.148 R^2=0.9869

HCT116 (IC_{50} = 0.50 ± 0.032 μM)

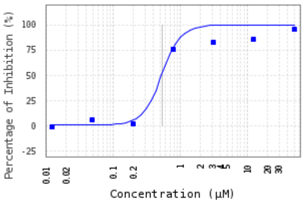

IC50: 0.5312 (0.3880 - 0.7272) pass
slope=3.000 min=1.034 max=100.000 err=536.778 R^2=0.9550

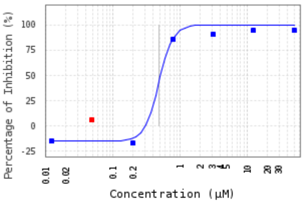

IC50: 0.4825 (0.3907 - 0.5960) pass
slope=4.000 min=-15.000 max=100.000 err=161.949 R^2=0.9894

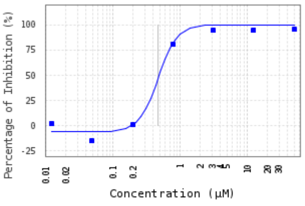

IC50: 0.4716 (0.3503 - 0.6349) pass
slope=3.028 min=-6.037 max=100.000 err=224.711 R^2=0.9858

HCT116p53-/- (IC_{50} = 0.58 ± 0.063 µM)

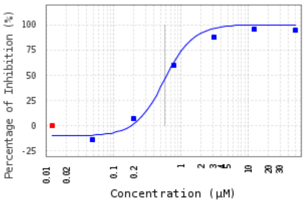

IC50: 0.5679 *(0.4510 - 0.7150)* pass
slope=2.000 min=-10.143 max=100.000 err=173.168 R^2=0.9846

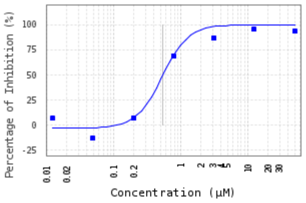

IC50: 0.5316 *(0.3962 - 0.7132)* pass
slope=2.200 min=-3.006 max=100.000 err=376.682 R^2=0.9717

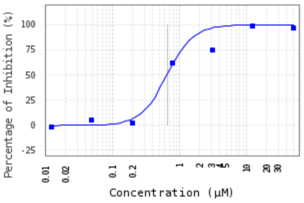

IC50: 0.6544 *(0.4701 - 0.9110)* pass
slope=2.200 min=-0.229 max=100.000 err=560.359 R^2=0.9541

U2OS (IC_{50} = 0.64 ± 0.019 µM)

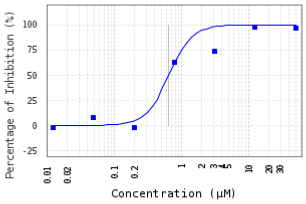

IC50: 0.6506 *(0.4641 - 0.9122)* pass
slope=2.500 min=0.351 max=100.000 err=732.395 R^2=0.9398

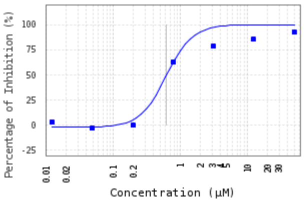

IC50: 0.6191 *(0.4320 - 0.8875)* pass
slope=2.200 min=-2.076 max=100.000 err=657.118 R^2=0.9425

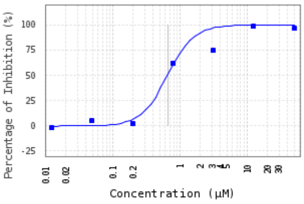

IC50: 0.6544 *(0.4701 - 0.9110)* pass
slope=2.200 min=-0.229 max=100.000 err=560.359 R^2=0.9541

MRC5 (IC_{50} = 5.69 ± 0.639 µM)

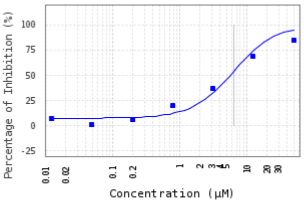

IC50: 6.3738 *(4.1315 - 9.833)* pass
slope=1.400 min=7.559 max=100.000 err=257.691 R^2=0.9603

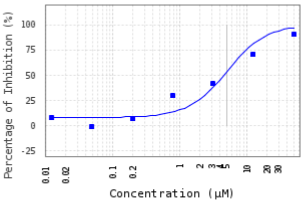

IC50: 5.1049 *(2.6592 - 9.7998)* pass
slope=1.500 min=8.321 max=100.000 err=502.914 R^2=0.9297

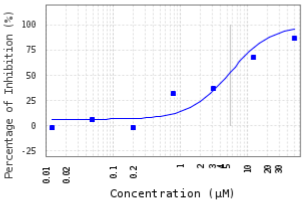

IC50: 5.6039 *(2.8002 - 11.215)* pass
slope=1.400 min=6.465 max=100.000 err=708.035 R^2=0.9025

BJ (IC_{50} = 8.35 ± 0.419 µM)

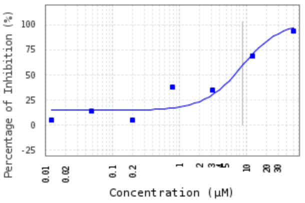

IC50: 8.6646 *(3.7555 - 19.991)* pass
slope=1.500 min=14.821 max=102.904 err=648.722 R^2=0.9031

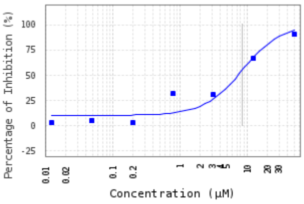

IC50: 8.5045 *(4.0882 - 17.692)* pass
slope=1.500 min=10.390 max=100.140 err=521.873 R^2=0.9260

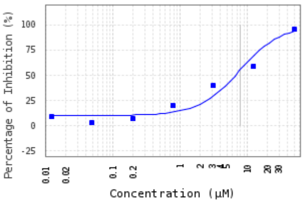

IC50: 7.8727 *(4.6975 - 13.194)* pass
slope=1.400 min=10.188 max=100.000 err=323.695 R^2=0.9535

CB2

CCRF-CEM (IC_{50} = 0.33 ± 0.005 µM)

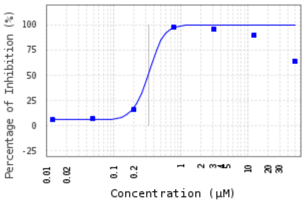

IC50: 0.3320 *(0.1563 - 0.7052)* pass
slope=3.800 min=5.932 max=100.000 err=1388.890 R^2=0.8742

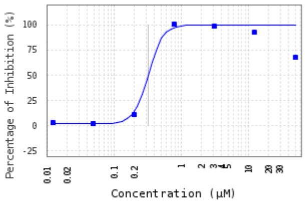

IC50: 0.3226 *(0.1726 - 0.6027)* pass
slope=4.000 min=1.932 max=100.000 err=1121.926 R^2=0.9128

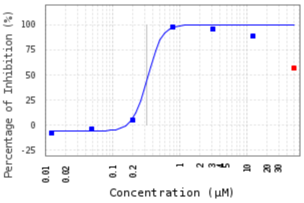

IC50: 0.3266 *(0.2529 - 0.4219)* pass
slope=4.000 min=-5.713 max=100.000 err=155.745 R^2=0.9888

CEM-DNR (IC_{50} = 1.45 ± 0.060 µM)

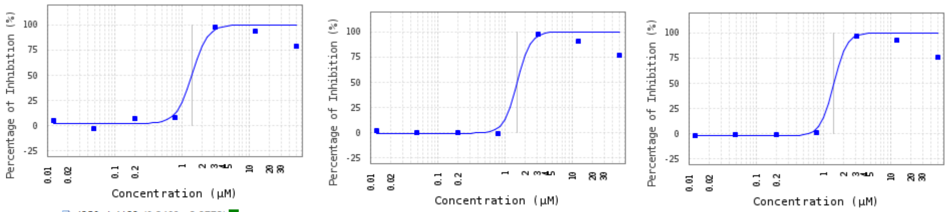

IC50: 1.4189 (0.8469 - 2.3772) pass
slope=3.800 min=2.404 max=100.000 err=565.400 R^2=0.9556

IC50: 1.5235 (0.7126 - 3.2573) pass
slope=4.500 min=-0.305 max=100.000 err=640.760 R^2=0.9523

IC50: 1.4187 (0.7389 - 2.7239) pass
slope=4.500 min=-1.993 max=100.000 err=649.221 R^2=0.9527

K562 (IC_{50} = 0.19 ± 0.040 µM)

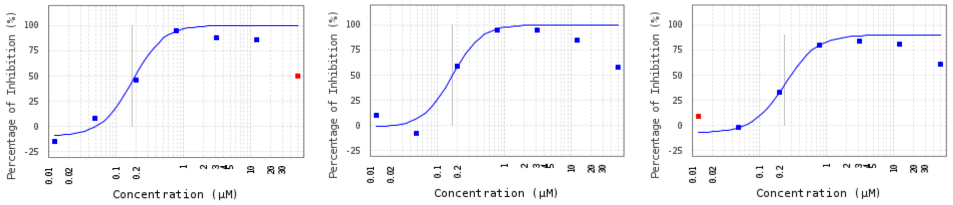

IC50: 0.1760 (0.1212 - 0.2556) pass
slope=1.900 min=-9.147 max=100.000 err=461.781 R^2=0.9567

IC50: 0.1665 (0.0784 - 0.3535) pass
slope=2.000 min=-1.512 max=100.000 err=2389.497 R^2=0.7597

IC50: 0.2395 (0.1263 - 0.4542) pass
slope=1.800 min=-7.215 max=90.000 err=956.376 R^2=0.8385

K562-TAX (IC_{50} = 0.58 ± 0.035 µM)

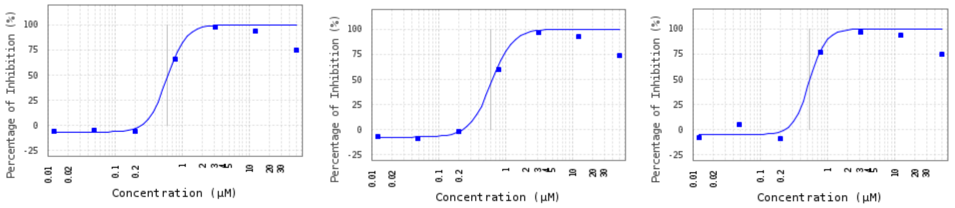

IC50: 0.5951 (0.4494 - 0.7882) pass
slope=3.000 min=-6.408 max=100.000 err=673.674 R^2=0.9519

IC50: 0.6064 (0.3847 - 0.9560) pass
slope=2.658 min=-7.234 max=100.000 err=774.044 R^2=0.9437

IC50: 0.5407 (0.3810 - 0.7673) pass
slope=3.500 min=-4.665 max=100.000 err=869.895 R^2=0.9383

HCT116 (IC_{50} > 50 µM)

Primary screen (50 µM)

1 (Plate 1)	K9	LEM00014574	47.737		50.0uM	+	✓ ⊗
1 (Plate 1)	K10	LEM00014574	38.755		50.0uM	+	
1 (Plate 1)	K11	LEM00014574	34.265		50.0uM	+	

1 (Plate 1)	K9	LEM00014574	48.921		50.0uM	+	✓ ⊗
1 (Plate 1)	K10	LEM00014574	48.003		50.0uM	+	
1 (Plate 1)	K11	LEM00014574	28.178		50.0uM	+	

HCT116p53-/- (IC_{50} > 50 µM)

Primary screen (50 µM)

1 (Plate 1)	K9	LEM00014574	51.919		50.0uM	+	✓ ⊗
1 (Plate 1)	K10	LEM00014574	49.794		50.0uM	+	
1 (Plate 1)	K11	LEM00014574	42.529		50.0uM	+	

1 (Plate 1)	K9	LEM00014574	37.892		50.0uM	+	✓ ⊗
1 (Plate 1)	K10	LEM00014574	37.825		50.0uM	+	
1 (Plate 1)	K11	LEM00014574	8.2055		50.0uM	+	

U2OS (IC_{50} > 50 µM)

Primary screen (50 µM)

1 (Plate 1)	K9	LEM00014574	53.835		50.0uM	+	✓ ⊗
1 (Plate 1)	K10	LEM00014574	46.627		50.0uM	+	
1 (Plate 1)	K11	LEM00014574	50.443		50.0uM	+	

1 (Plate 1)	K9	LEM00014574	46.021		50.0uM	+	✓ ⊗
1 (Plate 1)	K10	LEM00014574	41.104		50.0uM	+	
1 (Plate 1)	K11	LEM00014574	47.509		50.0uM	+	

MRC5 (IC_{50} > 50 µM)

Primary screen (50 µM)

1 (Plate 1)	K9	LEM00014574	1.4143	●	50.0uM	+	✓ ⊗
1 (Plate 1)	K10	LEM00014574	2.1808	●	50.0uM	+	
1 (Plate 1)	K11	LEM00014574	4.8634	●	50.0uM	+	

1 (Plate 1)	K9	LEM00014574	8.269	●	50.0uM	+	✓ ⊗
1 (Plate 1)	K10	LEM00014574	0.1735	●	50.0uM	+	
1 (Plate 1)	K11	LEM00014574	4.3947	●	50.0uM	+	

BJ (IC_{50} > 50 µM)

Primary screen (50 µM)

1 (Plate 1)	K9	LEM00014574	19.382	●	50.0uM	+	✓ ⊗
1 (Plate 1)	K10	LEM00014574	10.546	●	50.0uM	+	
1 (Plate 1)	K11	LEM00014574	13.448	●	50.0uM	+	

1 (Plate 1)	K9	LEM00014574	22.199	●	50.0uM	+	✓ ⊗
1 (Plate 1)	K10	LEM00014574	19.106	●	50.0uM	+	
1 (Plate 1)	K11	LEM00014574	13.66	●	50.0uM	+	

CB3

CCRF-CEM (IC_{50} = 9.33 ± 0.952 µM)

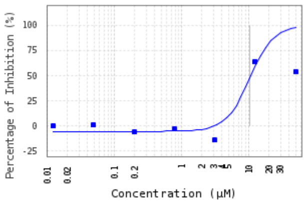

IC50: 10.411 (5.1577 - 21.015) pass
slope=2.416 min=-5.252 max=100.000 err=2231.521 R^2=0.6242

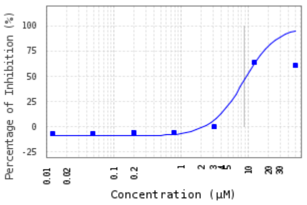

IC50: 8.6136 (4.0606 - 18.271) pass
slope=1.735 min=-9.088 max=100.000 err=1245.700 R^2=0.8090

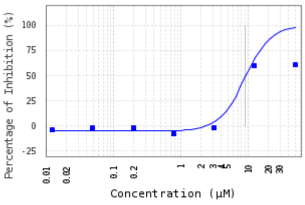

IC50: 8.9681 (4.5887 - 17.527) pass
slope=2.217 min=-5.136 max=100.000 err=1479.484 R^2=0.7438

CEM-DNR (IC_{50} > 50 µM)

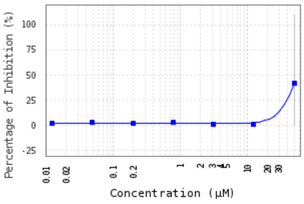

IC50: >50 pass
slope=3.000 min=1.920 max=110.000 err=5.303 R^2=0.9962

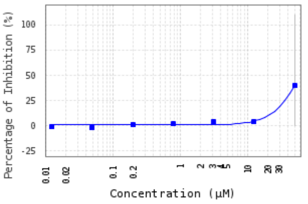

IC50: >50 pass
slope=2.027 min=1.100 max=110.000 err=20.250 R^2=0.9841

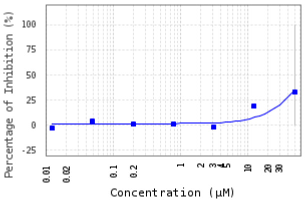

IC50: >50 pass
slope=1.500 min=1.677 max=100.038 err=179.266 R^2=0.8345

K562 (IC_{50} = 9.50 ± 2.37 µM)

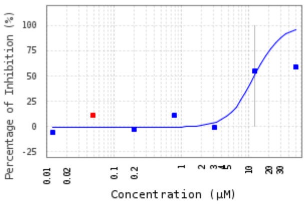

IC50: 12.221 (6.1906 - 24.124) pass
slope=2.200 min=-0.636 max=100.000 err=1523.299 R^2=0.6587

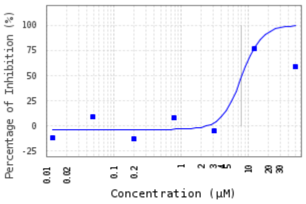

IC50: 7.9405 (3.2742 - 19.257) pass
slope=2.827 min=-3.340 max=100.000 err=2134.557 R^2=0.7225

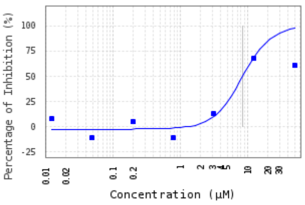

IC50: 8.3242 (3.9289 - 17.637) pass
slope=2.000 min=-2.242 max=100.000 err=1629.644 R^2=0.7419

K562-TAX (IC$_{50}$ = 40.11 ± 1.075 µM)

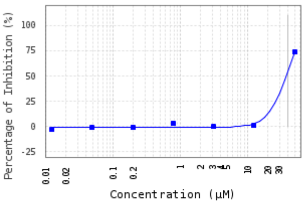

IC50: 39.413 *(21.962 - 70.729)* pass
slope=3.000 min=-0.508 max=110.000 err=27.993 R^2=0.9940

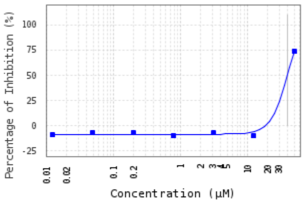

IC50: 39.58 *(20.276 - 77.264)* pass
slope=3.500 min=-8.201 max=110.000 err=20.210 R^2=0.9965

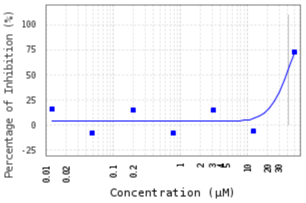

IC50: 41.353 *(0.7606 - 2248.3)* pass
slope=3.000 min=3.873 max=110.000 err=833.485 R^2=0.8276

HCT116 (IC$_{50}$ = 31.61 ± 4.544 µM)

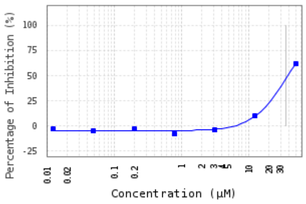

IC50: 36.269 *(5.4075 - 243.26)* pass
slope=1.738 min=-4.631 max=100.000 err=13.091 R^2=0.9964

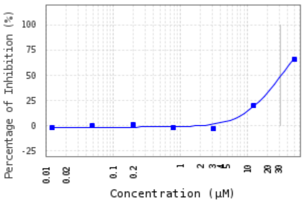

IC50: 31.356 *(6.6821 - 147.14)* pass
slope=1.506 min=-1.253 max=100.000 err=28.953 R^2=0.9924

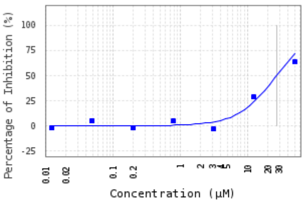

IC50: 27.191 *(1.7103 - 432.3)* pass
slope=1.500 min=0.369 max=100.000 err=184.158 R^2=0.9485

HCT116p53-/- (IC$_{50}$ = 36.36 ± 5.031 µM)

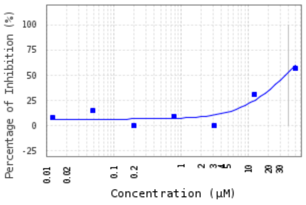

IC50: 40.128 *(9.0862 - 177.22)* pass
slope=1.200 min=6.631 max=100.000 err=271.995 R^2=0.8910

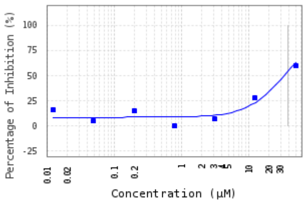

IC50: 38.316 *(0.0119 - 123416)* pass
slope=1.500 min=8.724 max=100.000 err=238.303 R^2=0.9027

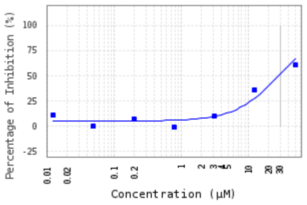

IC50: 30.65 *(12.386 - 75.843)* pass
slope=1.300 min=5.285 max=100.000 err=224.970 R^2=0.9255

U2OS (IC$_{50}$ > 50 µM)

Primary screen (50 µM)

1 (Plate 1)	J12	LEM00014587	44.145	●	50.0uM	+	✓ ⊗
1 (Plate 1)	J13	LEM00014587	22.275	●	50.0uM	+	
1 (Plate 1)	J14	LEM00014587	51.068	●	50.0uM	+	

1 (Plate 1)	J12	LEM00014587	46.91	●	50.0uM	+	✓ ⊗
1 (Plate 1)	J13	LEM00014587	49.029	●	50.0uM	+	
1 (Plate 1)	J14	LEM00014587	21.046	●	50.0uM	+	

MRC5 (IC$_{50}$ > 50 µM)

Primary screen (50 µM)

1 (Plate 1)	J12	LEM00014587	6.3963	●	50.0uM	+	✓ ⊗
1 (Plate 1)	J13	LEM00014587	-9.38	●	50.0uM	+	
1 (Plate 1)	J14	LEM00014587	-1.5238	●	50.0uM	+	

1 (Plate 1)	J12	LEM00014587	12.085	●	50.0uM	+	✓ ⊗
1 (Plate 1)	J13	LEM00014587	-2.9491	●	50.0uM	+	
1 (Plate 1)	J14	LEM00014587	11.218	●	50.0uM	+	

BJ (IC$_{50}$ > 50 µM)

Primary screen (50 µM)

1 (Plate 1)	J12	LEM00014587	17.769	●	50.0uM	+	✓ ⊗
1 (Plate 1)	J13	LEM00014587	16.737	●	50.0uM	+	
1 (Plate 1)	J14	LEM00014587	16.415	●	50.0uM	+	

1 (Plate 1)	J12	LEM00014587	19.98	●	50.0uM	+	✓ ⊗
1 (Plate 1)	J13	LEM00014587	11.172	●	50.0uM	+	
1 (Plate 1)	J14	LEM00014587	19.98	●	50.0uM	+	

CB4

CCRF-CEM (IC_{50} = 0.020 ± 0.0009 μM)

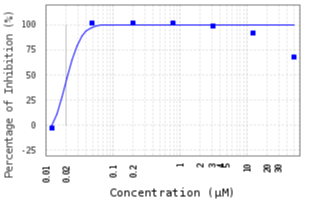

IC50: 0.0196 (0.0016 - 0.2365) pass
slope=4.000 min=-15.000 max=100.000 err=1121.243 R^2=0.8745

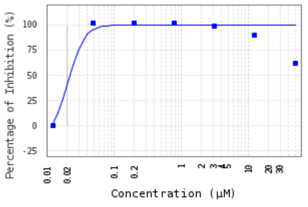

IC50: 0.0200 (0.0025 - 0.1609) pass
slope=3.500 min=-15.000 max=100.000 err=1576.787 R^2=0.8148

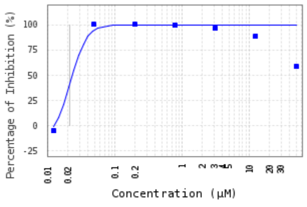

IC50: 0.0213 (0.0036 - 0.1283) pass
slope=3.500 min=-14.879 max=100.000 err=1855.184 R^2=0.7961

CEM-DNR (IC_{50} = 0.36 ± 0.014 μM)

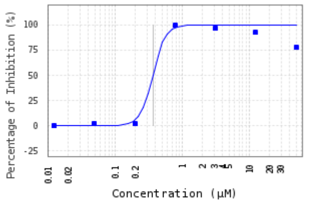

IC50: 0.3631 (0.1827 - 0.7217) pass
slope=4.500 min=0.030 max=100.000 err=579.915 R^2=0.9588

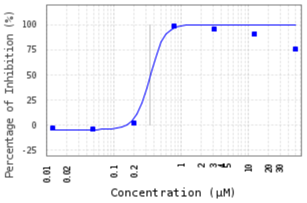

IC50: 0.3401 (0.1998 - 0.5789) pass
slope=4.000 min=-4.319 max=100.000 err=698.023 R^2=0.9527

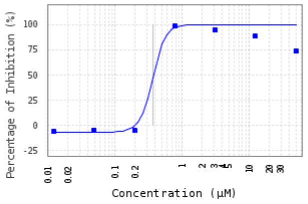

IC50: 0.3645 (0.1647 - 0.8066) pass
slope=4.500 min=-6.396 max=100.000 err=864.542 R^2=0.9438

K562 (IC_{50} = 0.088 ± 0.013 μM)

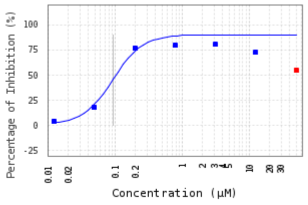

IC50: 0.0931 (0.0571 - 0.1517) pass
slope=2.000 min=1.444 max=90.000 err=488.631 R^2=0.9182

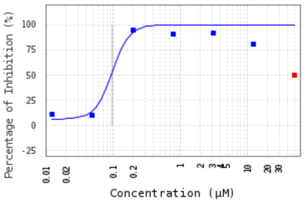

IC50: 0.0977 (0.0500 - 0.1910) pass
slope=3.500 min=6.526 max=100.000 err=577.165 R^2=0.9311

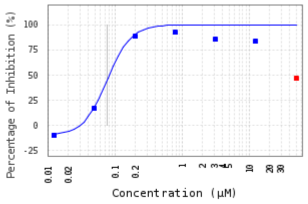

IC50: 0.0767 (0.0480 - 0.1227) pass
slope=2.488 min=-9.337 max=100.000 err=518.969 R^2=0.9466

K562-TAX (IC_{50} = 0.19 ± 0.021 μM)

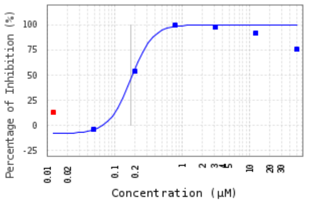

IC50: 0.1724 (0.1240 - 0.2397) pass
slope=2.700 min=-7.790 max=100.000 err=663.134 R^2=0.9154

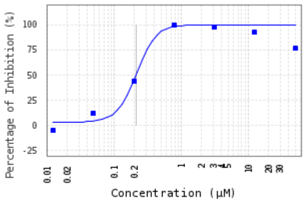

IC50: 0.2133 (0.1601 - 0.2841) pass
slope=3.000 min=3.164 max=100.000 err=757.649 R^2=0.9313

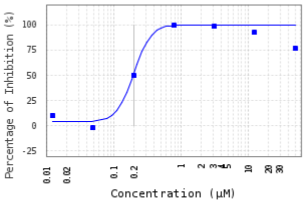

IC50: 0.1971 (0.1575 - 0.2467) pass
slope=3.500 min=3.919 max=100.000 err=664.709 R^2=0.9381

HCT116 (IC_{50} = 0.10 ± 0.004 μM)

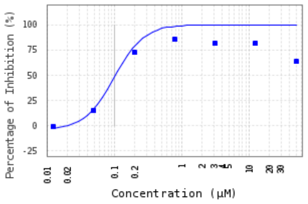

IC50: 0.0994 (0.0413 - 0.2393) pass
slope=2.000 min=-3.926 max=100.000 err=2201.744 R^2=0.7005

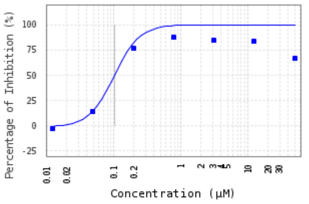

IC50: 0.1021 (0.0425 - 0.2451) pass
slope=2.328 min=-1.025 max=100.000 err=1790.174 R^2=0.7823

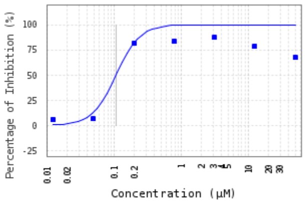

IC50: 0.1070 (0.0484 - 0.2365) pass
slope=2.500 min=0.637 max=100.000 err=1913.405 R^2=0.7575

HCT116p53-/- (IC_{50} = 0.12 ± 0.012 µM)

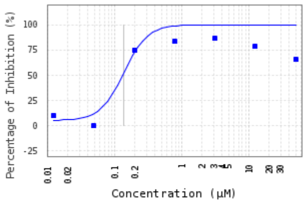

IC50: 0.1368 (0.0676 - 0.2770) pass
slope=2.500 min=5.312 max=100.000 err=2180.658 R^2=0.7222

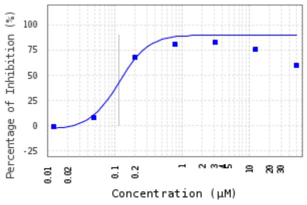

IC50: 0.1163 (0.0639 - 0.2120) pass
slope=2.000 min=-3.204 max=90.000 err=1195.863 R^2=0.8367

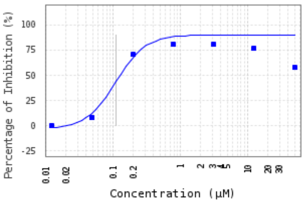

IC50: 0.1109 (0.0578 - 0.2129) pass
slope=2.000 min=-2.934 max=90.000 err=1343.031 R^2=0.8137

U2OS (IC_{50} = 0.14 ± 0.004 µM)

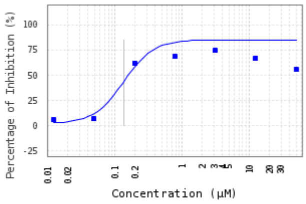

IC50: 0.1352 (0.0669 - 0.2735) pass
slope=2.000 min=2.381 max=85.000 err=1500.686 R^2=0.7093

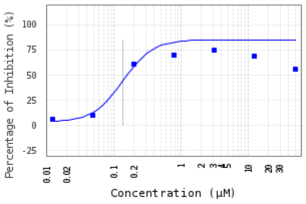

IC50: 0.1366 (0.0685 - 0.2725) pass
slope=2.000 min=3.754 max=85.000 err=1408.136 R^2=0.7198

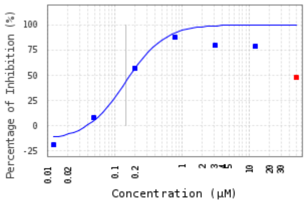

IC50: 0.1440 (0.0660 - 0.3141) pass
slope=1.500 min=-13.646 max=100.000 err=905.154 R^2=0.9070

MRC5 (IC_{50} > 50 µM)

Primary screen (50 µM)

1 (Plate 1)	M9	LEM00014576	5.8853		50.0uM	+	✓ ⊗
1 (Plate 1)	M10	LEM00014576	14.636		50.0uM	+	
1 (Plate 1)	M11	LEM00014576	12.145		50.0uM	+	

1 (Plate 1)	M9	LEM00014576	4.2212		50.0uM	+	✓ ⊗
1 (Plate 1)	M10	LEM00014576	11.97		50.0uM	+	
1 (Plate 1)	M11	LEM00014576	8.7894		50.0uM	+	

BJ (IC_{50} > 50 µM)

Primary screen (50 µM)

1 (Plate 1)	M9	LEM00014576	20.349		50.0uM	+	✓ ⊗
1 (Plate 1)	M10	LEM00014576	23.252		50.0uM	+	
1 (Plate 1)	M11	LEM00014576	17.834		50.0uM	+	

1 (Plate 1)	M9	LEM00014576	23.611		50.0uM	+	✓ ⊗
1 (Plate 1)	M10	LEM00014576	22.603		50.0uM	+	
1 (Plate 1)	M11	LEM00014576	24.418		50.0uM	+	

CB5

CCRF-CEM (IC_{50} = 0.42 ± 0.014 µM)

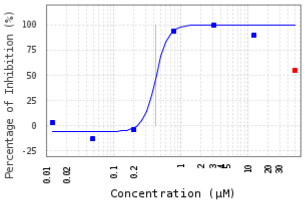

IC50: 0.4229 (0.2616 - 0.6836) pass
slope=4.500 min=-5.523 max=100.000 err=244.060 R^2=0.9836

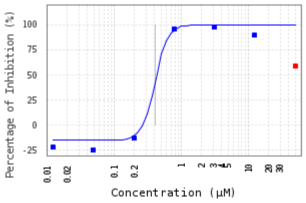

IC50: 0.4030 (0.2484 - 0.6539) pass
slope=4.500 min=-15.000 max=100.000 err=249.204 R^2=0.9871

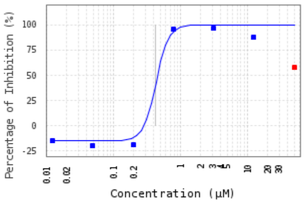

IC50: 0.4291 (0.2820 - 0.6531) pass
slope=4.500 min=-15.000 max=100.000 err=238.352 R^2=0.9871

CEM-DNR (IC$_{50}$ = 1.39 ± 0.139 µM)

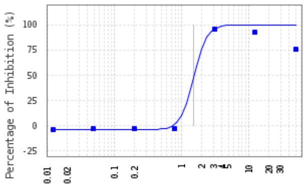

IC50: 1.5197 *(0.7259 - 3.1812)* pass
slope=4.500 min=-3.931 max=100.000 err=653.780 R^2=0.9548

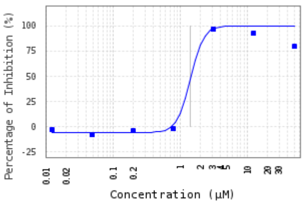

IC50: 1.4201 *(0.8170 - 2.4684)* pass
slope=4.500 min=-5.459 max=100.000 err=495.878 R^2=0.9674

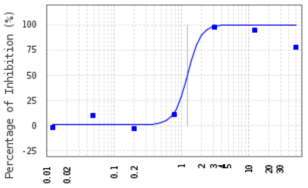

IC50: 1.2443 *(0.8042 - 1.9252)* pass
slope=4.500 min=1.363 max=100.000 err=645.146 R^2=0.9503

K562 (IC$_{50}$ = 0.50 ± 0.122 µM)

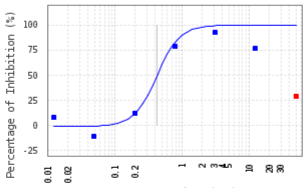

IC50: 0.4251 *(0.2260 - 0.7996)* pass
slope=2.502 min=-0.868 max=100.000 err=796.731 R^2=0.9197

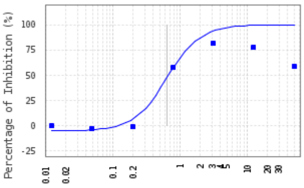

IC50: 0.6427 *(0.2880 - 1.4342)* pass
slope=1.750 min=-5.036 max=100.000 err=2340.001 R^2=0.7369

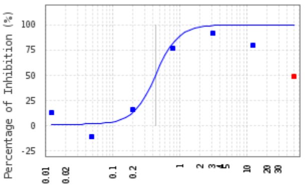

IC50: 0.4396 *(0.2471 - 0.7818)* pass
slope=2.500 min=1.627 max=100.000 err=790.754 R^2=0.9151

K562-TAX (IC$_{50}$ = 0.90 ± 0.026 µM)

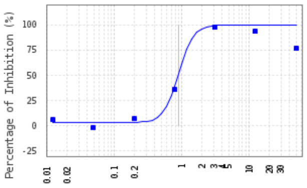

IC50: 0.9124 *(0.7422 - 1.1217)* pass
slope=4.000 min=3.376 max=100.000 err=625.795 R^2=0.9452

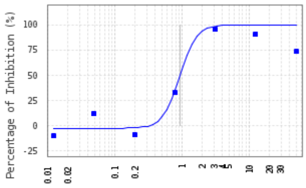

IC50: 0.9259 *(0.6879 - 1.2462)* pass
slope=3.500 min=-2.321 max=100.000 err=1121.837 R^2=0.9100

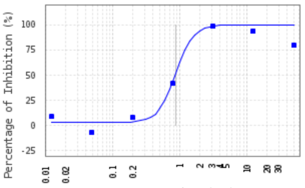

IC50: 0.8748 *(0.6959 - 1.0997)* pass
slope=3.300 min=3.149 max=100.000 err=589.102 R^2=0.9499

HCT116 (IC$_{50}$ = 0.64 ± 0.065 µM)

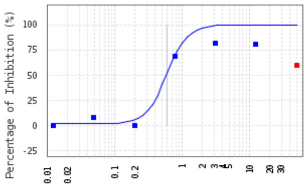

IC50: 0.5955 *(0.3997 - 0.8871)* pass
slope=2.800 min=1.807 max=100.000 err=754.006 R^2=0.9092

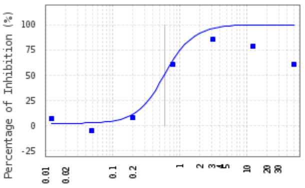

IC50: 0.6038 *(0.2607 - 1.3985)* pass
slope=2.017 min=2.378 max=100.000 err=2175.509 R^2=0.7424

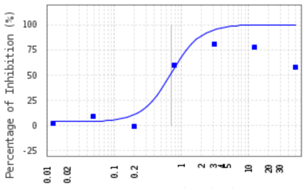

IC50: 0.7121 *(0.3208 - 1.5807)* pass
slope=2.000 min=3.828 max=100.000 err=2592.633 R^2=0.6721

HCT116p53-/- (IC$_{50}$ = 0.77 ± 0.063 µM)

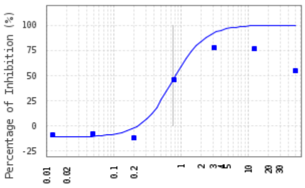

IC50: 0.7507 *(0.3259 - 1.7294)* pass
slope=1.843 min=-10.814 max=100.000 err=2856.166 R^2=0.7149

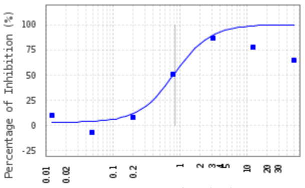

IC50: 0.8380 *(0.3522 - 1.994)* pass
slope=1.632 min=3.282 max=100.000 err=1839.769 R^2=0.7816

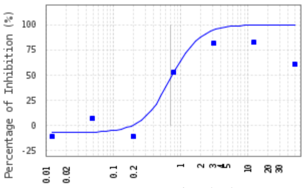

IC50: 0.7147 *(0.3644 - 1.4015)* pass
slope=2.000 min=-6.919 max=100.000 err=2288.313 R^2=0.7775

U2OS (IC_{50} = 1.04 ± 0.141 µM)

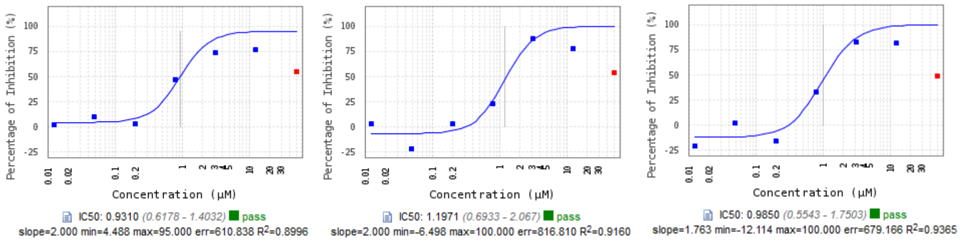

MRC5 (IC_{50} > 50 µM)

Primary screen (50 µM)

1 (Plate 1)	N9	LEM00014577	10.101		50.0uM	+	✓	⊗
1 (Plate 1)	N10	LEM00014577	0.7756		50.0uM	+		
1 (Plate 1)	N11	LEM00014577	0.5201		50.0uM	+		
1 (Plate 1)	N9	LEM00014577	6.939		50.0uM	+	✓	⊗
1 (Plate 1)	N10	LEM00014577	2.313		50.0uM	+		
1 (Plate 1)	N11	LEM00014577	4.626		50.0uM	+		

BJ (IC_{50} > 50 µM)

Primary screen (50 µM)

1 (Plate 1)	N9	LEM00014577	17.447		50.0uM	+	✓	⊗
1 (Plate 1)	N10	LEM00014577	16.544		50.0uM	+		
1 (Plate 1)	N11	LEM00014577	17.124		50.0uM	+		
1 (Plate 1)	N9	LEM00014577	14.534		50.0uM	+	✓	⊗
1 (Plate 1)	N10	LEM00014577	20.115		50.0uM	+		
1 (Plate 1)	N11	LEM00014577	18.837		50.0uM	+		

CB6

CCRF-CEM (IC_{50} = 0.14 ± 0.010 µM)

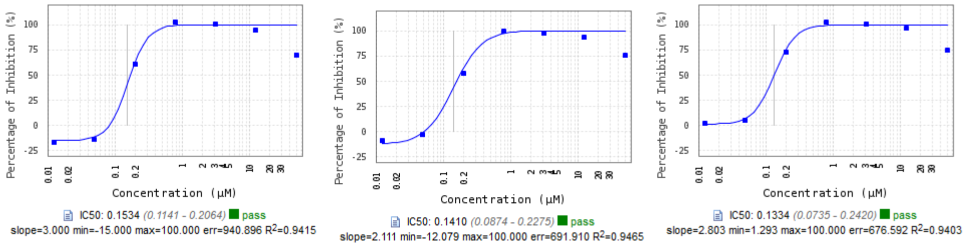

CEM-DNR (IC_{50} = 0.50 ± 0.037 µM)

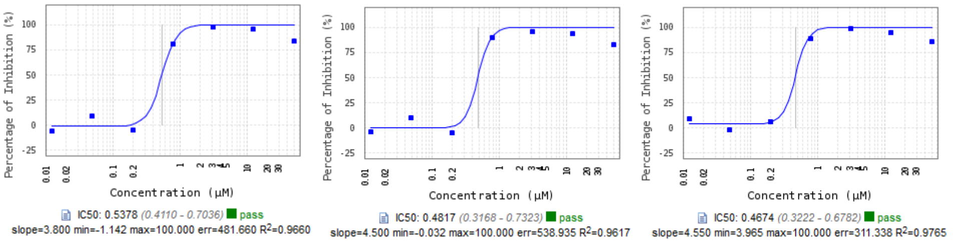

K562 (IC_{50} = 0.026 ± 0.001 μM)

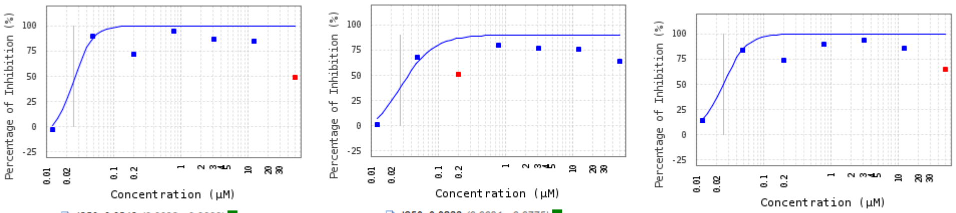

IC50: 0.0249 (0.0093 - 0.0666) pass
slope=3.000 min=-9.850 max=100.000 err=1256.247 R^2=0.8147

IC50: 0.0266 (0.0091 - 0.0775) pass
slope=1.700 min=-15.000 max=90.000 err=1265.628 R^2=0.7166

IC50: 0.0250 (0.0094 - 0.0666) pass
slope=2.500 min=1.258 max=100.000 err=1030.588 R^2=0.7649

K562-TAX (IC_{50} = 0.38 ± 0.034 μM)

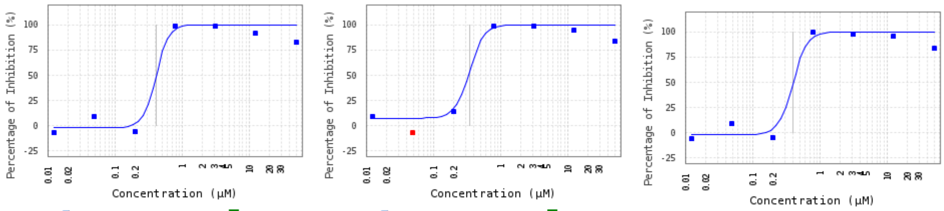

IC50: 0.4057 (0.2009 - 0.8193) pass
slope=4.500 min=-1.986 max=100.000 err=584.706 R^2=0.9621

IC50: 0.3412 (0.2069 - 0.5625) pass
slope=4.000 min=7.591 max=100.000 err=296.794 R^2=0.9676

IC50: 0.3942 (0.2245 - 0.6924) pass
slope=4.000 min=-1.924 max=100.000 err=520.547 R^2=0.9666

HCT116 (IC_{50} = 0.43 ± 0.0325 μM)

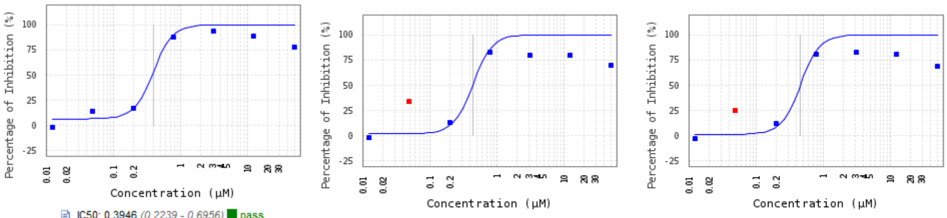

IC50: 0.3946 (0.2239 - 0.6956) pass
slope=3.000 min=6.657 max=100.000 err=779.131 R^2=0.9261

IC50: 0.4389 (0.1809 - 1.0647) pass
slope=3.000 min=2.148 max=100.000 err=1743.751 R^2=0.7594

IC50: 0.4576 (0.2031 - 1.0311) pass
slope=3.000 min=1.471 max=100.000 err=1669.267 R^2=0.7755

HCT116p53-/- (IC_{50} = 0.38 ± 0.057 μM)

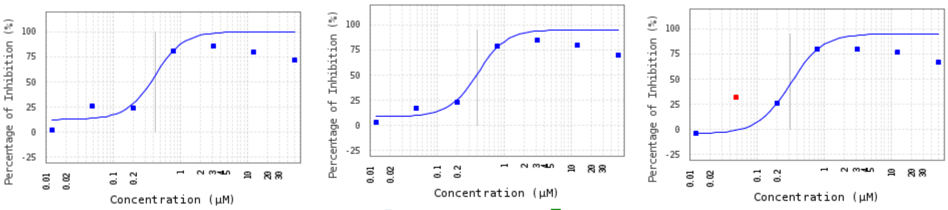

IC50: 0.4169 (0.1798 - 0.9662) pass
slope=2.000 min=12.608 max=100.000 err=1664.633 R^2=0.7599

IC50: 0.4018 (0.2244 - 0.7195) pass
slope=2.000 min=8.867 max=95.000 err=1024.370 R^2=0.8605

IC50: 0.3110 (0.1577 - 0.6136) pass
slope=1.800 min=-4.069 max=95.000 err=1276.819 R^2=0.7942

U2OS (IC_{50} = 0.48 ± 0.058 μM)

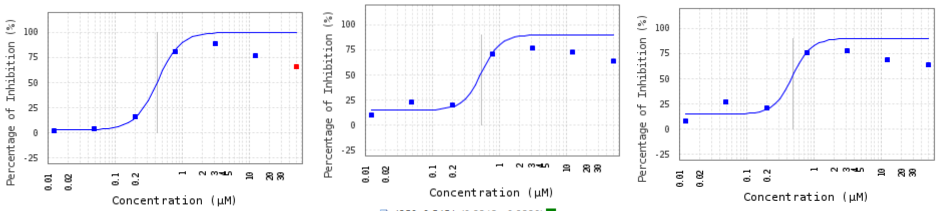

IC50: 0.4287 (0.2353 - 0.7814) pass
slope=2.482 min=3.058 max=100.000 err=666.158 R^2=0.9212

IC50: 0.5451 (0.3218 - 0.9236) pass
slope=3.000 min=15.000 max=90.000 err=1243.653 R^2=0.7518

IC50: 0.4800 (0.2416 - 0.9536) pass
slope=3.000 min=15.000 max=90.000 err=1469.433 R^2=0.7102

MRC5 (IC_{50} > 50 μM)

Primary screen (50 μM)

1 (Plate 1)	O9	LEM00014578	15.338		50.0uM	+	✓ ⊗
1 (Plate 1)	O10	LEM00014578	20.576		50.0uM	+	
1 (Plate 1)	O11	LEM00014578	14.827		50.0uM	+	

1 (Plate 1)	O9	LEM00014578	12.432		50.0uM	+	✓ ⊗
1 (Plate 1)	O10	LEM00014578	18.62		50.0uM	+	
1 (Plate 1)	O11	LEM00014578	20.47		50.0uM	+	

BJ (IC_{50} > 50 μM)

Primary screen (50 μM)

1 (Plate 1)	O9	LEM00014578	19.317		50.0uM	+	✓ ⊗
1 (Plate 1)	O10	LEM00014578	14.222		50.0uM	+	
1 (Plate 1)	O11	LEM00014578	9.6425		50.0uM	+	

1 (Plate 1)	O9	LEM00014578	25.696		50.0uM	+	✓ ⊗
1 (Plate 1)	O10	LEM00014578	19.644		50.0uM	+	
1 (Plate 1)	O11	LEM00014578	21.998		50.0uM	+	

CB7

CCRF-CEM (IC_{50} = 0.84 ± 0.058 μM)

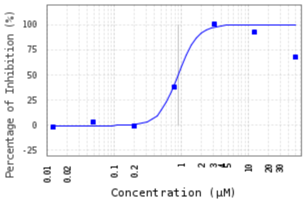

IC50: 0.9021 (0.6431 - 1.2653) pass
slope=3.000 min=-0.357 max=100.000 err=1105.046 R^2=0.9071

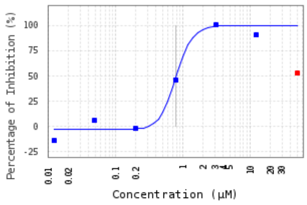

IC50: 0.7956 (0.6612 - 0.9573) pass
slope=3.551 min=-3.170 max=100.000 err=286.459 R^2=0.9760

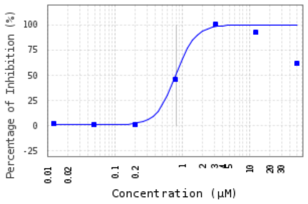

IC50: 0.8110 (0.5501 - 1.1956) pass
slope=3.000 min=1.057 max=100.000 err=1547.650 R^2=0.8613

CEM-DNR (IC_{50} = 1.34 ± 0.2228 μM)

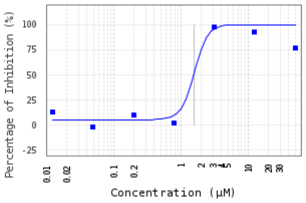

IC50: 1.5817 (0.6480 - 3.8606) pass
slope=4.500 min=5.554 max=100.000 err=758.251 R^2=0.9377

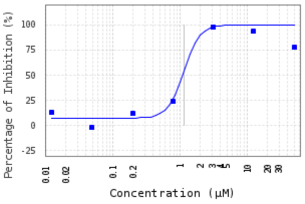

IC50: 1.1492 (0.7972 - 1.6566) pass
slope=3.700 min=7.444 max=100.000 err=700.944 R^2=0.9359

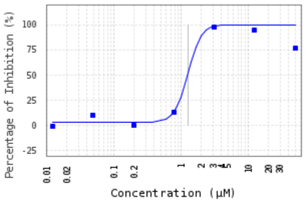

IC50: 1.2748 (0.7817 - 2.0789) pass
slope=4.500 min=3.417 max=100.000 err=656.321 R^2=0.9475

K562 (IC_{50} = 0.77 ± 0.224 μM)

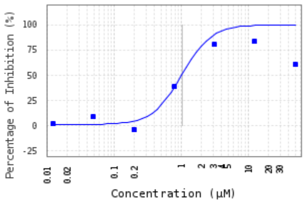

IC50: 1.0311 (0.5051 - 2.1048) pass
slope=2.000 min=1.347 max=100.000 err=1973.901 R^2=0.7609

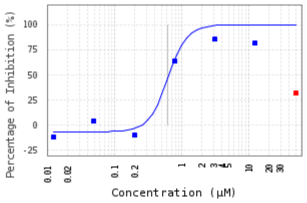

IC50: 0.6238 (0.4526 - 0.8599) pass
slope=3.000 min=-6.434 max=100.000 err=705.666 R^2=0.9337

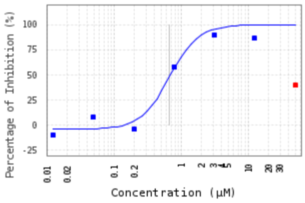

IC50: 0.6676 (0.4626 - 0.9636) pass
slope=2.000 min=-4.144 max=100.000 err=445.366 R^2=0.9568

K562-TAX (IC_{50} = 0.62 ± 0.084 µM)

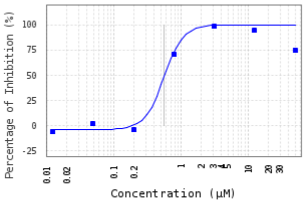

IC50: 0.5604 (0.4074 - 0.7708) pass
slope=3.000 min=-3.765 max=100.000 err=707.740 R^2=0.9479

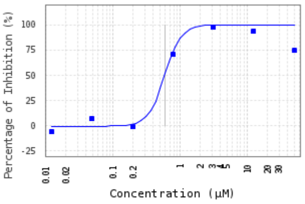

IC50: 0.5933 (0.4429 - 0.7948) pass
slope=3.500 min=-0.298 max=100.000 err=782.720 R^2=0.9381

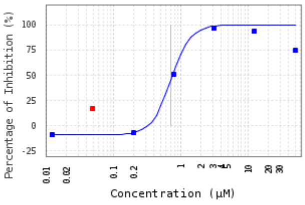

IC50: 0.7199 (0.5452 - 0.9507) pass
slope=3.000 min=-8.972 max=100.000 err=699.432 R^2=0.9385

HCT116 (IC_{50} = 1.51 ± 0.33 µM)

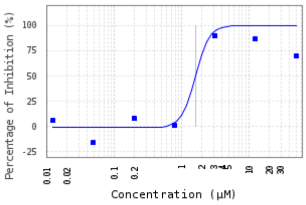

IC50: 1.6013 (0.5218 - 4.9137) pass
slope=4.251 min=-1.020 max=100.000 err=1478.661 R^2=0.8775

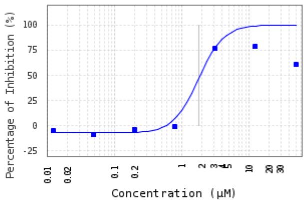

IC50: 1.7817 (0.8226 - 3.859) pass
slope=2.373 min=-6.979 max=100.000 err=1977.037 R^2=0.8075

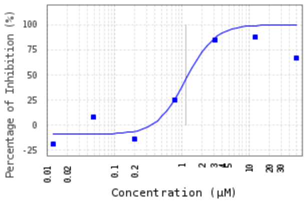

IC50: 1.1476 (0.6185 - 2.1291) pass
slope=2.000 min=-9.094 max=100.000 err=1658.443 R^2=0.8655

HCT116p53-/- (IC_{50} = 1.43 ± 0.162 µM)

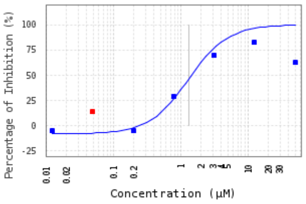

IC50: 1.3093 (0.5182 - 3.3079) pass
slope=1.500 min=-7.923 max=100.000 err=1634.315 R^2=0.7774

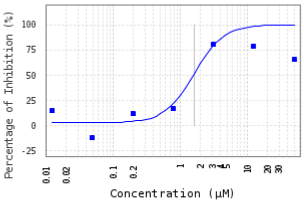

IC50: 1.6152 (0.6982 - 3.7366) pass
slope=2.000 min=3.058 max=100.000 err=2008.535 R^2=0.7575

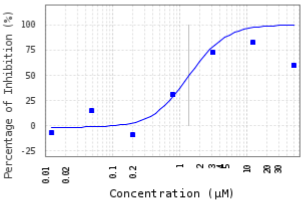

IC50: 1.3724 (0.5435 - 3.466) pass
slope=1.600 min=-1.519 max=100.000 err=2220.962 R^2=0.7350

U2OS (IC_{50} = 1.96 ± 0.096 µM)

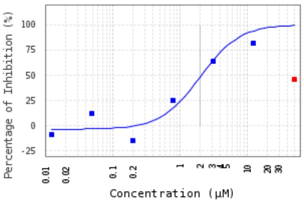

IC50: 1.9851 (0.8668 - 4.5461) pass
slope=1.500 min=-3.466 max=100.000 err=676.112 R^2=0.9107

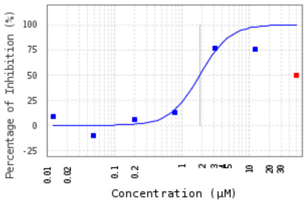

IC50: 1.8527 (1.0105 - 3.3967) pass
slope=2.000 min=0.517 max=100.000 err=709.837 R^2=0.8999

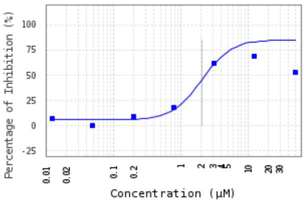

IC50: 2.0388 (1.0905 - 3.8115) pass
slope=2.000 min=5.799 max=85.000 err=1228.848 R^2=0.7567

MRC5 (IC_{50} > 50 µM)

Primary screen (50 µM)

1 (Plate 1)	B12	LEM00014579	-3.4399	●	50.0uM	+	✓ ⊗
1 (Plate 1)	B13	LEM00014579	-9.6355	●	50.0uM	+	
1 (Plate 1)	B14	LEM00014579	-14.298	●	50.0uM	+	

1 (Plate 1)	B12	LEM00014579	12.085	●	50.0uM	+	✓ ⊗
1 (Plate 1)	B13	LEM00014579	14.167	●	50.0uM	+	
1 (Plate 1)	B14	LEM00014579	0.4626	●	50.0uM	+	

BJ (IC_{50} > 50 µM)

Primary screen (50 µM)

1 (Plate 1)	B12	LEM00014579	22.607		50.0uM	+	✓	⊗
1 (Plate 1)	B13	LEM00014579	20.156		50.0uM	+		
1 (Plate 1)	B14	LEM00014579	21.446		50.0uM	+		

1 (Plate 1)	B12	LEM00014579	26.032		50.0uM	+	✓	⊗
1 (Plate 1)	B13	LEM00014579	18.972		50.0uM	+		
1 (Plate 1)	B14	LEM00014579	19.241		50.0uM	+		

CB8

CCRF-CEM (IC_{50} = 7.11 ± 1.141 µM)

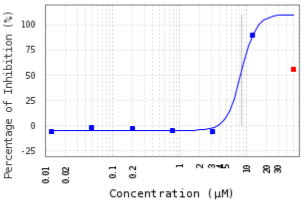

IC50: 8.4197 (4.7088 - 15.055) pass
slope=3.820 min=-4.496 max=110.000 err=28.606 R^2=0.9961

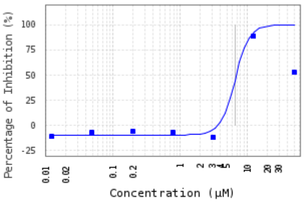

IC50: 6.5747 (2.0624 - 20.96) pass
slope=4.000 min=-9.311 max=100.000 err=2272.302 R^2=0.7642

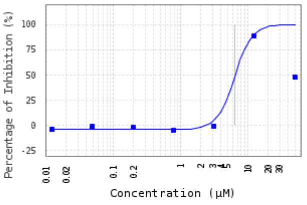

IC50: 6.3344 (2.1507 - 18.657) pass
slope=3.300 min=-4.016 max=100.000 err=2734.031 R^2=0.6567

CEM-DNR (IC_{50} = 6.53 ± 0.458 µM)

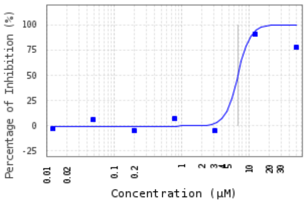

IC50: 6.8261 (3.0288 - 15.385) pass
slope=4.550 min=-0.213 max=100.000 err=690.740 R^2=0.9329

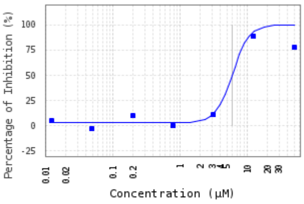

IC50: 6.0011 (3.5245 - 10.218) pass
slope=3.400 min=2.849 max=100.000 err=595.458 R^2=0.9337

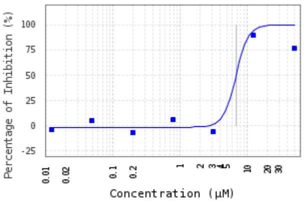

IC50: 6.7582 (2.964 - 15.409) pass
slope=4.500 min=-1.378 max=100.000 err=727.585 R^2=0.9296

K562 (IC_{50} = 7.83 ± 0.892 µM)

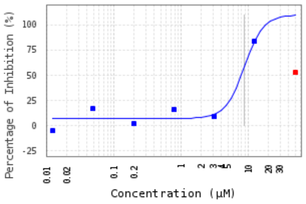

IC50: 8.8258 (1.6041 - 48.561) pass
slope=3.000 min=7.058 max=110.000 err=346.622 R^2=0.9322

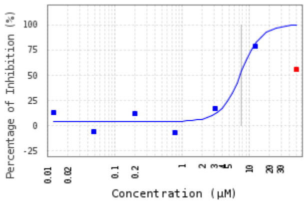

IC50: 7.5659 (2.5651 - 22.316) pass
slope=2.800 min=4.419 max=100.000 err=380.544 R^2=0.9216

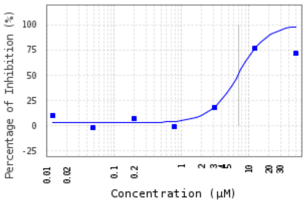

IC50: 7.1029 (3.9749 - 12.692) pass
slope=2.000 min=3.157 max=100.000 err=815.316 R^2=0.8809

K562-TAX (IC_{50} = 6.35 ± 0.335 µM)

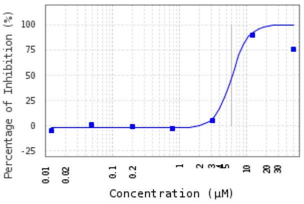

IC50: 6.007 (3.3216 - 10.863) pass
slope=3.462 min=-1.956 max=100.000 err=595.802 R^2=0.9409

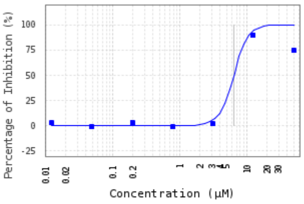

IC50: 6.3801 (3.1874 - 12.771) pass
slope=4.000 min=0.262 max=100.000 err=671.221 R^2=0.9298

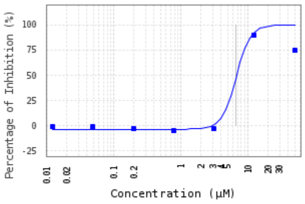

IC50: 6.6758 (3.4571 - 12.891) pass
slope=4.000 min=-3.335 max=100.000 err=668.783 R^2=0.9352

HCT116 (IC_{50} = 8.81 ± 1.937 µM)

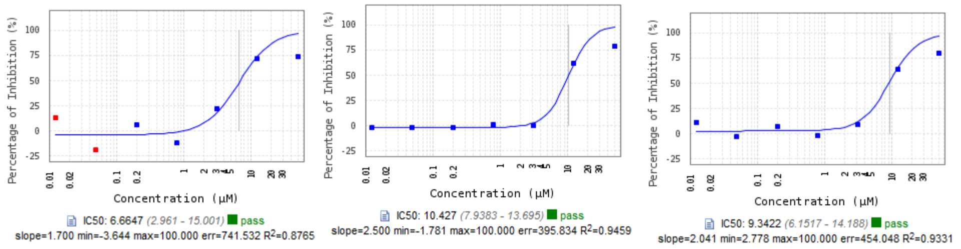

HCT116p53-/- (IC_{50} = 9.36 ± 0.870 µM)

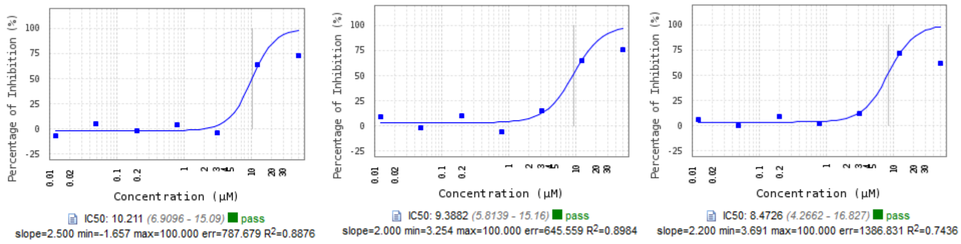

U2OS (IC_{50} = 11.88 ± 1.106 µM)

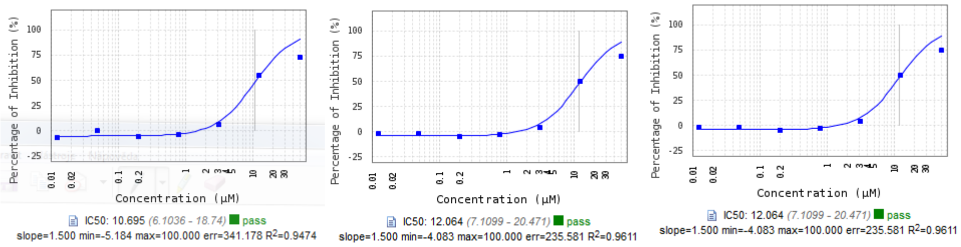

MRC5 (IC_{50} = 22.09 ± 1.223 µM)

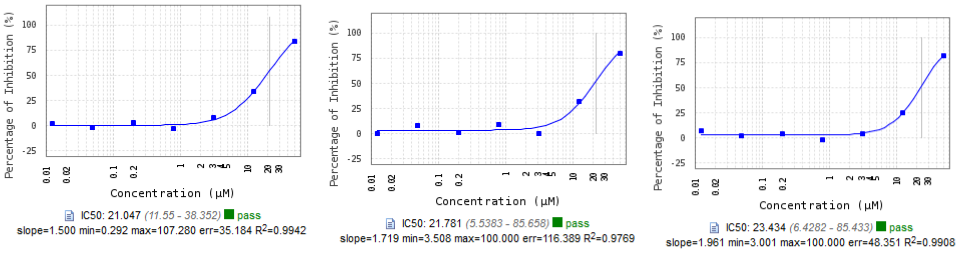

BJ (IC_{50} = 36.92 ± 5.680 µM)

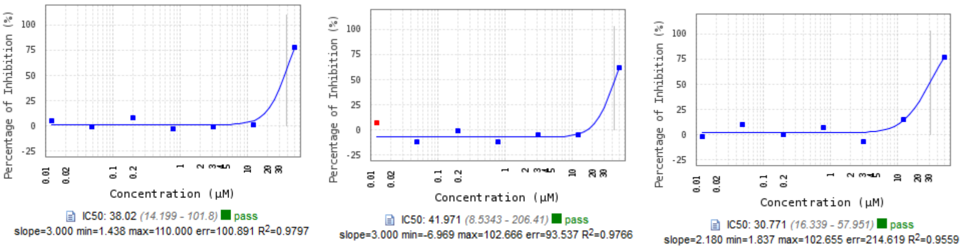

CB9

CCRF-CEM (IC_{50} = 4.81 ± 0.641 µM)

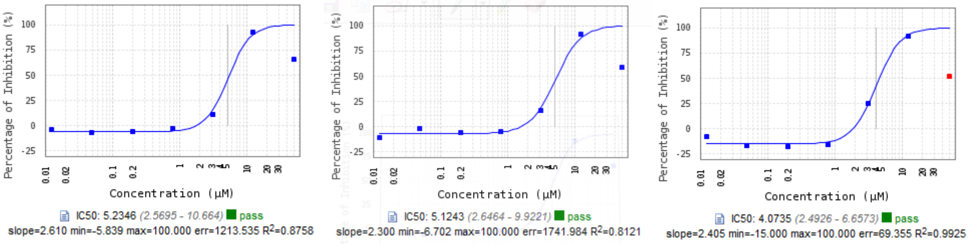

CEM-DNR (IC_{50} = 6.49 ± 0.639 µM)

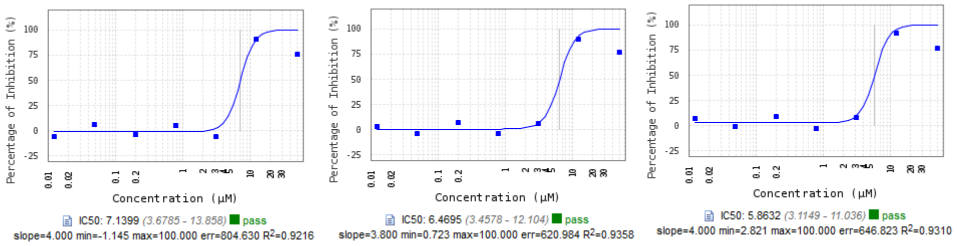

K562 (IC_{50} = 7.69 ± 0.450 µM)

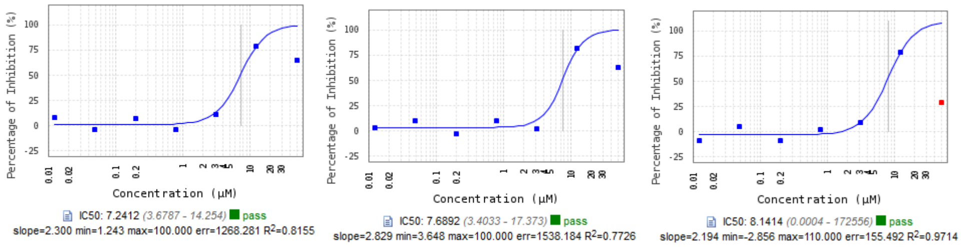

K562-TAX (IC_{50} = 5.78 ± 0.561 µM)

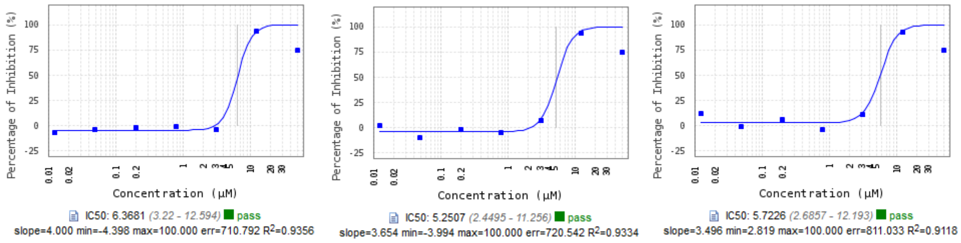

HCT116 (IC_{50} = 8.27± 1.432 µM)

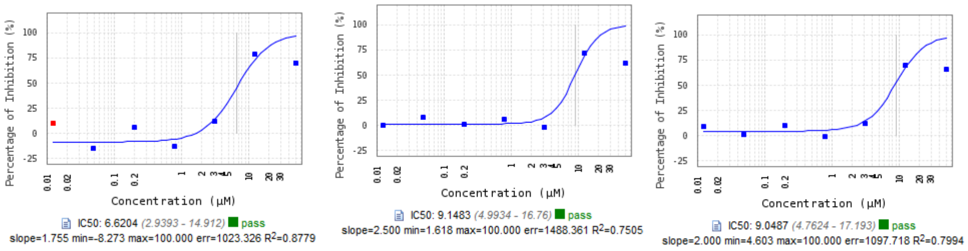

HCT116p53-/- (IC_{50} = 8.56 ± 0.365 μM)

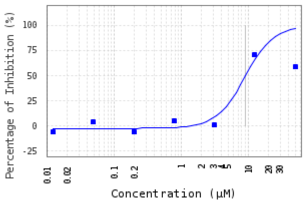

IC50: 8.9597 (4.2875 - 18.723) pass
slope=2.000 min=-2.253 max=100.000 err=1651.323 R^2=0.7348

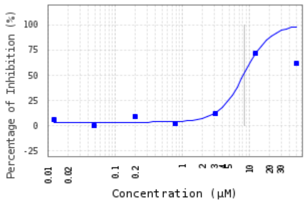

IC50: 8.4726 (4.2662 - 16.827) pass
slope=2.200 min=3.691 max=100.000 err=1386.831 R^2=0.7436

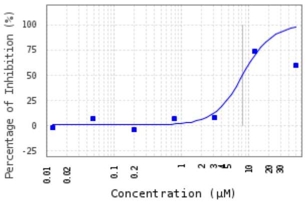

IC50: 8.2459 (3.8564 - 17.632) pass
slope=2.000 min=0.836 max=100.000 err=1561.315 R^2=0.7381

U2OS (IC_{50} > 50 μM)

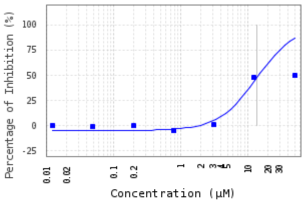

IC50: >50 pass
slope=1.500 min=-4.658 max=100.000 err=1474.139 R^2=0.5771

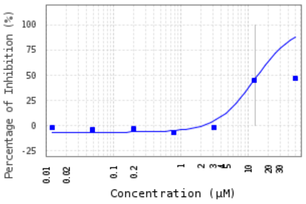

IC50: >50 pass
slope=1.500 min=-6.323 max=100.000 err=1710.564 R^2=0.5169

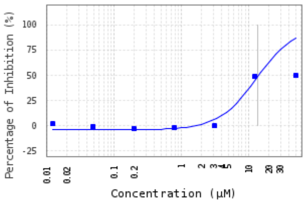

IC50: >50 pass
slope=1.500 min=-4.024 max=100.000 err=1505.152 R^2=0.5848

MRC5 (IC_{50} > 50 μM)

Primary screen (50 μM)

1 (Plate 1)	D12	LEM00014581	-9.4439	●	50.0uM	+	✓ ⊗
1 (Plate 1)	D13	LEM00014581	-6.1864	●	50.0uM	+	
1 (Plate 1)	D14	LEM00014581	-3.3122	●	50.0uM	+	

1 (Plate 1)	D12	LEM00014581	-1.7926	●	50.0uM	+	✓ ⊗
1 (Plate 1)	D13	LEM00014581	2.8912	●	50.0uM	+	
1 (Plate 1)	D14	LEM00014581	0.6361	●	50.0uM	+	

BJ (IC_{50} > 50 μM)

Primary screen (50 μM)

1 (Plate 1)	D12	LEM00014581	13.254	●	50.0uM	+	✓ ⊗
1 (Plate 1)	D13	LEM00014581	15.254	●	50.0uM	+	
1 (Plate 1)	D14	LEM00014581	13.061	●	50.0uM	+	

1 (Plate 1)	D12	LEM00014581	17.156	●	50.0uM	+	✓ ⊗
1 (Plate 1)	D13	LEM00014581	20.518	●	50.0uM	+	
1 (Plate 1)	D14	LEM00014581	13.794	●	50.0uM	+	

CB10

CCRF-CEM (IC_{50} = 7.11 ± 0.651 μM)

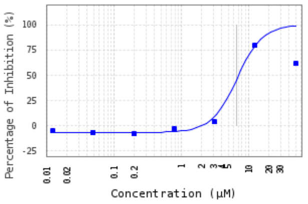

IC50: 6.7076 (3.2318 - 13.922) pass
slope=2.251 min=-6.567 max=100.000 err=1441.548 R^2=0.8225

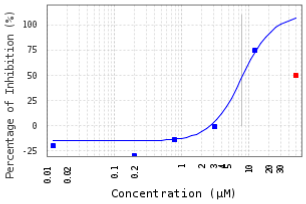

IC50: 7.8635 (0.0001 - 463979) pass
slope=1.857 min=-15.000 max=110.000 err=744.027 R^2=0.9101

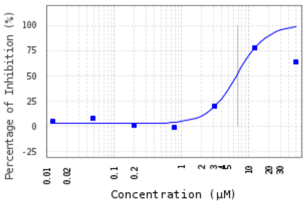

IC50: 6.7655 (3.3085 - 13.835) pass
slope=2.000 min=2.968 max=100.000 err=1239.459 R^2=0.8003

CEM-DNR (IC_{50} = 11.09 ± 0.959 µM)

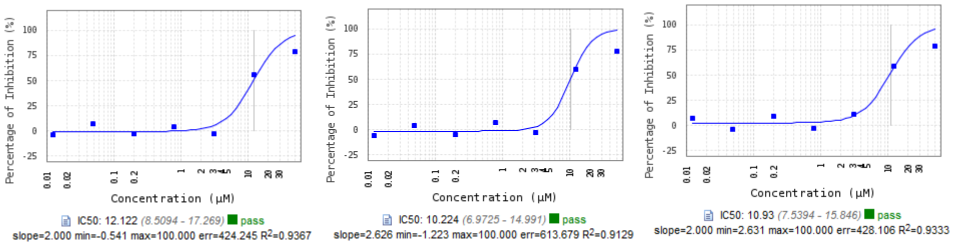

K562 (IC_{50} = 10.82 ± 0.431 µM)

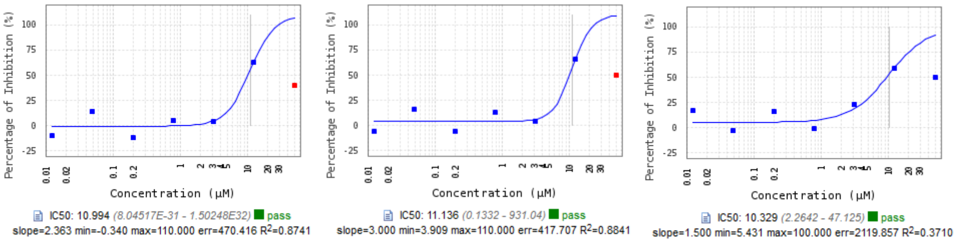

K562-TAX (IC_{50} = 14.85 ± 2.939 µM)

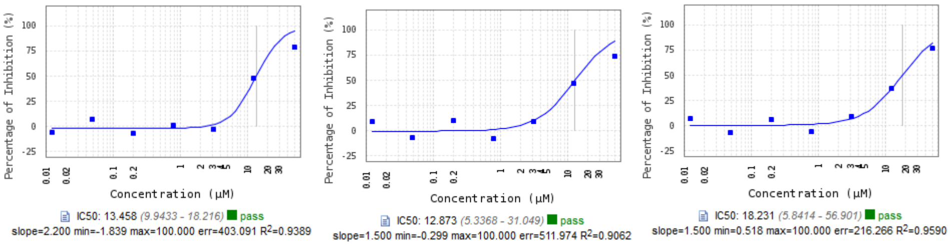

HCT116 (IC_{50} = 35.77 ± 5.909 µM)

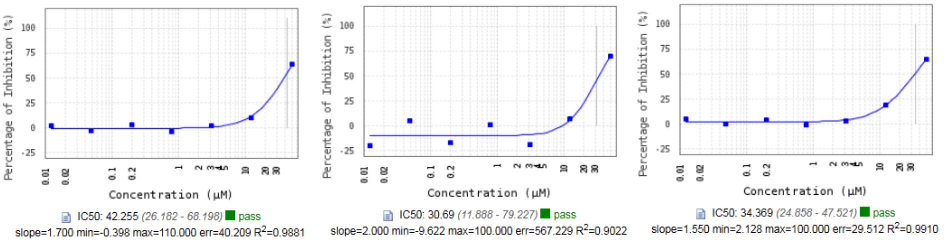

HCT116p53-/- (IC_{50} > 50 µM)

Primary screen (50 µM)

1 (Plate 1)	H12	LEM00014585	32.042		50.0uM	+	
1 (Plate 1)	H13	LEM00014585	55.826		50.0uM	+	
1 (Plate 1)	H14	LEM00014585	55.894		50.0uM	+	

1 (Plate 1)	H12	LEM00014585	33.022		50.0uM	+	
1 (Plate 1)	H13	LEM00014585	34.49		50.0uM	+	
1 (Plate 1)	H14	LEM00014585	21.548		50.0uM	+	

U2OS (IC$_{50}$ > 50 µM)

Primary screen (50 µM)

1 (Plate 1)	H12	LEM00014585	35.109		50.0uM	+		1 (Plate 1)	H12	LEM00014585	29.198		50.0uM	+	
1 (Plate 1)	H13	LEM00014585	25.286		50.0uM	+		1 (Plate 1)	H13	LEM00014585	20.852		50.0uM	+	
1 (Plate 1)	H14	LEM00014585	27.83		50.0uM	+		1 (Plate 1)	H14	LEM00014585	26.093		50.0uM	+	

MRC5 (IC$_{50}$ > 50 µM)

Primary screen (50 µM)

1 (Plate 1)	H12	LEM00014585	-2.5457		50.0uM	+		1 (Plate 1)	H12	LEM00014585	1.3878		50.0uM	+	
1 (Plate 1)	H13	LEM00014585	5.3743		50.0uM	+		1 (Plate 1)	H13	LEM00014585	3.4117		50.0uM	+	
1 (Plate 1)	H14	LEM00014585	-4.4619		50.0uM	+		1 (Plate 1)	H14	LEM00014585	4.7995		50.0uM	+	

BJ (IC$_{50}$ > 50 µM)

Primary screen (50 µM)

1 (Plate 1)	H12	LEM00014585	17.253		50.0uM	+		1 (Plate 1)	H12	LEM00014585	1.4224		50.0uM	+	
1 (Plate 1)	H13	LEM00014585	11.835		50.0uM	+		1 (Plate 1)	H13	LEM00014585	18.568		50.0uM	+	
1 (Plate 1)	H14	LEM00014585	23.187		50.0uM	+		1 (Plate 1)	H14	LEM00014585	26.839		50.0uM	+	

CB11

CCRF-CEM (IC$_{50}$ = 2.50 ± 0.567 µM)

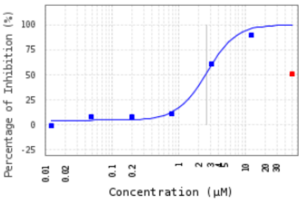

IC50: 2.6286 (2.1502 - 3.2134) pass
slope=2.000 min=4.731 max=100.000 err=89.250 R^2=0.9869

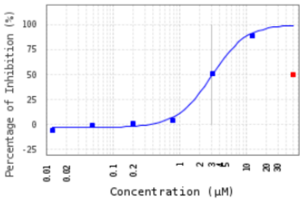

IC50: 2.9977 (2.4697 - 3.6385) pass
slope=1.673 min=-2.792 max=100.000 err=35.884 R^2=0.9951

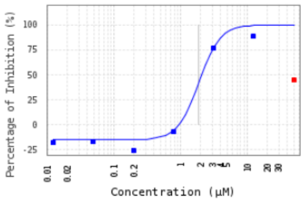

IC50: 1.8853 (1.3769 - 2.5815) pass
slope=2.765 min=-15.000 max=100.000 err=235.855 R^2=0.9823

CEM-DNR (IC$_{50}$ = 5.49 ± 0.935 µM)

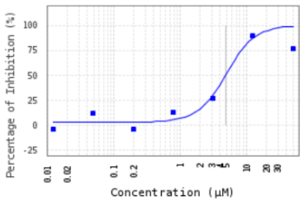

IC50: 4.9458 (2.9802 - 8.2079) pass
slope=2.000 min=3.065 max=100.000 err=761.009 R^2=0.9128

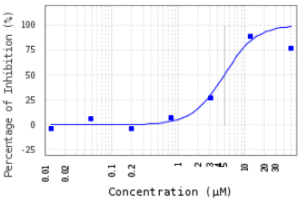

IC50: 4.959 (3.1067 - 7.9158) pass
slope=1.800 min=0.104 max=100.000 err=593.864 R^2=0.9338

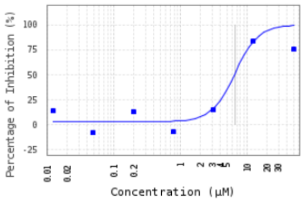

IC50: 6.5713 (3.3348 - 12.949) pass
slope=2.435 min=3.175 max=100.000 err=1036.795 R^2=0.8770

K562 (IC$_{50}$ = 1.85 ± 0.204 µM)

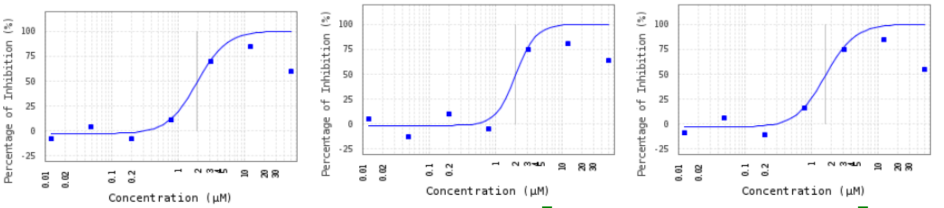

IC50: 1.9269 *(0.9119 - 4.0717)* pass
slope=2.000 min=-2.633 max=100.000 err=1919.563 R^2=0.7926

IC50: 2.0063 *(0.8181 - 4.9203)* pass
slope=2.950 min=-1.351 max=100.000 err=2073.896 R^2=0.7862

IC50: 1.6204 *(0.6815 - 3.8528)* pass
slope=2.000 min=-2.940 max=100.000 err=2415.562 R^2=0.7475

K562-TAX (IC$_{50}$ = 5.18 ± 0.198 µM)

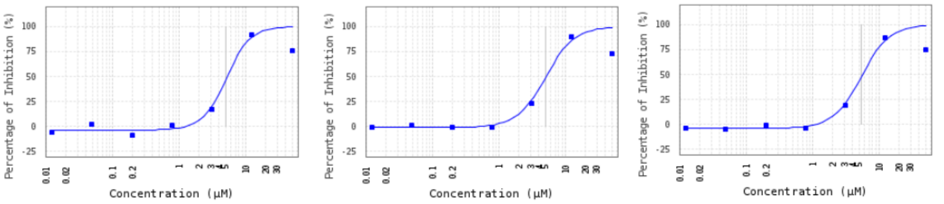

IC50: 5.1242 *(3.0094 - 8.7252)* pass
slope=2.400 min=-3.522 max=100.000 err=673.039 R^2=0.9344

IC50: 5.0291 *(2.8439 - 8.8933)* pass
slope=1.993 min=-1.136 max=100.000 err=702.025 R^2=0.9227

IC50: 5.4095 *(3.2107 - 9.1142)* pass
slope=2.023 min=-3.935 max=100.000 err=619.922 R^2=0.9344

HCT116 (IC$_{50}$ = 4.93 ± 0.729 µM)

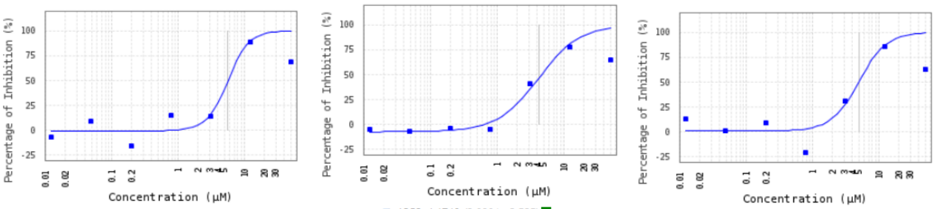

IC50: 5.6303 *(2.7982 - 11.329)* pass
slope=2.700 min=-0.475 max=100.000 err=1548.038 R^2=0.8306

IC50: 4.1748 *(2.0301 - 8.585)* pass
slope=1.400 min=-7.208 max=100.000 err=1122.601 R^2=0.8613

IC50: 4.9802 *(1.8687 - 13.272)* pass
slope=2.080 min=0.977 max=100.000 err=2052.080 R^2=0.7492

HCT116p53-/- (IC$_{50}$ = 5.52 ± 0.762 µM)

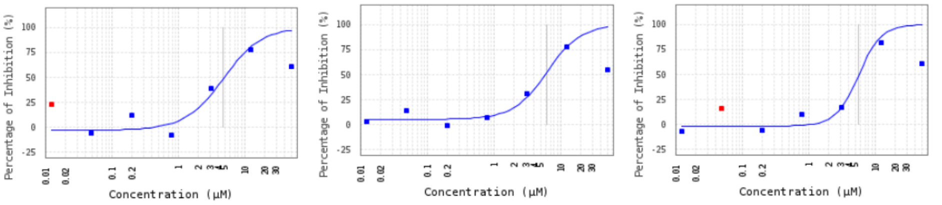

IC50: 4.7168 *(1.3942 - 15.957)* pass
slope=1.500 min=-2.721 max=100.000 err=1692.473 R^2=0.7295

IC50: 6.2341 *(2.3133 - 16.8)* pass
slope=1.700 min=5.489 max=100.000 err=1943.111 R^2=0.6287

IC50: 5.598 *(2.4459 - 12.812)* pass
slope=2.500 min=-1.536 max=100.000 err=1663.032 R^2=0.7555

U2OS (IC$_{50}$ = 4.62 ± 1.230 µM)

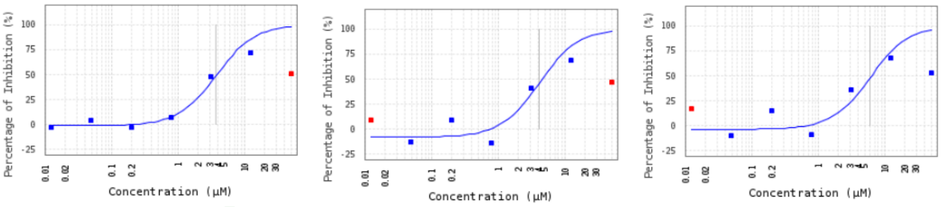

IC50: 3.7582 *(1.4695 - 9.6117)* pass
slope=1.500 min=-1.098 max=100.000 err=255.302 R^2=0.9485

IC50: 4.0771 *(0.4479 - 37.11)* pass
slope=1.500 min=-7.890 max=100.000 err=714.524 R^2=0.8596

IC50: 6.0295 *(1.3072 - 27.811)* pass
slope=1.500 min=-3.470 max=100.000 err=2455.024 R^2=0.5272

MRC5 (IC_{50} > 50 µM)

Primary screen (50 µM)

1 (Plate 1)	I12	LEM00014586	10.292	●	50.0uM	+	✓ ⊗
1 (Plate 1)	I13	LEM00014586	14.955	●	50.0uM	+	
1 (Plate 1)	I14	LEM00014586	7.8015	●	50.0uM	+	

1 (Plate 1)	I12	LEM00014586	9.9459	●	50.0uM	+	✓ ⊗
1 (Plate 1)	I13	LEM00014586	10.119	●	50.0uM	+	
1 (Plate 1)	I14	LEM00014586	11.392	●	50.0uM	+	

BJ (IC_{50} > 50 µM)

Primary screen (50 µM)

1 (Plate 1)	I12	LEM00014586	13.254	●	50.0uM	+	✓ ⊗
1 (Plate 1)	I13	LEM00014586	17.253	●	50.0uM	+	
1 (Plate 1)	I14	LEM00014586	17.189	●	50.0uM	+	

1 (Plate 1)	I12	LEM00014586	18.03	●	50.0uM	+	✓ ⊗
1 (Plate 1)	I13	LEM00014586	19.577	●	50.0uM	+	
1 (Plate 1)	I14	LEM00014586	22.535	●	50.0uM	+	

CB12

CCRF-CEM (IC_{50} = 5.54 ± 0.475 µM)

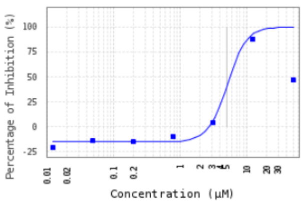

IC50: 5.1109 (2.4562 - 10.635) pass
slope=3.000 min=-15.000 max=100.000 err=2838.702 R^2=0.7147

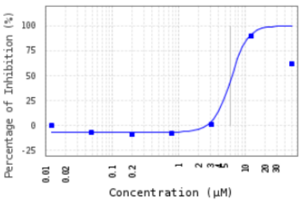

IC50: 6.0494 (2.8529 - 12.828) pass
slope=3.300 min=-6.519 max=100.000 err=1472.693 R^2=0.8493

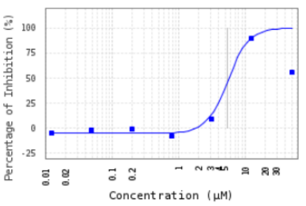

IC50: 5.4506 (2.1673 - 13.708) pass
slope=2.706 min=-4.929 max=100.000 err=1980.990 R^2=0.7667

CEM-DNR (IC_{50} = 36.03 ± 2.042 µM)

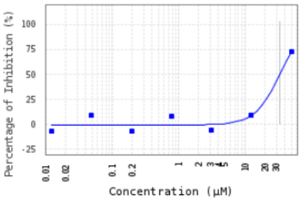

IC50: 33.982 (13.885 - 83.163) pass
slope=2.300 min=-0.470 max=102.764 err=273.160 R^2=0.9422

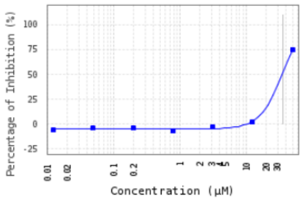

IC50: 36.027 (0.2780 - 4668.2) pass
slope=2.541 min=-4.598 max=110.000 err=7.849 R^2=0.9985

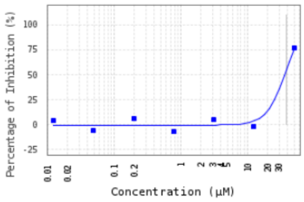

IC50: 38.066 (10.11 - 143.32) pass
slope=3.000 min=-0.301 max=110.000 err=186.538 R^2=0.9642

K562 (IC_{50} = 4.25 ± 0.890 µM)

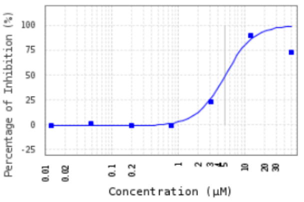

IC50: 5.0291 (2.8439 - 8.8933) pass
slope=1.993 min=-1.136 max=100.000 err=702.025 R^2=0.9227

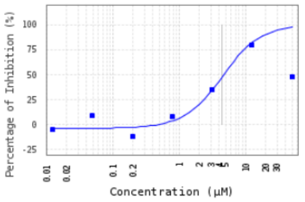

IC50: 4.4469 (1.2229 - 16.17) pass
slope=1.500 min=-3.507 max=100.000 err=2702.528 R^2=0.5803

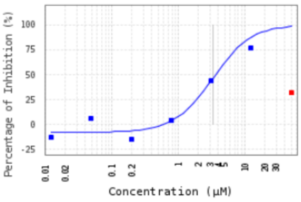

IC50: 3.2806 (1.3901 - 7.742) pass
slope=1.500 min=-7.786 max=100.000 err=393.981 R^2=0.9393

K562-TAX (IC$_{50}$ = 8.07 ± 0.706 μM)

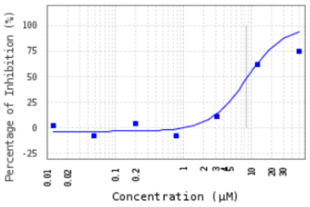

IC50: 8.5813 (4.7107 - 15.632) pass
slope=1.571 min=-3.249 max=100.000 err=534.301 R^2=0.9237

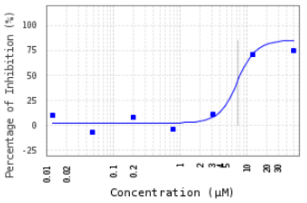

IC50: 7.2681 (5.3569 - 9.8612) pass
slope=3.000 min=2.468 max=85.000 err=316.837 R^2=0.9549

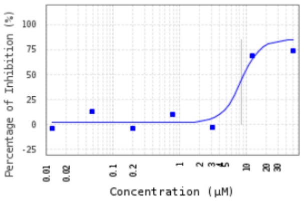

IC50: 8.3751 (6.1987 - 11.316) pass
slope=3.000 min=2.045 max=85.000 err=458.108 R^2=0.9344

HCT116 (IC$_{50}$ = 8.09 ± 0.268 μM)

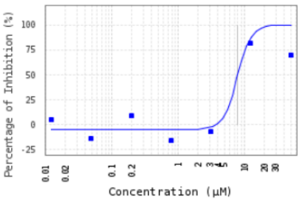

IC50: 7.8018 (2.4621 - 24.722) pass
slope=4.069 min=-4.722 max=100.000 err=1438.758 R^2=0.8526

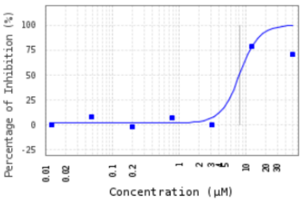

IC50: 8.1688 (4.8661 - 13.713) pass
slope=2.950 min=1.896 max=100.000 err=970.658 R^2=0.8697

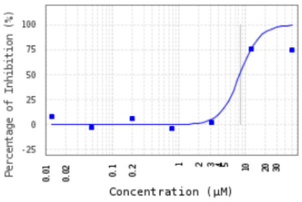

IC50: 8.321 (4.8488 - 14.28) pass
slope=2.874 min=0.025 max=100.000 err=752.865 R^2=0.9027

HCT116p53-/- (IC$_{50}$ = 8.35 ± 0.549 μM)

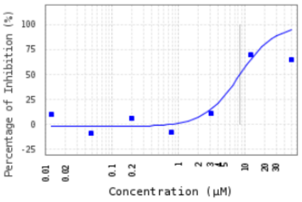

IC50: 8.3102 (3.4032 - 20.293) pass
slope=1.616 min=-1.917 max=100.000 err=1264.223 R^2=0.8027

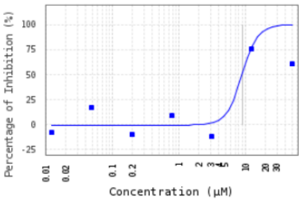

IC50: 8.9165 (2.514 - 31.624) pass
slope=3.618 min=-0.360 max=100.000 err=2257.682 R^2=0.6990

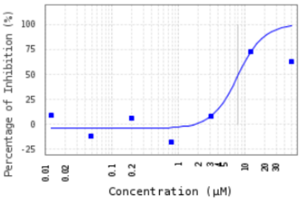

IC50: 7.8201 (3.6889 - 16.578) pass
slope=2.200 min=-3.733 max=100.000 err=1780.715 R^2=0.7613

U2OS (IC$_{50}$ = 9.12 ± 0.174 μM)

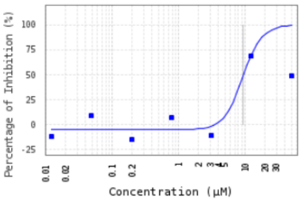

IC50: 9.2882 (4.5578 - 18.928) pass
slope=3.000 min=-5.010 max=100.000 err=3084.870 R^2=0.5195

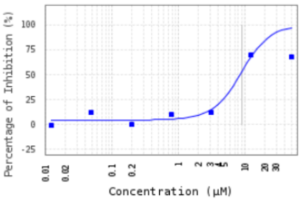

IC50: 8.94 (4.8443 - 16.498) pass
slope=2.000 min=4.572 max=100.000 err=992.513 R^2=0.8262

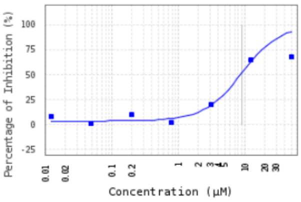

IC50: 9.1271 (4.0485 - 20.577) pass
slope=1.500 min=3.689 max=100.000 err=710.562 R^2=0.8593

MRC5 (IC$_{50}$ > 50 μM)

Primary screen (50 μM)

1 (Plate 1)	G12	LEM00014584	20.512		50.0uM	+	
1 (Plate 1)	G13	LEM00014584	17.701		50.0uM	+	
1 (Plate 1)	G14	LEM00014584	13.103		50.0uM	+	

1 (Plate 1)	G12	LEM00014584	15.439		50.0uM	+	
1 (Plate 1)	G13	LEM00014584	14.341		50.0uM	+	
1 (Plate 1)	G14	LEM00014584	20.47		50.0uM	+	

BJ (IC_{50} > 50 µM)

Primary screen (50 µM)

1 (Plate 1)	G12	LEM00014584	16.092		50.0uM	+
1 (Plate 1)	G13	LEM00014584	-1.1932		50.0uM	+
1 (Plate 1)	G14	LEM00014584	14.093		50.0uM	+

1 (Plate 1)	G12	LEM00014584	24.956		50.0uM	+
1 (Plate 1)	G13	LEM00014584	20.249		50.0uM	+
1 (Plate 1)	G14	LEM00014584	22.603		50.0uM	+

Fig. S22. Raw MTS cytotoxicity test data, dose-response curves used to calculate IC_{50}.

Tab. S3. Effect of cytotoxic compounds on cell cycle, apoptosis, mitosis and polyploidy in CCRF-CEM lymphoblasts (% of positive cells). Flow cytometry analysis was used for the quantification of cell cycle distribution and apoptotic cells. [a]phospho-Histone (Ser10).

Compound	Apoptosis	G_0/G_1	S	G_2/M	Mitosis[a]	Polyploid cells
**control**	3.68	42.96	25.81	31.24	1.54	7.77
**Col** 1×IC_{50}	13.67	2.61	16.84	80.55	58.35	14.45
**Col** 5×IC_{50}	11.47	4.73	24.71	70.57	64.48	14.73
**CB1** 1×IC_{50}	5.32	36.69	24.71	38.6	7.96	17.25
**CB1** 5×IC_{50}	11.73	50.26	1.72	48.02	17.85	19.14
**CB2** 1×IC_{50}	8.51	6.59	4.22	89.19	45.60	23.67
**CB2** 5×IC_{50}	13.84	5.2	1.29	93.51	79.51	26.18
**CB3** 1×IC_{50}	16.41	2.89	18.03	79.08	58.58	26.03
**CB3** 5×IC_{50}	20.64	7.84	8.15	84.01	57.12	26.25
**CB4** 1×IC_{50}	3.04	41.19	31.06	27.75	2.76	19.29
**CB4** 5×IC_{50}	12.24	5.49	1.83	92.68	64.43	23.72
**CB5** 1×IC_{50}	9.64	11.02	3.51	85.47	42.49	24.32
**CB5** 5×IC_{50}	10.96	5.27	0.77	93.96	60.70	26.07
**CB6** 1×IC_{50}	7.55	12.32	32.1	55.58	29.41	23.13
**CB6** 5×IC_{50}	14.32	3.72	1.88	94.4	61.11	24.76
**CB7** 1×IC_{50}	13.02	26.66	27.45	45.89	19.77	21.60
**CB7** 5×IC_{50}	14.67	6.62	4.54	88.84	68.32	23.77
**CB8** 1×IC_{50}	11.09	8.68	11.31	80.01	5.79	23.43
**CB8** 5×IC_{50}	3.98	9.49	3.17	87.34	13.33	20.18
**CB9** 1×IC_{50}	8.93	12.83	27.11	60.07	32.84	29.32
**CB9** 5×IC_{50}	14.27	19.56	31.53	48.91	31.00	26.30
**CB10** 1×IC_{50}	3.87	34.04	39.32	26.65	2.86	12.43
**CB10** 5×IC_{50}	11.26	8.21	29.17	62.62	36.02	25.51
**CB11** 1×IC_{50}	5.26	35.98	33.64	30.38	4.83	13.64
**CB11** 5×IC_{50}	16.42	10.48	10.23	79.29	59.86	24.88
**CB12** 1×IC_{50}	4.12	32.27	41.12	26.62	9.86	18.91
**CB12** 5×IC_{50}	10.47	21.05	25.39	53.56	23.11	23.39

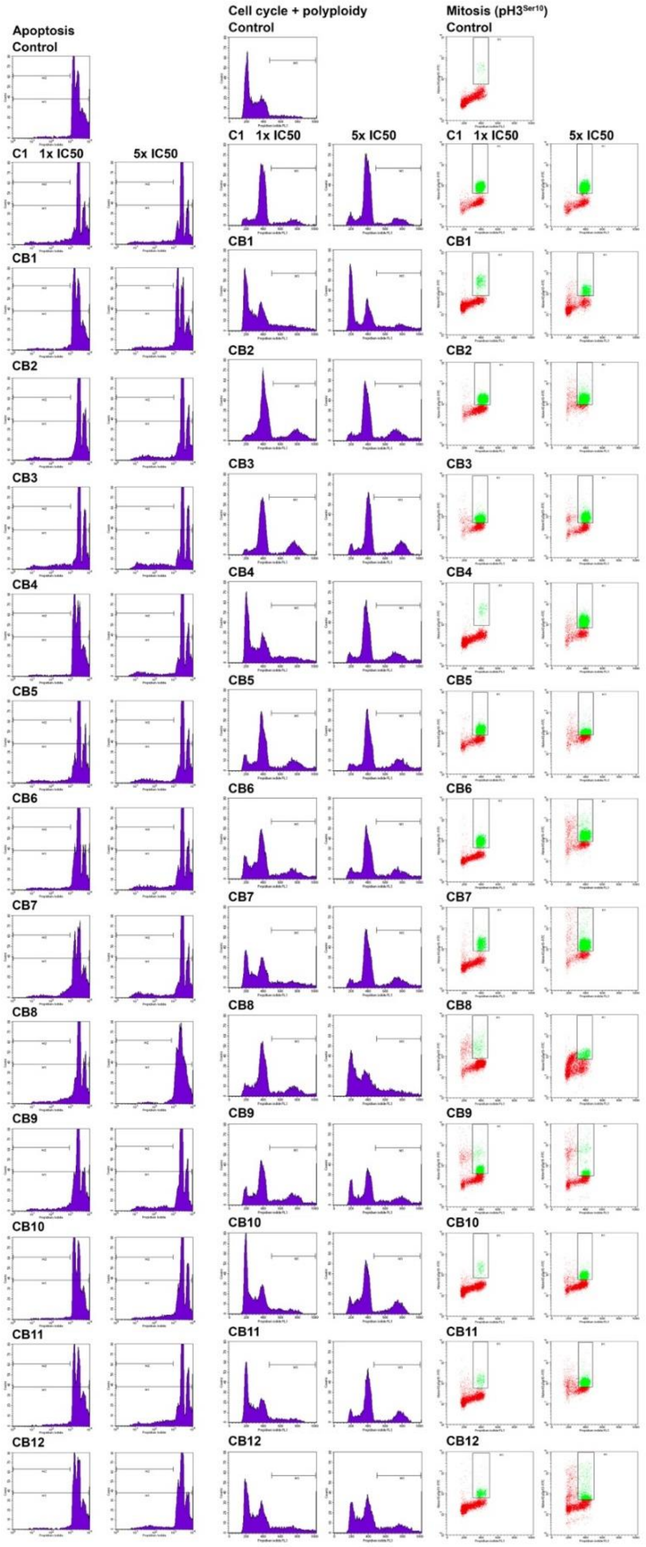

Fig. S23. Raw data of apoptosis, cell cycle, polyploidy and mitosis at 1× and 5× IC_{50} in CCRF-CEM lymphoblasts.

Tab. S4. Values of V_{max} from tubulin assay.

Compound	V_{max} (mOD/min)
**DMSO**	11.6 ± 1.5
**CB1**	10.4 ± 2.1
**CB2**	9.1 ± 3.2
**CB3**	6.5 ± 1.8
**CB4**	4.0 ± 1.0
**CB5**	9.5 ± 2.6
**CB6**	8.6 ± 1.4
**CB7**	9.4 ± 1.0
**CB9**	10.3 ± 1.0
**Col**	1.5 ± 0.8

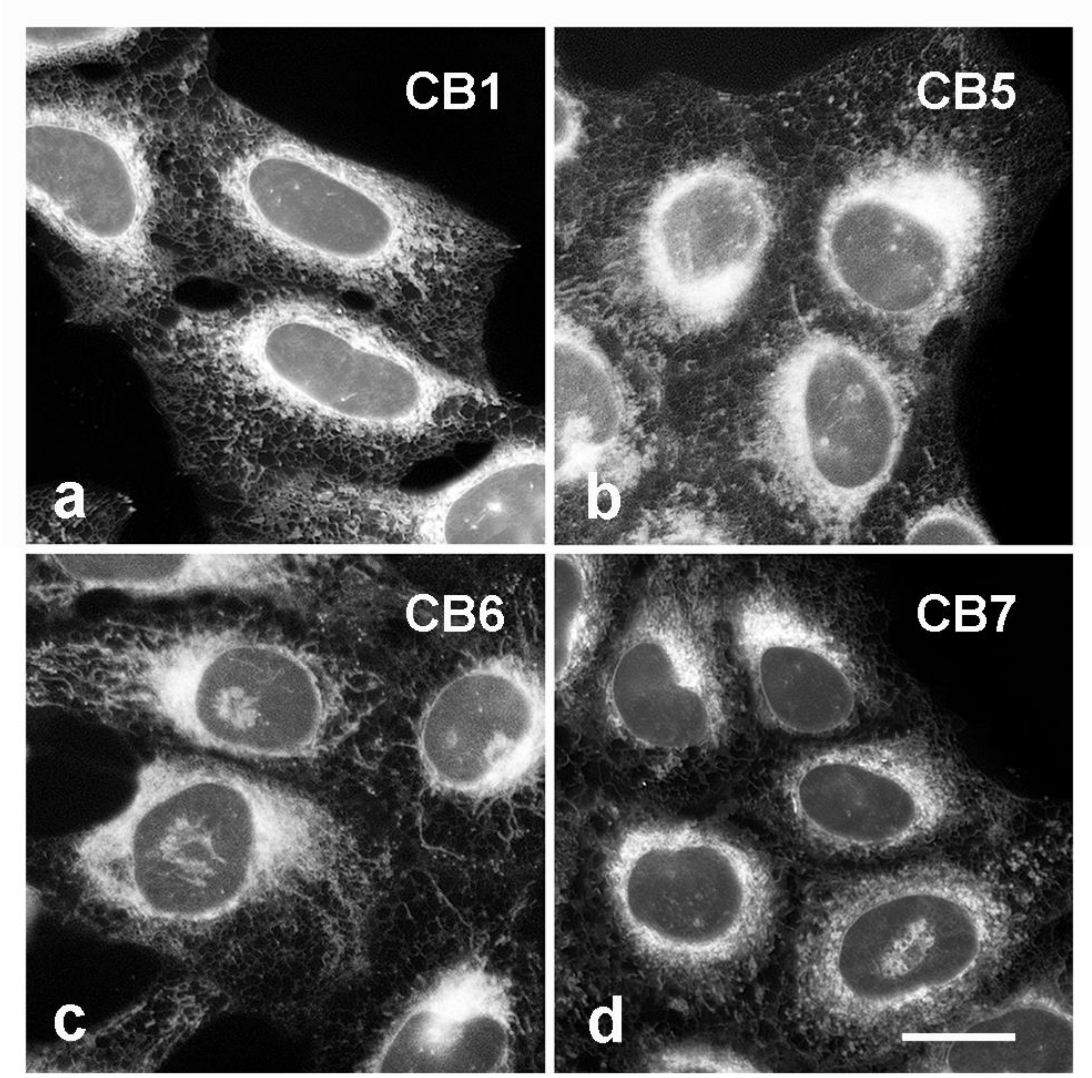

Fig. S24 Distribution of colchicine-BODIPY conjugates **CB1**, **CB5**, **CB6,** and **CB7** in living cells. U2OS cells were incubated with the studied compounds (1 μM) for 5 min at 37 °C and evaluated by live-cell imaging. Scale bar for **a-d**, 20 μm.

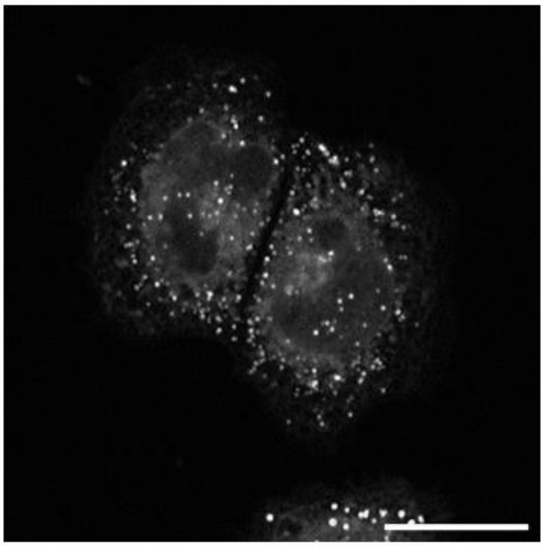

Fig. S25. Distribution of BODIPY moiety **B9** in living cells. U2OS cells were incubated for 5 min at 37 °C with **B9** at a concentration of 1 μM and evaluated by live-cell imaging. Scale bar, 20 μm.

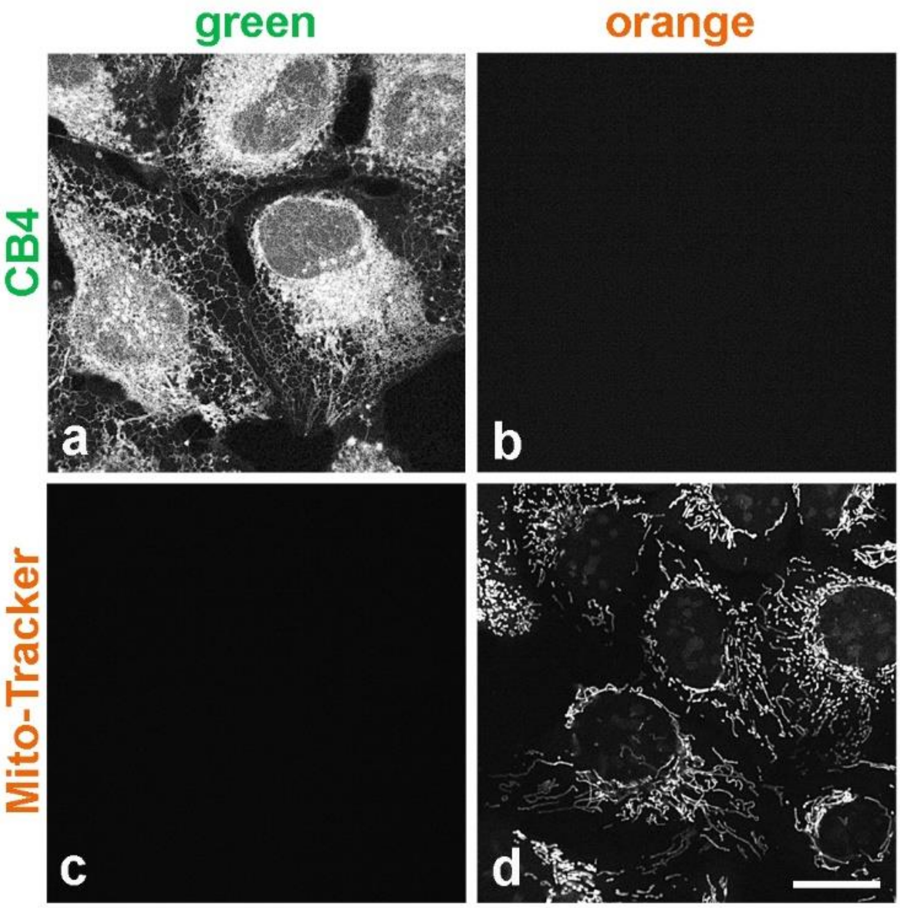

Fig. S26. Distribution of CB4 and Mito-Tracker in living cells. U2OS with green CB4 (**a-b**) or orange Mito-Tracker (**c-d**) were evaluated in green and orange channels. The images (**a-b**) or (**c-d**) were acquired and processed in the same way. See no spectral overlap. Scale bar for **a-d**, 20 μm.

3. Molecular docking

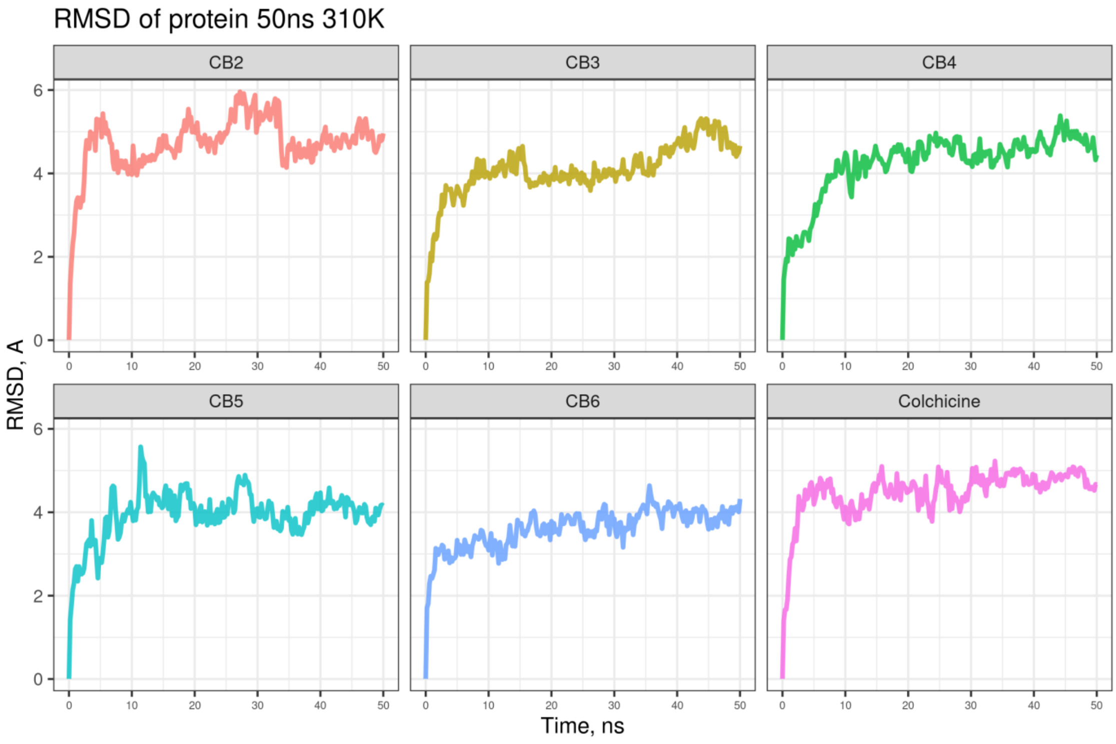

Fig. S27. RMSD of tubulin from 50 ns MD simulations of its complexes with compounds CB2-CB6 and colchicine.

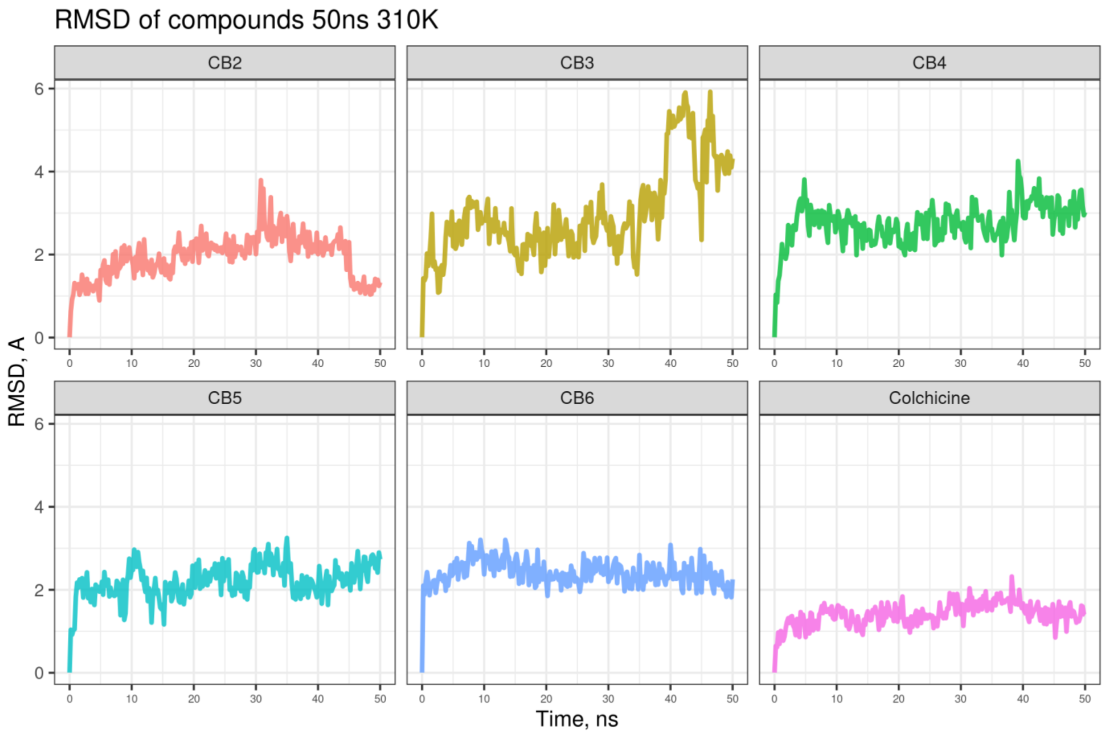

Fig. S28. RMSD of compounds CB2-CB6 and colchicine from 50 ns MD simulations of their complexes with tubulin.

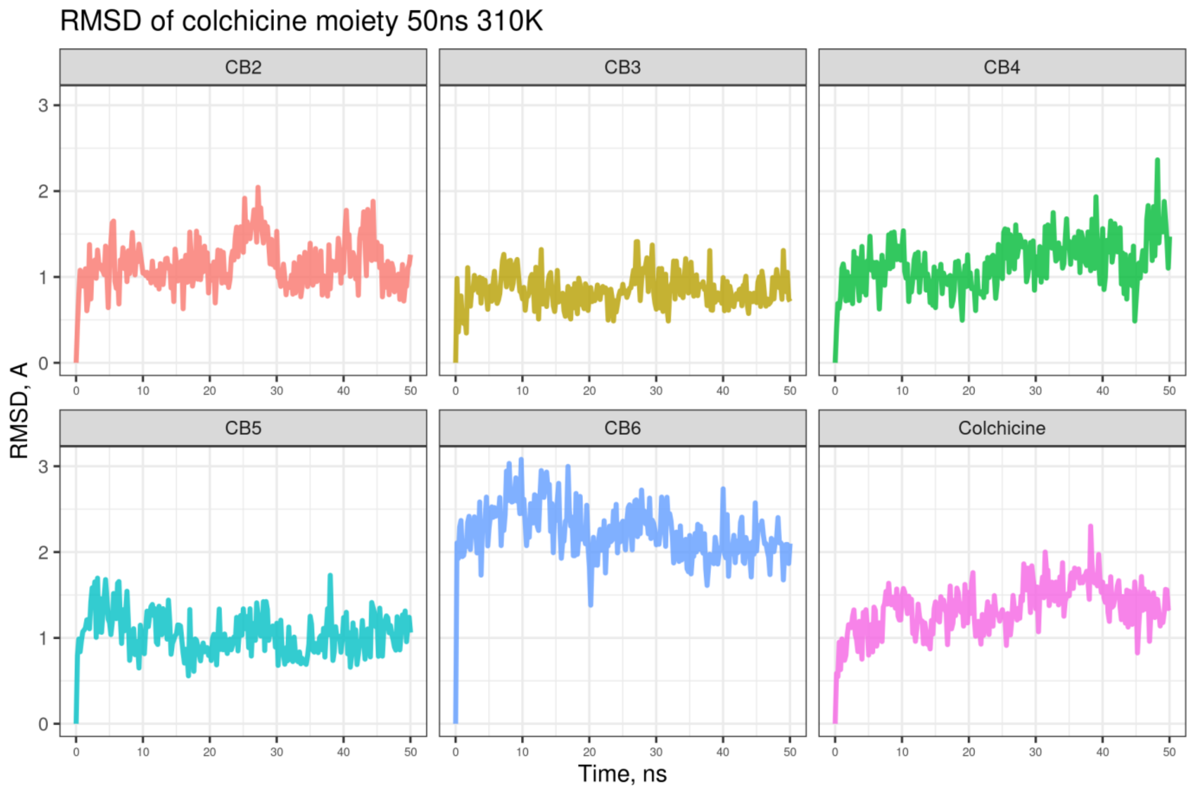

Fig. S29. RMSD of the colchicine moiety of compounds CB2-CB6 and colchicine from 50 ns MD simulations of their complexes with tubulin.

<table>
<tr>
<td>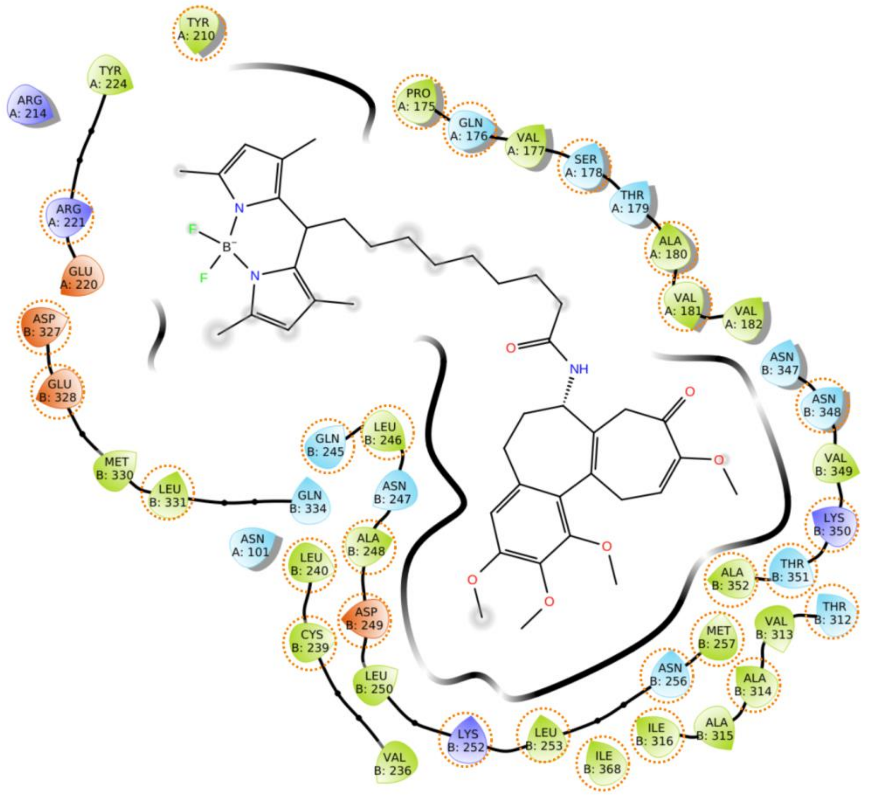
</td>
<td>CB2</td>
</tr>
<tr>
<td>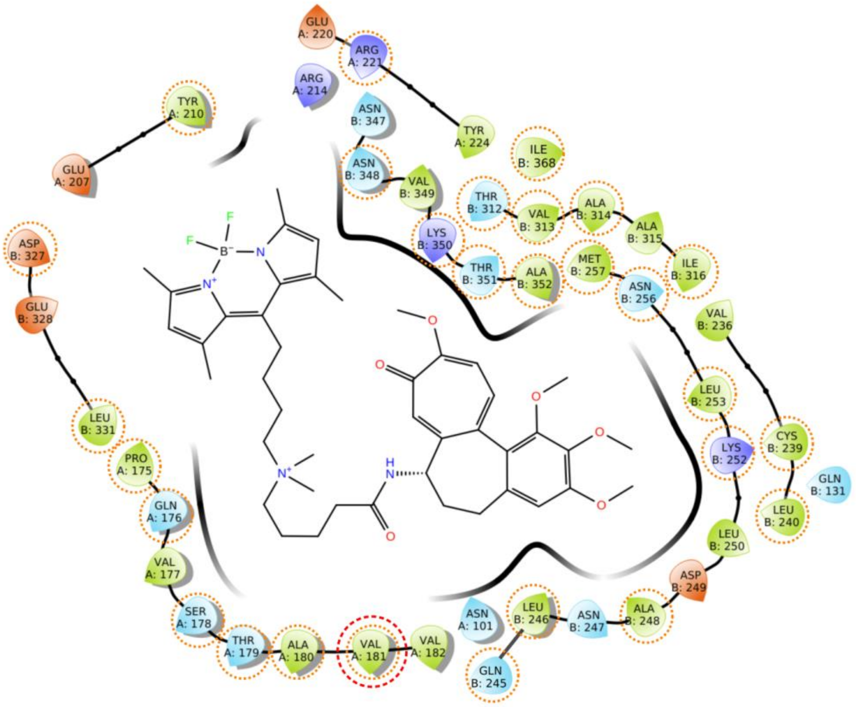
</td>
<td>CB3</td>
</tr>
</table>

<table>
<tr><td>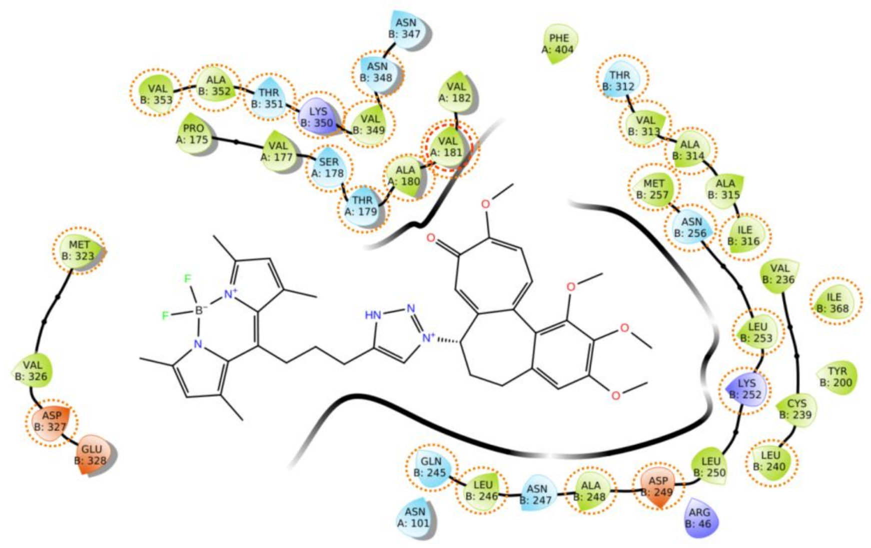
</td><td>CB4</td></tr>
<tr><td>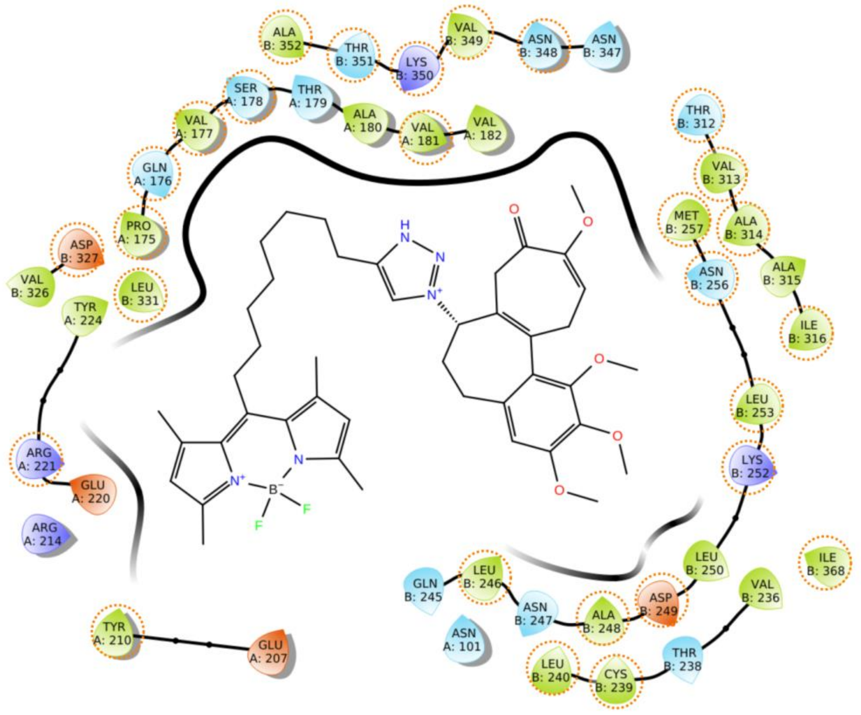
</td><td>CB5</td></tr>
</table>

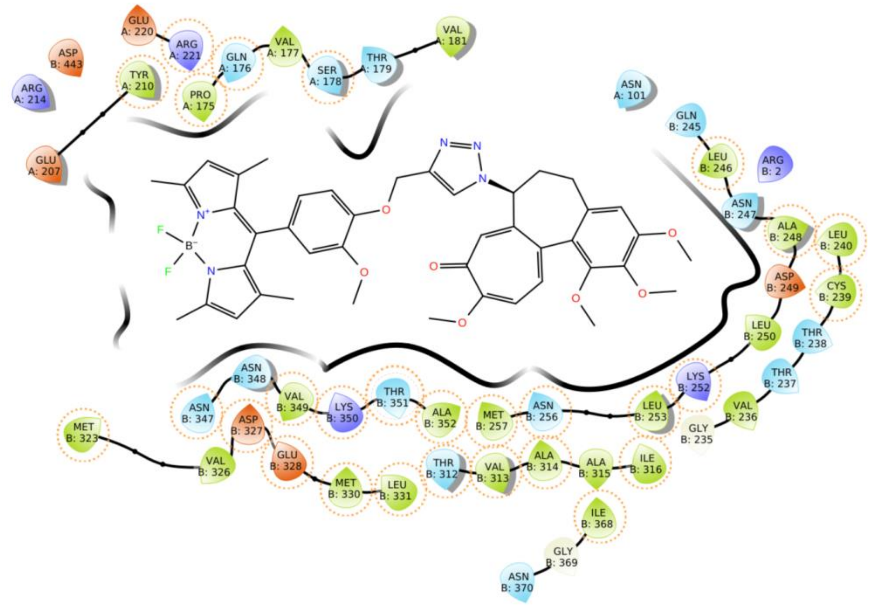	CB6
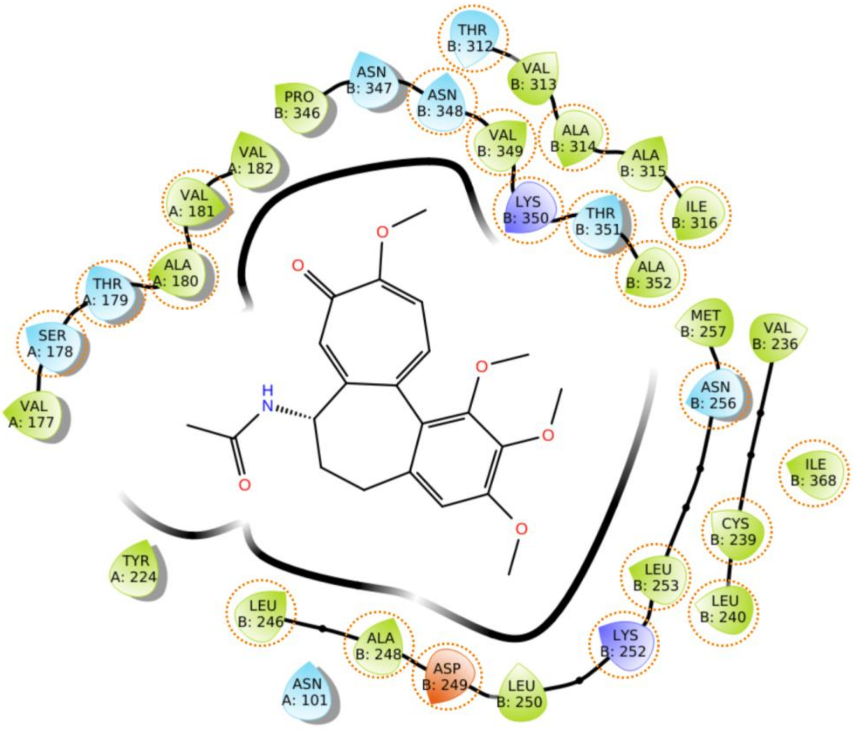	Colchicine
Charged (negative) Charged (positive) Hydrophobic Polar Solvent exposure	

Fig S30. 2D plots of protein-ligand interaction patterns of colchicine and compounds CB2-CB6

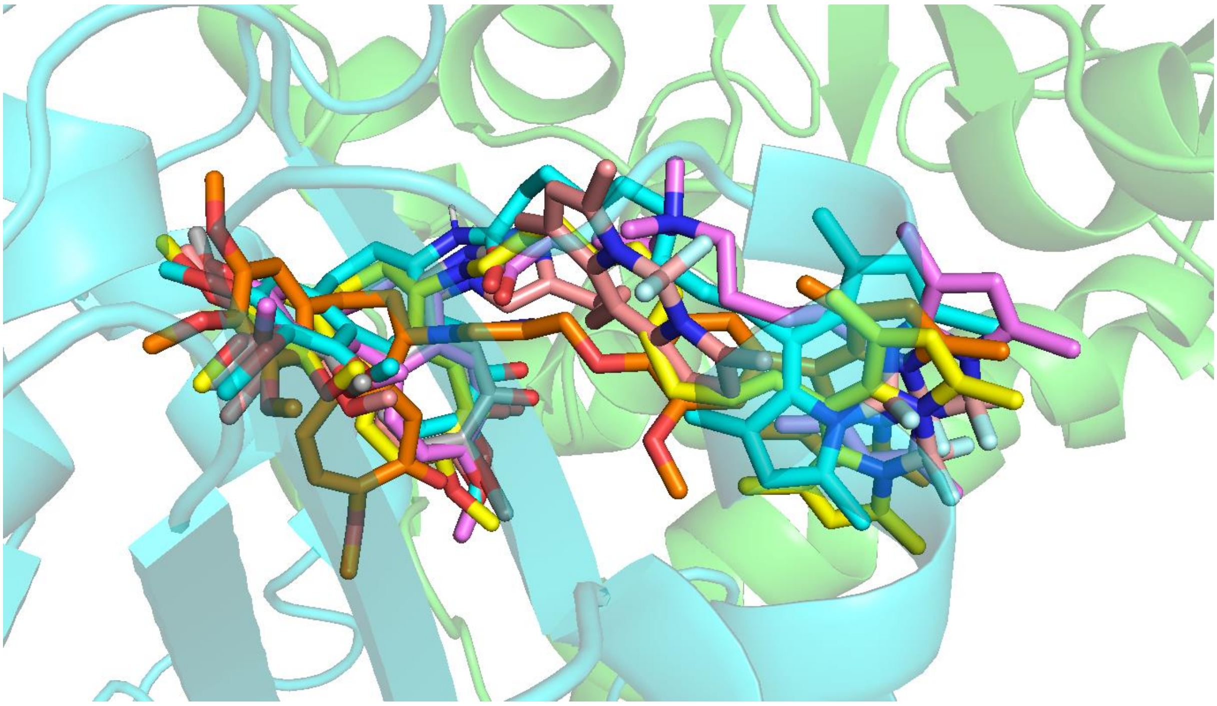

Fig S31. Binding poses of colchicine (grey), CB2 (cyan), CB3 (magenta), CB4 (pink), CB5 (yellow) and CB6 (orange).
